# Status and implications of the knowledge, attitudes and practices towards AWaRe antibiotic use, resistance and stewardship among low- and middle-income countries

**DOI:** 10.1093/jacamr/dlaf033

**Published:** 2025-03-25

**Authors:** Zikria Saleem, Catrin E Moore, Aubrey C Kalungia, Natalie Schellack, Olayinka Ogunleye, Audrey Chigome, Kona Chowdhury, Freddy Eric Kitutu, Amos Massele, Nishana Ramdas, E Sam Orubu, Aislinn Cook, Felix Khuluza, Trust Zaranyika, Elisa Funiciello, Giulia Lorenzetti, Miriam Nantamu, Ayuska Parajuli, Amanj Kurdi, Hellen Nabayiga, Ammar Abdulrahman Jairoun, Mainul Haque, Stephen M Campbell, Dena Van Der Bergh, Brian Godman, Mike Sharland

**Affiliations:** Department of Pharmacy Practice, Faculty of Pharmacy, Bahauddin Zakariya University, Multan 60800, Pakistan; Centre for Neonatal and Paediatric Infection, Institute for Infection and Immunity, City St George’s, University of London, London SW17 0RE, UK; Department of Pharmacy, School of Health Sciences, University of Zambia, Lusaka P.O. Box 50110, Zambia; Department of Pharmacology, Faculty of Health Sciences, University of Pretoria, Pretoria 0084, South Africa; Department of Pharmacology, Therapeutics and Toxicology, Lagos State University College of Medicine, Ikeja, Lagos 100271, Nigeria; Department of Medicine, Lagos State University Teaching Hospital, Ikeja 100271, Nigeria; Department of Public Health Pharmacy and Management, School of Pharmacy, Sefako Makgatho Health Sciences University, Ga-Rankuwa 0208, South Africa; Department of Pediatrics, Gonoshasthaya Somaj Vittik Medical College, Dhaka 1344, Bangladesh; Sustainable Pharmaceutical Systems (SPS) Unit, School of Health Sciences, Makerere University, PO Box 7072, Kampala, Uganda; Department of Women’s and Children’s Health, International Maternal and Child Health (IMCH), Uppsala University, SE-751 85 Uppsala, Sweden; Department of Pharmacy, Makerere University School of Health Sciences, Kampala, Uganda; Department of Clinical Pharmacology and Therapeutics, Hurbert Kairuki Memorial University, 70 Chwaku Road Mikocheni, Dar Es Salaam P.O. Box 65300, Tanzania; Department of Public Health Pharmacy and Management, School of Pharmacy, Sefako Makgatho Health Sciences University, Ga-Rankuwa 0208, South Africa; Department of Biomedical Engineering, Boston University College of Engineering, Boston, MA, USA; Institute for Health System Innovation & Policy, Boston University, Boston, MA, USA; Centre for Neonatal and Paediatric Infection, Institute for Infection and Immunity, City St George’s, University of London, London SW17 0RE, UK; Health Economics Research Centre, Nuffield Department of Population Health, University of Oxford, Oxford OX1 2JD, UK; Pharmacy Department, Formerly College of Medicine, Kamuzu University of Health Sciences (KUHeS), Blantyre P.O. Box 278, Malawi; Faculty of Medicine and Health Sciences, University of Zimbabwe, Harare P.O. Box MP167, Zimbabwe; Centre for Neonatal and Paediatric Infection, Institute for Infection and Immunity, City St George’s, University of London, London SW17 0RE, UK; Centre for Neonatal and Paediatric Infection, Institute for Infection and Immunity, City St George’s, University of London, London SW17 0RE, UK; Centre for Neonatal and Paediatric Infection, Institute for Infection and Immunity, City St George’s, University of London, London SW17 0RE, UK; HERD International, Lalitpur, Nepal; Public Health Research Society, Kathmandu, Nepal; Department of Public Health Pharmacy and Management, School of Pharmacy, Sefako Makgatho Health Sciences University, Ga-Rankuwa 0208, South Africa; Department of Clinical Pharmacy, College of Pharmacy, Hawler Medical University, Kurdistan Regional Governorate, Erbil, Iraq; Strathclyde Institute of Pharmacy and Biomedical Science (SIPBS), University of Strathclyde, Glasgow G4 0RE, UK; College of Pharmacy, Al-Kitab University, Kirkuk 36015, Iraq; Management Science Department, Strathclyde Business School, University of Strathclyde, 199 Cathedral Street, Glasgow G4 0QU, UK; Health and Safety Department, Dubai Municipality, Dubai, United Arab Emirates; Center of Medical and Bio-Allied Health Sciences Research, Ajman University, Ajman 346, United Arab Emirates; Department of Clinical Sciences, College of Pharmacy and Health Sciences, Ajman University, Ajman 346, United Arab Emirates; Unit of Pharmacology, Faculty of Medicine and Defence Health, Universiti Pertahanan Nasional Malaysia (National Defence University of Malaysia), Kem Sungai, Besi, 57000 Kuala Lumpur, Malaysia; Karnavati Scientific Research Center, Karnavati School of Dentistry, Karnavati University, Gandhinagar 382 422, Gujarat, India; Department of Public Health Pharmacy and Management, School of Pharmacy, Sefako Makgatho Health Sciences University, Ga-Rankuwa 0208, South Africa; School of Health Sciences, University of Manchester, Manchester, UK; Division of Infectious Diseases and HIV Medicine, Department of Medicine, Groote Schuur Hospital, University of Cape Town, Cape Town, South Africa; Centre for Neonatal and Paediatric Infection, Institute for Infection and Immunity, City St George’s, University of London, London SW17 0RE, UK; Department of Public Health Pharmacy and Management, School of Pharmacy, Sefako Makgatho Health Sciences University, Ga-Rankuwa 0208, South Africa; Strathclyde Institute of Pharmacy and Biomedical Science (SIPBS), University of Strathclyde, Glasgow G4 0RE, UK; Center of Medical and Bio-Allied Health Sciences Research, Ajman University, Ajman 346, United Arab Emirates; Centre for Neonatal and Paediatric Infection, Institute for Infection and Immunity, City St George’s, University of London, London SW17 0RE, UK

## Abstract

**Background:**

There are concerns globally with rising rates of antimicrobial resistance (AMR), particularly in low- and middle-income countries (LMICs). AMR is driven by high rates of inappropriate prescribing and dispensing of antibiotics, particularly Watch antibiotics. To develop future interventions, it is important to document current knowledge, attitudes and practices (KAP) among key stakeholder groups in LMICs.

**Methods:**

We undertook a narrative review of published papers among four WHO Regions including African and Asian countries. Relevant papers were sourced from 2018 to 2024 and synthesized by key stakeholder group, country, WHO Region, income level and year. The findings were summarized to identify pertinent future activities for all key stakeholder groups.

**Results:**

We sourced 459 papers, with a large number coming from Africa (42.7%). An appreciable number dealt with patients’ KAP (33.1%), reflecting their influence on the prescribing and dispensing of antibiotics. There was marked consistency of findings among key stakeholder groups across the four WHO Regions, all showing concerns with high rates of prescribing of antibiotics for viral infections despite professed knowledge of antibiotics and AMR. There were similar issues among dispensers. Patients’ beliefs regarding the effectiveness of antibiotics for self-limiting infectious diseases were a major challenge, although educational programmes did improve knowledge. The development of the AWaRe (Access, Watch and Reserve) system, including practical prescribing guidance, provides a future opportunity for the standardization of educational inputs.

**Conclusions:**

Similar KAP regarding the prescribing and dispensing of antibiotics across LMICs and stakeholder groups presents clear opportunities for standardization of educational input and practical training programmes based on the AWaRe system.

## Introduction

Antimicrobial resistance (AMR) increases morbidity, mortality and healthcare costs, and is now seen as a serious public health concern.^1–4^ If not addressed, AMR could cause or exacerbate the next pandemic.^5,6^ Whereas AMR is ubiquitous, the burden is disproportionately higher in low- and middle-income countries (LMICs), which currently account for 80% of an estimated 10 million deaths annually attributable to AMR.^7–10^ AMR levels will continue increasing in LMICs unless addressed, with current high rates of AMR exacerbated by the growing use of antibiotics from the WHO’s Watch list, with resultant implications for increasing rates of MDR bacteria.^11,12^ The classification of antibiotics into Access, Watch and Reserve (AWaRe) groups in the WHO Essential Medicines List was part of global initiatives to reduce inappropriate use of Watch and Reserve antibiotics with their greater potential for selection of antibiotic resistance (ABR).^11,13,14^ Alongside this, across LMICs including African countries, there is continued inappropriate prescribing and dispensing of antibiotics generally, including among children, exacerbated by limited knowledge and awareness of AMR.^11,15–21^

A number of global and regional initiatives have been introduced in recent years to try to minimize rising rates of ABR and reduce its consequences.^22–25^ These include the WHO’s Global Action Plan (GAP) to reduce AMR, translated into National Action Plans (NAPs) across member states.^22,26^ Other initiatives include the classification of antibiotics into AWaRe groups as well as the WHO AWaRe antibiotic book guiding optimal use of antibiotics in 35 infections in adults and children for both the primary care and hospital settings, published in 2022.^14,27,28^ However, there are currently major concerns with the implementation of NAPs, especially among LMICs, including among African and Asian countries, given the demands on available personnel and resources.^29–33^ Similar concerns exist regarding national and local implementation of the guidance included within the WHO AWaRe book.^34–36^

The greatest use of antibiotics in humans in LMICs is in primary care, which can account for up to 90% of total antibiotic use in humans and consists of mainly older multiple sourced oral Access and Watch antibiotics.^37^ Consequently, strategies aimed at reducing inappropriate antibiotic use, and consequently ABR, among patients in LMICs should principally be focused on primary care. Primary care includes hospital outpatients as well as community pharmacies and drugstores in addition to primary care clinics in the community. These strategies should be aimed at reducing considerable rates of inappropriate prescribing and dispensing of antibiotics that currently takes place in primary care across LMICs, especially for self-limiting conditions such as minor upper respiratory tract infections (URTIs).^9,15–17,38^ Physicians and other prescribers in LMICs often prescribe antibiotics for self-limiting conditions given severe time pressures, and the resultant insufficient time to fully diagnose patients, combined with pressure from patients to prescribe antibiotics.^9,15,39,40^ Within primary care in LMICs, nurses and other healthcare professionals (HCPs), including clinical officers, are also often involved alongside physicians in the prescribing of antibiotics in public healthcare clinics (PHCs).^41–44^ Similar to physicians, there have been concerns with their knowledge and prescribing practices regarding antibiotics.^45–47^

Many people across LMICs also self-purchase antibiotics without a prescription for themselves or their children in both the formal and informal sectors despite legislation.^16,17,38^ High rates of self-purchasing are driven by a number of factors.^16,17,48^ Key factors include high patient copayments, limited knowledge of antibiotics, long waiting times to see HCPs in PHCs, shortages of pertinent medicines in clinics, as well as the convenience and availability of community pharmacies, drugstores and drug sellers especially in rural areas.^16,17,49–51^ There are though appreciable concerns regarding the knowledge and communication skills of pharmacists and their assistants, as well as among informal drug sellers, with respect to ABR and AMR.^17,52–54^ However, pharmacy personnel can play a key public health role in improving antibiotic use in the community, which builds on their role during the recent COVID-19 pandemic.^55–57^ The increased role of community pharmacists is reflected by some countries, including LMICs, allowing pharmacists to dispense certain antibiotics without a prescription.^17,58,59^ The lack of knowledge regarding antibiotics, their use, ABR and AMR, as well as key aspects surrounding antimicrobial stewardship, among physicians, pharmacists, nurses and other HCPs, usually starts in college or university and can continue post qualification unless adequately addressed.^17,60–62^ This is a particular concern in LMICs compared with high-income countries such as the UK.^62,63^

As mentioned, patients and clients also play an appreciable role in LMICs regarding the overuse and misuse of antibiotics through requesting or suggesting antibiotics to HCPs, including pharmacists, for often self-limiting conditions.^15,64–67^ Requests for antibiotics are often driven by limited knowledge of antibiotics and ABR.^15,65,66^ Educational campaigns can be effective in LMICs to improve the knowledge of patients and reduce their requests for antibiotics for self-limiting viral conditions, although there are challenges.^68–70^ There can also be language barriers to full comprehension, as well as a lack of a specific word or language about infection and its treatment in LMICs, which can make it difficult for pharmacists and their assistants to fully convey concepts such as the actions of antibiotics, ABR and AMR, unless they are aware of possible language issues in advance in their deliberations with patients.^70–72^

Antimicrobial stewardship programmes (ASPs) are being increasingly encouraged across sectors, including primary care, to improve future antibiotic use.^73–77^ There have been challenges with developing ASPs in LMICs because of resource and personnel issues.^77,78^ However, this is changing across LMICs and sectors, enhanced by groups such as the British Society of Antimicrobial Chemotherapy, the WHO, the Fleming Fund and the Open University providing high-quality training on AMR and ASPs as well as improving connectivity, with published ASPs in primary care also providing exemplars.^68,78–87^ There is also growing use of hub and spoke models as part of ASPs, and this will continue.^88^

We are also seeing the growing use of quality indicators across LMICs, including adherence to current guidelines, to improve antibiotic use across sectors.^81,89–92^ The use of quality indicators to improve future antibiotic use is likely to accelerate with the recent publication of the WHO AWaRe book providing treatment guidance for a range of common infections.^14,93,94^ There can though be challenges with the introduction, implementation and monitoring of quality indicators in LMICs.^95^

Consequently, given rising AMR rates across LMICs, there is a need to ascertain the current situation regarding key stakeholder knowledge, attitudes and practices (KAP) towards antibiotics, AMR and ASPs in primary care. Current KAPs are important as misinformation or poor attitudes and knowledge towards antibiotics and AMR, including lack of knowledge regarding local AMR patterns and the implications for antibiotic dosing regimens, as well as concerns with language, will further increase AMR unless addressed.^70,96,97^ We recognize it is challenging to change attitudes as well as prescribing and dispensing practices; however, this is changing.^17,71,81,82^ Changes in attitudes and practices are important to achieve the United Nation General Assembly (UNGA-AMR) goals to increase the use of Access antibiotics to 70% across sectors, and combined with other activities to reduce AMR.^98^

As a result, the objective of this narrative review is to document current KAP among all key stakeholder groups in LMICs where AMR is a particular concern. Subsequently, to use the findings to provide future direction to key policy makers, academics, HCPs and others to improve future antibiotic use in primary care in LMICs, including potential behavioural interventions.^83^ Instigating activities to improve antibiotic use in LMICs builds on the WHO GAP initiative in 2015 to raise awareness and encourage best practices among HCPs, policy makers and the public through undertaking surveys using instruments provided by the WHO.^99^ In this study, we used a narrative review to understand KAP surrounding AMR and the use of antibiotics in four critical WHO Regions during the past 7 years (2018–2024).

## Methodology

### Study design

A narrative review approach was undertaken for this study, similar to other reviews of KAP studies and their implications among LMICs.^65^ The rationale for our adopted approach was to allow for a broader scope of sourced papers given that information contained within identified papers may be part of a broader paper, whose insight and findings may be missed when undertaking a systematic review. Examples include, for instance, knowledge and attitudes towards antibiotics, ABR and ASPs among patients visiting community pharmacies or drugstores for antibiotics to treat their infection without initially seeking help from physicians or nurses.^17,38,100^ In addition, parts of studies can be included, for example, when assessing point-of-care testing for infections,^101^ or adherence rates to current standard treatment guidelines (STGs);^102,103^ alternatively, evaluating the impact of ASPs in ambulatory care where pertinent.^104^ The greater flexibility and coverage of possible papers also helps provide robust future direction to all key stakeholder groups. LMICs were chosen since, as mentioned, they face the greatest challenges with ABR and AMR, particularly for African and Asian countries.^1,105^

This narrative review builds on previously published systematic reviews assessing KAPs of key stakeholder groups, which have included studies published appreciably earlier than 2018.^61,70,96,106–108^ As a result, this review focuses on more up-to-date findings, not just on antibiotic use but also surrounding ABR, AMR and AMS in one comprehensive review. We have adopted similar approaches previously when documenting, as well as suggesting, activities to improve the care of patients with infectious disease across sectors in both Africa and Asia to minimize AMR.^17,29,91,109^ Consequently, we believed this approach was more suitable for this comprehensive review, and summarizing the resulting implications, as the principal aim of this paper is to suggest potential ways forward for all key stakeholder groups to improve their KAP surrounding antibiotics, AMR and ASPs, besides acknowledging the difficulties involved with improving future prescribing and dispensing of antibiotics in LMICs. The suggestions will be based on the considerable experiences of the co-authors, similar to other narrative reviews undertaken by the group.^17,91,109,110^

A number of databases were initially searched by the corresponding author (B.G.), including Google Scholar and PubMed/MEDLINE for published papers between January 2018 and October 2024. Search terms included ambulatory care; antibiotics; antibiotic prescribing; primary health facilities; primary healthcare centres; community pharmacists; patients; antimicrobial stewardship; antimicrobial stewardship programs; and KAP among the targeted African and Asian countries within the four key WHO Regions. These are the WHO African, Eastern Mediterranean, South-East Asia and Western Pacific Regions.^111^ The references of sourced papers, especially those published from January 2019 to October 2024, were also examined for potential additional references that could be included in the narrative review to enhance its comprehensive nature. The co-authors from across the four WHO Regions also provided additional relevant papers to enhance the review. As a measure of quality, only the sourced papers that are cited in PubMed are included in the tables.

**Figure 1.**
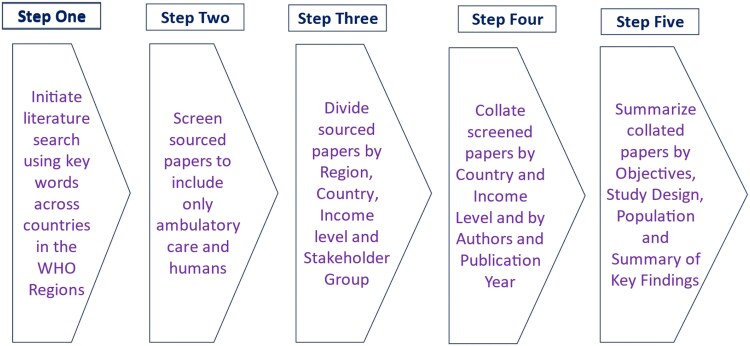
Synopsis of the literature review methodology.

Sourced papers concerning KAP regarding antibiotics, ABR, AMR and AMS in primary care, including hospital outpatients, were divided into the four key WHO Regions for comparative purposes (Figure 1). After this, the results were further divided into individual countries in each WHO Region according to their current World Bank economic status (Figure 1). The current economic status includes three groups: low-, low-middle- and upper-middle-income countries.^112^ This breakdown has been undertaken since there can be differences in utilization patterns and challenges between income levels due to many issues including access and affordability of care.^15^ The main stakeholders within each country have been further divided into four groups for comparative purposes: (i) prescribers including physicians and nurses, (ii) dispensers including pharmacists and drug sellers, (iii) students including both healthcare and non-healthcare students, and (iv) patients and the public.

### Inclusion and exclusion criteria

Within the LMICs, given the high numbers of papers reviewed, we concentrated on countries in Africa, the Middle East and Asia in the four WHO Regions where concerns with antibiotic utilization patterns and ABR levels are at a critical point.^1,7,11^

Only papers dealing with primary care, including hospital outpatients, and published from 2018 onwards until November 2024 were eligible for inclusion in this review. Primary care was selected as the focus for this review because it currently accounts for up to 90% of antibiotic use in humans in LMICs.^37^ The year 2018 was taken as the early cut-off for sourced papers, with NAPs only launched from 2017 onwards following the WHO GAP in 2015; however, we recognized ongoing concerns with their implementation.^22,29,33,113^

We are aware that the extensive social restriction measures introduced to combat COVID-19, which included closure of universities, had a profound impact on the education of HCPs across LMICs.^114–116^ However, we have not separated out papers dealing specifically with COVID-19 and its implications, principally treating COVID-19 as another viral infectious disease as patients with COVID-19 were often inappropriately treated with antibiotics, especially across LMICs.^117–119^

Exclusion criteria included studies discussing KAPs of key HCPs treating inpatients in hospitals as well as LMICs outside the four designated WHO Regions. We also excluded papers published before 2018, those exclusively dealing with animals/veterinary scientists, and any paper not cited in PubMed (for inclusion in the tables).

### Data analysis and narrative synthesis of findings

The data analysis process followed a systematic approach to identify, extract and organize relevant information from the studies, including information about the studies’ characteristics and outcomes of interest. Activities included creating tables containing a summary of the objectives, methodology and key findings by each key stakeholder group (prescribers, dispensers, students and patients/public) as well as by country and region. Furthermore, the documented synopsis for each cited paper included the country, its economic status and the study design as well as the number of participants. Details of any questionnaire design were also included where documented as a key element of the paper and its findings, especially how the questionnaire was developed including any pilot studies as well as any validation. The publication year along with the first author was also included in the tables, with the papers listed in year sequence to assess changes over time where pertinent (Figure 1). Key findings were also documented as part of the narrative synthesis led by the principal author (B.G.). Key findings included any differences between perceived knowledge of antibiotics, ABR, AMR and AMS, among the stakeholder groups versus actual practices, especially in terms of prescribing, dispensing or using antibiotics for essentially self-limiting conditions, driving up ABR.

The emerging themes from each stakeholder group across the LMICs and the four WHO Regions were subsequently synthesized into major themes for comparisons within and across the four WHO Regions. No attempt was made to combine the findings and produce any summary statistics apart from documenting the number of sourced papers by country and WHO Region in view of the recognized heterogeneity of the sourced papers in terms of the populations studied and the methodology. However, key reported quantitative descriptive statistics or measures of effect, such as prevalence rates or mean/median scores related to specific study outcomes or thematic areas, were summarized. This approach was employed to capture the anticipated variability across different studies and contexts, particularly given the diverse methodologies and populations examined across LMICs, as well as key differences between stated knowledge regarding antibiotics and AMR and actual practice. The use of descriptive statistics, including ranges where pertinent, allows for a broader representation of the findings, accounting for regional and contextual differences while providing an aggregated view of key outcomes, including KAP towards antibiotic use, ABR and AMS. Where relevant, the figures are discussed in the context of each theme to highlight variations and commonalities across the different studies and regions.

The consolidated themes were subsequently used to structure a range of future potential interventions to improve KAP in all key stakeholder groups, summarizing key activities to instigate and improve antibiotic use in primary care.

### Ethical considerations

Ethical approval was not needed for this review as this study did not involve direct contact with humans as noted in similar studies.^9,17,82,109^

## Results

The general findings will be discussed first before synthesizing the findings by each stakeholder group and region.

### General

A total of 459 papers were sourced across the LMICs that met the inclusion criteria. These have been divided into the four key stakeholder groups across the four WHO Regions (Tables S1 to S16, available as Supplementary data at *JAC-AMR* Online).

An appreciable number of sourced papers came from the African Region in all four stakeholder categories, representing 42.7% of all published studies, with most coming from Ghana, Nigeria, South Africa and Tanzania (Table 1). The number of papers is perhaps not surprising, with the greatest burden of infectious diseases worldwide, including HIV and AIDS, acute respiratory diseases, malaria and TB, currently in Africa.^120,121^ Alongside this burden, there are high and growing rates of AMR among African countries.^105,120–122^ The appreciable number of publications emanating from China (8.1% of total publications) may reflect high rates of inappropriate antibiotic prescribing and dispensing in the country, encouraged by limited patient knowledge, despite recent government initiatives, along with high rates of AMR.^123–127^ This may be changing; however, there is a continued need to encourage appropriate prescribing and dispensing of antibiotics to reduce AMR in the country.^128–130^

**Table 1.** Breakdown of published studies by WHO Regions and stakeholder groups^a^

WHO Region	Prescribers	Dispensers	Students	Patients/public
**African Region**				
Countries	23	13	12	18
Published studies	90 (40.7%)	40 (18.1%)	27 (12.2%)	64 (29.0%)
Top countries with published studies	South Africa: 23 studies Tanzania: 12 studies	South Africa: 7 studies Ethiopia and Tanzania: 5 studies	Nigeria: 7 studies Uganda: 4 studies	Ghana: 11 studies Ethiopia, Nigeria, Tanzania: 7 studies each
**Eastern Mediterranean Region**				
Countries	6	9	7	12
Published studies	19 (18.8%)	28 (27.7%)	20 (19.8%)	34 (33.7%)
Top countries with published studies	Jordan: 5 studies Pakistan: 4 studies	Pakistan: 13 studies Egypt: 4 studies	Pakistan: 7 studies Jordan: 5 studies	Pakistan: 9 studies Jordan: 7 studies
**South-East Asia Region**				
Countries	8	6	5	6
Published studies	29 (26.6%)	34 (31.2%)	17 (15.6%)	29 (26.6%)
Two top countries	India and Nepal: 8 studies each	Bangladesh: 12 studies Nepal: 11 studies	Bangladesh, India, Nepal and Sri Lanka: 4 studies each	Bangladesh: 11 studies India: 7 studies
**Western Pacific Region**				
Countries	5	4	2	5
Published studies	27 (30.7%)	8 (9.1%)	10 (11.4%)	43 (48.8%)
Two top countries	China: 13 studies Vietnam 5 studies	China: 3 studies Vietnam: 3 studies	China: 6 studies Malaysia: 4 studies	China: 20 studies Malaysia and Vietnam: 8 studies each

^a^Percentage values in brackets of the total number of papers from each Region for each stakeholder group. The total number of documented studies at 518 is more than 459 as some studies included different countries and stakeholder groups.

The considerable number of papers documenting the KAP of the patients/public (33.1% of the total number of publications within each stakeholder group—ascertained by documenting the number of papers cited in Tables S13 to S16 by the total number of cited studies in Tables S1 to S16) reflects their appreciable role in influencing the prescribing and dispensing of antibiotics in LMICs (Table S20).

Differences have been seen between high-income countries and LMICs, especially with respect to antibiotic use patterns, including the self-purchasing of antibiotics, which is appreciably greater in LMICs, as well as educational campaigns among patients.^11,15,48,131–135^ However, concerns about the knowledge of physicians with respect to antibiotics and AMR have been seen across all countries irrespective of income level.^136^ Initially, educational campaigns among patients to improve their knowledge were principally undertaken in high-income countries.^15,137,138^ However, this is now changing.^68,139–143^

The findings from the four key stakeholder groups across the four WHO Regions are summarized in Tables S17 to S20. The consolidated findings have subsequently been expanded to provide additional background to potential interventions that can be implemented by key stakeholders going forward. This includes documenting concerns and issues with the KAP of each stakeholder group across the four Regions, alongside any appreciable differences between stated knowledge regarding antibiotics and ABR and actual practice. The latter is seen as particularly important when it comes to suggesting future activities.

### Summary of key findings among each stakeholder group across the four WHO Regions

#### Prescribers

Overall, reported rates of antibiotic prescribing in primary care were broadly comparable across the LMICs in the four WHO Regions independent of World Bank income levels (Table S17).

High rates of prescribing of antibiotics were noted among physicians (range: 23%–97%) as well as nurse practitioners and clinical officers (range: 35.8%–69%) across the four WHO Regions. However, in some of the LMICs, a higher rate of prescribing of antibiotics was seen among physicians compared with other HCWs.^46,144^ Private health professionals and informal providers also tended to have higher rates of antibiotic prescribing (range: 26.2%–64.3%) across the four WHO Regions. This though was not always the case.^35,42^

Antibiotics were often prescribed for self-limiting conditions, including acute respiratory infections (up to 97%), across the four WHO Regions. This was exacerbated by the lack of time that prescribers typically have with patients in PHCs coupled with patient demands for antibiotics across the LMICs. However, there were exceptions among some countries. This was particularly the case in the Eastern Mediterranean Region where a number of prescribers believed antibiotics were not effective against viral infections, with the body able to fight mild infections without the need for antibiotics.^68,145–147^ However, this was not always the case in this Region, especially where a diagnosis or indication was lacking.^52,148,149^

There were also variable adherence rates to current guidelines among LMICs in the four WHO Regions. This is important with adherence to guidelines increasingly seen as an important marker of the quality of care provided.^9,89,90^ Where recorded, compliance rates varied across the WHO African (45.1%–94.9%), Eastern Mediterranean (32.5%–69.5%), South-East Asia (31.9%–98.5%) and Western Pacific Regions (29.3%–93.37%. As a result, in some LMICs and Regions, there were high rates of prescribing of Watch antibiotics.^144,150–152^ This included the African Region, where up to 55.3% of antibiotics prescribed in one country and sector were from the Watch group;^153^ in the South-East Asia Region up to 54.9% of antibiotics in India in one study were from the Watch group;^154^ and in the Western Pacific Region the rate was up to 31% or more.^155^

There were also concerns with knowledge regarding antibiotics, ABR, AMR and AMS among prescribers across the four WHO Regions irrespective of World Bank income levels. However, there was variability regarding the extent of concerns for these key issues both within and across the Regions. Typically, qualified physicians had greater knowledge of antibiotics, ABR and AMR than other prescribers, including unqualified prescribers. Terminology was also an issue in some countries.

Another key identified concern was that although in some LMICs there was good knowledge of antibiotics, ABR and AMR among prescribers, this typically did not always translate into actual prescribing practices with, as mentioned, typically excessive prescribing of antibiotics across the four WHO Regions. Excessive prescribing of antibiotics includes prescribing them essentially for viral infections such as URTIs as well as those from the Watch list where Access antibiotics or no antibiotics initially are more appropriate. For instance, in the African Region in Sierra Leone in one study, physicians demonstrated sound knowledge and attitudes regarding antibiotics and ABR; however, 68% of them believed that the prescribing of antibiotics may speed up recovery from a cold or cough.^156^ In Nigeria, 49.2% of prescribers had good knowledge of ABR and AMR; however, 75.7% admitted prescribing antibiotics for sore throats.^157^ Alongside this in South Africa, 95.8% of prescribers in one study believed ABR is a major problem; however, 66.5% felt pressure from patients to prescribe antibiotics for their presenting infectious disease irrespective of their potential effectiveness for the presenting infectious disease and the need.^158^

In the Eastern Mediterranean Region in Palestine in one study, despite only 9.2% of participating physicians believing antibiotics can be used to treat viral infections, 60.8% would prescribe antibiotics for acute sinusitis, and 43.4% for a 17-year-old complaining of a sore throat or nonproductive cough.^159^ In Egypt in one study, 98.7% of participating physicians believed bacteria can become resistant to antibiotics; however, 61.4% believed that broad-spectrum antibiotics are preferred for treating URTIs.^146^ In the South-East Asia Region in one study in India, although over 90% of prescribers claimed their antibiotic knowledge was adequate, over 88% of physicians reported prescribing antibiotics for viral infections such as common colds or sore throats.^46^ In Sri Lanka, 99.6% of participating physicians in one study stated that antibiotics should not be prescribed to any patient with fever, but 52.3% almost always/often/sometimes felt under pressure to prescribe antibiotics if patients expect these.^160^ In the Western Pacific Region, for instance in Lao, 91% of physicians stated that extensive use of antibiotics increases the risk of ABR, but 39% would prescribe an antibiotic to an adult with a runny nose, cough or fever to hasten recovery.^161^

The need for enhanced training regarding antibiotics, the AWaRe classification, ABR and AMS, was seen as a definite requirement among all prescribers, including physicians and nurses, across the LMICs in the four WHO Regions to improve future prescribing. This also included training surrounding current national STGs, and with the recent publication of the WHO AWaRe book where no current national STGs exist.^27,28^ This is important with cited studies (Tables S1 to S4) showing that educational and other interventions surrounding STGs increased the appropriateness of antibiotic prescribing.^68,135,162–165^ In addition, prescribers who had access to STGs were less likely to prescribe antibiotics.^166^ Multiple initiatives in China in recent years have also enhanced appropriate prescribing of antibiotics with improved training.^128,129^

#### Dispensers

A similar picture to that for prescribers was seen among dispensers across the four WHO Regions (Table S18), with again the findings irrespective of World Bank income levels. There were typically high rates of dispensing of antibiotics without a prescription across the four WHO Regions where rates were documented. Rates of self-purchasing included up to 100% of patients visiting community pharmacies in the African and Eastern Mediterranean Regions,^167,168^ up to 94% among community pharmacies and drug sellers in the Western Pacific Region,^169^ and up to 86.7% among patients visiting community pharmacies in the South-East Asia Region.^166^

Alongside this, similar to the prescribers across LMICs, the dispensing of antibiotics without a prescription was typically for self-limiting conditions including URTIs, acute respiratory infections (ARIs) and acute diarrhoea as well as for aches, pain and inflammation. Identified concerns going forward also included appreciable dispensing of antibiotics from the Watch group in some of the studied LMICs. High rates of dispensing of azithromycin were also seen among patients with COVID-19 (99.1%) in Kenya.^170^ Watch antibiotics also accounted for 53.6% of antibiotics dispensed among patients visiting community pharmacists in Bangladesh,^171^ and for 49.3% of antibiotics dispensed among patients visiting community pharmacies in Pakistan.^172^ Pharmaceutical company promotional activities also enhanced the dispensing of azithromycin without a prescription in Nepal.^173^ There were also high rates of dispensing of antibiotics from the Reserve group among community pharmacies in Bangladesh (10%) and Pakistan (19.0%).^171,172^

Overall, the reasons for high rates of dispensing of antibiotics without a prescription where this occurs were similar across the LMICs and WHO Regions irrespective of World Bank income status. Key reasons included high patient copayments, convenience of pharmacies, ease of access to antibiotics in community pharmacies and drugstores as well as pressure from patients or carers to dispense.^52,168,174–176^ If anything, rates of dispensing of antibiotics without a prescription were higher in drug outlets versus community pharmacies, and for children versus adults.^176–183^ However, this was not always the case.^184^

Not all countries within the four Regions experienced high rates of dispensing of antibiotics without a prescription. There were stricter regulations and greater access to HCPs in some of the LMICs, for example Botswana and Namibia in Africa and Malaysia in South-East Asia. In these countries, there are strict regulations surrounding the dispensing of antibiotics without a prescription, which is typically enforced, alongside activities of pharmacists to offer alternatives through training.^17,57,185^ There was also limited dispensing of antibiotics without a prescription to patients for essentially viral infections in community pharmacies linked to the University of Nairobi in Kenya.^186,187^

Pharmacists with greater experience, and following educational activities, were generally less likely to sell antibiotics without a prescription.^68,188,189^ However, this was not always the case.^190^ Similar to prescribers, although knowledge of antibiotics, ABR and AMR appeared reasonable to good among dispensers in a number of LMICs, this again did not always translate into actual practice. Dispensers across the four WHO Regions typically recognized their own knowledge and skills gap. They also acknowledged issues of language surrounding the terminology for antibiotics, ABR and AMR as a barrier to reducing inappropriate dispensing of antibiotics for essentially self-limiting conditions.^69–71,191^ Real-time feedback to community pharmacists using mobile and other technologies could also facilitate improved dispensing alongside improved adherence to standard guidance including treatment guidance in the recently launched WHO AWaRe book.^27,28^

#### Students

There were again similar issues regarding the knowledge of healthcare students concerning antibiotics, ABR, AMR and AMS across the LMICs in the four WHO Regions. There were also similar concerns among the countries and Regions irrespective of country income levels (Table S19).

Good knowledge regarding antibiotics among students was seen in some studies in the African Region. In Zambia, 87.3% of participating medical students had good knowledge regarding antibiotic use and AMR, with 99.2% believing antibiotics can cure bacterial infections and 93.5% that antibiotics cannot cure viral infections.^192^ In Malawi, over 88% of surveyed students in one study correctly answered more than half the questions regarding antibiotics and AMR.^193^ In addition, in Nigeria 98.4% of surveyed students in one study believed AMR was an important global public health issue,^194^ with 94.8% in another study believing that antibiotics are overused in their country.^195^ In Rwanda, 98.7% of participating students had heard about AMR, and 78.6% had discussed AMR during their courses, with 95.2% believing that inappropriate use of antimicrobials increases AMR.^196^ In Uganda, 87.5% of surveyed students had sufficient knowledge about AMR.^197^ In another study among universities in East Africa, 72.9% of participating students had a good attitude and perception regarding antibiotic use. The extent of good attitudes was highest in Uganda versus Kenya and Tanzania.^198^ There was also typically greater knowledge regarding antibiotics, ABR and AMR among final-year students versus students with less experience, and among healthcare versus non-healthcare students across studies.^192,193,199,200^

Despite this, there were generally considerable concerns regarding the attitude and practice towards antibiotics and AMR among the surveyed students across Africa.^41,194,201–204^ This was also reflected by high rates of self-purchasing of antibiotics without a prescription, including for self-limiting conditions such as coughs, colds, sore throats and diarrhoea, among students across Africa.^201,203,204^ Rates of 55.3% to 93.8% for self-purchasing without a prescription were seen in studies including among students from Ghana, Nigeria, Tanzania and Uganda.^108,199,201,203,205,206^ These high rates of self-purchasing were driven by easy access and convenience of pharmacies, perceived only minor illnesses, the costs involved, including cost savings with self-medication, and prior knowledge.^207–209^

A similar situation was seen among students in the Eastern Mediterranean Region. In Palestine in one study, 97.4% of students stated they knew about AMR and 86.7% that the overuse of broad-spectrum antibiotics increases AMR.^210^ In Egypt in one study, 95.7% of surveyed students believed indiscriminate use of antibiotics increases ABR and AMR,^211^ and in Jordan, 93% of students in one study stated they knew that unnecessary use of antibiotics makes them ineffective, with 85.5% stating that antibiotics are ineffective against viruses.^212^ In Pakistan, 85.7% of students in one study stated that they knew about AMR, with 79.4% agreeing that a contributing factor for AMR was poorly designed dosing regimens.^213^

However, despite these encouraging signs, there was again appreciable concerns with the overall knowledge of students regarding antibiotics, ABR and AMS across this Region. This was reflected in high rates of self-purchasing of antibiotics among students in a number of the countries. Rates of self-purchasing of antibiotics ranged from 38.1% to 60.8% among surveyed students in the Region.^214–216^ There was also a high percentage of students who believed that antibiotics are effective and/or first line in treating self-limiting viral infections including coughs, colds and influenza.^211,217–219^ In the Middle East, including Egypt, Jordan and Lebanon, 51.7% of pharmacy students reported taking antibiotics to manage a fever.^216^ In Pakistan in one study, 51.4% of surveyed students believed antibiotics can be taken as preventative measures.^220^ Among Arabic-speaking countries, including Jordan, Iraq and Palestine, 44.5% of students believed antibiotics are effective against colds and coughs,^221^ and in another study involving students in Palestine, 66.1% were not confident about AMS.^210^ Alongside this, 43.3% of students in one study in Egypt believed that ABR is only a problem if antibiotics are taken regularly.^217^

Similar to the situation in the African Region, there was generally greater knowledge regarding antibiotics, ABR and AMR among more mature students in the Eastern Mediterranean Region, and with healthcare compared with non-healthcare students.^213,216^

Similar to the other Regions, appreciable differences were seen between knowledge and practice among countries in the South-East Asia Region. In India, 91.8% of students in one study believed antibiotics were useful for treating bacterial infections,^222^ with 84.1% and 82.4% of students, respectively, in another study believing inappropriate use of antibiotics, and not completing the full course, increases ABR.^223^ A study in Bangladesh found that 91.7% of students believed that dispensing antibiotics without a prescription increases ABR, with 82% believing the overuse of antibiotics increases ABR.^224^ In one study in Nepal, 83.3% of students believed that ABR means that antibiotics are taken too often and will not work.^225^ In three Asian countries, including Indonesia, 80.1% of participating students in one study recognized it was inappropriate to treat diarrhoea with antibiotics, with 75.8% strongly disagreeing or disagreeing that AMR is not a serious problem.^226^

However, again there were appreciable concerns regarding the practical knowledge of students concerning antibiotics, ABR, AMR and AMS. In Nepal in one study, 73.7% of participating students thought that antibiotics can cure infections caused by viruses.^227^ In Indonesia, only 26.1% of participating students in one study stated it was inappropriate to treat URTIs with antibiotics.^226^ In Sri Lanka, between 40.2% and 63% of surveyed students in various studies believed antibiotics could cure colds, sore throats and influenza.^228–231^ In Bangladesh, 44.4% of students in one study believed that antibiotics could prevent viral infections.^224^ There was also appreciable self-purchasing of antibiotics among students, including 67.78% of students in one study in India,^232^ up to 61.7% of students in Nepal,^233,234^ and 42.2% to 91.7% in Bangladesh.^224,235,236^ However, this was not always the case among the Regions.^237^ Self-purchasing of antibiotics among students in this Region was again typically for self-limiting conditions including fevers and ARIs.^234–236,238^ Principal reasons for self-medication with antibiotics among students again included convenience, long waiting times to see physicians, previous experiences with antibiotics, the condition is not serious, and costs.^232,236^

Mirroring other Regions, there again appeared greater knowledge regarding antibiotics, ABR and AMR among healthcare and biology students versus other students in the South-East Asia Region as well as among more mature students.^224,227–229^

Lastly, in the Western Pacific Region there were again differences between perceived knowledge and actual practice among students. In one study in China, more than 85.0% of surveyed students were aware that the overuse of antibiotics is a serious problem that could cause AMR as well as lead to difficulties in treating bacterial infections.^239^ In other studies, 74.2%–88.5% of students reported being aware of the dangers posed by the overuse of antibiotics.^240,241^ In one study in Malaysia, more than 88% of surveyed students totally agreed or agreed that penicillin or amoxicillin are antibiotics, and that antibiotics are useful for treating bacterial infections, with 77.5% also stating they do not take antibiotics to treat a cold or sore throat.^242^

In contrast, only 47.0% of surveyed students in one study in China did not agree that antibiotics could reduce the symptoms of common colds,^239^ and in another, 75.2% of students stated they had performed inappropriate antimicrobial practices.^243^ Another study in China reported that 54.3% of students were unaware that antibiotics were ineffective against viral infections, with only 41.6% agreeing that every person treated with antibiotics is at an increased risk of antibiotic-resistant infections.^237^ There was a similar situation in Malaysia, with only 3.3% of surveyed students in one study having a good level of knowledge about antibiotics;^244^ in another study, only 26.1% of students, including those from Malaysia, believed it inappropriate to treat URTIs with antibiotics.^226^ Self-medication with antibiotics was also seen in 48.8%–73.9% of students in China,^240,245^ and in up to 33.7% of students for a cough in Malaysia.^244^

Similar to other studies, students with a medical background in the Western Pacific Region were significantly associated with better antibiotic use behaviours and knowledge versus other students.^244,245^ In addition, more mature students had better knowledge regarding antibiotics, ABR and AMS than junior students.^242,246^

Overall, there was a recognized need among healthcare students across all four WHO Regions, irrespective of World Bank income levels, for greater training on antibiotics, ABR, AMR and AMS during their undergraduate years, with greater training seen as effective with improving knowledge scores.^244,247^ There was recognition that HCPs would need further updated training post-qualification with continuing professional development (CPD). This should include practical tools to help with diagnosis and treatment, as well as tools to provide up-to-date information on local resistance patterns to aid future prescribing and dispensing. To this end, training about robust guidelines such as the recent AWaRe classification and guidance is seen as important.^13,27,28^

#### Patients/public

There were similar concerns and issues to the other key stakeholder groups among patients and the public across the LMICs in the four WHO Regions with respect to their KAP towards antibiotics, ABR and AMR (Table S20).

In the Democratic Republic of Congo in the WHO African Region, all interviewed patients in one study were familiar with the term ‘antibiotics’, with most describing antibiotics as medicines used to treat infections.^248^ Similarly in Ethiopia, 95.9% of participants correctly agreed with the definition of antibiotics, with 59.4% of participants having heard the term AMR, and of these 50.4% were aware of the problems of ABR.^249^ In other studies in Ethiopia, 58.2% of participants were aware of the existence of ABR, 48% had moderate knowledge of antibiotics and AMR, with 35% having good knowledge.^249–252^ In Cameroon, 88.3% of those surveyed in one study stated that antibiotics treat microbes, with only 11.8% believing that antibiotics can be used to treat viral diseases.^253^ In Ghana, 82.4% of respondents in one study were aware that the misuse of antibiotics is a major driver of ABR, and 74.4% knew that ABR is a failure of antibiotics to kill germs.^254^ In another study in Ghana, 75.9% of participants had good knowledge regarding bacteria’s ability to become resistant to antibiotics.^255^ In Tanzania, 96.33% of participants in one study believed that self-medication with antibiotics increases ABR.^256^ A similar situation was seen in Nigeria, Senegal, South Africa and Zambia.^257–262^ In Nigeria, 76.6% of patients in one study stated that bacteria would become less resistant to antibiotics after prolonged use, with only 22.1% believing that antibiotics cure colds and sore throats faster than other remedies.^261^

However, a number of concerns were identified across the countries in the African Region. In Tanzania in one study, only 10.9% of participating parents or guardians had good knowledge about antibiotics,^263^ and in the Democratic Republic of Congo, antibiotics were being used to treat a range of conditions including fever, coughs and profuse sweating.^248^ In Malawi, 92.4% of participants in one study believed antibiotics could stop a fever,^264^ and in Senegal, 72.3% believed antibiotics are effective against a cough,^257^ with 68% of participants in Tanzania in one study admitting to using antibiotics to treat coughs.^265^ Elsewhere in Tanzania, 61.4%–62.5% of participants in different studies believed sore throats could be treated with antibiotics.^266,267^ In South Africa, 66% of participants in one study incorrectly stated that antibiotics are effective for treating viruses,^262^ with generally a common misconception that antibiotics can treat colds, influenza and fever.^268^ Alongside this in Uganda in one study, 60.2% of children with ARIs were treated with antimicrobials,^269^ and in Zambia in different studies, 58.2% of participants had taken an antibiotic for a common cold,^259^ and 59.1% had used antibiotics inappropriately.^270^ Similar findings were seen in Eritrea, Ethiopia, Ghana, Nigeria, Mozambique and different studies in Zambia.^250,271–277^

Among the countries in the WHO African Region, patients also admitted to appreciable self-purchasing of antibiotics without a prescription. Self-purchasing was typically for self-limiting conditions including coughs, colds and fever, which was driven by ease of access to community pharmacies and drugstores and their availability in these locations, previous experience, condition seen as minor, costs, location (e.g. rural area) as well as a desire for quick relief of their infectious diseases.^262,272,274,278–284^ Documented rates of self-purchasing of antibiotics among participating patients in the studies varied across the countries. These were 23.6% in Kenya,^285^ 38.9% in Ethiopia,^281^ 45.1% among participants in Eritrea including for URTIs,^280^ 55.4% in Uganda,^286^ 58%–76.3% in Tanzania,^256,284^ 36%–76% among participants in Ghana,^100,287,288^ 74.0% in Zambia,^259^ 47%–81.4% in Cameroon,^253,289^ 26%–86.5% in Nigeria,^208,290^ and 93.75% in Mozambique.^291^

In the WHO Eastern Mediterranean Region, there were again appreciable differences between stated knowledge and practice. In Jordan, 88.3% of participants agreed that antibiotics are being excessively used to treat URTIs,^292^ with up to 92.6% of participants in one study believing antibiotics are ineffective against common colds,^293^ and only 27% of participants in another study believing antibiotics can be used to treat both bacteria and viruses.^294^ There were similar findings in studies in Morocco, Pakistan and Yemen.^64,295,296^ For instance in Pakistan, 61.7% of participants in one study had good knowledge regarding antibiotics and AMR and the fact that antibiotics kill bacteria.^64^

Among participants in a study in Afghanistan there was poor knowledge regarding antibiotics, with surveyed patients typically seeing antibiotics as the ‘desired medication’, with high expectations that antibiotics will be prescribed by physicians for their infections whatever the cause.^297^ In Yemen, 88.8% of participants in one study stated they had used antibiotics to treat a fever,^295^ and in Morocco, 78% of those surveyed in one study believed that respiratory tract infections (RTIs) should be treated with antibiotics.^296^ In Jordan, 72.4% of participants strongly agreed/agreed that once a child develops a fever, they should be given antibiotics regardless of the cause.^292^ In Pakistan in one study, the majority of participants believed antibiotics can treat viral infections and that all types of infection can be cured with the help of antibiotics,^64^ with overall knowledge regarding ABR seen as poor.^298^ Among Arabic-speaking countries including Egypt, Iraq, Jordan and Palestine, 58.8% of surveyed patients thought antibiotics are effective against sore throats, and 57.6% believed they combat common colds, coughs and nasal congestion.^221^ Similar findings were also seen in other studies in Jordan,^292,299^ Iraq^300^ and Pakistan,^52,301,302^ as well as in Lebanon and Tunisia.^303–305^

Self-medication with antibiotics among patients was also common in the WHO Eastern Mediterranean Region. Documented rates among patients ranged from 20.6% of participants in Tunisia^303^ to 32.1% in Egypt,^306^ 33.4%–59% in Pakistan,^301,307^ 44% in Jordan,^308^ 45.8% in Iraq,^300^ up to 56.1% in Sudan,^309^ 61.6% in Iran^310^ and 68% in Morocco.^296^

Similarly in the WHO South-East Asia Region, there were again considerable differences between stated knowledge and actual practices. In Nepal, 94.1% of participants in one study answered correctly that antibiotics are useful for killing germs, and 84.1% that antibiotics are often not needed for treating colds and influenza.^311^ In India, 78.7%–89.7% of participants in various studies agreed that it is the obligation of everyone to use antimicrobials responsibly to reduce AMR.^312,313^ In Indonesia, 67.7% of participants in one study correctly stated that inappropriate use of antibiotics will cause ABR,^314^ and only 12.5% believed antibiotics are effective against viruses.^315^ Similar findings regarding the appropriate and inappropriate use of antibiotics and ABR were seen in studies in Bangladesh and Myanmar.^316,317^

In contrast in Nepal, 84.6% of participants in one study reported sometimes preferring an antibiotic when they have a cough or sore throat,^311^ and in another study participants typically demonstrated a low level of knowledge concerning AMR.^318^ In Bangladesh in one study, 90.7% of participants believed antibiotics are effective in treating viral infections, and 84.76% thought that antibiotics speed up recovery from most coughs/colds.^316^ In addition, community pharmacists in Bangladesh believed that if they advise patients against taking antibiotics for minor illnesses including colds/coughs and fever, or insisting on dispensing antibiotics only with a prescription, patients may take their business elsewhere.^183^ In India, there was minimal understanding of the term antibiotic in one study,^319^ and in Sri Lanka, overall knowledge among participants about antibiotics was poor.^320^ Similar findings were seen in other studies in Bangladesh and Nepal,^321,322^ as well as in India, Indonesia and Myanmar.^313,314,317,323^

Again, self-purchasing of antibiotics was reported by patients across the WHO South-East Asia Region. Reported rates ranged from 11% in Sri Lanka,^320^ up to 50.9%–80.9% in Bangladesh, which included high rates of dispensing of antibiotics from the Watch list.^171,324^ The top three infectious diseases for self-medication with antibiotics in Bangladesh were coughs, colds and a fever.^325^ In one study in India, participants stated that they typically go to informal providers as a result of economic conditions, with coughs and colds typically treated with antibiotics for 3 to 5 days.^326^

Similarly, in the WHO Western Pacific Region there were again appreciable differences between stated knowledge regarding antibiotics, ABR and AMR and actual practices. In China, 83.5% of participants in one study believed that antibiotics are not necessary for common colds, and 70.2% averred that overuse of antibiotics increases ABR, with similar findings seen in an additional study (62.8% of participants).^327,328^ In Cambodia, 74.8% of participants in one study believed that AMR will make infections more difficult to treat,^329^ and in Malaysia in one study, the majority of respondents had good knowledge of antibiotic usage and ABR problems.^330^ Similar findings regarding good attitudes towards antibiotics were also seen in other studies in China, Malaysia and Vietnam.^331–333^

However, common uses of antibiotics among patients in Lao and Thailand included for coughs (30.5%), fevers (30.5%) and sore throats (28.9%).^70^ In Vietnam, only 18.8% of participants in one study knew that ABR has a negative effect on antibiotics in terms of reducing their effectiveness.^334^ Frequent and indiscriminate use of antibiotics is common among patients in Vietnam, driven by the powerful appeal that antibiotics have, with 67.9% of participants in one study believing that antibiotics can be used to treat viruses.^335,336^ Similarly in China, 91.6% of participants in one study believed that antibiotics can control viruses,^337^ with 81.3% of participants in another study admitting to using antibiotics to treat their RTIs.^338^ In Malaysia, studies among patients also documented a high percentage believing antibiotics can hasten recovery from viral infections, or cure these, ranging from 41.5% to 83.7% of surveyed participants.^339–342^ In other studies in Malaysia, only 20.6% to 23.8% of participants knew that antibiotics are not effective against viral infections.^330,342^ Similar findings were also seen in other studies in Cambodia, China, Lao and Vietnam.^327–329,331,343–348^

Self-purchasing of antibiotics was also common in the WHO Western Pacific Region. Drug suppliers in Vietnam stated that patients often request specific antibiotics to treat their infections based on previous experiences.^169^ Overall reported rates of self-purchasing among patients ranged from 10.3% to 92.1% in China, depending on the population and location,^123,127,327,332,337,348–353^ 15.1% in Malaysia,^341^ and up to 81.7% in Vietnam.^354^

Overall, patients’ and parents’ beliefs regarding the effectiveness of antibiotics across a range of self-limiting infectious diseases are a major challenge across the four WHO Regions as they have resulted in considerable pressures on both prescribers and dispensers to prescribe/issue antibiotics in situations where they are not needed and could cause harm (Table S20). Generally, though, there was a better attitude towards antibiotics among more educated patients.

Encouragingly, education initiatives across the Regions did appear to appreciably improve mean knowledge scores and the practical use of antibiotics in terms of reducing their use for essentially viral infections.^68,139–143^ In addition, in the study of Otieku *et al.*,^355^ participants exposed to an educational intervention to increase their knowledge base were significantly more likely to recommend restrictive access to antibiotics. However, there can be challenges with undertaking educational campaigns among patients.^356^ Issues and concerns with patients’ understanding of the terminology regarding antibiotics and ABR were recognized as needing to be addressed, especially by dispensers, when patients or their care givers are educated regarding the appropriate management of their infectious diseases.^69–71,357^ Understanding these issues is crucial when health authorities and others are designing educational initiatives for patients.^358,359^ However, it was noted that any intervention or targeted programme must include a range of stakeholders to ensure these are appropriate for the health system, community and cultural context in question to enhance the chances of success.^360^

## Discussion

We believe this to be the most comprehensive narrative synthesis to date of KAPs across multiple stakeholder groups in LMIC primary care settings. The wide range of priorities identified in the reviewed papers have been broken down into different key stakeholder groups: governments/health authorities, prescribers (physicians/nurses), dispensers (principally community pharmacists), universities/students and patients/public. We are aware that dispensers also include the informal sector in many LMICs, e.g. Bangladesh, Malawi and Zimbabwe. However, in this section we will deal just with activities with community pharmacies and discuss implications and activities for the informal sector in future publications. The need for enhanced activities surrounding the AWaRe system and prescribing guidance in the WHO AWaRe book was noted for all key stakeholder groups to meet UNGA targets.^98^

Following the principles of the narrative review, the main training needs identified have been adapted into a range of potential AWaRe-based educational activities and are summarized in Box 1.

Box 1.
**Potential cross-stakeholder AWaRe educational initiatives through the WHO and other international organizations and governments.**

*A) Initial phase*—building AWaRe-ness focused on optimal antibiotic use and the WHO AWaRe book

- Practical updated guidance on the principles of optimal antibiotic use, including recent major research findings that impact on routine clinical care.
- Simple short guidance based on the WHO AWaRe book. These could include R-U-AWaRe infographics—simple and easy summaries of the AWaRe book guidance for the main infections seen in ambulatory care translated into local languages where necessary.
- The Quick-AWaRe book—a short version of the most common infections seen in primary care broken down into summary key points for each clinical area locally adapted to reflect normative standards of care.
- The Your-AWaRe book localization tool—integration of the WHO AWaRe book with local government/national guidelines with an online tool. This would include a localization process to ensure that the guidance has been appropriately adapted to specific settings.
- AWaRe-ness training—building a series of webinars/talks/training based on the WHO AWaRe book and system relevant to the stakeholder group.
- Local and nationally adapted AWaRe quality indicators adapted per WHO Region/Country building on ongoing activities.^94^

*B) Second phase*

- Development and dissemination of a global community of AWaRe champions and trainers including a toolkit to improve AWaRe-ness through local and national projects/audits/implementation projects.

As noted above, the main findings with respect to the KAPs of the different stakeholder groups were remarkably similar across the four WHO Regions (Tables S17–S20). As a result, the suggested activities to improve future antibiotic prescribing and dispensing to meet future national targets for the optimal use of Access antibiotics will also be similar across the four WHO Regions. In view of this, just the suggested activities for government/health authorities will be documented here (Table 2). Suggested activities for the other key stakeholders, including prescribers, dispensers, universities/students and patients, are included in Tables S21–S24.

**Table 2.** Potential interventions for government/health authority activities

Short to medium term (1 to 5 y)	**A) National Action Plans** Governments and health authorities across LMICs need to focus on reducing inappropriate use of antibiotics in the primary healthcare setting alongside other measures to reduce AMR in a One Health approach.^361^ This will necessarily involve resources (technical/personnel and financial) to address current challenges including concerns with the KAP of all key stakeholder groups translating into high levels of inappropriate prescribing and dispensing of antibiotics. This includes a focus on the integration of routine antibiotic surveillance into electronic healthcare systems such as DHIS2 and others as part of the primary care agenda.^362^ Routine surveillance would include the monitoring of prescribing and dispensing patterns against agreed AWaRe-based prescribing/quality indicators and quantity metrics. As part of this, governments and health authority personnel in each country may need to refine existing indicators to ensure their relevance and enhance their use.^94^ Continue to encourage the routine use and standardization of primary care ASPs among prescribers and dispensers to improve future antibiotic use. As part of this, continue to assess the cost-effectiveness of policy and ASP interventions with the aid of academic institutions to help refine future strategies given often limited resources in LMICs.^140,363^ This includes the future role of artificial intelligence/machine learning to improve future antibiotic use as part of ASPs,^364–366^ and possible virtual reality initiatives.^367^ Seek to pilot/introduce educational campaigns among patients to help attain NAP goals, cognizant of language and potential gender issues, and monitor their outcomes with the help of academic units as part of NAP activities.^70,166,357,368^ **B) Prescribers (physicians and nurses)** Continue to evaluate the KAP of prescribers given current concerns regarding antibiotics, AMR and ASPs among LMICs in practice, and instigate additional educational activities where necessary through the help of universities as part of CPD activities. Encourage prescriber organizations in each country to work with governments/health authorities to refine local guidelines based on the WHO AWaRe book and local resistance patterns. As part of this, key organizations need to work closely with physicians and other prescribers in their country to encourage them to regularly consult national/local guidelines concerning optimal treatment for patients, with guideline adherence increasingly seen as a key quality marker. Where necessary, key organizations should work with governments/health authorities to make guidelines easily accessible to prescribers—increasingly through simple, easy-to-use applications and other systems (Box 1). Such activities should be part of local ASP activities to help achieve UNGA goals. This will require key organizations to work with national governments and health authorities to improve electronic systems within countries to enhance real-time monitoring of prescribing practices. Alongside this, key organizations should work with governments and health authorities to refine and prioritize the introduction of pertinent quality indicators based on the AWaRe system. **C) Dispensers (community pharmacists** *)* Continue researching the KAP of dispensers given current concerns regarding antibiotics, AMR and ASPs among LMICs in practice across the four WHO Regions. Seek to instigate additional educational activities where necessary through the help of universities as part of CPD activities. This includes activities to help dispel misinformation, which was problematic during the COVID-19 pandemic.^369–372^ As part of this, governments/health authorities in each country need to work with key pharmacy organizations to help encourage dispensers to regularly consult national/local guidelines concerning optimal treatment for patients presenting with infectious diseases where there are concerns. Where necessary, governments/health authorities need to work with pharmacy organizations to make guidelines easily accessible to dispensers—increasingly through simple, easy-to-use applications and other systems (Box 1). Governments/health authorities also need to ensure that any list of antibiotics that can be dispensed in community pharmacies is in line with WHO AWaRe book recommendations where there are current concerns.^373^ This includes any antibiotics that are part of potential quality indicators introduced in community pharmacies to improve future antibiotic dispensing and their monitoring. Alongside this, governments/health authorities need to work with pharmacy organizations to improve monitoring systems in community pharmacies, which can include the use of mobile technologies.^17,374^ **D) Universities** Governments/health authorities need to work with universities to ensure that current HCP curricula meet agreed standards. This increasingly includes the fact that no HCP student should leave university without being fully aware of the WHO AWaRe classification and guidance. Governments/health authorities also need to work closely with universities to ensure that CPD activities are aimed at improving dispenser knowledge of national/AWaRe book guidelines in order to offer appropriate advice to patients when presenting with self-limiting conditions such as URTIs. Alongside this, instigation of communication skills with patients.^375^ Governments/health authorities also need to work closely with universities to monitor the KAP of all key stakeholders towards antibiotics, AMR and AMS, as well as research the outcomes of any national initiatives, including guideline introduction and possible quality indicators, as part of academic activities. This also includes the future role of artificial intelligence/machine learning activities to improve antibiotic use as part of ASPs.^364–366^ Alongside this, use academic institutions to help assess the cost-effectiveness of any targeted educational campaign among patients. **E) Patients/patient organizations** Work with patient organizations across LMICs to improve existing communication regarding the lack of effectiveness of antibiotics for viral infections/high levels of inappropriate use will increase AMR—building on successful campaigns among LMICs.^68,139–143^ Patient organizations can also help governments/health authorities to understand key issues surrounding the terminology of antibiotics and AMR. These issues are crucial when health authorities and others are designing educational initiatives for patients to enhance their chance of success.^357–359^ In addition, work with patient organizations to explore new social media channels to effectively communicate with patients as well as address misinformation, which was problematic during the COVID-19 pandemic.^172,363,376–378^ Alongside this, also explore virtual reality initiatives to increase awareness of antibiotics and AMR among the public.^367^ Work with patient organizations to encourage people to become antibiotic guardians where such programmes exist.^379^
Longer term	Regularly monitor antimicrobial use patterns across all sectors as part of agreed NAPs. Instigate where pertinent additional multiple strategies to improve antibiotic utilization based on the WHO AWaRe book guidance. This includes continuing to improve AMR surveillance as well as enhancing electronic monitoring systems where pertinent. Continue to refine international guidance and quality indicators where pertinent to continue to enhance their acceptance. As a result, ensure any indicators remain current as well as avoiding overloading HCPs across locations. In addition, continue to research the cost-effectiveness of ASPs as well as educational activities among patients to help achieve UNGA goals, and refine where necessary. Continue to work with universities to ensure adequate training of HCPs as well as support for CPD and research activities.

Similar to high-income settings, even when a higher level of theoretical knowledge was identified, attitudes and practice were markedly different to self-reported knowledge about antibiotics and AMR. This was a very notable finding across all the four WHO Regions and countries of varying income groups. There was also a clear lack of any postgraduate education or training on optimal antibiotic use across all relevant stakeholders, with most studies noting the respondents surveyed were typically dissatisfied with their own knowledge and the resultant implications for routine practice.

Overall, across the major stakeholder groups, there was a clear need to improve the level of basic knowledge on optimal antibiotic use. This was particularly notable for the primary healthcare sector, where no detailed WHO guidance on undertaking ASPs in the primary care setting currently exists. However, this will require financial resources and personnel to help instigate, or update, information technology (IT) and surveillance systems, to routinely collect pertinent healthcare data to improve future antibiotic use in primary care. Such activities can build on systems such as DHIS2, which has been developed for Africa, Asia and the Middle East,^380^ as well as GLASS.^362^ Activities to enhance IT systems, including electronic patient records, are essential to undertake ASPs on a routine and real-time basis. This is extremely difficult with paper-based systems, with analysis and reporting typically taking several months after the event. As part of this, governments and health authorities also need continually to assess the cost-effectiveness of any ASPs that are being instigated, including the potential role of artificial intelligence, to improve future antibiotic use in a cost-efficient manner. This is particularly important in LMICs given their limited resources.

There also needs to be continuing research regarding effective and efficient ways to educate patients on antibiotics and AMR given their increasing role in LMICs, while remaining mindful of limited resources. Governments and health authorities also need to work closely with key stakeholder groups to refine the WHO AWaRE book guidance for the local situation, building on activities suggested in Box 1. These include developing and agreeing potential indicators to help progress towards UNGA Access targets.

Governments and health authorities also need to work with key pharmacy organizations to reduce inappropriate dispensing of antibiotics, especially for self-limiting conditions. This again includes instigating systems to better monitor antibiotic dispensing patterns alongside any agreed indicators. Universities also need to ensure that on graduation all healthcare students are fully conversant with the WHO AWaRe classification and guidance, as well as ASPs, given ongoing concerns across all four WHO Regions (Table S19). Consequently, universities need critically to re-evaluate their curricula for the different student HCPs to see if they are fit for purpose for the future. Similarly for any CPD activities.

Finally, governments and health authorities need to engage more with patients and their organizations to address current misconceptions regarding the effectiveness of antibiotics for viral infections as well as the need to complete the full course of any antibiotics prescribed or dispensed. This would reduce the pressure on prescribers and dispensers to provide antibiotics for essentially self-limiting conditions. This includes closer working with patient organizations and others to explore new social media channels to more effectively communicate with patients in languages and with concepts that they understand. Such activities can build on the social media experiences and learnings from the COVID-19 pandemic, including vaccinations, where there were appreciable concerns.^369,381–383^ These various activities are explored further in Table 2 as well as in Tables S21–S24, for each key stakeholder group.

We are aware of many limitations to this study. It was not practically possible, given the number of studies identified, to report data across all WHO regions and countries. However, all the major stakeholders involved in the day-to-day prescribing and dispensing of antibiotics were included, as well as students across all subject areas alongside patients and the public. Given the wide variety of methodologies used in the included papers, a formal systematic review was not possible. However, the marked similarity of the main findings across all stakeholders and countries included made the wider generalizability of the key findings more likely.

This review was also conducted during the preparations for UNGA-AMR in 2024. The major commitment of UNGA that 70% of all antibiotics used in humans should be Access antibiotics has added urgency to the key findings of the results. There is a clear need for major development in the training across all stakeholders of optimal antibiotic use. This review provides a strong rationale that although there is a need for adaptation and localization, the core components of the practical training required could be addressed by a standard programme with national and regional modules. This is practical and affordable with a small number of globally relevant current educational training programmes that could be developed and enhanced, with a particular focus on primary care.

## Conclusions

The development of the AWaRe system by the WHO, including the practical prescribing guidance of the WHO AWaRe book, has created a clear opportunity for the standardization of education regarding optimal antibiotic use, and focusing on practical training in primary healthcare in LMIC settings. This narrative review has noted that the unmet training needs to enhance KAPs were very similar across all key stakeholder groups, regions and countries.

Consequently, the next step is to identify all current educational provision focusing on improving antibiotic use, developing a network of current providers including universities and educational partners, and building collaboration with the WHO regarding a new, and sustainably funded, AWaRe antibiotic educational training initiative. Alongside this, other key aspects associated with increasing ABR in LMICs, including the role and influence of the informal sector, should be investigated further as part of a co-ordinated holistic approach. Such activities are essential to meet UNGA targets for the use of Access antibiotics and AMR.

## Supplementary Material

dlaf033_Supplementary_Data

## Data Availability

Additional data is available from the Corresponding Author upon reasonable request. However, all available information has been referenced.

## Supplementary data

Tables S1 to S24 are available as Supplementary data at *JAC-AMR* Online.
