# Supplementary material for: Status and implications of the knowledge, attitudes and practices towards AWaRe antibiotic use, resistance and stewardship among low- and middle-income countries
Source: JAC Antimicrob Resist. 2025 Mar 25;7(2):dlaf033. doi: 10.1093/jacamr/dlaf033 (PMC11934068; doi:10.1093/jacamr/dlaf033)
Supplement: dlaf033_Supplementary_Data [file dlaf033_supplementary_data.pdf]

Supplementary Material

The findings from the 459 sourced papers among low- and middle-income countries (LMICs) where there are concerns about the KAP of antibiotics, ABR, AMR, and ASPs, have been divided into four key stakeholder groups. These include (i) Prescribers including physicians and nurses; (ii) Dispensers including pharmacists and their assistants as well as drug store owners; (iii) Students and (iv) Patients/ Public. Subsequently, the LMICs have been divided into four pertinent WHO Regions: (i) the African Region; (ii) Eastern Mediterranean Region; (iii) South East Asian Region and (iv) Western Pacific Region, with the countries in each Region further broken down by income level. This has resulted in 16 Tables (Tables S1 to S16). Within each Region, the countries are broken down by income level. Within each income level, the countries are listed in alphabetic order and year sequence starting with the earliest year.

The findings from each stakeholder group across each of the four WHO regions have been consolidated into four Tables, each with key themes (Tables S17 – S20). The objective is to be able to compare and contrast key stakeholder groups by WHO Region to give future guidance. The future guidance for prescribers (Physicians, Nurses and their Associations – Table S21), Dispensers (principally Community Pharmacists and their Associations – Table S22), Universities (Table S23) and Patients and their Associations (Table S24) build on Tables S17 – S20 as well as the guidance to Governments and Health Authorities (Table 2 – Discussion).

Table S1: Knowledge, Attitude and Practices, antibiotics, antimicrobial resistance and antimicrobial stewardship – WHO African Region Prescribers

Country	Author and Year	Objectives, Study Design and Population	Summary of the Findings
**African Region**	Bulabula et al., 2018 (1)	• Assess the current involvement of nurses in the use and management of antimicrobials and their training in AMS across Africa<br>• Cross-sectional study using a structured and piloted questionnaire<br>• 173 respondents	• 96% of respondents were aware of the term 'AMS' and 88.5% undertook AMS tasks as part of their job; however, 44.9% reported barriers in doing so<br>• AMS training was delivered to 36.7% and 53.6% of nurses respectively during their undergraduate and postgraduate education<br>• Three-quarters of the institutions of the surveyed nurses had AMS initiatives; however, only ~41% reported having seen any national AMS guidelines<br>• Overall, 95.4% of respondents asked for further education on AMS and the majority preferred AMS training to be part of the infection prevention curriculum (IPC) education
	Allwell-Brown et al., 2020 (2)	• Compiled data from national surveys in LMICs documenting antibiotic use for sick children under 5 years between 2005-17 with fever, diarrhea, or cough with fast or difficult breathing<br>• Data based on 132 Demographic and Health Surveys/ Multiple Indicator Cluster Surveys among 73 LMICs by WHO region, World Bank income classification, and symptoms<br>• African LMICs accounted for 46.1% of surveyed children	• Antibiotic use among sick children under 5 years with these conditions increased from 36·8% in 2005 among surveyed LMICs, including African countries, to 43·1% in 2017, with low-income countries having the greatest relative increase rising 34% from 29·6% in 2005 to 39·5% in 2017. However, remained the lowest of any income group throughout the study period<br>• Overall, LMICs in the Africa Region had the lowest reported antibiotic use in 2017 at 35·4%; however, had the greatest relative increase in antibiotic use rising from 29·8% in 2005 to 35·4% in 2017 (overall 19%)<br>• Reported antibiotic use among sick children with diarrhoea among the surveyed LMICs remained relatively unchanged during the study period; however, among those with fever alone antibiotic use rose 13% from 38·8% in 2005 to 43·7% in 2017. Similarly, those with a cough with fast or difficult

			breathing symptoms alone – antibiotic use increased 20% from 41·4% in 2005 to 49·6% in 2017
	Knowles et al., 2020 (3)	• Assess antibiotic availability and use among PHC in 20 LMICs including Benin, DR Congo, Kenya, Malawi, Namibia, Rwanda, Senegal, Sierra Leone, Somalia, Tanzania, Uganda, Zambia and Zimbabwe<br>• Obtained data on availability among 13,561 health facilities in 13 service provision assessment/ 8 service availability and readiness assessment surveys involving 27 antibiotics (19 Access, 7 Watch, 1 unclassified)	• Co-trimoxazole and metronidazole were the most widely available antibiotics in the surveys - stocked in 89.5% of health facilities<br>• This contrasts with 17 other Access and Watch antibiotics stocked by fewer than a median of 50% of facilities<br>• Of the 22,699 children observed, 60.1% were prescribed antibiotics - mostly co-trimoxazole or amoxicillin<br>• Children with respiratory conditions were most often prescribed antibiotics (76.1%) followed by undifferentiated fever (50.1%) and diarrhoea (45.7%) and malaria
	Tesema et al., 2024 (4) and Bezie et al., 2024 (5)	• Assessing antibiotic prescription sources and use among children <5 y of age with fever and cough in sub-Saharan Africa using Demographic and Health Survey data from 37 countries<br>• Prescriptions formal healthcare settings included those from clinics with informal settings including traditional practitioners, drug sellers and supermarkets<br>• 18,866 children <5 years who had a fever/cough were included	• 67.19% of children <5 y of age who took antibiotics for a cough/ fever obtained these from unqualified sources - ranging from 40.34% in Chad to 92.67% in Sao Tome<br>• The odds of the use of antibiotics prescribed from unqualified sources were 1.23 times higher in rural areas vs urban areas, with similar findings with antibiotics prescribed from qualified sources<br>• The use of antibiotics from qualified sources for fever/cough among children <5 years were lower for mothers with primary, secondary and higher education. Overall, rates decreased by 14%, 21% and 21% respectively compared with those whose mothers had no formal education
**Low Income countries***			
**Burkina Faso**	Sie et al., 2019 (6)	• To understand antibiotic prescribing practices among 6 PHC facilities in a rural setting in children <5 years of age<br>• Retrospective analysis of patient records and interviews among 301 caregivers using a structured questionnaire	• There were 1,444 prescriptions among 3,401 analysed children over the 3-month study period<br>• There were 1.70 prescriptions/ child-year with penicillins the most commonly prescribed antibiotic (1.04 prescriptions/ child-year) followed by sulfonamides (0.69 prescriptions/ child-year) and macrolides (0.38 prescriptions/ child-year)<br>• 27.3% of caregivers reported their child had visited a health facility in the previous month, mostly for malaria (22.0%), fever (19.0%) or a cough (7.3%) with antibiotics prescribed in 84.1% of visits
	Sie et al., 2021 (7)	• Evaluate antibiotic prescribing patterns among children <5 years with common childhood illnesses and identify the diagnoses with the most potential to reduce unnecessary antibiotic use<br>• Data extracted from patient records (61355 visits during the study period	• 50.5% of children received an antibiotic – more common in the rainy season, 76% prescribed for pneumonia, malaria, diarrhoea, dysentery, fever, or cough<br>• Highest rate of prescribing was for pneumonia (97.1%) and dysentery (91.9%)<br>• Amoxicillin was the most commonly prescribed antibiotic for pneumonia; metronidazole and ciprofloxacin for dysentery<br>• Approximately 20.0% of children received an antibiotic for non-bloody diarrhoea - mostly for ciprofloxacin
	Valia et al., 2024 (8)	• Quantify antibiotic use by type of provider as well as compliance with the WHO AWaRe Book<br>• 2196 patients were interviewed - 2108 had acute illness with 67.9% attending health	• Antibiotics were more frequently prescribed at health centres (54.8%) versus formal pharmacies (26.2%, *P* < 0.001) and informal medicine vendors (26.9%, *P* < 0.001)<br>• Among all antibiotics prescribed for outpatients, 85.2% were Access and 14.8% Watch antibiotics

		centres for their treatment principally as out-patients (87.3%), with 15.6% visiting pharmacies and 16.6% informal medicine vendors	• Clinical presentations including rhinopharyngitis, bronchitis, undifferentiated fever, and gastroenteritis for which antibiotics are not recommended accounted for 68.4% of all Watch antibiotics, with ciprofloxacin the most frequently used Watch antibiotic<br>• Overall, high prescribing of antibiotics in health centres for bronchitis (92.0%) and undifferentiated fever (72.1%)
	Wieters et al., 2024 (9)	• Assess antibiotic use by WHO AWaRe classification among patients visiting healthcare facilities in 4 African countries including Burkina Faso<br>• Infectious diseases surveyed included acute febrile disease of unknown cause (AFDUC), GI infections and RTIs<br>• Out of 19,700 enrolled patients, 36.8% reported antibiotic use in the previous ten days for these infections	• Out of the 36.8% across the countries stating antibiotic use in the previous 10 days, 41.5% were prescribed for RTIs, 30.3% for AFDUC and 22.6% for GI infections<br>• A higher number of enrolled patients in Burkina Faso had RTIs and AFDUC treated with antibiotics - 47.0% and 35.6% respectively<br>• The most common antibiotic prescribed was ceftriaxone (31.7% of antibiotics prescribed – higher in Burkina Faso at 48.2% of all antibiotics taken). This was the case among patients with AFDUC - ranging from 29.0% of patients in Côte d'Ivoire to 62.6% in Burkina Faso.<br>• Among those with RTIs, amoxicillin/clavulanic acid was the most commonly reported antibiotic prescribed in Côte d'Ivoire (32.3%) and Burkina Faso (41.4%)
**Democratic Republic of Congo (DRC)**	Ingelbeen et al., 2022 (10)	• To quantify healthcare provider-specific and communitywide antibiotic use among different sectors including healthcare clinics, medicine stores and self-treatment<br>• Household survey based on previous surveys in two health zones	• Private clinics (31.0%) and PHCs (25.5%) were the most frequently visited healthcare facilities<br>• Antibiotics were prescribed/ dispensed during 64.3% of visits to private clinics and 51.1% to PHCs vs. 48.8% to medicine stores.<br>• The most frequent antibiotic classes used were the penicillins (49.5%) and cephalosporins (14.2%), with cephalosporins more frequently used in private clinics (25.3%) vs. medicine stores (11.8%) or health centres (9.3%)<br>• % of Watch antibiotics prescribed/ dispensed was highest in private clinics (30.3%) vs. medicine outlets (25.6%) or PHCs (25.1%)<br>• Treatment durations <3 days were more frequent among private clinics (5.3%) and medicine stores (4.1%) vs. PHCs (1.8%)
	Kakumba et al., 2023 (11)	• Explore antibiotic dispensing patterns among HCPs<br>• 400 medical prescriptions were collected from 80 drugstores<br>• Prescribed antibiotics were broken down by ATC class and AWaRe classification	• 75.25% of prescriptions contained antibiotics<br>• Of these, 54.5% of prescriptions contained one antibiotic, 38.9% two antibiotics, 5% three antibiotics and 1.6% four antibiotics. As a result, a total of 463 antibiotics were prescribed<br>• Third generation cephalosporins were the most prescribed antibiotics (34.33%), followed by penicillins (17.17%) and macrolides (7.63%)<br>• Overall, 43.2% of antibiotics prescribed were from the Watch group, 36.5% were from the Access group and 20.3% were from the not-recommended antibiotics group
	Wieters et al., 2024 (9)	• Assess antibiotic use by WHO AWaRe classification among patients visiting healthcare facilities in 4 African countries including DRC Congo	• Out of the 36.8% across the countries stating antibiotic use in the previous 10 days, 41.5% were prescribed for RTIs, 30.3% for AFDUC and 22.6% for GI infections<br>• A lower number of enrolled patients in DRC had RTIs treated with antibiotics 34.9%

		• Infectious diseases surveyed included acute febrile disease of unknown cause (AFDUC), GI infections and RTIs<br>• Out of 19,700 enrolled patients, 36.8% reported antibiotic use in previous the ten days for these infections	• The most common antibiotic taken was ceftriaxone (31.7% – lower in DRC at 28.9%). This was the case among patients with AFDUC syndrome - ranging from 29.0% of patients in Côte d'Ivoire to 62.6% in Burkina Faso (29.9% in DRC)<br>• Among those with RTIs in DRC, 30.6% received ceftriaxone vs. 21.9% amoxycillin and only 5.7% amoxicillin/clavulanic acid
**Eritrea**	Abdu et al., 2024 (12)	• Assess antibiotic prescribing practices and their determinants among outpatient prescriptions to the elderly<br>• 2680 prescriptions were analysed among specialists, GPs and nurse prescribers	• Prevalence of antibiotic prescriptions among outpatients was 35.8% - greatest among GPs and least likely among nurse practitioners<br>• 53.7% of prescribed antibiotics were from the Access group and 32.1% from the Watch group<br>• Overall, nurse practitioner prescribers were 40% less likely to prescribe an antibiotic than medical specialists and 51% less likely to prescribe a 'Watch' antibiotic than specialists
**Eswatini**	Ness et al., 2021 (13)	• Assess antimicrobial use before and after the implementation of the WHO STGs to improve their use<br>• 100 random patient encounters were selected to assess if the indication, duration, and dose were consistent with the STG	• After implementation of the STG, there was a significant decrease in the proportion of clinic visits where an antibiotic was prescribed ($p < 0.001$)<br>• Incorrect indications for prescribing decreased from 20.4% in the initial period to 10.31% and 10.2% of prescriptions in subsequent periods<br>• Incorrect dose/duration decreased from 10.47% in the initial period to 7.37% and 3.1% in the subsequent periods<br>• All prescribers felt that the STG positively impacted on their prescribing
**Ethiopia**	Wendie et al., 2021 (14)	• Assess drug use patterns in PHCs using WHO/INRUD indicators<br>• Data collected retrospectively from 1500 prescriptions, with encounters also observed<br>• Pharmacy heads at participating PHCs were also interviewed	• The average number of drugs per encounter was 2.1, with antibiotics included in 44% of encounters. The most commonly prescribed antibiotics were amoxicillin - 20.75% of antibiotics prescribed, ciprofloxacin - 16% and doxycycline -15%<br>• None of the health centers had an essential drug list<br>• On average, patients spent 4.7 min for consultation and 105 seconds for dispensing, with 54.8% of patients surveyed having adequate knowledge of their medication
	Dereje et al., 2023 (15)	• Assess antibiotic prescribing practices and potential determinants<br>• 1200 encounters were reviewed including 2354 individual medicines<br>• Prescribers include physicians, health officers, nurses and midwives	• Antibiotics constituted 35.3% of all medicines prescribed<br>• 55.3% of prescribed antibiotics were from the Watch group, 43.1% from the Access group and 1.7% from the Reserve group<br>• Antibiotic prescriptions were 2.5 times more likely for patients under 18 years than those 65 years or older, with men more likely to be prescribed antibiotics than women
	Abebe et al., 2024 (16)	• Assesses the prevalence and pattern of inappropriate antibiotic prescribing in ambulatory care visits in Ethiopia<br>• 911 prescriptions were fully analyzed for appropriateness	• 55.3% of all prescriptions contained at least one antibiotic<br>• 2640 antibiotics were contained in the 911 prescriptions with amoxicillin (19.73%), ciprofloxacin (15.83%) and amoxicillin/clavulanic acid (13.86%) the most prescribed antibiotics<br>• 49.54% of antibiotics prescribed were non-compliant with the NTG in 46% of patients, with inappropriate prescribing significantly higher amongst patients prescribed amoxicillin/clavulanic acid

	Abejew et al., 2024 (17)	• Assess the factors affecting antibiotic prescribing behaviors of physicians<br>• Cross-sectional study using a self-administered questionnaire<br>• 185 physicians took part	• Overall, physicians prescribed antibiotics for 54.8% of weekly outpatient encounters, with 96.2% estimating they prescribed antibiotics for patients with symptoms of a URTI<br>• Physicians aged ≤30 years were less likely to prescribe antibiotics for patients who presented with URTIs than others and general practitioners were less likely to prescribe antibiotics for patients who presented with URTIs than residents<br>• During outpatient visits, physicians ≤30 years old were less likely to prescribe antibiotics than others, male physicians were less likely to prescribe antibiotics than female physicians, and general practitioners were less likely to prescribe antibiotics than residents<br>• Physicians with good knowledge were less affected by perceived social pressures than those with poor knowledge and felt it was easier to make rational prescribing choices compared with those with poor knowledge
	Altaye et al., 2024 (18)	• Explore factors affecting antibiotic prescribing at PHCs in Ethiopia<br>• In-depth interviews with 20 prescribers and 24 key informants including pharmacy case team co-ordinators working at the PHCs	• Key factors affecting the prescribing of antibiotics included not updating knowledge on antibiotic use and ABR, not reviewing patient history and competency issues<br>• Key patient-related factors included low awareness about antibiotics, lack of respect for prescribers and pressure on prescribers to prescribe antibiotics<br>• System-related factors included a lack of updated AMR information, patient workloads, inadequate capacity and inadequate follow-up and support
**Guinea**	Eibs et al., 2020 (19)	• Address the knowledge gap regarding antibiotic use in Medecins Sans Frontiéres (MSF) projects by exploring antibiotic prescription habits and drivers<br>• In-depth interviews and FDGs (2) involving prescribers (6) and patients	• Most physicians in Guinea-Bissau reported they knew current guidelines and dosages; consequently, they only consulted protocols on rare conditions<br>• However, concerns with ABR due to uncontrolled use of antibiotics by ambulatory care prescribers and local pharmacies<br>• As a result, prescribers in MSF clinics typically prescribe 2nd and 3rd line choices with lack of local ABR data<br>• In addition, typically prescribe more antibiotics than necessary to be 'on the safe side' with nurses at the PHCs following such patterns
**Madagascar**	Ardillon et al., 2023 (20)	• Characterize inappropriate antibiotic prescribing among young children visiting outpatients and to identify its determinants in 3 LMICs including Senegal<br>• Data included from the prospective BIRDY cohort study including children at birth and followed up for 3 to 24 months<br>• 572 children were enrolled in Cambodia, 1,816 in Madagascar and 331 in Senegal	• Out of 11,762 outpatient consultations, 29.3% led to antibiotic prescriptions across the 3 countries<br>• 76.5% of consultations resulting in antibiotic prescriptions were determined not to require antibiotics - ranging from 71.5% in Madagascar to 83.3% in Cambodia. Among the 10,416 consultations (88.6%) determined not to require antibiotics, 25.3% none the less resulted in antibiotics being prescribed with lower rates in Madagascar (15.6%) vs. Cambodia (57.0%) or Senegal (57.2%)<br>• In Madagascar, the diagnoses accounting for the greatest absolute share of inappropriate prescribing was gastroenteritis without blood in the stool<br>• The most frequently prescribed inappropriate antibiotic was amoxicillin<br>• Factors associated with an increased risk of inappropriate antibiotics were patient's age greater than 3 months and rural vs. urban setting
**Malawi**	Dixon et al., 2021 (21)	• Increase understanding regarding the patterns of antibiotic prescribing and use and the reasons behind this	• Amoxicillin was overall the most prescribed/used antibiotic (range: 13.5% in Uganda to 53.0% in Zimbabwe), followed by cotrimoxazole (range: 8.1% in Uganda to 37.9% in Malawi) and metronidazole (range: 6.1% in Malawi to

		• Data collection involved household surveys using a structured questionnaire<br>• 1811 households participated of which 51.0% were in Malawi, 24,8% in Uganda and 24.0% in Zimbabwe and 24.8%	28.5% in Uganda), with a greater proportion of households frequently using one or more antibiotics from the Access group (range: 68.8% in Zimbabwe to 94% in Uganda) than the Watch group (up to 25.0% in Zimbabwe).<br>• Purchasing antibiotics apart from cotrimoxazole (typically prescribed for a range of illnesses in Malawi apart from HIV as provided by donors) was common from private pharmacies/ informal sellers as stockouts in clinics were common even though medicines were provided free in public facilities
	MacPherson et al., 2022 (22)	• Undertake an in-depth portrayal of antibiotic primary care prescribing practices in rural Malawi and interrogate how the broader funding landscape shapes care practices<br>• Observe 1348 health worker-patient consultations and undertake 49 in-depth interviews with healthcare staff and patients using a structured questionnaire	• Medicines, including antibiotics, were conceptualised by both prescribers and patients as central to care, with all consultations resulting in a prescription and/or dispensing of medicines<br>• This was exacerbated by limited time with each patient (typically 200 patients/ clinic), with the diagnosis and prescription contained in patients' health passports before handing them back. In addition, fear of criticism among prescribers for not prescribing medicines, including antibiotics, during the consultation
**Senegal**	Ardillon et al., 2023 (20)	• Characterize inappropriate antibiotic prescribing among young children visiting outpatients and to identify its determinants in 3 LMICs including Senegal<br>• Data included from the prospective BIRDY cohort study including children at birth and followed up for 3 to 24 months<br>• 572 children were enrolled in Cambodia, 1,816 in Madagascar and 331 in Senegal	• Out of 11,762 outpatient consultations, 29.3% led to antibiotic prescriptions across the 3 countries<br>• 76.5% of consultations resulting in antibiotic prescriptions were determined not to require antibiotics - ranging from 71.5% in Madagascar to 83.3% in Cambodia. Among the 10,416 consultations (88.6%) determined not to require antibiotics, 25.3% none the less resulted in antibiotics being prescribed with lower rates in Madagascar (15.6%) vs. Cambodia (57.0%) or Senegal (57.2%)<br>• In Senegal, uncomplicated bronchiolitis accounted for the greatest proportion of inappropriate prescriptions (84.4%), with cefixime the most frequently prescribed inappropriate antibiotic<br>• Factors associated with an increased risk of inappropriate antibiotics were patient's age greater than 3 months and rural vs. urban setting
**Sierra Leone**	Kabba et al., 2020 (23)	• Assess KAP regarding the prescribing of antibiotics by medical doctors<br>• The first part of the study involved a cross-sectional survey using a self-administered questionnaire to assess doctors' KAP<br>• 119 doctors took part (91.5% response rate)	• Overall, sound knowledge regarding AMR, with generally a positive attitude towards prescribing antibiotics<br>• However, 68% of doctors believed that antibiotics may speed up recovery from a cold or cough, with 21% agreeing that antibiotics may speed up the recovery when prescribed for patients with malaria<br>• Overall, children <5 y of age were more likely to be prescribed an antibiotic than pregnant women/lactating mothers
	Koroma et al., 2024 (24)	• Assessed HCW's (including physicians) knowledge, attitudes, and prescribing practices toward antibiotics and associated factors<br>• Cross-sectional survey using a pre-tested questionnaire<br>• 337 HCWs took part including 84 doctors (25% of the total participants)	• Overall, good knowledge of antibiotics among HCWs, with the highest proportion among medical doctors (89%)<br>• Encouragingly, 91% and 94% of HCWs surveyed strongly agreed and 72% agreed that ABR is a problem worldwide, in Sierra Leone, and in their health facilities, respectively. In addition, 98% strongly agreed or agreed that standard treatment guidelines are useful for prescribing antibiotic especially as 80% of HCWs had not received any training on AMR in the past year<br>• 87% of those surveyed strongly agreed or agreed that the more antibiotics are used, the higher the risk of ABR. In addition, 93% strongly agreed or agreed

			that patients should complete the full course of antibiotics even when there is an improvement to reduce ABR<br>• However, 51% of HCWs surveyed had prescribed antibiotics because they were uncertain of the diagnosis and 41% strongly agreed that antibiotics are indicated in most infectious disease cases
**Uganda**	Lyus et al., 2020 (25)	• Assess the proportion of essential and non-essential antimicrobials that are registered on the drug registers in Kenya, Tanzania and Uganda for prescribing<br>• Categorized all antimicrobials on the national drug registers and EMLs using the British National Formulary as well as the AWaRe classification	• In 2018, Kenya had 2105 registered antimicrobials, Uganda 1563 and Tanzania had 1327. Of these 64.3% were non-essential in Kenya, 51.1% in Uganda and 53.2% in Tanzania<br>• Kenya had 160 antimicrobials on its EML, Uganda 187 and Tanzania 182; of these, 20.7% were not registered in Kenya, 26.7% not registered in Uganda and 28.6% not registered in Tanzania<br>• Of essential Access antibiotics, 14.3% were not registered in Kenya, 8.6% not registered in Uganda and 20.5% not registered in Tanzania; and of essential Watch antibiotics 25.0% were not registered in Kenya, 14.3% not registered in Uganda and 19.1% not registered in Tanzania
	Bonniface et al., 2021 (26)	• Evaluated antibiotic prescribing practices in a rural district via a cross-sectional study involving a retrospective review of 500 prescriptions from five health facilities<br>• Appropriateness assessed using WHO and facility indicators	• In 23% of encounters, one or more antibiotics were prescribed, with 1.3 the mean number of antibiotics/ prescription<br>• 27% of the diagnoses and 42% of the prescriptions were noncompliant with the STG – with the prescribing antibiotics for nonbacterial, e.g. malaria (32%), noninfectious conditions such as dysmenorrhea (15%) and nonspecific diagnosis such as RTIs (40%) considered noncompliant with STGs<br>• On average, only 68% of antibiotics prescribed were available on the day of the visit to the health facilities
	Dixon et al., 2021 (21)	• Increase understanding regarding the patterns of antibiotic prescribing and use and the reasons behind this<br>• Data collection involved household surveys using a structured questionnaire<br>• 1811 households participated of which 51.0% were in Malawi, 24.8% in Uganda and 24.0% in Zimbabwe	• Amoxicillin was overall the most prescribed/used antibiotic (range: 13.5% in Uganda to 53.0% in Zimbabwe) - followed by cotrimoxazole (range: 8.1% in Uganda to 37.9% in Malawi) and metronidazole (range: 6.1% in Malawi to 28.5% in Uganda), with a greater proportion of households frequently using one or more antibiotics from the Access group (range: 68.8% in Zimbabwe to 94% in Uganda) than the Watch group (up to 25.0% in Zimbabwe)<br>• Use of metronidazole was common in Uganda as could be purchased relatively cheaply and made symptoms, if not completely better, at least tolerable<br>• Catastrophic healthcare costs could be mitigated through borrowing medicines to treat acute illness and reciprocating at a later date with leftover antibiotics, with purchasing of antibiotics common from private pharmacies/ informal sellers as stockouts in clinics were common and high co-payments
	Kagoya et al., 2021 (27)	• Explore the experiences and views of HCPs regarding antibiotic prescribing<br>• Exploratory qualitative study using semi-structured interviews<br>• Overall, 56 HCPs took part in interviews and workshops including physicians working in outpatients/ ambulatory care	A number of key themes emerged. These included:<br>• HCPs were aware of the existence of the problem of AMR. However, they did not have sufficient knowledge of its burden and impact, and they believed others should take ownership of the problem including improving sanitation<br>• Most HCPs did not have time to discuss and share information about the correct use of antibiotics with their patients<br>• They also had CHWs demanding antibiotics from them for their use as well as to distribute to their respective communities exacerbated by high rates of

			poverty amongst patients making antibiotics unaffordable from private pharmacies
	Allwell-Brown et al., 2022 (28)	• Document patterns and determinants of antibiotic prescribing for febrile children under-five attending outpatients at primary and secondary healthcare facilities<br>• Retrospective analysis of outpatient registers in 37 healthcare facilities<br>• Data was collected on 3598 febrile under-five children	• Of 9745 prescriptions, 42.2% were for antimicrobials of which 63.5% were antibacterials<br>• Acute URTIs were the commonest single indication for antibiotic prescribing, with 76.3% of children with this indication receiving at least one antibiotic prescription. 96.6% of children with a diagnosis of pneumonia prescribed antibiotics<br>• Most antibiotics prescribed were from the Access group - amoxicillin (52.2% of antibiotics prescribed), co-trimoxazole (14.7%), metronidazole (6.9%) and gentamicin (5.7%), with limited prescribing of Watch antibiotics - ceftriaxone (5.3%)<br>• Higher health centre levels as well as private-not-for-profit ownership were significant contextual determinants of antibiotic prescribing
	Obakiro et al, 2022 (29)	• Evaluate outpatient antibiotic prescribing among HCPs and whether this is in accordance with current STGs<br>• Cross-sectional retrospective review of health management information systems<br>• 4312 observations were included in the analysis	• Overall, 82.6% non-adherence to guidelines, with male HCPs twice as likely to non-adhere to STGs vs. female prescribers<br>• Respiratory diseases made up 20.9% of infectious diseases, lower than genitourinary diseases (26.9%)<br>• Most prescribed antibiotics were the penicillins (32.6%) followed by the macrolides (7.2%)
	Mambula et al., 2023 (30)	• Obtain insights regarding their perspectives on the use of antibiotics<br>• Semi-structured interviews with HCWs (doctors, nurses, pharmacists/ dispenses) and FGDs with caregivers<br>• In Uganda, 18 HCWs participated in semi-structured interviews and 24 caregivers in FGDs (Overall 59 in Niger including 29 HCWs)	• In both Uganda and Niger, there were knowledge gaps regarding the use of antibiotics among HCWs<br>• Non-availability of antibiotics was common among healthcare facilities in both countries - with implications for the choice of prescribed antibiotic, the duration and adherence in practice<br>• Among caregivers – there was awareness of specific antibiotic names but not the word 'Antibiotic'
	Murungi et al., 2023 (31)	• Determine the national consumption of all antimicrobials in Uganda as a reflection of prescribing and dispensing behaviour<br>• Consumption by antibiotic class and DDDs/ 1000 inhabitants and AWaRe classification	• In 2021, the average DDD per 1000 inhabitants was 29.02 for all antimicrobials - 80.7% were oral<br>• Penicillins (27.6%) were the most consumed antimicrobial class, followed by sulfonamides and trimethoprim (15.5%)<br>• 62.91% of current utilisation was from the Access class, with those from the Watch class averaging 14.51% between 2018–2021, spiking in 2021 (34.2%) during COVID-19 pandemic compared to 2020 (24.29%). Azithromycin and ciprofloxacin were the most consumed Watch class in 2021<br>• Overall, very limited use of Reserve antibiotics
	Igirikwayo et al., 2024 (32)	• Assess antibiotic prescription patterns and associated factors among outpatients with RTIs<br>• Mixed method study involving data abstraction from all patient records with a	• 79.8% were prescribed antibiotics, 40.9% for un-categorized ARTIs, 28.5% for un-categorized URTIs and 8.13% for common colds<br>• 86.6% of prescribed antibiotics were from the Access group with amoxicillin accounting for 50.45% of all antibiotic prescriptions and 13.4% were from the Watch group with azithromycin (4.56% of all antibiotics) and erythromycin

		• diagnosis of RTIs (1542 records after data cleaning)<br>• Interviewer-administered questionnaire among 30 drug prescribers (40% nurses) in relevant health facilities	(3.9%) the most prescribed. No antibiotics were prescribed from the Reserve group<br>• Overall high rates of inappropriate prescribing (74.4% to 74.9%) when compared with European guidelines<br>• Encouragingly prescribers who had access to Uganda STGs and Integrated Management of Childhood Illness guidelines were less likely to prescribe antibiotics. Prescribers who had not received training on antibiotic use were 3.55 times more likely to prescribe antibiotics
	Lundin et al., 2024 (33)	• Assess the prevalence of antibiotic use among children under five visiting private healthcare providers with diarrhea following an intervention to increase the use of oral rehydration salts	• The prevalence of antibiotic use among children under five with diarrhoea decreased from 30.5% (pre-intervention) to 20.0% (p < 0.001) post-intervention<br>• There was no significant association between antibiotic prescriptions, the location of the medical outlet or the sex/ age of the child
	Olamijuwon et al., 2024 (34)	• Assess the differences in treatment-seeking behaviours and antibiotic use for UTI-like symptoms among patients visiting healthcare clinics before and during the COVID-19 pandemic	• Over time, there was a significant increase in the use of antibiotics not recommended for treating UTIs in the NTG<br>• The use of metronidazole use increased from 30% to 53% and doxycycline from 28% to 40% between the pre- COVID-19 and COVID-19 phase
**Zambia**	Kalungia et al., 2019 (35)	• Explore physicians' and pharmacists' knowledge and perceptions regarding AMS<br>• Cross-sectional study using a self-administered questionnaire<br>• 137 physicians and 61 pharmacists participated	• Encouragingly, 95% of those surveyed perceived AMR as a current problem in their practise<br>• However, despite positive perceptions, basic knowledge of AMS was relatively low among physicians (51%), with knowledge levels significantly associated with years of practice and previous AMS training.<br>• In addition, 92% of physicians had not undertaken AMS training before - indicating the need for context-specific educational interventions to promote AMS activities in Zambia
	Mudenda et al., 2024 (36)	• Assess KAP regarding antimicrobial use, AMR and AMS among HCWs involved in the implementation of AMS activities<br>• Cross-sectional study using interviewer-administered questionnaire<br>• 64 HCWs participated including 24 nurses	• Encouragingly, 93.7% of surveyed HCWs agreed that frequent use of antibiotics may decrease their efficacy, 96.9% that their use should be regulated/ controlled and 92.2% of HCWs disagreed that antibiotics should be stopped once someone felt better<br>• In addition, 90.9% agreed that patient's self-medication practices contribute to the inappropriate use of antibiotics<br>• Overall, 75% of the HCWs had good knowledge, 84% were highly aware and 84% stated they had good practices/ knowledge regarding antibiotic use, AMR and AMS – helped by AMS teams in hospitals and increasing implementation/ monitoring of the WHO AWaRe classification of antibiotics
	Yamba et al., 2024 (37)	• Assess antibiotic prescribing patterns in primary healthcare facilities (6 in total)<br>• Prescription pattern data obtained from pharmacies with identification and antimicrobial susceptibility determined by conventional methods	• Most prescribed antibiotics among the facilities belonged to the Acces' group (74%), with all PHC facilities adhering to the AWaRe guidance of ≥60% of prescribed antibiotics belonging to the Access group<br>• However, appreciable resistance to Access antibiotics was seen - *E. coli* resistance to ampicillin ranged from 71% to 77% and to co-trimoxazole from 74% to 80%, with *enterococcal* resistance to tetracycline between 59%–64%<br>• MDR was highest in *E. coli* (75%) isolates with XDR highest in *enterococcal* isolates (97%)

**Zimbabwe**	Dixon et al., 2021 (21)	• Increase understanding regarding the patterns of antibiotic prescribing and use and the reasons behind this<br>• Data collection involved household surveys using a structured questionnaire<br>• 1811 households participated of which 51.0% were in Malawi, 24.8% in Uganda and 24.0% in Zimbabwe	• Amoxicillin was overall the most prescribed/used antibiotic (range: 13.5% in Uganda to 53.0% in Zimbabwe) - followed by cotrimoxazole (range: 8.1% in Uganda to 37.9% in Malawi) and metronidazole (range: 6.1% in Malawi to 28.5% in Uganda), with a greater proportion of households frequently using one or more antibiotics from the 'Access' group (range: 68.8% in Zimbabwe to 94% in Uganda) than the Watch group (up to 25.0% in Zimbabwe)<br>• Amoxicillin was typically seen as the 'drug of choice' for a range of conditions including pneumonia, sinusitis, otitis media and UTIs, with expectations that antibiotics would be prescribed during consultations<br>• Catastrophic healthcare costs could be mitigated through borrowing medicines to treat acute illness and reciprocating at a later date with leftover antibiotics, with purchasing of antibiotics common from informal sellers due to the high costs of antibiotics in pharmacies
	Olaru et al., 2021 (38)	• Explore attitudes and practices regarding microbiology tests, antibiotic prescribing and AMR among HCPs (nurses and midwives) at primary health clinics<br>• Cross-sectional survey using a questionnaire based on a literature review<br>• 91 HCPs participated	• Encouragingly, the decision to prescribe antibiotics was mainly influenced by the clinical presentation and laboratory results (98% of surveyed HCPs), severity of the illness (89%) and national guidelines (97%), with national guidelines often the main source for guiding prescribing in practices (93%) as well as a means to increase antibiotic prescribing knowledge (97%).<br>• In addition, HCPs stated prescribing unnecessary antibiotics sometimes (only 30% of them) and almost never - 21%. Only 8% of HCPs very often and 9% often believed they unnecessarily prescribed antibiotics<br>• However, 89% of HCPs would prescribe antibiotics in a patient with symptoms suggestive of a viral RTI
	Olaru et al., 2021 (38)	• Explore KAP towards microbiology tests, AMR and antibiotic prescribing among HCPs and PHCs (private and public)<br>• Self-administered cross-sectional survey using a structured questionnaire based on the literature<br>• 91 prescribers took part (principally nurses), with 68% more than 10 years' experience	• Decision to prescribe antibiotics was mainly influenced by the clinical presentation and laboratory results (98%) and severity of illness (89%) as well as by the national guidelines (97%). In addition, influenced by patients' (7%) or their seniors' expectations (15%); however, to a more limited extent<br>• Belief that generally limited unnecessary prescribing of antibiotics, i.e. very often (8%), often (9%), about half of the times (32%), and sometimes (30%)<br>• 29% and 46% respectively felt that antibiotic prescriptions should be reduced to reduce AMR. However, 22% of participants were unsure if AMR was a problem in their clinics<br>• 100% of participants felt that availability of national guidelines and training sessions on antibiotic prescribing would help improve future antibiotic prescribing alongside regular audit and feedback on prescribing (93%)
	Olaru et al., 2021 (39, 40); Olaru et al., 2022 (41)	• Assess the impact of antibiotic treatment on clinical and bacteriological outcomes in patients presenting with UTI symptoms to PHCs as well as explore possible causes of negative urine cultures in patients presenting with symptoms of UTI to PHCs	• Effective treatment was taken by 32.1% participants presenting at PHCs with UTIs, with urine cultures negative at follow up in 75.9% of participants who took appropriate treatment<br>• Negative urine cultures were only seen and in 28.9% of patients who did not take appropriate treatment ($p<0.001$)<br>• Overall, symptoms had improved or resolved in 96.3% of those on appropriate treatment vs. only 62.3% ($p<0.001$) of those without appropriate treatment<br>• In a separate analysis of the same patients, there was a high prevalence of STIs and evidence of prior antimicrobial use as possible explanations for the

		• Alongside this, assess possible novel culture systems for the detection of uropathogens and ESBL-producing organisms in patients with UTIs	low proportion of positive urine cultures seen in practice – implications for other African countries with high rates of STIs<br>• A separate analysis also showed a good performance for the novel culture systems for the detection of uropathogens and ESBL-producing organisms with the potential to decentralize laboratory testing
**Lower-Middle Income countries***			
**Cameroon**	Chem et al., 2018 (42)	• Investigate prescribing and predictors of antibiotic prescriptions among PHCs<br>• Cross sectional retrospective study analysing 30,096 prescriptions as well as questionnaires administered to 59 prescribers	• 36.71% of prescriptions contained an antibiotic, with a mean of 1.14 antibiotics prescribed per patient<br>• Amoxicillin was the most prescribed antibiotic - 29.9% of antibiotic prescriptions – followed by cotrimoxazole (19.09%) and metronidazole (15.59%) - with penicillins accounting for 45.82% of all antibiotics prescribed<br>• 21.27% of antibiotics were prescribed for RTIs, 11.75% for uncomplicated malaria and 10.32% for gastroenteritis<br>• Laboratory guidance and patient turnout significantly impacted on antibiotic prescription rates
	Menkem et al., 2023 (43)	• Assess knowledge and use of third-generation cephalosporins among medical doctors practicing in Cameroon<br>• Cross-sectional study using an online questionnaire coupled with review of patients' notes<br>• 54 physicians took part in the online questionnaire	• There was average knowledge regarding third-generation cephalosporins among surveyed physicians, with ceftriaxone being the most widely known and prescribed<br>• However, misuse of cephalosporins was common<br>• Overall, only 32.7% of physicians had correct knowledge regarding the number of generations of cephalosporins, with only 48.1% knowing current antimicrobial targets<br>• In addition, only 17% and 9.4% respectively of physicians knew the correct posology for cefotaxime and ceftazidime
**Côte d'Ivoire (CIV)**	Wieters et al., 2024 (9)	• Assess antibiotic use by WHO AWaRe classification among patients visiting healthcare facilities in 4 African countries including CIV<br>• Infectious diseases surveyed included acute febrile disease of unknown cause (AFDUC), GI infections and RTIs<br>• Out of 19,700 enrolled patients, 36.8% reported antibiotic use in previous the ten days for these infections	• Out of 36.8% of respondents across the countries stating antibiotic use in the previous 10 days, 41.5% were prescribed for RTIs, 30.3% for AFDUC and 22.6% for GI infections<br>• A lower number of enrolled patients in CIV had RTIs treated with antibiotics – 39.6%<br>• The most common antibiotic prescribed was ceftriaxone (31.7% – appreciably lower in CIV at 17.7%). This was the case among patients with AFDUC - ranging from 29.0% of patients in CIV to 62.6% in Burkina Faso<br>• Among those with RTIs in CIV, only 7.8% received ceftriaxone vs. 32.3% amoxicillin/clavulanic acid and 28.2% amoxycillin
**Ghana**	Ghebrehewet et al., 2020 (44)	• Assess the feasibility of implementing different delayed/back-up prescribing models on antibiotic prescribing for URTIs<br>• Patients were assigned to Group 0: No prescription given; Group 1; Patient received post-dated antibiotic prescription; Group 2: Offer of a rapid reassessment by a nurse practitioner after 3 days; and Group 3: Post-dated prescription	• 142 patients met inclusion criteria. Group 0 – 61 patients, Group 1 – 16 patients, Group 2 – 44 patients and Group 3 - 21 patients<br>• The most common diagnosis was a sore throat (73%)<br>• Only one patient took antibiotics after 3 days<br>• 141/142 patients were successfully contacted on day 10, and of these, 102 (72%) rated their experiences as good or very good<br>• Informal discussions with staff revealed improved knowledge of AMR<br>• Overall, delayed/back-up prescribing can be implemented safely in LMICs

		• Patients were contacted 10 days afterwards to ascertain wellbeing and actions taken as well as rate the service (Likert scale)<br>• Post-study informal discussions with staff	
	Opoku et al., 2020 (45)	• Determine the factors associated with anti-biotic prescribing among febrile outpatients seeking care<br>• Secondary data obtained from the medical records of 2519 febrile outpatients and ana-lysed	• Prevalence of antibiotic prescribing for these patients was 70.1%, highest at 83.8% among those under 5<br>• The largest proportion of the prescriptions with antibiotics were written by pre-scribers who had been practising for 3 to 5 years (37.4%).
	Sefah et al., 2021 (46)	• Evaluate adherence to the Ghana STGs for empirical antibiotic treatment of outpatients with CAP<br>• Cross-sectional design extracting pertinent data from medical records using a struc-tured form<br>• 1929 records were reviewed	• There was a 32.50% adherence of prescribers to the Ghana STGs on the choice of antibiotics for CAP, with 7.31% not receiving any antibiotics as part of their management<br>• Adherence was associated with the duration of antibiotic prescribing, number of additional antibiotics prescribed and some patients' clinical characteristics including respiratory symptoms and chest X-rays
	Owusu et al., 2022 (47)	• Assess compliance to STGs among adults with uncomplicated UTIs<br>• 3717 patient records were analyses<br>• 46.2% of prescriptions were from Residents/ Specialists with 53.8% from Physician Assistants/ Medical Officers/ Senior Medical Officers	• 83% of patients were prescribed an antibiotic empirically – lower among pa-tients who underwent routine urine examination (81%) vs others (86%)<br>• Only 68% of patients were prescribed antibiotics for the correct duration - sig-nificantly lower among male (10%) vs. female patients (90%)<br>• Among patients prescribed antibiotics, only 60% were prescribed in line with the STGs. However – no difference by prescriber type
	Pinto Jimenez et al., 2023 (48)	• Assess the awareness of ABR among HCPs from six LMICs in both human and animal health including Ghana<br>• Questionnaire based study using a pre-tested questionnaire among 1091 participants including 726 human HCPs of which 106 were from Ghana (46.2% physicians and 20.2% nurses)	• Higher awareness scores regarding ABR among HCPs in Ghana compared with those from Tanzania, enhanced by 30.8% stating they had training regarding AMR or AMS vs. only 25.4% for Tanzania<br>• 62.5% of human HCPs from Ghana stated their medical decisions on prescribing antibiotics were driven by fear/ worse health outcomes (versus 63.5% from Tanzania and 75.3% of HCPs from Nigeria)<br>• Prescribing decisions influenced by:<br>○ Lack of availability of local resistance data across countries (two thirds of HCPs taking part across countries – 83.7% in Ghana)<br>○ Exposure to company advertising (two thirds of HCPs – 67.3% in Ghana)<br>○ Promotional activities from medical representatives (96.1% - Sometimes/ always)
	Sefah et al., 2023 (49)	• Assess the KAP of HCPs towards AMS<br>• Cross-sectional survey using a validated self-administered electronic questionnaire<br>• 339 HCPs participated from primary and secondary care settings – 13.3% were physicians and 78.2% nurses (the majority – 66.4% from primary care)	• The majority of participants had poor knowledge (91.2% of participants) and poor practice (64.6%); however, good attitude (78.8%) towards AMS<br>• Ongoing exposure to AMS structured training, CPD training on AMS in the previous year, and the number of years of working experience were predictors of HCPs' KAP scores<br>• Overall, concentrated efforts are needed to address current low levels of knowledge and poor practices regarding AMS among HCPs in Ghana

	Sefah et al., 2024 (50)	• Assess the appropriate use of antibiotics for eye infections in an ambulatory clinic<br>• Analyse the medical records of all patients who sought eye care	• 1925 patient medical records were extracted<br>• The appropriate choice of antibiotic prescribed was 42.44% - positivity associated with age, number of antibiotics prescribed, the prescription of topical dosage forms, and WHO Access class
**Kenya**	Kleczka et al., 2019 (51)	• Document changes in antibiotic use for common infections in PHCs following improved documentation and feedback<br>• 889 patient encounters were analysed including adherence to STGs among 9 participating centres	• Overall, antibiotics were prescribed in 94.3% of the 889 patient encounters across the four infectious diseases. These were 97.3% for URTIs, 94.2% for UTIs, 91.6% for STIs and 91.3% for GI infections<br>• Overall, antibiotic use according to the STGs was appropriate in only 58.6% of patients with URTIs and 47.2% with GI infections<br>• Whilst feedback did not affect the number of antibiotics prescribed for UTIs, the use of nitrofurantoin (appropriate narrow-spectrum antibiotic) increased from 9.2% to 29.9%; p<0.0001 and the use of quinolones decreased (30.0% to 16.1%; p<0.05).
	Mekuria et al, 2019 (52)	• Assess the prescribing of antibiotics for ARIs in primary care<br>• Mixed-method study involving analysis of claims data and interviews with 12 physicians (and 17 patients)	• Out of 36,210 clinic visits by 21,913 patients, 45,706 diagnoses were made with 85,484 prescriptions<br>• Acute URTIs were the most common diagnoses (39.7%) followed by other ARIs (24.9%) with antibiotics prescribed in 78.5% of patients with diagnosed ARIs<br>• Amoxicillin was the most prescribed antibiotic for patients with ARIs (45%) followed by azithromycin (12.5%)<br>• High prescription rates were exacerbated by high patient work loads, clinician and patient perceptions that antibiotics should be prescribed for ARIs, absence of any policies and surveillance and lack of current guidelines
	Rhee et al., 2019 (53)	• Assess the level of inappropriate antibiotic prescription for childhood diarrhoea among PHCs<br>• Examined the frequency of antibiotic over-prescription (for non-dysentery diarrhoea), under-prescription (no antibiotic prescription for dysentery) and inappropriate antibiotic selection (non-recommended antibiotics) based on patient records<br>• Overall, 7505 visits for final analyses	• In one site, 95.6% of visits were for non-dysentery diarrhoea and the prevalence of antibiotic over-prescription was 52.5%. 4.4% were dysentery visits and the prevalence of under-prescription of antibiotics was 26.8%<br>• In another site, 96.2% of visits were for non-dysentery diarrhoea and the prevalence of antibiotic over-prescription was 20.0%. 3.8% were dysentery visits and the prevalence of antibiotic under-prescription was 73.7%<br>• Excessive prescribing of antibiotics was associated with concomitant gastroenteritis or RTIs while a malaria diagnosis was negatively associated with antibiotic over-prescriptions but positively associated with under-prescriptions
	Lyus et al., 2020 (25)	• Assess the proportion of essential and non-essential antimicrobials that are registered on the drug registers in Kenya, Tanzania and Uganda for prescribing<br>• Categorized all antimicrobials on the national drug registers and EMLs using the British National Formulary as well as the AWaRe classification	• In 2018, Kenya had 2105 registered antimicrobials, Uganda 1563 and Tanzania had 1327. Of these 64.3% were non-essential in Kenya, 51.1% in Uganda and 53.2% in Tanzania<br>• Kenya had 160 antimicrobials on its EML, Uganda 187 and Tanzania 182; of these, 20.7% were not registered in Kenya, 26.7% not registered in Uganda and 28.6% not registered in Tanzania<br>• Of essential Access antibiotics, 14.3% were not registered in Kenya, 8.6% not registered in Uganda and 20.5% not registered in Tanzania; and of essential Watch antibiotics 25.0% were not registered in Kenya, 14.3% not registered in Uganda and 19.1% not registered in Tanzania

	Sulis et al. 2020 (54)	• Estimate the proportion of Standardised Patient (SP) – provider interactions resulting in inappropriate antibiotic prescribing in primary care in LMICs including Kenya<br>• Secondary analysis of published SP studies<br>• AWaRe classification used to categorise antibiotic utilisation	• Across health facilities, antibiotics were given inappropriately on 50.0% of occasions in Kenya (Nairobi) for SPs presenting with either watery diarrhoea, presumptive tuberculosis (TB), angina or asthma<br>• The prevalence of antibiotic overuse in India was significantly lower in urban versus rural areas – however, similar in Kenya<br>• Access antibiotics were predominantly prescribed in Kenya (85%)
	Hooft et al., 2021 (55)	• Assess the extent of antibiotic prescribing among children in Kenya presenting in clinics with undifferentiated febrile illness<br>• Examining the extent of antibiotic use among all pertinent children presenting at one of five clinic sites in the study<br>• 5735 children were analysed	• 68% of children were prescribed antibiotics despite only 28% being diagnosed with a bacterial illness – 14% at primary diagnosis and 14% as a differential diagnosis<br>• Among those with a primary diagnosis of bacterial illness - 86% were prescribed an antibiotic<br>• In children with a viral disease diagnosis (primary diagnosis) 84% were prescribed antibiotics<br>• Amoxicillin, was the most frequently prescribed antibiotic (59% of those prescribed an antibiotic) followed by amoxicillin-clavulanate (20%)
	Kwan et al., 2022 (56)	• Examine the effects on quality of care when patients demand inappropriate medicines<br>• Unannounced standardised patients (SPs) in private clinics as caretakers of children with watery diarrhoea<br>• 200 visits to private clinics with half of the SPs demanding amoxicillin and the others demanding albendazole (antiparasitic)	• Among the visits, 15% resulted in a correct diagnosis or suspicion of watery diarrhoea, and 75% of the visits were correctly managed with 31% of SPs asked to return and 6% referred elsewhere<br>• However, for all visits regardless of SP demands, the most frequently prescribed antibiotics were metronidazole (27% of patients), sulfamethoxazole and trimethoprim (19%), metronidazole benzoate (12%) and amoxicillin (10%)
**Nigeria**	Chukwu et al., 2021 (57)	• Assess knowledge, attitude and prescribing behaviour of HCWs<br>• Cross-sectional study using a structured self-administered questionnaire<br>• 358 HCWs took part (85.2% response rate)	• Encouragingly, 49.2% of participating HCWs had good knowledge of AMR, with physicians significantly better knowledge than other HCWs. In addition, 70.9% stated they frequently or moderately used STGs<br>• However, 50.3% agreed their prescribing behaviour could promote AMR<br>• Participants also stated they prescribed antibiotics for common viral infections including sore throats (75.7%), measles (37.7%), common colds and influenza flu (21.2%)<br>• In addition, 60.3% admitted prescribing antibiotics just to be on the safe side
	Ogaji (58) et al., 2023	• Assess the quality of prescribing in PHCs according to WHO/ INRUD criteria<br>• Direct observation of 325 consecutive patient encounters in the selected PHCs and 1300 retrieved prescriptions	• 3805 medications were prescribed among the 1300 encounters - giving 2.9 (± 0.5) medications prescribed/encounter<br>• 75.6% of the medicines prescribed were from the Nigeria essential medicine list for PHCs, with pharmacies stocking 88.7% of the key medications<br>• Antibiotics were the most prescribed medicines - included within 62.6% of prescriptions
	Pinto Jimenez et al., 2023 (48)	• Assess the awareness of ABR among HCPs from six LMICs in both human and animal health including Nigeria<br>• Questionnaire based study using a pre-tested questionnaire among 1091 participants including 726 human HCPs of	• Higher awareness scores regarding ABR for HCPs in Nigeria compared with those from Tanzania, enhanced by 35.3% stating they had training regarding AMR or AMS vs. only 25.4% for Tanzania<br>• 75.3% of human HCPs from Nigeria stated their medical decisions on prescribing antibiotics were driven by fear/ worse health outcomes (versus 63.5% from Tanzania and 75.3% 62.5% of HCPs from Ghana)

		which 112 were from Nigeria (64.7% physicians)	• Prescribing decisions influenced by:<br>○ Lack of availability of local resistance data across countries (two thirds of HCPs taking part across countries – 77.6% in Nigeria)<br>○ Exposure to company advertising (two thirds of HCPs – 61.2% in Nigeria)<br>○ Promotional activities from medical representatives (91.8% - Sometimes/ always)
	Chukwu et al., 2024 (59)	• Explore prescriber experiences and perceptions regarding the usefulness and feasibility of strategies to enhance the implementation of ASPs and the challenges across primary care and hospital facilities<br>• Cross-sectional mixed-method survey including using a structure questionnaire and FGDs<br>• 25 questionnaires analysed for the quantitative section and 20 HCWs (physicians and pharmacists) took part in the FGD	• Encouragingly, 96.0% of respondents believed ABR was a problem in Nigeria; however, only 84.0% agreed/strongly agreed that ABR was a problem in their facility, with pharmacists (100%) were more likely to agree that ABR was a problem vs. physicians (78.6%). Overall, 80.0% of respondents (pharmacists and physicians) believed inappropriate prescribing of antibiotics was a problem in their facilities<br>• Approximately half of the participants either did not have, or were not sure if, an AMS team existed in their facility. 68.0% agreed that ASPs were needed to effectively deal with ABR; however, they would need a lot of help to implement ASPs with ASPs (if they exist) typically at an early stage in facilities<br>• Overall, participants believed establishing passionate ASP teams would help improve future antibiotic prescribing in facilities. However, concerns with the lack of resistance data and available personnel for ASPs
**Tanzania**	Lyimo et al., 2018 (60)	• Assess KAP toward antibiotics among HCPs (principally nurses, assistant medical officers and clinical officers)<br>• Descriptive study using a structured questionnaire based on previous studies<br>• 217 HCPs took part (98.6% response rate)	• Encouragingly, 51.2% of HCPs strongly agreed that inappropriate prescribing of antibiotics puts patients at risk and 50.7% stated they were influenced to prescribed antibiotics by positive microbiological results in symptomatic patients<br>• In addition, 62.7% reported they had access to/used antibiotic therapy guidelines when prescribing antibiotics<br>• However, 51.6% stated that their decision to start antibiotic therapy was influenced by a patient's clinical condition<br>• Alongside this, only 24.0% of HCPs had received regular training and education regarding antibiotic prescription practices, with the authors concluding that training and education is needed for HCPs to improve the appropriateness of their antibiotic prescribing
	Lyus et al., 2020 (25)	• Assess the proportion of essential and non-essential antimicrobials that are registered on the drug registers in Kenya, Tanzania and Uganda for prescribing<br>• Categorized all antimicrobials on the national drug registers and EMLs using the British National Formulary as well as the AWaRe classification	• In 2018, Kenya had 2105 registered antimicrobials, Uganda 1563 and Tanzania had 1327. Of these 64.3% were non-essential in Kenya, 51.1% non-essential in Uganda and non-essential 53.2% in Tanzania<br>• Kenya had 160 antimicrobials on its EML, Uganda 187 and Tanzania 182; of these, 20.7% were not registered in Kenya, 26.7% not registered in Uganda and 28.6% not registered in Tanzania<br>• Of essential Access antibiotics, 14.3% were not registered in Kenya, 8.6% not registered in Uganda and 20.5% not registered in Tanzania; and of essential Watch antibiotics 25.0% were not registered in Kenya, 14.3% not registered in Uganda and 19.1% not registered in Tanzania
	Emgard et al., 2021 (61)	• Describe PHC workers' experiences of antibiotic prescribing for children < 5 years and their beliefs regarding ABR	• Concerns that some HCPs prescribe antibiotics for children with fever without a focus and/or negative laboratory results based on their previous experience

		• In-depth interviews with 20 prescribing PHC workers including physicians, clinical officers and nurses	• Most mothers expect HCPs to prescribed an antibiotic for their child with an infectious disease whatever the cause or have encountered children in whom an antibiotic was initiated at home before the visit<br>• Children not finishing the course of prescribed antibiotics and/ or not adhering to the recommended time intervals for administration of antibiotics was seen as common among participating HCPs<br>• Most HCPs were aware of the challenges associated with ABR; however, few experienced ABR as a problem in their daily practice
	Khalfan et al., 2021 (62)	• Determine the prevalence as well as describe patterns of antibiotic prescriptions among insured patients<br>• Cross-sectional analysis of claim forms<br>• 993 insured patients were analysed	• 46.4% of patients were prescribed antibiotics; of these 19.9% received more than one antibiotic<br>• The most common antibiotics prescribed were co-amoxiclav (17.1%), amoxicillin (16.5%), ampicillin/ cloxacillin FDC (14.8%), metronidazole (10.6%) and ciprofloxacin (9.5%)<br>• 60.8% were from the Access group, 33.3% in the Watch group and 17.4% in the not recommended group. None were from the 'Reserve' group<br>• 92.2% of the antibiotics prescribed were in agreement with the Tanzania STG
	Kilipamwambu et al., 2021 (63)	• Assess antibiotic prescribing patterns at health care facilities in Tanzania<br>• One-year retrospective cross-sectional study<br>• 604 prescriptions were reviewed	• 1203 drugs were prescribed from 604 analysed prescriptions - 1.99 medicines prescribed/ consultation<br>• The majority of analysed patients had either URTIs (33.3%), UTIs (31.1%) or diarrhoea (21.2%)<br>• Out of the 1203 medicines prescribed, 51.9% were for antibiotics. Of the 624 antibiotics prescribed, amoxicillin was the most common (22.7%), followed by ciprofloxacin (13.6%) and metronidazole (11.6%)<br>• 97.6% of the antibiotics prescribed appeared on the EML
	Van de Maat et al., 2021 (64)	• Provide insight into the case management of febrile children under 5 years in primary care facilities as well as identify areas for improving the quality of care<br>• Prospective study from the ePOCT trial<br>• 547 febrile children aged 2–59 months treated in primary care facilities were included	• Most diagnoses of children coming to the clinics were viral in origin, e.g. URTIs (60%) and/ or gastro-enteritis (18%)<br>• 95% of presenting children were prescribed an antibiotics; however, only 22% of presenting children had an indication for antibiotics based on local guidelines<br>• The doses of antibiotics prescribed were frequently out of recommended ranges, with non-recommended treatments common (29%)<br>• Vital signs, anthropometric measurements and urinary testing typically failed to influence treatment decisions
	Wiedenmayer et al., 2021 (65)	• Investigate prescribers' adherence of diagnoses and treatments according to national STGs<br>• Cross-sectional study involving 120 randomly public primary care facilities and 2886 patient cases. However, no data on either diagnosis or treatment in 14 cases	• The most prevalent conditions seen in the primary care facilities were URTIs (25%), malaria (18%), diarrhoea (9.9%), pneumonia (6.1%) and skin problems (5.8%)<br>• Complete adherence to STGs for all 25 illness groups evaluated was seen in 29.9% of cases, partial adherence in 38.7% of cases and non-adherence in 30.9%<br>• Highest non-adherence rates were for URTIs and diarrhoea<br>• The wrong medication was given in 30.9% of cases<br>• Overall, 61% of patients were prescribed an antibiotic regardless of the diagnoses - exacerbating non-adherence rates

	Khalfan et al., 2022 (66)	• Identify factors influencing antibiotic prescribing among insured patients (ambulatory care/ in-patients)<br>• Cross sectional study using specific data collection forms<br>• 993 patients were included in the analysis	• Overall, 46.4% of patients were prescribed an antibiotic - 65.4% of children vs. 45.2% for adults and 23.0% for the elderly, greatest (77.0%) in those attending lower-care facilities<br>• Greater prescribing of antibiotics among assistant medical/ clinical officers vs. physicians or specialists (1.9 times higher among clinical officers)<br>• Prevalence of being prescribed an antibiotic was approximately 4 times higher in patients with chronic rhinitis, nasopharyngitis or pharyngitis versus those with no such diagnosis and 1.7 times higher in children vs. adults
	Mabilika et al., 2022 (67)	• Ascertain current prevalence rates and predictors of antibiotic prescribing in primary healthcare facilities<br>• Cross-sectional study of the medical records<br>• Overall, 1021 consultations were analysed - 94.12% public primary healthcare facilities<br>• Clinical officers and assistants accounted for 95.2% of consulted personnel – with only 4.8% physicians	• An antibiotic was prescribed in 76.3% of consultations, with amoxicillin (over 30%) and cotrimoxazole (over 29%) the most prescribed antibiotics, with limited prescribing of 'Watch' antibiotics<br>• Overall, amoxicillin and cotrimoxazole accounted for over 60% of antibiotic prescriptions<br>• The most common empirical diagosis among presenting patients were URTIs (30.3%), UTIs (12.1%) and diarrhoea (7.7%), with prescribing of antibiotics by clinical officers almost 2.55 times higher than seen among medical doctors. This needs urgent addressing to reduce AMR with clinical officers accounting for the majority of prescribers in public PHCs in Tanzania<br>• Only 44.9% % of antibiotic prescriptions adhered to the current STGs
	Pinto Jimenez et al., 2023 (48)	• Assess the awareness of ABR among HCPs from six LMICs in both human and animal health involving Tanzania<br>• Questionnaire based study using a pre-tested questionnaire among 1091 participants including 726 human HCPs of which 126 were from Tanzania (25.4% physicians and 44.4% nurses)	• HCPs from Tanzania had the lowest awareness scores regarding ABR among the 6 studied LMICs, with only 25.4% of HCPs stating they had training regarding AMR or AMS<br>• 63.5% of human HCPs from Tanzania stated their medical decisions on prescribing antibiotics were driven by fear/ worse health outcomes (versus 62.5% in Ghana, and 75.3% of HCPs in Nigeria)<br>• Prescribing decisions influenced by:<br>○ Lack of availability of local resistance data across countries (two thirds of HCPs taking part across countries – 69.8% Tanzania)<br>○ Exposure to company advertising (two thirds of HCPs – 50.8% Tanzania)<br>○ Promotional activities from medical representatives (84.9% - Sometimes/ always)
	Virhia et al, 2023 (68)	• Explore HCPs' motivation to engage in health matters and broader roles in community health including KAP surrounding AMR and any constraints in their daily practices<br>• 24 in-depth interviews and focus-group discussions using structured questionnaires	• Variable knowledge surrounding bacteria, viruses, and the causes of common infections with several misperceptions, e.g., anthrax as a virus, malaria can be transmitted sexually, and women can acquire UTIs from stepping in unclean water or on soil contaminated with urine<br>• Having said this, most medical officers and nurses could articulate their understanding of antibiotics and AMR, with HCPs demonstrating their awareness of issues relating to AMR. However, AMR was not always understood in relation to infectious diseases<br>• A concern is that patients often do not take their antibiotics as prescribed and often stopped their antibiotics when they begin to feel better<br>• Obstacles to improving antibiotic use include poor physical infrastructures, diagnostic capacity, staffing levels, and access to treatment

	Lamshöft et al, 2024 (69)	• Assess the pre-management of children treated in hospital with symptoms of fever and/ or diarrhoea<br>• 322 parents interviewed using a structured questionnaire	• The main admission diagnoses were ARI (61.8%), malaria (25.3%), diarrhoea (18.4%) and suspected sepsis (8.1%)<br>• 91% of children received treatment prior to admission, mostly antipyretics (75.6%), local herbal medicines (26.8%), and antibiotics (17.8%)—half of them without a prescription from a clinician<br>• For diarrhoea, the use of oral rehydration solution was rare (9.0%), with 49.4% of the parents presenting their children directly to hospital, 23.2% to a pharmacy/ drug shop and 19.3% to a primary health facility first
	Olamijuwon et al., 2024 (34)	• Assess the differences in treatment-seeking behaviours and antibiotic use for UTI-like symptoms among patients visiting healthcare clinics before and during the COVID-19 pandemic	• Over time, there was a significant increase in the use of antibiotics not recommended for treating UTIs in the NTG<br>• The use of ampicillin increased from 3% to 7% and azithromycin from 4% to 8% of patients between the pre-COVID-19 and COVID-19 phase
	Zimbwe et al., 2024	• Evaluate antimicrobial prescriptions and usage patterns for treating bacterial infections among outpatients<br>• Assessment based on WHO/INRUD criteria, STG/ EML and AWaRe classification<br>• 1557 prescriptions were evaluated	• Out of 1557 prescriptions - 26.1% included antimicrobials, with 1.4 antimicrobials/ prescription<br>• 90.9% of prescriptions adhered to STG/EML recommendations<br>• Out of 21 commonly prescribed antimicrobials, 42.9% of the 21 were Access antibiotics 47.6% and 9.5% Reserve
**Zanzibar**	Hadley and Beard, 2019 (70)	• Evaluate the impact of performance-based financing (PBF) to improve antibiotic prescribing based on STGs<br>• Randomised study in 2 active districts vs. controls alongside vigorous verification of prescribing	• The proportion of patients treated with an antibiotic not in accordance with STGs after PBF fell to 2%, 6% and 5% in 2014, 2015 and 2016, respectively, in active facilities compared with an increase from 25% (2013) to 31% (2014) and 22% (2015, 2016) in non-PBF facilities<br>• Overall, rigorous monitoring of prescriber habits is needed for a sustained reduction in the proportion of unnecessary antibiotic prescriptions
**Upper-Middle Income countries***			
**Gabon**	Adegbite et al., 2022 (71)	• Assess knowledge and awareness about AMR among physicians and nurses in Gabon in a referral centre as well as primary care facilities<br>• Cross-sectional study using a questionnaire adapted from previous studies<br>• 47 HCPs took part (89% response rate) – 40.4% were physicians and 59.6% were nurses	• 64% of participating HCPs believed that AMR is a problem in Gabon, with 30% believing AMR is a problem in their health facilities<br>• AMR was enhanced by 70% believing there is currently uncoordinated antimicrobial prescribing in their health facility, with 81% believing that self-medication with antimicrobials (common in Gabon) could contribute to AMR<br>• Overall, knowledge about possible causes of the spread of AMR was limited among participating HCPs – not helped by 55% of participants not receiving any recent training in antimicrobial prescribing
**Namibia**	Niaz et al., 2020 (72)	• Assess prescribing practices and drivers of compliance to National STGs including infectious diseases among public health care facilities<br>• Mixed method approach including patient exit and prescriber interviews	• There was high prescribing of antibiotics (69% of prescriptions) higher than the target of <25% although 35% acceptable<br>• Compliance with the Namibian STGs was 73% - lower than the target of >80%. This was despite 94.6% of prescribers aware of, and had access to, the STGs, 82% of prescribers reporting that it is easy to use the STGs and 32.4% stated they referred to them on a daily basis

		• 1243 prescriptions were analysed and 37 prescribers interviewed (92.5% response rate). 23 were physicians and 14 nurses	• Key drivers of compliance to the Namibian STGs were (i) programmatic, i.e. good access to up-to date objective guidelines, (ii) support systems available for continued education on their use, (iii) easy to reference
**South Africa**	Farley et al., 2018 (73)	• Assess KAP of ABR among primary care prescribers in South Africa<br>• Cross sectional survey using a self-administered questionnaire. Knowledge about antibiotic use was assessed through 7 multiple-choice questions<br>• 264 prescribers completed the survey – 98.3 % doctors with 84.8 % practised exclusively in the private sector	• Encouragingly 95.8% of those interviewed believed ABR is a major problem in South Africa.<br>• The median knowledge score was 5 out of 7, with higher scores in those under 55 years and linked to positive prescribing behaviours - including preferring narrow-spectrum antibiotics, explaining disease features to patients as alternatives to prescribing and being less likely to prescribe unnecessary antibiotics.<br>• 87.5% expressed a desire for more education on appropriate antibiotic use with 96.2% requesting data on local resistance patterns. Prescribers also showed interest in STGs provided in various formats<br>• Of concern is that 66.5% of those surveyed felt pressure from patients to prescribe antibiotics for their presenting infectious disease irrespective of the need
	Gasson et al., 2018 (74)	• Assess antibiotic prescribing in PHCs and Community Day Centres and compare with national STGs<br>• Retrospective review of antibiotic prescribing together with potential reasons for non-adherence<br>• 654 patient records were reviewed	• 68.7% of patients had been prescribed an antibiotic, with adherence to STGs at 45.1% of prescriptions<br>• Adherence differed significantly between surveyed facilities and whether the prescription was for an adult or a child<br>• Principal reasons for non-adherence to STGs included undocumented diagnoses (30.5% of prescriptions), antibiotics not required (21.6%), incorrect dosing (12.9%), incorrect duration of therapy (9.5%) and incorrect treatment (1.5%)
	Truter and Knoesen 2018 (75)	• Determine current antibiotic prescribing habits among primary care physicians<br>• Self-designed questionnaire based on the literature<br>• 16 community pharmacists participated	• 81.3% of community pharmacists felt that antibiotics were being over-prescribed by physicians, including for viral infections, exacerbated by patient pressures<br>• Amoxicillin /co-amoxiclav were the most prescribed antibiotics followed by clarithromycin, ciprofloxacin and azithromycin<br>• Patients with URTIs and sinusitis were the most common infectious diseases for which antibiotics were prescribed
	van Hecke et al., 2019 (76)	• Assess the perceptions of clinicians working in publicly funded clinics about antibiotic prescribing for acute coughs and UTIs and their experiences regarding POCTs<br>• Qualitative interviews with 23 prescribers (including 8 nurses)	• Antibiotic prescribing decisions among participating HCPs were typically influenced by their clinical assessment of patients, patient comorbidities, social factors including their access to care and perceived patient expectations<br>• However, communication difficulties between HCPs and patients often hampered efforts to explain non-antibiotic management strategies including differences between bacterial and viral infections<br>• Their experiences with currently available POCTs were largely positive, and they were optimistic about possible new POCTs to help support evidence-based prescribing and reduce unnecessary antibiotic prescribing<br>• Resources and workflow disruptions were perceived to be the principal barriers to uptake of new POCTs into routine care

	Sharma et al., 2020 (77)	• Analysis of antibiotic consumption and procurement data in in PHCs to help drive evidence-based policies and practices<br>• ‘ABC’ analysis of procured antibiotics	• Antibiotics made up approximately 7% of the total annual pharmaceutical expenditure in PHCs<br>• The most procured antimicrobials were: (1) isoniazid, (2) flucloxacillin, (3) azithromycin, (4) a combination of rifampicin, isoniazid, pyrazinamide, and ethambutol and (5) amoxicillin<br>• 55% of antibiotics were from the Access group, 2% from the Watch group and 15% from ‘Access and Watch’ categories; 28% of antimicrobials were antituberculosis medicines<br>• No antibiotics from the Reserve group were procured
	Balliram et al., 2021 (78)	• Assess KAP of doctors, pharmacists and nurses regarding antimicrobials, AMR and AMS.<br>• National online survey<br>• 1,120 doctors participated, with a response rate of 6.89%. In addition, 744 pharmacists and 659 nurses also participated (comprising the total number of HCPs participating)	• Encouragingly, 93.37% of HCPs recognized AMR as a severe global threat (96.4% for doctors), with 96.6% of doctors seeing it as a significant problem in South Africa.<br>• Only 37.70% of doctors felt ≤ 50% confidence in their knowledge of antimicrobials, AMR and AMS with overall highest knowledge scores among HCPs, e.g. 94.9% believed antibiotics are not effective against viral infections vs. 75.3% for nurses and 99.1% that common colds are caused by viruses vs. 90.2% nurses<br>• 91.61% of HCPs identified overuse of antimicrobials greatest contributor to AMR followed by patient pressure (75.26%) and non-adherence to prescribed treatments (73.26%)<br>• 44.6% of doctors had attended workshops and training on either AMS and antimicrobials; however, 80.1% expressed a need for more education and training on antimicrobial use, AMR, and AMS<br>• Doctors identified educational campaigns (91.22%), use of therapeutic guidelines (84.72%), and improved infection control measures (66.31%) as important strategies to combat AMR.
	Engler et al., 2021 (79)	• Explore factors affecting the implementation, success and sustainability of the national initiative to enhance AMS activities in public facilities in South Africa<br>• Mixed methodology involving a structured questionnaire (HCPs from 26 facilities) followed by FDGs and in-depth interviews involving 83 participants	• A number of key concerns were identified including that public healthcare facilities currently face many challenges especially among PHCs where AMS and ASP activities are not fully implemented. There are also concerns with available diagnostic facilities and routine AMR surveillance data to guide antimicrobial prescribing<br>• Suggested ways forward including continuous education for HCPs - especially for the majority of prescribers at PHC level alongside health campaigns among patients<br>• In addition, greater involvement and visibility of management to help implement and drive AMS and ASP activities
	Govender et al., 2021 (80)	• Evaluate the use and implementation of the STGs/EML by prescribers at a public tertiary institution and associated PHC facilities<br>• Mixed approach evaluating patient records and interviews using a structured questionnaire<br>• 98 nurses (98% of those approached) responded at the PHC level	• 41% of nurses had access to the latest STG/EML<br>• All nurses surveyed often/ sometimes referred to the STG/EML when managing patients<br>• 78.3% of prescriptions had a diagnosis recorded, with 76.4% of prescriptions had a diagnosis recorded in the STG/EML<br>• There was a 59.7% adherence rate for prescriptions to the STG/EML

			• 94.9% of nurses requested training on the use of STGs/EML to improve their future prescribing including antibiotics/ infectious diseases as most of them had not received formal training on its use
	Alabi et al, 2022 (81)	• Assess the appropriateness of antibiotic prescribing among GPs in the private sector<br>• Analysis of 188,141 antibiotic prescriptions for 174,889 patients<br>• Appropriateness based on ICD-10 classification and whether an antibiotic is warranted	• 92.9% of surveyed patients were prescribed one antibiotic, with 7.1 % prescribed two or more antibiotics<br>• Penicillins were the most prescribed antibiotics (40.7% of all antibiotics prescribed followed by macrolides (16.8%) and cephalosporins (15.7%) with diseases of the respiratory system (J00–J99) accounting for 46.1% of all diagnoses<br>• 8.8% of all the prescriptions were appropriate; 32.0% potentially appropriate, 45.4% inappropriate and 13.8% could not be assessed due to a lack of specific codes/ contained unlisted codes/ contained unclear descriptions<br>• 57.7% of antibiotics were prescribed at correct doses, 27.4% with wrong doses and 14.9% could not be assessed
	De Vries et al., 2022 (82)	• Evaluate the impact of a multidisciplinary audit and feedback intervention to improve future antibiotic prescribing at the primary care level<br>• Feedback meetings monthly at 13 PHCs with 10 prescriptions randomly selected for peer review<br>• Subsequently scored for adherence to seven key measures including antibiotic choice in STGs<br>• Antibiotic utilisation patterns also assessed	• Mean overall level of adherence to STGs increased from 11% at the start of the study to 53% over a 2-year period<br>• However, adherence to STGs was significantly lower in the winter and spring - concurrent with higher antibiotic prescribing and consumption potentially reflect inappropriate antibiotic prescribing for increased viral ARIs during these months<br>• 19% correct prescriptions in the first 6 months (mean - baseline) rising to a mean of 47% correct prescriptions in the last 6 months of the study (p<0.001)<br>• A reduction of 12.9 DDDs was also seen between the pre- and post-intervention periods (p=0.0084), i.e., a 19.3% decrease in antibiotic consumption over the study period
	Guma et al., 2022 (83)	• Assess current empiric prescribing rates of antibiotics among private GPs for patients with ARIs and associated key factors<br>• Semi-structured web-based questionnaire based on the literature<br>• 209 GPs took part (57% response rate)	• 55.5% of surveyed private GPs prescribed antibiotics empirically for patients with ARIs more than 70% of the time - primarily for symptom relief and the prevention of complications<br>• GPs between the ages of 35–44 years, >55 years, and in practice <15 years, were significantly more likely to prescribe antibiotics empirically. Overall, GPs with more experience and working alone were slightly less likely to prescribe antibiotics empirically<br>• Key factors significantly associated with empiric prescribing were workload/time pressures, diagnostic uncertainty and the use of a formulary
	Keuler et al., 2022 (84)	• Describe the treatment of UTIs in PHCs and determine compliance with current STGs/EML<br>• Retrospective review of medical records of patients diagnosed with UTIs<br>• 6 PHCs took part involving 401 UTIs among 383 patients	• 84.3% of UTIs occurred in females, with complicated UTIs (74.1%) more common than uncomplicated UTIs<br>• Antibiotics were prescribed in all male and 98.5% of females, all uncomplicated and 98.3% of complicated UTIs<br>• Nitrofurantoin was prescribed in the majority of UTIs (57.1%), followed by ciprofloxacin (39.7%), with nitrofurantoin more frequently prescribed in uncomplicated (75.0%) vs. complicated (50.8%) UTIs and in women (63.9%)<br>• Nitrofurantoin was appropriately selected in 75.0% of uncomplicated UTIs. In complicated cases, compliance was higher with ciprofloxacin (44.4%) vs. nitrofurantoin (25.6%)

			• Overall compliance with STGs was greater for uncomplicated (61.5%) than complicated UTIs (52.9%)<br>• Failure to comply with STGs was mostly due to inappropriate antibiotic selection for complicated UTIs and duration of therapy
	Lagarde and Blaauw, 2023 (85)	• Assess prescribing practices for young and healthy simulated patients (SP) presenting with viral bronchitis among both private and public PHCs<br>• 102 SP visits in the public sector and 99 in the private sector (as 3 SPs were detected in the private sector)<br>• 125 providers (across sectors) were also interviewed face-to-face	• Antibiotics were recommended in 72.6% of consultations, higher in the public sector (78.4%) vs. private sector (66.7%) - enhanced by perceived patient pressure to prescribe antibiotics for this condition<br>• This was despite 84% of prescribers knowing the SP case was likely a viral infection (88% in the private sector vs. 77% in the public sector), with 58% of prescribers knowing that antibiotics would not hasten recovery (40% public vs. 68% private; p=0.002)<br>• 91% of patients who received antibiotics in the public sector were prescribed amoxicillin, with overall only 5% of antibiotics prescribed being from the Watch list. This compares with 20% in the private sector (p=0.001) - despite lower awareness of AMR among public vs. private prescribers (p<0.001)<br>• 47% of public prescribers thought patients would not come back if no antibiotics were prescribed – higher in the private sector at 72% (p=0.008) - despite SPs not demanding antibiotics. However, antibiotics prescriptions fell in both sectors by 20% when HCPs explicitly told prescribers they did not want antibiotics (86)
	Wieters et al., 2024 (9)	• Assess antibiotic use by WHO AWaRe classification among patients visiting healthcare facilities in 4 African countries including South Africa<br>• Infectious diseases surveyed included acute febrile disease of unknown cause (AFDUC), GI infections and RTIs<br>• Out of 19,700 enrolled patients, 36.8% reported antibiotic use in previous the ten days for these infections	• Out of the 36.8% across the countries stating antibiotic use in the previous 10 days, 41.5% were prescribed for RTIs, 30.3% for AFDUC and 22.6% for GI infections. There were similar rates for RTIs in South Africa at 41.4% and AFDUC at 27.8%<br>• The most common antibiotic prescribed was ceftriaxone (31.7% of antibiotics prescribed – lower in South Africa at 2342% of all antibiotics taken). This was the case among patients with AFDUC - 40.2% of antibiotics prescribed<br>• Among those with RTIs, amoxicillin/clavulanic acid was generally the most commonly reported antibiotic taken – although this was ampicillin in South Africa (22.8%) – with ceftriaxone at 15.0%

*World Bank Classification; ABR = Antibacterial Resistance; AMR: Antimicrobial Resistance; AMS: Antimicrobial Stewardship; AWaRe: Access, Watch, Reserve; CAP = Community Acquired Pneumonia; EML = Essential Medicine List; HCP = Healthcare Professional; HCW = Healthcare Worker; GI = Gastro-Intestinal Infections; KAP = Knowledge, Attitudes, Practices; LMICs = Low- and Middle Income Countries; PHC = Primary Healthcare; RTIs = Respiratory Tract Infections; STGs: Standard Treatment Guidelines; UTIs = Urinary Tract Infections; URTIs = Upper Respiratory Tract Infections

Table S2: Knowledge, Attitude and Practices, antibiotics, antimicrobial resistance and antimicrobial stewardship – WHO Eastern Mediterranean Prescribers

Country	Author and Year	Objectives, Study Design and Population	Summary of the Findings
**Eastern Mediterranean Countries**			
**Eastern Med. LMIC countries**	Allwell-Brown et al., 2020 (2)	• Compiled data from national surveys in LMICs documenting antibiotic use for sick children under 5 years between 2005-17 with fever, diarrhea, or cough with fast or difficult breathing<br>• Data based on 132 Demographic and Health Surveys/ Multiple Indicator Cluster Surveys among 73 LMICs by WHO region, World Bank income classification, and symptoms<br>• Eastern Mediterranean LMICs accounted for 8.6% of surveyed children	• Antibiotic use among sick children under 5 years with these conditions increased from 36·8% in 2005 among the surveyed LMICs to 43·1% in 2017, with low-income countries having the greatest relative increase rising 34% from 29·6% in 2005 to 39·5% in 2017. However, remained the lowest of any income group throughout the study period<br>• Reported antibiotic use among sick children with diarrhoea among the surveyed LMICs remained relatively unchanged during the study period; however, among those with fever alone rose 13% from 38·8% in 2005 to 43·7% in 2017<br>• Similarly, among young children with a cough with fast or difficult breathing symptoms alone increased 20% from 41·4% in 2005 to 49·6% in 2017
**Low Income countries***			
**Palestine**	Maraqa et al., 2023 (87)	• Evaluate KAP regarding antimicrobial therapy among primary care physicians<br>• Cross-sectional study using a questionnaire based on the literature, piloted and validated<br>• 336 physicians took part (94% response rate)	• There was variable knowledge and attitudes among physicians<br>• Encouragingly, only 9.2% of participating physicians believed antibiotics can be used to treat viral infections, 82.3% strongly disagreed they would prescribe antibiotics to improve patients' trust in their skills and 72.5% strongly disagreed they would prescribe an antibiotic for an RTI when they did not have time for a clinical evaluation<br>• In addition, 84.8% strongly disagreed/ disagreed that antibiotics do not affect bacteria's resistance when prescribed for RTIs, with 95.3% strongly agreeing/ agreeing antibiotics for RTIs should only be recommended by a doctor<br>• Alongside this, 94.6% routinely advise their patients about the necessity of completing the prescribed course of antibiotics, with 96.5% stating they usually prescribe antibiotics based on available guidelines<br>• However, 29.1% believed antibiotics reduce the severity of URTIs, 60.8% that antibiotics can be prescribed for acute sinusitis and 43.4% for a 17-year-old complaining of sore throat or nonproductive cough
**Yemen**	Orubu et al., 2021 (88)	• Assessing prescribers'/physicians' and pharmacists' perceptions of antibiotic use, AMR and AMS<br>• Mixed-method study involving 27 physicians and 27 pharmacists using semi-structured questionnaires in Arabic and English (41% response rate)	• 56% of physicians and 70% of pharmacists perceived AMR to be an understudied/ underreported problem, with physicians (37%) in support of the enforcement of prescription laws to reduce purchasing of antibiotics without a prescription vs. increasing awareness of AMR among patients (22%), with pharmacists more in support of increased training (59%) and increased awareness among clients (22%) to improve future use<br>• Of concern is that 74% of physicians felt under pressure to prescribe broad spectrum antibiotics with reasons for empiric use being cost (59%) and patient

			symptoms (56%). The practice of selling antibiotics without a prescription was reported by 67% of pharmacists alongside counter prescribing (63%)<br>• Infections for which antibiotics were demanded included inflammation affecting the throat/mouth or ear (37%), as well as UTIs and RTIs (33% each)<br>• Penicillins were the most commonly prescribed and dispensed antibiotic group - of the penicillins, amoxicillin (and amoxicillin/ clavulanic acid) was the most commonly prescribed (63%) or dispensed (81%)<br>• Ciprofloxacin was the most common quinolone antibiotic prescribed (52%) or dispensed (74%)<br>• AMS activities were poor with limited use of AST impacting on awareness of resistant pathogens among HCPs in Yemen (89). This needs addressing going forward
**Low-Middle Income countries***			
**Egypt**	Kandeel et al., 2019 (90)	• Assess the effectiveness of campaigns to raise the awareness of physicians, pharmacists, and the public regarding antibiotic prescribing for ARIs. This included a 5-day training course for physicians<br>• Structured surveys with physicians also presented with three clinical scenarios that did not warrant antibiotics<br>• 1492 participants including 289 clinicians (19.4% of total)	• Overall, there was a 25% decrease in antibiotic prescribing by participating physicians post-intervention for children from 82.1% to 61.5%<br>• The self-reported % among participating physicians who never prescribe antibiotics for a common cold increased from 35.4% to 64.4%, and decreased for most times for bronchitis from 65.8% to 28.4% and sinusitis from 43.5% to 17%<br>• The mean knowledge supporting the judicious use of antibiotics improved for physicians from 3.8 ± 0.5 to 4.0 ± 0.7<br>• Attitude scores regarding reducing antibiotic prescribing for colds, limiting the use of antibiotics to preserve their effectiveness, and not giving antibiotics for nasal discharge to treat colds also improved among participating physicians
	El-Sokkary et al., 2021 (91)	• Identify knowledge and practice characteristics as well as describe the attitude of Egyptian prescribers towards antibiotic use<br>• A self-administered validated questionnaire was used<br>• 500 physicians participated from across sectors	• Encouragingly, 86.8% of participating physicians agreed on the importance of identifying resistance patterns and 80.8% expressed a need to attend antibiotic training courses<br>• Alongside this, 94.8% disagreed that antibiotics are effective against viruses, 95.2% agreed that bacteria become resistant to antibiotics, 92.2% that overuse of antibiotics makes them ineffective and 82.2% disagreed that coughs, colds and sore throats improve with antibiotics<br>• In addition, 70 to 80% of participating physicians agreed that frequent antibiotic prescribing - mainly broad-spectrum antibiotics for the same patient, inappropriate doses and patient demand all increase AMR<br>• However, 81.2% considered unindicated prescribing of antibiotics does not cause harm, only 22.6% stated they had ongoing ASPs in their workplace with only 13.4% currently having lists of antibiotics classified into the AWaRe groups. Alongside this, 69% of physicians only order cultures when there is no improvement in the clinical condition of patients<br>• A significant positive correlation was seen between knowledge and attitude and also between knowledge and practice ($p< 0.005$).

	Amin et al., 2022 (92)	• Investigate physicians' KAP towards antibiotic prescribing in children with acute URTIs<br>• Cross-sectional study using a validated questionnaire and piloted<br>• 153 physicians completed the questionnaire (84.1% response rate)	• Encouragingly, 98.7% of participating physicians believed bacteria can become resistant to antibiotics, 94.8% that physicians do play a role in decreasing ABR, 85.6% that antibiotics should not be stopped when symptoms improve and 81.1% that ABR is a considerable issue globally<br>• In addition, 77.8% of participating physicians believed that the body can usually fight mild infections without antibiotics and 72.6% that susceptibility testing helps determine the likelihood that a particular antibiotic will effectively treat certain bacterial infections<br>• However, 61.4% of participating physicians believed that broad spectrum antibiotics are preferred for treating URTIs and 50.3% that antibiotics are effective in treating fevers
**Pakistan**	Waseem et al, 2019 (93)	• Assess patients, physicians, and pharmacists' knowledge, attitudes and behaviour towards AMR using a specifically designed questionnaire<br>• 308 physicians were included in the study (53.9% response rate)	• 83.1% of participating physicians recognized that AMR is a worldwide problem<br>• However, 82.6% agreed that patients with a common cold typically demanded antibiotics from them<br>• Of concern is that only 30% of participating physicians had received any AMR related training in last three years, with 74.1% feeling the need for educational/ awareness programs to enhance their knowledge about AMR<br>• In addition, only 36.9% of participating physicians knew about the environmental route for progressing AMR
	Hashmi et al, 2021 (94)	• Document URTI-specific antibiotic prescription frequency in a public primary healthcare setting<br>• Conduct a prescription analysis of 50,705 prescriptions with appropriateness based on International Guidelines	• From 50,705 prescriptions - 4,126 (8.13%) were for URTIs. Of these, 2,880 (69.80%) prescriptions contained antibiotics.<br>• Nearly 40% of the prescriptions were diagnosed as non-specific URTIs followed by a cough (16.8%) and rhinitis (11.9%)<br>• Penicillins (including amoxicillin and amoxicillin + clavulanate) were the most prescribed antibiotic for URTIs (45.9%) of total antibiotics prescribed followed by the macrolide group (18.2%)<br>• Specialists were more competent in antibiotic prescribing based on their diagnosis followed by postgraduates and house officers
	Rakhshani et al, 2022 (95)	• Describe antibiotic dispensing/ prescribing practices and underlying factors associated with these practices among community-based HCWs including physicians<br>• Mixed-Method approach using an adapted questionnaire<br>• 177 HCWs took part including 41 physicians alongside 57 nurses, midwives, and lady health workers	• Participating physicians had a high likelihood of prescribing antibiotics for children with sore throats (75.6%), diarrhoea lasting more than one day (51.2%), fever (48.8%) and a cough (26.8%). Participating nurses and other prescribers also had a high likelihood of prescribing an antibiotic for a child with a sore throat (44.8% - 60%), diarrhoea lasting more than one day (50% - 65.5%), fever (30% - 44. %) and a cough (23.3% - 41.4%)<br>• Physicians also had a high likelihood of prescribing an antibiotic for adults with a sore throat (70.0%), burning sensation when urinating (63.4%), diarrhoea lasting more than one day (58.5%), fever (48.8%) and a cough (31.7%). Similarly, nurses and other HCWs had a high likelihood of also prescribing an antibiotic for adults with a sore throat (58.6% - 80%), burning sensation when urinating (52.2% - 90%), diarrhoea lasting more than one day (55.2% - 80%), fever (48.8% - 60%) and a cough (40% - 55.6%)

			• 100% of physicians were aware of the term AMR. However, only 65% - 80% of HCWs including nurses, midwives and lady health workers were aware of the term AMR<br>• 36.6% of participating physicians believed antibiotics could be used to treat COVID-19 with 44.6% of all respondents (including other HCWs) incorrectly believing antibiotics could be used to treat viral infections and 49.2% that antibiotics could be used to cure colds and influenza<br>• 55.0% of physicians were familiar with the AWaRe categories and guidelines. Overall, though only 32.0% of all respondents (including nurses) were familiar with the AWaRe categories and guidelines
	Mustafa et al, 2023 (96)	• Assess KAP of self-medication among HCPs including physicians during the COVID-19 pandemic<br>• Descriptive cross-sectional study using a questionnaire principally based on previous studies<br>• 1173 HCWs took part – 31.9% were physicians, 56.3% included nurses and other HCWs	• There was no significant difference in the knowledge scores regarding self-medication including concerns between physicians and pharmacists; however, both had better knowledge scores than other HCWs<br>• 66% of HCWs reported they self-medicated during the pandemic with the top three most commonly purchased medicines being antipyretics (100%), antibiotics (80.4%) and vitamins (59.9%)<br>• 42% of HCWs self-medicated to prevent catching the virus, 20.7% because they suspected they had COVID-19 symptoms and 27.9% self-medicated to treat their colds and influenza
**High-Middle Income countries***			
**Iran**	Vakili-Arki et al., 2019 (97)	• Assess the perspectives and the behavior that physicians with improving antibiotic use<br>• 330 physicians took part using a validated questionnaire	• 94% of those surveyed believed that the prescription rate of antibiotics in Iran is high and higher than the global average<br>• High rates not helped by patients' tendency to prescribe/request antibiotics (94% of physicians)<br>• Encouragingly, 84% were keen on receiving performance feedback, with 71% stating that SMS notification was their preferred medium for receiving feedback
	Sami et al., 2022 (98)	• Assess knowledge, perceptions, and attitudes regarding antibiotic prescribing among physicians at PHCs and academic hospitals<br>• Cross-sectional study using a questionnaire developed from the literature/ experts and piloted in PHCs<br>• 182 physicians completed the questionnaire (77.4% response rate)	• Encouragingly, 97.2% of participating physicians agreed or strongly agreed that AMR was a concern in Iran, 95.6% that current prescribing of antimicrobials was not appropriate and 87.5% agreeing or strongly agreeing that antibiotics are being overused in Iran putting patients at risk of AMR/ main cause of AMR (98.9%). As a result, affecting the health of themselves and their families (97.2% of participating physicians)<br>• In addition, 89% agreed or strongly agreed they can play an effective role with introducing ASPs<br>• However, 59.9% of participating physicians agreed they were under pressure from colleagues or acquaintances to prescribe broad spectrum antibiotics for patients, which needs to be addressed
	Aeenparast et al., 2024 (99)	• Determine WHO prescription indicators in outpatient services at Iran Social Security Organization healthcare facilities<br>• 150,981,752 drug items were assessed	• 37.5% of prescriptions contained at least one antibiotic<br>• GPs were at higher risk of prescribing an antibiotic than other physicians in hospital outpatient clinics

**Jordan**	Karasneh et al., 2021 (100)	• Evaluate prescribers' knowledge, attitudes and behaviour concerning antibiotics, antibiotic use and ABR in Jordan<br>• Cross-sectional study utilizing an online questionnaire that was piloted<br>• 613 prescribers took part – 409 physicians and 204 dentists	• 95.8% agreed that antibiotics were not effective against viruses and 91.2% that they were not effective against colds. In addition, 79.3% of prescribers agreed that every person treated with antibiotics is at increased risk of an ABR infection<br>• Encouragingly as well, most prescribers either agreed or strongly agreed that they knew what ABR is (87.1%), what information to give to patients about the prudent use of antibiotics and ABR (81.56%), and that they have sufficient knowledge about how to use antibiotics appropriately (85.63%)<br>• 62% of prescribers also agreed or strongly agreed they had easy access to required guidelines on managing infections, with 67.3% agreeing or strongly agreeing they had good opportunities to provide such advice to their patients
	Karasneh et al., 2021 (101)	• Explore physicians' preferred sources of information regarding antibiotics and their awareness of available information and initiatives on prudent antibiotic prescribing<br>• Cross-sectional study using a structured questionnaire developed and tested by ECDC<br>• 409 physicians took part in the study	• Encouragingly, 59.4% of participants reported they had received information to avoid unnecessary prescribing of antibiotics in the last 12 months, with this information contributing to changing their views (overall 97.1% of this subset stated they had changed their prescribing practices)<br>• Published guidelines (31.8%), workplace (25.7%), colleagues or peers (20.0%), group/ conference training (18.3%), and professional bodies (18.1%) were the main sources of information, with the influence of these sources on changing prescribers' views being 34.7%, 17.1%, 11%, 13.4%, and 7.6% respectively<br>• However, 33.7% of participating physicians reported no knowledge of any initiatives in the country on antibiotic awareness and ABR<br>• Having said this, 58.9% of physicians showed interest with receiving more information on ABR, 42.2% on how to use antibiotics, 41.3% on medical conditions for which antibiotics should be used and 35.2% on more prescribing information regarding antibiotics
	Orubu et al, 2022 (102)	• Assess the KAP among prescribers at PHC facilities of the United Nations' Relief and Works Agency in Jordan<br>• Cross-sectional study using a semi-structured questionnaire based on previously validated instrument<br>• 34 prescribers took part (59% initial response rate)	• All respondents had prescribed antibiotics in the past 6 months with 91% reporting that the most frequently used antibiotic was amoxicillin, including amoxicillin-clavulanate, for RTIs<br>• 65% reported pressure to prescribe antibiotics based on factors including patients' condition (78%); perceived patient demand (60%); fear of complications (50%) and need to provide rapid relief (47%)<br>• Overall, knowledge of AMR was good among participants; however, poor motivation for rational prescribing or behavioural change
	Ababneh et al., 2024 (103)	• Evaluation of ASPs and antibiotic prescribing patterns among physicians in ambulatory care settings<br>• Cross-sectional study using a structured questionnaire among 187 physicians	• Encouragingly, 69.5% of participating physicians used effective communication strategies to educate patients when antibiotics are necessary<br>• Their adoption of core elements of ASPs in ambulatory care settings to improve future prescribing was also positive<br>• In addition, 46.5% reported writing and displaying public commitments supporting AMS activities in ambulatory care settings<br>• However, 40.1% reported not including AMS duties in their activities and 24.6% reported a lack of self-evaluation of their antibiotic-prescribing practices

	Abu-Farha et al., 2024 (104)	• Assess the KAP of physicians regarding antibiotic deprescribing in Jordan<br>• Cross-sectional survey using a structured questionnaire that was piloted<br>• 252 physicians took part including those from hospital and ambulatory care	• Encouragingly 92.9% of participating physicians were familiar with the concept of antibiotic deprescribing, 94% stated they were knowledgeable about appropriate situations, and 96.8% stated they recognised its potential benefits<br>• 81.8% of participants reported they had received formal training in antibiotics deprescribing and 85.3% were informed about the availability of deprescribing tools<br>• However, there were challenges including insufficient time (44.4%), resistance from patients (41.3%) who believed an antibiotic was necessary and colleagues (42.1%). Despite these challenges, 46.9% of participants stated they regularly assessed antibiotic necessity in their prescribing (46.9%) and 40.5% educated patients about antibiotic-related harms

*World Bank Classification; ABR = Antibacterial Resistance; AMR: Antimicrobial Resistance; AMS = Antimicrobial Stewardship; AWaRe = Access, Watch, Reserve; FGD = Focus Group Discussions; HCP = Healthcare Professional; HCW = Healthcare Worker; PHC = Primary Healthcare; KAP: Knowledge, Attitude and Practice; URTI: Upper Respiratory Tract Infection; WHO = World Health Organization

Table S3: Knowledge, Attitude and Practices, antibiotics, antimicrobial resistance and antimicrobial stewardship – WHO South East Asian Region Prescribers

Country	Author and Year	Objectives, Study Design and Population	Summary of the Findings
**South East Asian Countries**			
**South East Asian Region LMIC countries**	Allwell-Brown et al., 2020 (2)	• Compiled data from national surveys in LMICs documenting antibiotic use for sick children under 5 years between 2005-17 with fever, diarrhea, or cough with fast or difficult breathing<br>• Data based on 132 Demographic and Health Surveys/ Multiple Indicator Cluster Surveys among 73 LMICs by WHO region, World Bank income classification, and symptoms<br>• South East Asian LMICs accounted for 27.3% of surveyed children	• Antibiotic use among sick children under 5 years with these conditions increased from 36·8% in 2005 among surveyed LMICs to 43·1% in 2017, with low-income countries having the greatest relative increase rising 34% from 29·6% in 2005 to 39·5% in 2017. However, remained the lowest of any income group throughout the study period<br>• Overall, LMICs in the South East Asian Region had the lowest reported antibiotic use in 2017 at 30·1%; however, had the greatest relative increase in antibiotic use rising from 24·5% in 2005 to 30·1% in 2017 (overall 23%)<br>• Reported antibiotic use among sick children with diarrhoea among the surveyed LMICs remained relatively unchanged during the study period; however, among those with fever alone rose 13% from 38·8% in 2005 to 43·7% in 2017. Similarly, those with a cough with fast or difficult breathing symptoms alone increased 20% from 41·4% in 2005 to 49·6% in 2017
**Low Income countries***			
**Nepal**	Shrestha et al., 2019 (105)	• Evaluate prescribing practices and the quality use of medicine in hospital outpatients<br>• Cross-sectional and quantitative study retrospectively analyzing prescriptions<br>• 770 prescriptions were reviewed	• 2448 medicines were prescribed in the 770 prescriptions or patients, with the average number of drugs per encounter at 3.2. % of encounters with antibiotic was 37.9%, with 21.3% of all medicines prescribed contained within the EML<br>• The average prescription errors per prescription were 3.4. The most common prescription errors were due to failure to mention prescriber name (87.5%), failure to mention the prescriber signature (19.2%) and failure to mention the diagnosis (39.2%)

	Aryal et al., 2020 (106)	• Analyze prescribing practices among PHCs<br>• 600 prescriptions were analyzed retrospectively based on adapted WHO/ INRUD criteria, containing 1559 medicines<br>• 120 patients along with health facility representatives were interviewed for patient-care/ health facility indicators	• Average number of medicines/ physician encounter was 2.6. Prescription encounters with antibiotics and injections were 58% and 4.2%, respectively, with amoxicillin followed by metronidazole and azithromycin the commonest antibiotics prescribed<br>• The average consultation time was 3.6 minutes and dispensing time 54.4 seconds<br>• The majority of dispensers were intern nursing students (42%), and all prescribers were medical officers<br>• 10 out of 12 PHCs surveyed had a copy of the EML. However, the availability of key drugs in PHCs was incomplete (only in 64.7% of facilities)
	Ban et al., 2020 (107)	• Characterize prescribers including community medical assistants and physicians and their role in the care and treatment of sick infants and children including the quality care provided (medicine shops/ private clinics)<br>• Face-to-face interviews in 501 shops and 82 physician-run clinics	• Quality of care problems were identified among medicine shop-based providers and physicians, including the over-use of antibiotics for treating diarrhoea and inaccurate weighing technique to determine antibiotic dose<br>• This included prescribing antibiotics for bloody diarrhoea as well as antibiotics for ARIs including amoxycillin (most prescribed) and cefixime<br>• 35% of medicine shop providers reported determining antibiotic dosing for young infants based on their age - less common among physicians (only 10%, $p < 0.001$).
	Gyanwali et al, 2020 (108)	• Assess the medicine prescribing patterns and patients' knowledge about medicine use at different levels of health care<br>• Cross sectional study involving 88 health facilities in Nepal and a structured questionnaire for patients	• Out of total drugs prescribed, 68.91% were essential drugs and 23.74 % were antibiotics<br>• Prevalence rates for antibiotic prescribing ranged from 20.18% to 26.63% of prescribed medicines depending on the facility – greatest for health posts
	Nepal et al., 2020 (109)	• Examined patterns of antibiotic prescribing in public health facilities in Nepal and explore factors influencing these practices<br>• Cross-sectional study of antibiotic prescribing in public health facilities<br>• 6,860 patient records were initially collected	• Third-generation cephalosporins (29.9%) were the most commonly prescribed class of antibiotic, followed by penicillins (24.9%) and quinolones (15.0%)<br>• Conditions for which the antibiotic prescribing was highest included pneumonia (85.5% of patients), diarrhoea (83.2%), respiratory infections (72.4%), pyrexia (66.1%), colds, sinusitis/ rhinitis (65.3%) and coughs (63.1%)<br>• Antibiotic prescribing was significantly associated with gender, age and type of facility/department
	Rijal et al., 2021 (110)	• Explore KAP of antibiotic prescriptions and uses across sectors<br>• Cross-sectional study using a previously validated WHO questionnaire and pre-tested<br>• 87 HCWs participated of which 90.2% were physicians	• 87.4% of HCWs believed that antibiotics are currently being overused all over the country, with 92% believing excessive prescribing of broad-spectrum anti-biotics will aggravate AMR. Overall, 94.3% supported a more controlled policy for antibiotic use<br>• Overall, 69–91% of HCWs surveyed had sound knowledge regarding key as-pects concerning potential mechanisms involved in AMR development
	Shrestha et al., 2021 (111)	• Ascertain the ways in which medicines were prescribed in hospital outpatients<br>• Cross-sectional descriptive study as-sessing 559 prescriptions	• 1427 medicines were prescribed in the 559 prescriptions with the average number of medicines/ prescription at 2.55±1.388<br>• Antibiotics constituted only 9.7% of total medicines prescribed – which may be due to the survey taking place in the outpatient Medical Department<br>• Only 65.8% of medicines prescribed were from the Nepal EDL

	Zheng et al, 2021 (112)	• Explore health care-seeking patterns and source of antibiotics for fevers, ARIs and diarrhoea among children<br>• Facilities included public and private clinics and pharmacies<br>• 5457, 5054 and 4861 children were included in the 2006, 2011 and 2016 surveys respectively.	• Overall, antibiotic use in rural areas increased over time with 2016 data demonstrating rural antibiotic consumption for ARIs and fever surpassing urban regions which is a concern<br>• Higher levels of maternal education were generally associated with increased antibiotic use, particularly for fever<br>• From 2006 to 2011, the percentage of U5s with only a cough receiving antibiotics increased from 7% to 12%, and 75% of children who received antibiotics for diarrhoea did not meet the required indication<br>• Overall, adherence to WHO-recommended antibiotics decreased over time, which needs addressing
**Low-Middle Income countries***			
**Bangladesh**	Nahar et al., 2020 (113)	• Explore HCPs' understanding of the use and functions of antibiotics; awareness of AMR, and perceived patient demand<br>• Qualitative study comprising in-depth interviews with 46 HCPs from both urban and rural areas including 19 graduate practitioners and auxiliary medical practitioners<br>• Interview guide based on a review of the literature combined with input from the researchers.	• Qualified doctors believed the general population sees antibiotics as medicine of power which encourages their use<br>• A few auxiliary practitioners mentioned that antibiotics give quick results – consequently gave these mainly to children in response to demands from mothers<br>• Encouragingly, unlike semi-qualified and unqualified HCPs, auxiliary practitioners had some knowledge about the different generations of antibiotics. In addition, qualified practitioners generally had a good understanding of the use, appropriate doses and actions of different antibiotics<br>• Encouragingly, all HCPs showed concern about ABR and practitioners who were informed and aware of ABR believed this was exacerbated by irregular consumption of antibiotics as well as incomplete doses being taken and their overuse<br>• However, HCPs typically shifted the responsibility of preventing/ reducing ABR onto others
	Samir et al., 2021 (114)	• Assess the prevalence and key factors associated with antibiotic use in children under five with febrile illness<br>• Secondary analysis of Bangladesh Demographic and Health Survey data<br>• 8421 children were surveyed<br>• Sources of antibiotic prescribing/ dispensing included public and private sector physicians/ unqualified providers as well as pharmacy personnel	• 33% of children had experienced fever in the preceding two weeks. Of these 9% also had symptoms of ARI and 14% also had diarrhoea<br>• 17% of surveyed children with a fever received antibiotics<br>• The highest use of antibiotics was in children under six months of age (25%)<br>• Children of parents who had completed secondary or higher education were more likely to receive antibiotics
	Bepari et al., 2023 (115)	• Identify the risk factors for developing AMR by exploring the KAP of unqualified village medical practitioner prescribers and pharmacy shopkeepers<br>• Population based cross-sectional study through structured interviews using a questionnaire based on previous studies	• 82% of unqualified local practitioners claimed they had enough sources of information regarding antibiotics<br>• However, only 29.96% of practitioners believed that antibiotics were ineffective against viral diseases reflected by 44% providing antibiotics to treat a cold or sore throat, with a high proportion also believing that antibiotics are indicated in pain and inflammation with patient demand exacerbating overuse (51%)

		• 149 pharmacy shopkeepers and 247 unqualified local medical practitioners took part	• Most correctly identified ABR refers to the bacterial loss of antibiotic sensitivity and most agreed they had a role in combating ABR with most practitioners having poor knowledge of antibiotic use and AMR<br>• Whilst 25% strongly agreed and 55% agreed that ABR is a global problem – they did not see this a problem in their regular practice (43%)<br>• Overall, unqualified local medical practitioners had higher scores regarding antibiotics and AMR than pharmacy shop keepers, with KAP scores slightly higher for unqualified village medical practitioners (median 72.38%) than for pharmacy shopkeepers (median 66.67%)
	Hanson et al., 2024 (116)	• Identify, map, and survey village doctors to understand their distribution, practice characteristics, clinical behaviours as well as access and use of technologies for clinical decision-making<br>• 371 village doctors took part (90.2% response rate)<br>• Structured survey used to collect responses	• Village doctors reported seeing a median of 84 patients per week, including a median of five pediatric diarrhea cases per week<br>• 99.7% stocked a range of antibiotics, with metronidazole (99.5% of village doctors), ciprofloxacin (98.9%) and azithromycin (98.1%) the most stocked<br>• Ciprofloxacin was the most prescribed antibiotic for diarrhea (94.1%) followed by metronidazole (87.3%)<br>• All village doctors had access to phones, with 82.7% also having an internet connection with their phones, with 78.4% using their smartphones to support clinical decision-making
	Mahmudul Islam et al, 2024 (117)	• Evaluate the appropriateness of antibiotic doses among paediatric patients among in-patients (33.6% of the sample) as well among out-patents and emergency patients (66.4%)<br>• Cross-sectional study analysing prescription data	• High prevalence of inappropriate antibiotic dosing among paediatric patents (61.5%) including overdosing (36.3%), underdosing (20.4%) and parenteral antibiotics (36%). The majority (230 out<br>• Rates of inappropriate antibiotic dosing varied by location - 33.8% for inpatients, 86.7% for outpatients, and 50% for emergency paediatric patients.
**India**	Nair et al., 2019 (118)	• Assess the KAP of formal prescribers (physicians - 96), nurses (96), informal prescribers (96) and pharmacy shopkeepers (96) with respect to antibiotic use<br>• Cross-sectional study using a validated questionnaire	• 95.9% of informal health providers, 98.9% of nurses, and 94.8% of pharmacy shopkeepers claimed their knowledge of antibiotics was important to them in their role even though none of these groups are legally permitted to prescribe/ dispense antibiotics independently<br>• Appreciable differences between the groups with 76.1% of physicians either strongly disagreeing or disagreeing with the statement that antibiotics were useful for viral infections. This compares to over 60% of nurses, over 70% of informal providers and over 80% of pharmacy shopkeepers who thought antibiotics are useful for viral infections<br>• Having said this, over 88% of physicians reported prescribing antibiotics for viral infections, e.g. common colds or sore throat, in reality enhanced by patient demands and as a precaution against secondary infections – similarly 85% of informal providers for some of their patients<br>• Typically, all 4 groups disagreed/ strongly disagreed with stopping antibiotics early; however, all 4 groups frequently disbursed shorter, 3-day courses
	Nair et al., 2019 (119)	• Assessing the key drivers of antibiotic use among both formal prescribers (6), nurses (5), informal prescribers (5), pharmacy shopkeepers (5) and patients (7)	• Many physicians are happy to prescribe antibiotics without any clinical indication – often seeing them as a vital part of care delivery - as a result of variable follow up/ questioning, lack of testing facilities, risk of secondary infections and often unhygienic living conditions

		• Qualitative study involving in-depth interviews using an adapted validated questionnaire	• Prescribing/ dispensing exacerbated by patient demand for antibiotics – with antibiotics seen as 'quick cures' – and lack of time with physicians typically seeing 80 – 100 patients/ day in PHCs<br>• Generally limited knowledge regarding AMR with some pharmacy shopkeepers believing AMR caused by bad weather with antibiotics often combined for a range of illnesses
	Sulis et al. 2020 (54)	• Estimate the proportion of Standardised Patient (SP) – provider interactions resulting in inappropriate antibiotic prescribing in primary care<br>• Secondary analysis of published SP studies<br>• AWaRe classification used to categorise antibiotic utilisation	• 2392 of 4798 (49.9%) SP–provider interactions resulted in at least one antibiotic prescription, with a peak of 59.4% among for children with diarrhoea<br>• 2768 antibiotics were given to 2392 patients, with the most prescribed antibiotics being azithromycin (13.8%), co-amoxiclav (12.4%), amoxicillin (9.5%), levofloxacin (7.3%) and cefixime (7.2%)<br>• Watch antibiotics were disproportionately prescribed (47.6%), higher in urban areas (54.9%) reflecting greater prescribing of quinolones, cephalosporins and macrolides
	Gautham et al., 2021 (120)	• Analyze the multiple drivers of antibiotic provision by informal providers (IPs) to inform future community stewardship interventions<br>• Surveyed 291 Ips with a structured questionnaire alongside in-depth interviews with 30 IPs and 17 key informants including pharmaceutical sales representatives, wholesalers/retailers and public doctors alongside 8 FGDs with community members	• There was a mosaic or bricolage of informal practices conducted by IPs, qualified doctors and industry stakeholders that sustained private enterprise and supplemented the current weak public health care sector<br>• IPs' intrinsic drivers included misconceptions about the therapeutic necessity of antibiotics, i.e. without antibiotics the patients will not be cured<br>• There were also direct and indirect economic benefits from dispensing medicines, although antibiotics were not the most profitable category. In addition, sub-optimal courses of antibiotics were frequently dispensed due to patients' financial constraints<br>• Private doctors were a key source of IPs' learning, with IPs constituting a substantial market for local and global pharmaceutical companies
	Chatterjee et al., 2022 (121)	• Address the lack of systematic data regarding the KAP of Indian physicians relating to antimicrobial use and AMR<br>• A validated questionnaire was used for the study<br>• 506 clinicians took part including those involved with treating patients in outpatients	• Encouragingly, 86.56% of participating clinicians believed that AMR is a major challenge in clinical settings, with 93.08% considering their AMR knowledge is beneficial in their practice and 94.26% agreeing that antibiotics are prescribed inappropriately<br>• Approximately 70% of participating clinicians felt that AMR was primarily driven by incorrect antibiotic dosing, duration and by excessive antimicrobial use both in community and hospital settings, with 64.3% believing that a major contributor towards the development of AMR was patients' demands for antibiotics<br>• However, 17.69% believed that no damage can be caused by prescribing antibiotics to patients even when they do not require them<br>• Overall, despite satisfactory knowledge among physicians regarding appropriate use of antibiotics and AMR, gaps in attitude and prescribing practices demand the introduction of specific interventions to improve prescribing
	Gautham et al., 2022 (122)	• Assess the extent of antibiotics stocked, their sales volumes, prices and mark-ups by informal providers (IPs)	• Of the 196 IPs that stocked antibiotics, 85% stocked tablets, 74% stocked syrups/suspensions/drops and 18% stocked injections<br>• Across all the IPs, 42 antibiotic active ingredients were stocked - 278 branded generics from 74 manufacturers. The top five antibiotics stocked were

		• Conducted a cross-sectional survey among 291 IPs using a structured questionnaire	amoxicillin/ clavulanate (52% of IPs), cefixime (39%), amoxicillin (33%), azithromycin (25%) and ciprofloxacin (21%)<br>• 71% of the IPs stocked an Access antibiotic and 84% stocked a Watch antibiotic, with median prices in line with government ceiling prices although substantial variation between the lowest and highest priced brands<br>• The most affordable among the top 5 tablets were ciprofloxacin, azithromycin, cefixime and amoxicillin (US$ 0.8 - 1.9/ course), and the most affordable among the syrups/suspensions/drops were azithromycin and ofloxacin (US$ 1.7 - 4.5/ course) - mostly Watch antibiotics
	Mittal et al., 2023 (123)	• Evaluating physicians' attitude, knowledge and prescribing behaviour of antibiotics which would help and guide to design strategies for rational antibiotic use<br>• Questionnaire based cross sectional online survey (questionnaire pre-tested)<br>• 544 physicians completed the survey (76.2% response rate)	• Encouragingly, 98.2% of participating physicians believed that indiscriminate use of antibiotic exacerbates AMR – with 95.8% believing use of broad-spectrum agents also contributes to AMR<br>• 85.7% knew the importance of hospital antibiograms to help with empiric antibiotic therapy; however, 60% were unclear about the difference in antibiogram data from different hospitals<br>• 57.5% of participating physicians believed that the challenge of ABR can be addressed by active combined role of all key stakeholders, with 87.8% believing that to combat AMR regular surveillance is essential with guidelines also helping<br>• Encouragingly 89.7% of physicians were against prescribing antibiotics based on patients' demands<br>• However, over 50% were not familiar with the WHO AWaRe classification of antibiotics and only 31.5% knew which antibiotics were effective against anaerobes. This needs to be addressed with 76.8% believing that their antibiotic prescribing habits impacts on the development of ABR in their region<br>• 31.6% opted for delayed or back up antibiotic prescribing practices to reduce AMR; however, 27.6% were unaware of this concept and 40.8% showed disagreement regarding the use of delayed antibiotics.
	Debnath et al, 2024 (124)	• Assessing among lower tier hospitals inc. primary hospitals antibiotic/ multiple antibiotic prescription rates for common infections and assessing existing strengths of health systems for optimizing antibiotic prescription<br>• Cross sectional convergent parallel mix-method study in 8 lower tier hospitals including quantitative analysis	• 600 OPD prescriptions of UTIs, ARIs, acute fever and acute diarrhoea were evaluated.<br>• Antibiotic prescription rates were 63.8% in OPDs of primary tier hospitals and 60.8% in secondary tier hospitals, with multiple prescription rates higher in secondary tier hospital (23.8%)<br>• Activities including prescription audit and monitoring of prescribing were identified as key facilitators for future implementation of ASPs in these hospitals
**Indonesia**	Alkaff et al., 2019 (125)	• Identify the prevalence of antibiotic use for common childhood illnesses and investigate factors associated with its use in children under 5 years old<br>• Cross-sectional study using a structured questionnaire	• 203 children had clinical symptoms, and the most common symptom was fever and respiratory symptoms<br>• In total, 49.3% received antibiotics, and 66% of them were prescribed by private health professionals. Only 2% of children received antibiotics without a prescription

		• Data from caregivers of 334 children of 302 caregivers (98.1% response rate)	• The highest proportion (62.2%) of antibiotic use in children who had a combination of respiratory symptoms, diarrhea, and fever. A lower proportion (30%) for respiratory symptoms only and diarrhea only (25.0%)<br>• The most common antibiotic used among children was amoxicillin.
	Christanti et al., 2021 (126)	• Identify antibiotic knowledge and attitudes among cadres working in Health Offices including those actively involved in local healthcare activities<br>• Cross-sectional study using a paper-based questionnaire based on the literature<br>• 112 cadres took part (100% response rate)	• Encouragingly 86.6% of respondents strongly agreed/ agreed that antibiotics must be used as directed and courses must be finished<br>• Of concern is that only 37.5% strongly disagreed/disagreed with the statement 'When I get a sore throat, I prefer to take antibiotics'; only 41.1% strongly disagreed/disagreed taking antibiotics when having a cough for more than a week and 47.3% "when I get influenza, I would take antibiotics to help me recover sooner"<br>• Alongside this, only 47.3% answered correctly that ABR means that antibiotics can no longer kill bacteria
	Wardani et al., 2021 (127)	• Analyze antibiotic use and factors affecting the quality in PHCs<br>• Patients visiting 4 PHCs between March and April 2018	• No significant difference in antibiotic use between urban and rural PHCs for patients with ARIs, non-pneumonia and nonspecific diarrhea<br>• Most prescribed antibiotics were amoxycillin and cephadroxil<br>• Physicians with more than 7 years of service were higher prescribers of antibiotics<br>• Overall, no significant difference in antibiotic use between physicians who had been trained on rational drug use and those not in urban PHCs; however, a significant difference in rural PHCs
**Myanmar**	Althaus et al. 2019 (128)	• Assess whether C-reactive protein (CRP) testing at point of care could rationalise antibiotic prescribing for febrile patients in primary care<br>• Multicentre open-labelled study among 6 PHCs in Thailand, 3 PHCs and one outpatient department in Myanmar<br>• Overall 2410 patients were recruited	• 39% of patients in the control group were prescribed an antibiotic by day 5 compared with 36% in CRP group A (CRP 20 mg/L) and 34% in CRP group B (CRP 40 mg/L).<br>• There was a significant difference between group B and the control but not group A<br>• Patients with high CRP levels were more likely to be prescribed an antibiotic with though no evidence of any difference in clinical outcomes
	Swe et al., 2021 (129)	• Quantify prescriber variability in antibiotic prescribing to patients with acute fever in primary care clinics<br>• Secondary analysis of prescribing data from 1090 patient consultations	• When two prescribers are randomly selected, in half of these pairs the odds of prescription will be greater than 1.82-fold higher in one prescriber than the other<br>• Overall, the top 25% of prescribers will prescribe antibiotics to over 41% of patients in their clinics whilst the bottom 25% will prescribe antibiotics to less than 23% of patients<br>• Inter-prescriber variation in antibiotic prescribing remained after adjustment for patient characteristics and CRP information ($P = 0.001$).
**Sri Lanka**	Shu et al., 2022 (130)	• Identify knowledge concerning antibiotic use and practices related to antibiotic prescribing among Sri Lankan doctors<br>• Cross-sectional study using a validated, pretested questionnaire<br>• 262 physicians took part in the study	• Encouragingly, 99.6% of participating physicians stated that antibiotics should not be prescribed to any patient with fever and 85.1% that antibiotics can prevent development of bacterial infections. However, only 60.1% stated that antibiotics are used to treat bacterial infections only<br>• In addition, 99.6% believed ABR is a world-wide problem and 98.5% a problem in Sri Lanka and only 2.9% of surveyed physicians believed antibiotics can be used to treat viral infections.

			• Alongside this, 98.5% believed AMR was caused by the over use of antibiotics with 66.0% believing AMR is exacerbated by the over use of broad-spectrum antibiotics and 91.6% that AMR is exacerbated by patients' poor adherence to antibiotic dosage regimens. 62.2% of surveyed physicians also stated they think about AMR when prescribing antibiotics<br>• However, 31.9% stated they do find it hard to select the correct antibiotic and 44.3% that patients will feel their illnesses are not taken seriously if an antibiotic is not prescribed<br>• Alongside this, 52.3% of surveyed physicians almost always/ often/ sometimes felt under pressure to prescribe antibiotics if patients expect these – with 98.5% stating they would like more education on antibiotic use, AMR and AMS activities
**Timor-Leste**	Harris et al., 2021 (131)	• Estimate antibiotic consumption in the country stratifying into resistance class and adherence to EML and AWaRe guidelines<br>• Retrospective review of distribution data from Timor-Leste central medical store	• Nationally antibiotic usage was estimated at 11.1 DDD/1000 inhabitants/day-comparable to other LMICs<br>• Oral amoxicillin accounted for 38 to 68% of total antibacterial consumption in the 13 different municipalities<br>• Restricted antimicrobials accounted for only 2.2% of total antimicrobial consumption – higher in 4 of the 13 municipalities
**Low-Middle Income countries***			
**Thailand**	Althaus et al. 2019 (128)	• Assess whether C-reactive protein (CRP) testing at point of care could rationalise antibiotic prescribing for febrile patients in primary care<br>• Multicentre open-labelled study among 6 PHCs in Thailand, 3 PHCs and one outpatient department in Myanmar<br>• Overall 2410 patients were recruited	• 39% of patients in the control group were prescribed an antibiotic by day 5 compared with 36% in CRP group A (CRP 20 mg/L) and 34% in CRP group B (CRP 40 mg/L).<br>• There was a significant difference between group B and the control but not group A<br>• Patients with high CRP levels were more likely to be prescribed an antibiotic with though no evidence of any difference in clinical outcomes

*World Bank Classification; ABR = Antibacterial Resistance; AMR: Antimicrobial Resistance; AWaRe = Access, Watch, Reserve; CRP = C-Reactive protein; FGD = Focus Group Discussions; HCP = Healthcare Professional; HCW = Healthcare Worker; PHC = Primary Healthcare; KAP: Knowledge, Attitude and Practice; URTI: Upper Respiratory Tract Infection

Table S4: Knowledge, Attitude and Practices, antibiotics, antimicrobial resistance and antimicrobial stewardship – WHO Western Pacific Region Prescribers

Country	Author and Year	Objectives, Study Design and Population	Summary of the Findings
**Western Pacific Region**			
**Western Pacific LMICs**	Allwell-Brown et al., 2020 (2)	• Compiled data from national surveys in LMICs documenting antibiotic use for sick children under 5 years between 2005-17 with fever, diarrhea, or cough with fast or difficult breathing<br>• Data based on 132 Demographic and Health Surveys/ Multiple Indicator Cluster Surveys among 73 LMICs by WHO region, World Bank income classification, and symptoms<br>• Western Pacific LMICs accounted for 2.9% of surveyed children	• Antibiotic use among sick children under 5 years with these conditions increased from 36·8% in 2005 among surveyed LMICs to 43·1% in 2017, with low-income countries having the greatest relative increase rising 34% from 29·6% in 2005 to 39·5% in 2017. However, remained the lowest of any income group throughout the study period<br>• Overall, LMICs in the Western Pacific Region had the highest reported antibiotic use in 2017 at 61.7%<br>• Reported antibiotic use among sick children with diarrhoea among the surveyed LMICs remained relatively unchanged during the study period; however, among those with fever alone rose 13% from 38·8% in 2005 to 43·7% in 2017. Similarly, those with a cough with fast or difficult breathing symptoms alone increased 20% from 41·4% in 2005 to 49·6% in 2017
**Low Income countries***			
**Cambodia**	Ardillon et al, 2023 (20)	• Characterize inappropriate antibiotic prescribing among young children visiting outpatients and to identify its determinants in 3 LMICs including Cambodia<br>• Data included from the prospective BIRDY cohort study including children at birth and followed up for 3 to 24 months<br>• 572 children were enrolled in Cambodia, 1,816 in Madagascar and 331 in Senegal	• Out of 11,762 outpatient consultations, 29.3% led to antibiotic prescriptions across the 3 countries<br>• 76.5% of consultations resulting in antibiotic prescriptions were determined not to require antibiotics - ranging from 71.5% in Madagascar to 83.3% in Cambodia. Among the 10,416 consultations (88.6%) determined not to require antibiotics, 25.3% resulted in antibiotics with lower rates in Madagascar (15.6%) vs. Cambodia (57.0%) or Senegal (57.2%)<br>• In Cambodia and Madagascar, the diagnoses accounting for the greatest absolute share of inappropriate prescribing were rhinopharyngitis (59.0% in Cambodia) and gastroenteritis without blood in the stool (61.6%)<br>• The most frequently prescribed inappropriate antibiotic was amoxicillin in Cambodia and Madagascar (42.1% and 29.2%, respectively)<br>• Factors associated with an increased risk of inappropriate antibiotics were patient's age greater than 3 months and rural vs. urban setting
**Lao PDR**	Keohavong et al., 2019 (132)	• Assess patterns of antibiotic prescriptions for under-fives with common cold or URTIs in paediatric outpatients<br>• 54 HCPs were interviewed using a structured questionnaire and 576 medical records examined	• Among the HCPs 85.2% stated they had seen the STG, with 77.8% stating they adhered to this guideline<br>• In addition, 90.7% stated they knew about AMR, with 18.5% participating I AMR activities<br>• Despite this, appreciable prescribing of antibiotics - 68.8% of children in one facility and 70.9% in another) – almost exclusively for URTIs (96.4% overall), and not for common colds (4.9%)<br>• Recommended antibiotics were prescribed in 81.5% of patients; mainly, beta-lactam antibiotics were prescribed (87.2% of prescribed antibiotics)

	Sychareun et al., 2021 (133)	• Assess KAP of HCPs including physicians regarding antibiotic use and ABR related to pregnancy, childbirth and infants<br>• Mixed-methods study using a pre-tested structured questionnaire<br>• 217 HCPs took part including 46 physicians/ assistant physicians and 110 midwives/ nurses	• 94% of participating HCPs had heard of ABR, 91% that extensive use of anti-biotics increases the risk of ABR, 89% that unnecessary use of antibiotics can make them ineffective and 76% that antibiotic courses should not be inter-rupted even when symptoms of sick patients are improving.<br>• Overall, 80% agreed that ABR is an appreciable problem in their practice with 67% believing ABR is a problem in Laos. In addition, 100% also correctly classified ampicillin, amoxicillin and penicillin as antibiotics<br>• However, only 68% stated cortisone is not an antibiotic. In addition, 39% would prescribe an antibiotic to an adult with a runny nose, cough or fever to hasten recovery – reflected by antibiotics being prescribed/dispensed for fever (69%), sore throats (63%) and coughs (51%)
**Papua New Guinea**	Zamunu et al., 2018 (134)	• Assess the extent of antibiotic use in children with a common cold or URTI in out-patients<br>• Cross-sectional descriptive study involving 108 children diagnosed with a common cold	• 82.4% of children with a common cold were prescribed antibiotics<br>• Children with fever on examination, those older than 12 months and those whose symptoms lasted ≥7 days were more likely to be prescribed antibiotics<br>• This was despite 95% of HCWs knowing that viruses were the cause of the common cold; however, 30% thought that antibiotics were needed for treatment.
**Philippines**	Berdida et al., 2022 (135)	• Assess KAP and the associated predictive variables regarding antibiotic use and ABR among key stakeholder groups<br>• Cross-sectional study using a questionnaire building on the Eurobarometer instrument<br>• 3767 people participated (90% response rate) including 1020 HCWs	• Encouragingly, 72.2% of participating males believed the body can usually fight mild infections (68.7% for females) – 70.0% for both HCWs and non-HCWs. 80.6% of males also believed bacteria can become resistant to antibiotics (77.9% for females) – 84.7% for HCWs vs. 76.7% for non HCWs<br>• 70.4% of males (62.1% for females) also believed the risks of ABR are higher the more antibiotics are consumed – 77.6% for HCWs and 60.5% for non HCWs<br>• In addition, only 0.2% of males believed physicians prescribe antibiotics when patients expect them (1.6% for females)<br>• However, less than half of the participants had ample knowledge that antibiotics are ineffective against a cold and 37.4% believed antibiotics aid recovery from colds (31.9% for females) – 36.2% for HCWs and 33.1% for non-HCWs<br>• Overall, age, educational attainment, profession, antibiotic use within the last 12 months and having household members taking antibiotics were significant predictors of the level of knowledge on ABR
**Vietnam**	Di et al, 2022 (136)	• Determine physician's perspective on key factors affecting ABR in Vietnam<br>• Qualitative approach using FGDs and a structured interview guide<br>• 6 FGDs were conducted with 34 hospital physicians including a limited number from outpatients	• Encouragingly, 88% of participating physicians agreed that they and their colleagues had sufficient information on how ABR develops in their hospitals, with most participants mentioned that the practice guidelines issued by the Vietnam Ministry of Health in 2015 are a common source of information regarding antibiotic prescribing<br>• Physicians' experience was identified by almost half of the physicians as the most relevant factor affecting the prescription of antibiotics in outpatient care<br>• Common causes of knowledge deficiency among physicians contributing to inappropriate antibiotic prescribing/ ABR include a lack of self-updates as well as a lack of regular and advanced training

			• Most of surveyed physicians were aware of how policies on the use of antibiotics can help reduce ABR under well-controlled ASPs
	Nguyen et al., 2023 (137)	• Investigate antibiotic prescribing patterns and patient-specific factors to treat patients with ARIs in rural primary care settings<br>• Review 193010 outpatient visits for ARIs<br>• Statistical analysis of prescribing data	• 97.0% of visits to outpatients resulted in an antibiotic prescription - 92.5% were ‘Access’ antibiotics and 5.6% were ‘Watch’ antibiotics<br>• Younger aged patients with ARIs, especially those under 5-years, more frequently prescribed ‘Watch’ antibiotics<br>• Watch antibiotics were also more likely to be prescribed in follow-up rather than first visits
	Pinto Jimenez et al., 2023 (48)	• Assess the awareness of ABR among HCPs from six LMICs in both human and animal health including Vietnam<br>• Questionnaire based study using a pre-tested questionnaire among 1091 participants including 726 human HCPs of which 226 were from Vietnam (32.3% physicians and 40.3% nurses/ physician assistants)	• Typically lower awareness scores regarding ABR compared with HCPs from the other LMICs despite 38.5% stating they had training regarding AMR or AMS vs. only 25.4% for Tanzania<br>• 68.1% of human HCPs from Vietnam stated their medical decisions on prescribing antibiotics were driven by fear/ worse health outcomes (versus for instance 63.5% from Tanzania and 75.3% of HCPs from Nigeria)<br>• Prescribing decisions influenced by:<br>○ Lack of availability of local resistance data across countries (two thirds of HCPs taking part across countries – 44.2% in Vietnam)<br>○ Exposure to company advertising (two thirds of HCPs – 77.4% in Vietnam)<br>• Promotional activities from medical representatives (87.2% - Sometimes/ always)
	Thi et al., 2024 (138)	• Evaluate antibiotic prescribing in children treated for CAP in outpatients<br>• Cross-sectional descriptive retrospective study involving 3,555 children with CAP	• All antibiotic prescriptions were considered empiric as no documented bacterial or viral tests<br>• 66% of children with CAP received single antibiotic therapy with 34% receiving combinations<br>• Amoxicillin/clavulanic acid (50.77%) and azithromycin (30.74%) were the most commonly prescribed antibiotics for children in both single and combination therapies
	Vu Minh et al., 2024 (139)	• Gain a greater understandingof factors influencing prescribers’ and dispensers’ behavior regarding antibiotics and AMS<br>• Qualitative study involving in-depth interviews and FDGs with38 doctors/ doctor associates and pharmacists using a structured questionnaire/ discussion guide	• 16 themes were identified directly mapping onto 7 theoretical domains including knowledge, skills, social influences and social/professional role<br>• Factors driving unnecessary prescribing/ dispensing of antibiotics included a low awareness of AMR, diagnostic uncertainty, inadequate medication supplies, insufficient financing, patients’ perception of health insurance medication as an entitlement and maintaining doctor-patient relationships<br>• Potential factors facilitating AMS activities include time availability for in-person patient consultations, training/ experience in health communication with patients and willingness to take effective action against AMR
**Upper-Middle Income countries***			
**China**	Chang et al., 2019 (140)	• Document clinical use, overuse and misuse of antibiotics in rural primary care institutions and evaluate any association between antibiotics use and physician/ patient characteristics<br>• Review of medical records from 16 primary care hospitals	• Uncomplicated RTIs were the most common infections accounting for 58.6% of all prescriptions, with penicillins (51.5%) cephalosporins and macrolides (14% each) the most common antibiotic dispensed<br>• Out of 57,009 patient visits, only 8.7% of the antibiotic prescriptions were deemed appropriate with unnecessary antibiotics use was found in 77.3% of patient visits

		• 74,648 antibiotics prescriptions were retrieved.	• Antibiotics misuse was significantly more likely among newly employed physicians with lower levels of professional education<br>• Adult patients and those with public insurance had a higher risk of being prescribed antibiotics unnecessarily
	Ding et al., 2019 (141)	• Commentary paper assessing the extent and rationale behind inappropriate prescribing of antibiotics in PHCs/ Outpatient clinics in China despite ongoing reforms<br>• This is important with AMR rates higher in China than Western countries and continuing to grow	• Reasons for continuing inappropriate prescribing include insufficient training, poor clinical behaviour, patients' beliefs and expectations<br>• In addition, many Chinese people receive outpatient care for their infections, with hospitals deriving a considerable amount of their revenue from drug sales with physicians also receiving bonuses tied to the revenue they bring in - this is despite China's zero-profit drug policy<br>• Busy physicians may also be more likely to write prescriptions than educate patients on appropriate use of antibiotics following demand<br>• The use of leftover antibiotics at home, and antibiotic sales without prescriptions also add to improper use of antibiotics in China
	Liu et al, 2019 (142)	• Measure the knowledge and attitudes of primary care physicians towards antibiotic prescribing and their impact<br>• Questionnaire survey, piloted before the main study<br>• 625 physicians took part in the study	• Respondents scored on average 55.0% of correct answers about antibiotic prescriptions, with respondents reporting they would prescribe antibiotics to about 40.0% of patients with URTIs<br>• In addition, a high level of knowledge was associated with a more positive attitude and behavioural intention for containing antibiotic prescriptions<br>• A high level of concern regarding ABR was linked with low intentions to prescribe antibiotics ($p < 0.001$) and high intentions to reduce antibiotic prescriptions ($p < 0.001$).<br>• Incorrect answers were most likely to appear in antibiotic prescriptions for URTIs (94.24%), dosage adjustment for patients with renal failure (89.76%), and effective antibiotic treatment for methicillin-resistant Staphylococcus aureus (70.88%)<br>• Of concern is that only 76.32% of respondents stated they had received train-ing in relation to antibiotics in the last year prior to the survey
	Wushouer et al., 2020 (143)	• Explore key factors, especially knowledge, influence the use and prescriptions of antibiotics among physicians<br>• Mixed method including a structured questionnaire among 334 physicians (92.8% response rate) and analysis of out-patient prescriptions (385,529)	• The mean score of the questionnaire was a passable 62.8<br>• The average percentage of encounters with antibiotics prescribed was 26.8% - combination 2.8%<br>• Physicians who scored in [80–100] group had lower antibiotic prescribing rates than physicians in [60–80) group, and physicians who scored in [0–60) group had the highest antibiotic prescribing rates<br>• The percentage of encounters with antibiotics prescribed in the respiratory medicine department was lower than those in paediatric departments ($P<.01$)
	Xu et al., 2020 (144)	• Investigate the impact of parental self-medication with antibiotics for their children before a consultation with physicians and subsequent antibiotic prescribing behaviour<br>• Cross-sectional survey using a structured questionnaire	• One-third of the studied children had parental self-medication with antibiotics before a consultation<br>• 83.9% of them were subsequently prescribed antibiotics by physicians for their infectious disease - with children with parental self-medication more likely to be prescribed antibiotics, including IV antibiotics, and both oral and IV antibiotics, than children without prior parental self-medication<br>• Parents with self-medication behaviours were more likely to request antibiot-ics, including IV antibiotics, and be fulfilled by doctors than other parents

		• Data from 1275 parents who had self-medicated their children were analysed	
	Xu et al., 2021 (145)	• Assess KAP regarding the prescribing of antimicrobials among physicians<br>• Cross-sectional study with physicians from health institutions/ county hospitals using a structured questionnaire<br>• 580 physicians completed the questionnaire (96.67% response rate)	• 32.1% of physicians would prescribe antibiotics to patients with URTIs<br>• Multivariate analysis indicated that young general practitioners with less training were more likely to prescribe antibiotics ($p < 0.05$)<br>• Older physicians with more training were more willing to provide patients with correct knowledge regarding antimicrobials and, as a result, less likely to prescribe antimicrobials for URTIs<br>• Overall, the level of physician's knowledge, attitude, and prescribing practice are related ($p<0.05$)
	Wang et al., 2022 (146)	• Explore patterns of antibiotic prescribing in children's outpatient clinics in primary care institutions<br>• Electronic prescription data from 75 primary care institutions<br>• Appropriateness based on published guidelines	• 158,267 antibiotic prescriptions were retrieved with acute URTIs the most common disease, accounting for 74.9% of all prescriptions<br>• Penicillins (63.7%) were the most prescribed antibiotics followed by cephalosporins (18.8%)<br>• Only 18.3% of antibiotic prescriptions were seen as appropriate with a high percentage of unnecessary use (76.9%) alongside incorrect spectrum of antibiotics chosen (2.4%) and combined use of antibiotics (2.4%)<br>• Physicians with lower professional titles and more than 40 years of work duration were relatively more likely to prescribe antibiotics inappropriately
	Xu et al., 2022 (147)	• Ascertain possible differences in antibiotics use following symptomatic RTIs between 2016 and 2021 and the introduction of a number of Government initiatives to enhance appropriate prescribing<br>• Data on antibiotics prescribed, diagnosis, and socio-demographic, were obtained through non-participative observation and a structured exit survey	• Encouragingly, the rate of antibiotics prescribing for patients with RTIs decreased from 89.6% in 2016 to 69.1% in 2021, and the proportion of prescriptions for two or more classes of antibiotics also decreased from 35.9% in 2016 and 11.0% in 2021<br>• There was also a statistically significant decrease in the number of days from symptom onset to clinic visits between the year groups<br>• Cephalosporins and penicillins were the most prescribed antibiotics in both study periods, with cephalosporin usage remaining unchanged at 33.7% and 32.2% of total antibiotics prescribed respectively<br>• There was a decrease in the rate of antibiotics administered intravenously from 49.3% in 2016 to 31.7% in 2021, which is also encouraging
	Xue et al., 2022 (148)	• Determine how primary care physicians weigh intervenable patient attributes in their decisions of antibiotic prescribing for patients with URTIs<br>• Discrete choice experiment among 386 primary care physicians	• Intervention able patient attributes considered by primary care physicians in their antibiotic prescribing decisions for patients with URTIs included those with difficulties scheduling follow-up appointments and those without a clear rationale to refuse antibiotics<br>• Overall, patient requests for antibiotics had a greater impact on prescribing versus ease of follow-up appointments - with the most profound impact among patients aged between 60 and 75 years
	Fu et al., 2023 (149)	• Assess the appropriateness of outpatient antibiotic prescriptions at primary healthcare facilities (PHFs)<br>• Outpatient-visit prescriptions from 269 Chinese PHFs were assess with antibiotic use were classified into appropriate, potentially appropriate, and inappropriate	• Out of 209,662 antibiotic prescriptions assessed, 70.5% were inappropriate (66.8% of the total costs for antibiotics)<br>• URTIs, acute bronchitis, and non-infectious gastroenteritis were responsible for 68.9% of inappropriately prescribed antibiotic<br>• High inappropriate antibiotic prescribing rates were observed among children aged 0 - 5 years (78.5%) and patients living in economically undeveloped areas (77.5%)

			• 82.2% of prescriptions were for broad-spectrum antibiotics, with second-generation cephalosporins (15.1%) and third-generation cephalosporins (14.6%) the most commonly prescribed subgroups
	He et al. 2023 (150)	• Assess the characteristics and patterns of the antibiotic prescriptions for children with ARIs in rural primary PHCs<br>• 46,699 first prescriptions for ARIs among children aged 0–18 years were screened from 444,979 outpatient prescriptions	• 83.0% of children received at least one antibiotic as part of their prescription<br>• 40.76% received parenteral antibiotics, 56.1% broad-spectrum antibiotics and 31.6% two or more kinds of antibiotics<br>• Multivariable analysis showed that children aged ≤5 years were less likely to be prescribed antibiotics vs. older children and those with health insurance were more likely to be prescribed with antibiotics vs. others
	Li et al., 2023 (151)	• Explore prescription patterns and usage trends of antibiotics among primary care institutions especially following recent reforms<br>• Retrospective analysis of antibiotic prescriptions among 25 primary care institutions	• 941,924 prescriptions were included, revealing a decreasing trend in both the number and rate of inappropriate antibiotic prescribing from 2017 to 2022, which is encouraging following a number of reforms and initiatives in China (147)<br>• Diseases of the respiratory system (70.7%) was the most frequent target for antibiotics, with URTIs representing for 52.0% of these. Penicillins were the most commonly used antibiotics (64.4% of prescriptions)<br>• Only 66.2% of prescriptions for antibiotics were seen as inappropriate, with physicians over 35 years, holding the title of associate chief physician and possessing more than 11 years of experience more likely to prescribe antibiotics inappropriately<br>• Higher rates of inappropriate antibiotic use were also seen among children aged five or younger vs. other ages
	Shen et al., 2023 (152)	• Explore how clinical uncertainty influences antibiotic prescribing practices among hospital physicians and village doctors<br>• Qualitative semi-structured interviews involving 36 physicians using a guide based on previous publications and experience	• Respondents reported that URTIs were the most common reason for antibiotic prescriptions in their facilities<br>• Clinical uncertainty was an important driver for their overuse for URTIs, with antibiotics prescribed to prevent both prolonged courses or recurrence as well as clinical worsening, hospital admission, or complications<br>• Clinical uncertainty was primarily due diagnostic uncertainty and insufficient prognostic evidence
**Malaysia**	Ooi et al., 2022 (153)	• Evaluate antibiotic use among patients seeking care from a dedicated community-based URTI clinic during COVID-19<br>• Data obtained from the medical records of patients<br>• 587 patients sought treatment in the URTI clinic during the study period	• 96.5% of patients were provided with symptomatic treatment in the URTI clinic for their infection. These included antipyretics (51.8%), antihistamines (49.8%), and antitussive agents (45.9%)<br>• Encouragingly only 26 patients (6.0%) diagnosed with an URTI were prescribed an antibiotic by the treating HCPs including physicians
	Lim et al, 2024 (154)	• Comparing antibiotic utilisation patterns between the public and private community healthcare sectors 2018-2021<br>• Rates of antibiotic utilisation were reported as Defined Daily Doses per 1000 inhabitants per day (DID), by class and AWaRe classification	• Overall antibiotic utilisation for 2018 was 6.14 DID, 6.56 DID in 2019, 4.54 DID in 2020 and 4.17 DID in 2021.<br>• Private primary care antibiotic utilisation was almost ten times higher vs. public primary care in 2021<br>• Penicillins dominated public care antibiotics (over 80% total utilisation)<br>• Access antibiotics accounted for over 90% total antibiotic utilization in the public sector, while the use of Access antibiotics ranged from 64.2 to 68.3% of total utilization in the private sector, which is a concern

	Lim et al., 2024 (155)	• Assess changes in antibiotic utilisation and appropriateness following implementation of national guidelines (NAG) in 2014 and 2019 along with Clinical Pathways 2019 containing flowcharts for managing infectious diseases most seen in primary care e.g. acute bronchitis and pneumonia, acute otitis media, acute pharyngitis, acute rhinosinusitis, skin and soft tissue infections, UTIs and acute gastroenteritis<br>• Time series analysis using prescription data from PHCs in Malaysia	• NAG2014 had an impact on subsequent antibiotic utilisation ($p < 0.0001$), with NAG2019 having a substantial impact on antibiotic utilization - decreasing DDDs by 1778 and increasing appropriateness by 54.6% ($p < 0.0001$) following only a minor increase with NG2014<br>• Following NAG2019, appropriateness of antibiotic prescriptions for acute otitis media was 91.8%, 90.1% for acute pharyngitis and 86.0% for acute rhinosinusitis but only 66.1% for acute bronchitis<br>• Typically, the proportion of prescriptions with inappropriate antibiotics showed a decreasing trend<br>• Overall, fewer variation in the number of antibiotic molecules being prescribed after NAG2019 for almost all diagnoses except impetigo

*World Bank Classification; ABR = Antibacterial Resistance; AMR: Antimicrobial Resistance; ARI = Acute Respiratory Infection; AWaRe = Access, Watch, Reserve antibiotics; CAP = Community-Acquired Pneumonia; FGD = Focus Group Discussions; HCP = Healthcare Professional; HCW = Healthcare Worker; PHC = Primary Healthcare Centres; KAP: Knowledge, Attitude and Practice; STG = Standard Treatment Guidelines; URTI: Upper Respiratory Tract Infection

Table S5: Knowledge, Attitude and Practices, antibiotics, antimicrobial resistance and antimicrobial stewardship – WHO African Region Dispensers

Country	Author and Year	Objectives, Study Design and Population	Summary of the Findings
**Low Income countries***			
**Burkina Faso**	Valia et al., 2024 (8)	• Quantify antibiotic use by provider type as well as compliance with the WHO AWaRe Book<br>• 2196 patients were interviewed - 2108 had acute illness with 67.9% attending health centres for their treatment principally as outpatients (87.3%), with 15.6% visiting pharmacies and 16.6% informal medicine vendors	• Antibiotics were more frequently prescribed at health centres (54.8%) versus formal pharmacies (26.2%, $P < 0.001$) and informal medicine vendors (26.9%, $P < 0.001$)<br>• Among all antibiotics dispensed in pharmacies (self-medication), 84.6% were Access and 15.4% Watch antibiotics<br>• Clinical presentations for self-medication with antibiotics included bronchitis, gastroenteritis, dermatosis and pain – which accounted for 64.3% of all Watch antibiotics, which could have been avoided<br>• Ciprofloxacin was the most frequently dispensed Watch antibiotic for self-medication (71.4%)<br>• Overall, high rates of dispensing of antibiotics for self-limiting conditions including bronchitis (86.1%), with 37.5% of patients with a stomach ache dispensed antibiotics
**Democratic Republic of Congo**	Ingelbeen et al., 2022 (10)	• To quantify healthcare provider-specific and communitywide antibiotic use to different sectors including healthcare clinics, medicine stores and self-treatment<br>• Household survey based on previous surveys in two health zones	• Private clinics (31.0%) and PHCs (25.5%) were most frequently visited healthcare facilities<br>• Antibiotics were prescribed/ dispensed during 64.3% of visits to private clinics and 51.1% to PHCs vs. 48.8% to medicine stores.<br>• The most frequent antibiotic classes used were the penicillins (49.5%) and cephalosporins (14.2%), with cephalosporins more frequently used in private clinics (25.3%) vs. medicine stores (11.8%) or health centres (9.3%)

			• % of Watch antibiotics prescribed/ dispensed was highest in private clinics (30.3%) vs. medicine outlets (25.6%) or PHCs (25.1%)<br>• Treatment durations <3 days were more frequent among private clinics (5.3%) and medicine stores (4.1%) vs. PHCs (1.8%)
**Ethiopia**	Ayele et al., 2018 (156)	• Document the involvement of community pharmacy personnel in the management of minor ailments<br>• Simulated patient (SP) visits (uncomplicated URTIs, back pain and acute diarrhoea) – 66 - combined with in-depth interviews	• 92.4% of community pharmacy personnel provided one or more medications to the SPs<br>• Ibuprofen alone or in combination with paracetamol were the most commonly dispensed analgesics for back pain, with oral rehydration fluid with zinc the most frequently dispensed medication (33.3%) for acute diarrhoea followed by mebendazole (23.9%). However, metronidazole (19.0%) and cotrimoxazole (19.0%) also dispensed<br>• Antibiotics were dispensed for all SPs with URTIs, with amoxicillin-clavulanic acid (35%) and amoxicillin (25%) the most commonly dispensed
	Erku et al., 2018 (157)	• Assess extent and motivations behind non-prescribed sales of antibiotics for acute childhood diarrhoea and URTIs<br>• Mixed methods including simulated patients (SP – 50 for each indication) and interviews	• 86% of SPs were provided with one or more medications<br>• The most frequently dispensed single medication for acute childhood diarrhoea was oral rehydration fluid (ORS) with zinc (32%). In all, 80% also received antibiotic – principally metronidazole (30%) and cotrimoxazole (22%)<br>• 92% of SPs were dispensed antibiotics for URTIs - the most common was amoxicillin (46% of all SPs) followed by amoxicillin-clavulanic acid (38%) and azithromycin (30%)<br>• Perceived financial benefits as well as high expectations and/or demand of patients were the principal drivers for selling antibiotics without a prescription
	Belachew et al., 2022 (158)	• Assess the knowledge and attitudes surrounding antibiotic use or supply and antibiotic ABR and dispensing practices in community drug retail outlets<br>• Cross-sectional survey using a validated self-administered questionnaire<br>• 276 staff members participated (86.8% response rate)	• 31.9% believed antibiotics can treat viral diseases, 45.1% that UTIs can be treated with antibiotics irrespective of the cause and 26.1% that acute sore throats can be treated with antibiotics whatever the cause<br>• Encouragingly only 9% believed keeping leftover antibiotics from a previous course to be used the next time is acceptable, only 19.6% that antibiotics should be stopped when symptoms improve and only 19.5% that colds and coughs should always be treated with antibiotics<br>• In addition, only 19.6% of participating staff members disagreed that dispensing antibiotics without a prescription will lead to ABR and 16.6% that inappropriate use of antibiotics increases ABR<br>• Overall, whilst participants had appropriate knowledge about and attitudes to antibiotic use and ABR, basic knowledge and attitude gaps still remain
	Ayenew et al, 2024 (159)	• Assess the prevalence of antibiotic self-medication and its associated factors in Ethiopia<br>• Systematic review and meta-analysis involving 9 studies	• The pooled prevalence of antibiotic self-medication among Ethiopians was 46.14%, with the most frequently used classes being the penicillins followed by tetracyclines<br>• Community pharmacies were the principal source of information regarding antibiotics/ infectious diseases<br>• The most common reported reasons for self-medication were previous experiences, saving cost, lack of time and avoiding long waiting times<br>• Participants with less than high school educational level had higher rates of self-medication with antibiotics

	Edessa et al, 2024 (160)	• Explore drug providers' perspectives on antibiotic misuse practices<br>• Qualitative study involving in-depth interviews among 15 drug providers using a structured questionnaire	• Pressures from patients, insufficient regulatory functions and a lack of specific antibiotic use policies are key contributors to antibiotic misuse<br>• Previous experiences, desire to avoid extra costs and lack of essential diagnostics are also key drivers of non-prescribed antibiotics access<br>• Non-prescribed antibiotic dispensing is also driven by client satisfaction, financial gain and market competition from informal sellers exacerbated by ineffective dispensing audits, inadequate regulatory oversights and policy gaps – all of which need addressing going forward
**Mozambique**	Torres et al., 2020 (161)	• Describe the practices and enablers for non-prescribed dispensing of antibiotics<br>• Qualitative in-depth study among 17 community pharmacists using a structured questionnaire	• Of concern is that interviewed pharmacists typically described their roles primarily as more selling of antibiotics than dispensing them<br>• In most cases, patients know what medicines they want with pharmacists typically handing over requested antibiotics without asking for a prescription or providing health information to patients<br>• These activities are enhanced by patients' behaviour and their beliefs regarding the curative power of antibiotics. In addition, current bonuses for pharmacists to sell medicines<br>• Raising awareness of AMR among patients and increasing awareness/ skills among community pharmacists could help in the future to reduce inappropriate dispensing of antibiotics
	Torres et al., 2020 (162)	• Identifying the commonly used antibiotics dispensed without a prescription and the principal health conditions<br>• Cross-sectional study involving both individual and FGDs using a structured questionnaire<br>• 32 patients visiting pharmacies and 17 pharmacists participated	• 88.2% of surveyed pharmacists admitted dispensing antibiotics without a prescription<br>• The most commonly dispensed antibiotics were amoxycillin (also known as 'two colours medicine'), cotrimoxazole and co-amoxiclav<br>• A concern was that patients requested antibiotics in smaller quantities than current recommendations<br>• Sore throats, fever, coughs, influenza, RTIs, UTIs and vaginal discharge were common conditions where antibiotics were purchased, with requests for antibiotics for STIs increasing
**Uganda**	Lanyero et al., 2020 (163)	• Determined the prevalence and factors associated with antibiotic use in managing ARIs in children in rural communities<br>• Cross-sectional survey among households using a questionnaire adapted from previous studies<br>• 856 households took part (98.9% response rate)	• 52.7% of caregivers visited health centres for their children with 47.3% self-medicating their children<br>• 60.2% of children were treated with antibacterials; the symptoms most commonly associated with antibiotics included runny noses (58%), cough (67%), fever (61%), fast breathing (79%), having symptoms of ARIs with diarrhoea (54%)<br>• The most commonly used antibiotics were amoxicillin (55.2%), cotrimoxazole (15.4%) and metronidazole (11.4%)<br>• Determinants of antibiotic use included getting treatment from a health facility, households located in peri-urban area and a child having a cough
	Bagonza et al, 2021 (164)	• Assess the effectiveness of peer-supervision among drug sellers on the appropriate treatment of pneumonia, malaria and non-bloody diarrhoea among children under 5 years of age	• 21.4% of surveyed children were appropriately treated with antibiotics for pneumonia symptoms in the comparison district while 15.4% of the children were appropriately treated for pneumonia symptoms in the intervention district<br>• A month after the introduction of peer supervision, the proportion of children appropriately treated for pneumonia symptoms was 10.84% higher in the intervention group

		• Data extracted from drug shop sick child registers over a 12-month period<br>• Interrupted time series analyses to determine the effectiveness of peer-supervision intervention	• Prior to introduction of peer supervision, 50.6% of the children were appropriately treated for uncomplicated malaria in the comparison district<br>• A month after the introduction of peer supervision, the proportion of appropriately treated children for uncomplicated malaria was 1.46% higher in the intervention group<br>• In the first month of the intervention, the proportion of appropriately treated children with non-bloody diarrhoea was 4% higher in the intervention compared to the comparison district.
	Kiragga et al., 2023 (165)	• Assess the extent of self-purchasing of medicines, especially antibiotics, pre- and during COVID-19<br>• 19,285 customers were surveyed using electronic sales data	• Self-purchasing of antibiotics was high pre-COVID-19. Out of 19,285 customers surveyed antibiotics purchased included azithromycin (n = 6077), ciprofloxacin (n = 6066) and erythromycin (n = 997)<br>• Antibiotics purchased to treat COVID-19 included azithromycin, erythromycin and ciprofloxacin as well as supplements including zinc and vitamin C and dexamethasone<br>• The number of patients purchasing azithromycin during the COVID-19 pandemic increased (19.7%) but decreased for erythromycin (-35.8%) and ciprofloxacin (-58.8%)
	Murungi et al., 2023 (31)	• Determine the national consumption of all antimicrobials in Uganda as a reflection of prescribing and dispensing behaviour<br>• Consumption by antibiotic class and DDDs/ 1000 inhabitants as well as by AWaRe classification	• In 2021, the average DDD per 1000 inhabitants was 29.02 for all antimicrobials - 80.7% were oral<br>• Penicillins (27.6%) were the most consumed antimicrobial class, followed by sulfonamides and trimethoprim (15.5%)<br>• 62.91% of current utilisation was from the Access class, with those from the Watch class averaging 14.51% between 2018–2021, spiking in 2021 (34.2%) during COVID-19 pandemic compared to 2020 (24.29%). Azithromycin and ciprofloxacin were the most consumed Watch class in 2021<br>• Overall, very limited use of Reserve antibiotics
**Zambia**	Mudenda et al., 2021 (166)	• Assess the KAP of community pharmacists on antibiotic use, ABR and ASPs<br>• Cross-sectional study using a questionnaire modified from previous studies and pretested<br>• 144 community pharmacists participated	• 93.8% of participating community pharmacists had good knowledge while 67% had positive attitudes towards antibiotics, e.g.96.6% agreed/ strongly agreed that ABR arises when antibiotics no longer work to treat bacterial infections, 99.3% that ABR is a problem in our community and 94.4% that many infections are becoming increasingly resistant to treat in Zambia<br>• In addition, 99.3% believed that pharmacists have to take responsibility to reduce ABR in Zambia<br>• However, 75% of participating pharmacists demonstrated poor practices towards ABR and AMS including dispensing antibiotics for longer than required and only sometimes educating patients on antibiotics
**Lower-Middle Income countries***			
**Ghana**	Afari-Asiedu et al., 2018 (167)	• Assess differences between regulatory and community demands on the sale of antibiotics and explore how these differences could be resolved to facilitate appropriate use of antibiotics in rural Ghana<br>• Mixed method approach involving 72 dispensers and community members in in-	• Abuse of antibiotics, including extensive self-medication is common, often for conditions that do not warrant antibiotics, e.g. stomach pains, hernias, wounds/sores, boils and headache with amoxicillin, tetracycline, metronidazole and chloramphenicol generally used to treat stomach aches

		depth interviews and FGDs using structured questionnaires	• Generally, community members did not know what antibiotics are and the diseases they should be used for with for instance tetracycline and metronidazole poured into "akpeteshie" (local gin) to treat hernia and perceived stomach sores<br>• Overall, inappropriate antibiotic use was influenced by general lack of knowledge on antibiotics among key stakeholders and identification of antibiotics by colours leads to confusion following requests and could lead to inappropriate antibiotic use which needs addressing
	Afari-Asiedu et al., 2021 (168)	• Assess the potential training of over-the-counter medicine sellers (OTCMS) and Community-based Health Planning and Services (CHPS)/health posts to appropriately dispense some antibiotics<br>• 10 in-depth interviews using a structured questionnaire among staff of the Ghana health services (GHS), pharmacy council and the association of OTCMS	• Two opposing views – The Leadership of OTCMS and GHS staff at the district health directorate supporting that OTCMS and CHPS personnel should be trained to dispense specific antibiotics as they are already dispensing them<br>• Participants from pharmacy council and GHS personnel in the Regional and National AMR platforms generally believed that OTCMS and CHPS should not be trained to dispense antibiotics because their level of education is inadequate – with concerns from GHS personnel that training OTCMS could further compromise inappropriate antibiotic use in the country. They suggested that OTCMS and CHPS should focus on public health education/ disease prevention and appropriate antibiotic use in their discussions with patients
	Ngyedu et al., 2023 (169)	• Evaluate the prevalence and practice of selling antibiotics without prescriptions among community pharmacies and drug outlets<br>• Simulated client study with 2 scenarios – URTIs and paediatric diarrhoea<br>• 265 out of 385 outlets were visited (68.8%)	• The prevalence of selling antibiotic without prescription was 88.3% - greatest for paediatric diarrhoea (95.3%) than URTIs (77.6% - cough syrups offered on 10% of occasions)<br>• Variations seen across the four regions. Variations also seen among pharmacy types<br>• Of concern was that azithromycin was the antibiotic typically offered (63/64 occasions) with metronidazole typically offered for paediatric diarrhoea<br>• Encouragingly – where antibiotics were refused – this involved a pharmacist
	Greene et al., 2024 (170)	• Assess challenges faced by medicine vendors related to dispensing antibiotic as well as identify opportunities for improving AMS<br>• 79 dispensaries surveyed using a structured instrument based on the literature and piloted	• Customers' attitudes were the most prevalent challenge and addressing this challenge is a problem with notion that the "the customer is always right" and how this idea influences responses to customers when they wish an antibiotics<br>• Language can also be a barrier when discussing possible treatment approaches and the rationale<br>• There were also concerns whether patients comply with a full course of antibiotics in view of financial difficulties with their costs – addressed to some extent by pharmacists calling patients to enquire whether they have completed the course
**Kenya**	Muloi et al., 2019 (171)	• Assess patterns of antibiotic sales and evaluate awareness and common behaviours regarding antibiotic use AMR<br>• Cross-sectional study using a pre-tested questionnaire<br>• 40 drug store personnel took part	• Penicillins (93%), metronidazole (65%), fluoroquinolones (63%) and first and second-generation cephalosporins (43%) were among the four most commonly sold antibiotic classes – with 52% stating they sold antibiotics without a prescription<br>• Encouragingly, more than two-thirds were aware of the terms "antibiotic resistance" and "drug resistance", with more than 75% agreeing ABR was increasing. However, fewer than half had heard of "AMR" and "super bugs" and

			>80% believed that AMR occurs when their body becomes resistant to antibiotics rather bacteria developing resistance<br>• Of concern as well is that increasing customer demand was the principal reason behind increased sales of antibiotics (58% of drug stores), with antibiotics frequently dispensed for RTIs (83%), GI infections (65%) and sore throats (58%) despite more than 80% of respondents agreeing that antibiotics should be used more prudently
	Kimathi et al., 2022 (172)	• Assess the extent of self-purchasing of antibiotics among patients with COVID-19 as well as KAP on self-medication<br>• Telephone-based survey using a coded questionnaire<br>• 280 participants took part	• High degree of self-medication with antibiotics dispensed from pharmacies/ drugs stores (23.6%) self-medicated with antibiotics<br>• This compares with feedback from 6 pharmacies allied to the University of Nairobi in the study of Opanga et al. where no pharmacists dispensed antibiotics for patients with COVID-19 - suggesting symptomatic relief (173) enhanced by knowledge of antibiotics and AMR - similar to the findings of a pilot study of Mukokinya et al. (2018) among community pharmacies allied to the University of Nairobi where no antibiotics were dispensed without a prescription for patients presenting with common colds or influenza - enhanced by their knowledge base (174)
	Gacheri et al. 2024 (175)	• Assess patterns of antibiotic dispensing and use in the community during the recent COVID-19 pandemic<br>• Conducted interviews with 243 pharmacies using a standardized and piloted questionnaire	• All pharmacies contacted reported selling one or more antibiotics to customers suspected of having COVID-19, with 81.5% reporting they prescribed an antibiotic without asking for a prescription<br>• Azithromycin (99.1%), amoxicillin-clavulanic acid (77%), cefuroxime (64.9%) and amoxicillin (approx. 50%) were the four most commonly sold antibiotics to customers suspected of having COVID-19<br>• This contrasts with the study of Opanga et al. where no pharmacy dispensed antibiotics for patients with COVID-19 - suggesting symptomatic relief (173)
**Nigeria**	Abubakar et al., 2020 (176)	• Evaluate KAP of community pharmacists towards dispensing antibiotics without prescription<br>• Cross-sectional using a validated self-administered questionnaire<br>• 98 pharmacists took part (75.3% Response Rate)	• 39.7% of community pharmacists indicated that they dispensed antibiotics without a prescription 5 or more times a day<br>• Antibiotics dispensed were for UTIs (83.7%), typhoid fever (83.7%) and STIs (66.3%)<br>• Pharmacist's confidence in their knowledge was the most common reason they dispensed antibiotics without a prescription<br>• Pharmacists with < 5 years of working experience were significantly more likely to dispense antibiotics without prescription 5 times or more in a day compared to those with more than 5 years community pharmacy experience
	Akpan et al., 2021 (177)	• Assess community pharmacists' management of self-limiting infections<br>• Simulated patients visiting 75 pharmacists<br>• Information recorded on developed data collection form after each visit	• 68% of pharmacists visited recommended an antibiotic for a common cold - with azithromycin (43%), co-amoxiclav (24%) and cotrimoxazole (20%) the most frequently dispensed antibiotics<br>• 72% of pharmacists dispensed one antibiotic for acute diarrhoea with 15% dispensing more than one antibiotic. The most frequently dispensed antimicrobial for this condition was metronidazole - 82% of occasions
**Tanzania**	Poyongo and Sangeda, 2020 (178)	• Determine the proportion of pharmacists who dispense without a prescription, reasons for this and their awareness of the	• 77.7% of surveyed pharmacists had excellent knowledge about the legal requirements for dispensing antibiotics and the AMR challenge with 72.6% admitting to dispensing antibiotics without a prescription in their daily practice.

		legal requirements to dispense antibiotics and AMR.<br>• Cross-sectional study using an online and piloted semi-structured questionnaire<br>• 197 pharmacists participated (20.5% response rate)	• 84.1% of surveyed pharmacists with a masters or PhD education were more likely to dispense without a prescription compared to 69.3% among bachelor holders (p-value = 0.04).<br>• Reasons for administering antibiotics without a prescription included the pharmacy business looking for more profit, patient failure to obtain a prescription and the lack of stringent inspection, with 71.6% of pharmacists believing they are knowledgeable enough to dispense without a prescription after a critical evaluation of the patient<br>• Of concern, the leading conditions for which antibiotics are commonly dispensed without a prescription include UTIs (72.6%) and coughs (56.9%) as well as colds and influenza (24.4%)<br>• Penicillins, macrolides and fluoroquinolones were the classes of antibiotics most commonly dispensed without a prescription
	Ndaki et al., 2021 (179)	• Assess antibiotic dispensing practices of pharmaceutical providers – particularly dispensing without a prescription<br>• Cross-sectional study using mystery clients' method among 1148 community pharmacies and accredited drugs dispensing outlets who asked directly for amoxicillin (and if asked for UTIs)	• Dispensing of amoxicillin without prescription was common - 88.2% of pharmacies across all three regions<br>• The vast majority of outlets were happy to sell a half course of amoxicillin on demand - Mwanza (98%), Mbeya (99%) and Kilimanjaro (98%) – typically without asking the client any questions<br>• There were though significant variations among regions - in Mbeya and Kilimanjaro, accredited drug dispensing outlets were more likely to do this than pharmacies but no difference was observed in Mwanza
	Myemba et al., 2022 (180)	• Assess the antimicrobial dispensing practices among drug dispensing outlets and key factors influencing the use of antimicrobials among children<br>• Community-based cross-sectional study utilizing both interviews) and simulated clients	• Overall considerable concerns with inappropriate dispensing and use of antimicrobials common among children - influenced by multiple factors including lack of knowledge/ poor attitude to antibiotics among patients and dispenser, financial constraints, and product-related factors<br>• Only 8% of dispensers asked for prescriptions, with 90% of dispensers willing to dispense without prescriptions<br>• 83% supplied incomplete doses of antimicrobials and only 60.5% gave proper instructions for antimicrobial use to simulated clients
	Ndaki et al, 2022 (181)	• Explore compliance with regulations as well as variations in dispensing practices/ drug recommendation, and the quality of dispenser-client interactions among different types of drug outlets<br>• 672 Drug Dispensing Outlets and community pharmacies were visited by mystery clients with UTIs	• 89.43% of drug sellers recommended antibiotics with UTIs although no prescriptions, with 58.93% willing to sell less than the minimum recommended course<br>• Female clients more likely to be asked if they were taking other medications (27.2% vs 9.8% for males) or had seen a doctor (27.8% vs 14.7%), and more likely to be advised to consult a doctor (21.6% vs 9.0%)<br>• Sellers recommended 32 different drugs to treat UTI symptoms, with only 7 appearing in the Tanzanian STG and 30% were 2nd and 3rd line drugs<br>• Principal antibiotics were azithromycin (35.4%) and ciprofloxacin (20.5%), with azithromycin suggested more often in pharmacies (40.8%) than in Drug Outlets (34.4%) and more often to male clients (36.0%)
	Ndaki et al., 2023 (182)	• Explored the practices and motives behind dispensing of antibiotics without prescription among community drug outlets in Tanzania	• Majority of dispensers admitted to providing antibiotics without prescriptions, selling incomplete courses of antibiotics and not giving detailed instructions to patients on how to use antibiotics

		• 28 In-depth interviews with Drug Dispensing Outlets and Community Pharmacists	• Inappropriate practices were motivated by a number of factors including pressure/demands from patients, business orientation/ financial gain for outlets and low purchasing power of patients/customers with high co-payments to see physicians and purchase medicines
**Upper-Middle Income countries***			
**Botswana**	Sono et al., 2023 (183)	• Narrative review paper discussing possible self-purchasing of antibiotics in Botswana<br>• Expert input from co-authors in Botswana	• Belief that little or no self-purchasing of antibiotic in Botswana over 85% of the population receiving health care services free-of-charge<br>• In addition, only prescribed medicines can be dispensed from public sector health facilities, which are provided free-of-charge, with the Botswana Medicines Regulatory Authority strictly enforcing current regulations
**Namibia**	Kamati et al., 2019 (184) and Sono et al., 2023 (183)	• Assess the extent of self-medication with antibiotics for children with ARIs among members of informal settlements in Namibia<br>• Cross-sectional study using a structured questionnaire	• Self-medication for ARIs in children under five included cold/ flu medicines, paracetamol, and decongestants<br>• However, no self-purchasing of antibiotics - enhanced by the banning of self-purchasing of antibiotics, training of pharmacist at the University on antibiotics and AMR, regular monitoring of pharmacies and access to PHCs (183)<br>• This was also seen in pharmacies in Namibia during the COVID-19 pandemic with other suggestions to prevent and treat COVID-19 (185)
**South Africa**	Truter and Knoesen 2018 (75)	• Determine current antibiotic prescribing habits as well as the perception of pharmacists towards their prescribing of antibiotics<br>• Self-designed questionnaire based on the literature<br>• 16 community pharmacists participated	• 81.3% of community pharmacists felt that antibiotics were being over-prescribed, including for viral infections, exacerbated by pressure from patients<br>• Amoxicillin /co-amoxiclav were the most dispensed antibiotics followed by clarithromycin, ciprofloxacin and azithromycin with URTIs and sinusitis the most common infectious diseases<br>• Pharmacists had mixed opinions whether they should prescribe antibiotics - 50.0% did not agree and 31.3% agreed – with most pharmacists concerned are not qualified to diagnose patients. However, they believed that with further training they should be able to diagnose minor ailments and counsel patients when requested
	Balliram et al., 2021 (78)	• Assess KAP of doctors, pharmacists and nurses regarding antimicrobials, AMR and AMS.<br>• National online survey<br>• 744 pharmacists participated (4.92% response rate)<br>• 1120 doctors and 659 nurses also participated (comprising the total number of HCPs participating)	• Encouragingly, 93.37% of HCPs recognized AMR as a severe global threat (95.0% for pharmacists), with 95.8% of participating pharmacists seeing it as a significant problem in South Africa.<br>• 45.3% of pharmacists felt ≤ 50% confidence in their knowledge of antimicrobials, AMR and AMS, higher than doctors but lower than nurses. However, (25%) reported no confidence in prescribing antimicrobials.<br>• 92.6% believed antibiotics are not effective against viral infections vs. 75.3% for nurses and 97.3% that common colds are caused by viruses vs. 90.2% nurses<br>• 91.61% of HCPs identified overuse of antimicrobials greatest contributor to AMR followed by patient pressure (75.26%) and non-adherence to prescribed treatments (73.26%)<br>• 38.7% of pharmacists had attended workshops and training on either AMS and antimicrobials; however, 79.2% expressed a need for more education and training on antimicrobial use, AMR, and AMS

			• 91.22% of HCPs identified educational campaigns as the most effective strategy to combat AMR, followed by STGs (84.72%) and improved infection control measures (66.31%)
	Mokwele et al., 2022 (186)	• Determine the extent of antibiotics being dispensed without a prescription in community pharmacies and whether guidance given to patients<br>• Simulated patient study with patients with URTIs and UTIs among 34 pharmacies (20 private and 14 franchised)	• Antibiotics were sold in privately owned pharmacies without a prescription in 80% of cases while no antibiotics were dispensed in franchised pharmacies - similar to Sono *et al.* 2024 (187, 188); however, different to Anstey Watkins *et al.* and Do et al. where little or no purchasing of antibiotics without a prescription was seen in South Africa (189, 190)<br>• Among pharmacies selling antibiotics without a prescription, pharmacist assistants were involved in 37.5%, and counselling was not provided to 19% of simulated patients<br>• Encouragingly, no antibiotics were dispensed for simulated patients with URTIs, only UTIs.

*World Bank Classification; ABR = Antibacterial Resistance; AMR: Antimicrobial Resistance; AMS: Antimicrobial Stewardship; ARIs: Acute Respiratory Infections; ASPs: Antimicrobial Stewardship Programmes; AWaRe = Access, Watch, Reserve; HCPs: Healthcare Professionals; HCW = Healthcare Worker; PHC = Primary Healthcare; RTIs = Respiratory Tract Infections; UTIs = Urinary Tract Infections; URTIs = Upper Respiratory Tract Infections

Table S6: Knowledge, Attitude and Practices, antibiotics, antimicrobial resistance and antimicrobial stewardship – WHO Eastern Mediterranean Region Dispensers

Country	Author and Year	Objectives, Study Design and Population	Summary of the Findings
**Low Income countries***			
**Yemen**	Orubu et al., 2021 (88)	• Assessing prescribers'/physicians' and pharmacists' perceptions of antibiotic use, AMR and AMS<br>• Mixed-method study involving 27 physicians and 27 pharmacists using semi-structured questionnaires in Arabic and English (41% response rate)	• 56% of physicians and 70% of pharmacists perceived AMR to be an understudied/ underreported problem, with physicians (37%) in support of the enforcement of prescription laws to reduce purchasing of antibiotics without a prescription vs. increasing awareness of AMR among patients (22%) and pharmacists more in support of increased training (59%) and increased awareness among clients (22%) to improve future use<br>• Of concern is that 74% of physicians felt under pressure to prescribe broad spectrum antibiotics with reasons for empiric use being cost (59%) and patient symptoms (56%). The practice of selling antibiotic without a prescription was reported by 67% of pharmacists alongside counter prescribing (63%)<br>• Infections for which antibiotics were demanded included inflammation affecting the throat/mouth or ear (37%), UTIs) and RTIs (33% each)<br>• Penicillins were the most commonly prescribed and dispensed antibiotic group - of the penicillins, amoxicillin (and amoxicillin/ clavulanic acid) was the most commonly prescribed (63%) or dispensed (81%)<br>• Ciprofloxacin was the most common quinolone antibiotic prescribed (52%) or dispensed (74%)<br>• AMS activities were poor with limited use of AST impacting on awareness of resistant pathogens among HCPs in Yemen (89). This needs addressing going forward

Low-Middle Income countries*			
**Egypt**	Abdelaziz et al., 2019 (191)	• Examined antibiotic dispensing patterns among Egyptian community pharmacies<br>• Simulated patient (SP) study where amoxicillin was available<br>• SPs with acute bronchitis visited 125 pharmacies and those with common colds 113 pharmacies requesting amoxicillin<br>• Subsequent interviews with 83 pharmacies (83% response rate)	• Encouragingly, 96% of interviewed pharmacists agreed/ strongly agreed that AMR was a major public health problem, with 64% stating that irrational prescribing/ dispensing of antibiotics was a major contributing factor<br>• However, amoxicillin was dispensed to 97.6% of SPs with acute bronchitis and to 99.1% of SPs with common colds<br>• 63.2% of pharmacies dispensed amoxicillin for acute bronchitis without collecting any information about the patient's clinical condition and 62.8% of SPs with common colds<br>• This is despite 66.1% of participating community pharmacists stating they often/ always asked patients about their symptoms when dispensing medicines, 50.8% about symptom duration and 86.4% about the problem history<br>• In addition, 35.5% agreed/ strongly agreed that an antibiotic was indicated for these two infectious diseases
	Zakaa El-Din et al., 2019 (192)	• Assess basic knowledge and attitudes towards ABR and antibiotic dispensing among community pharmacists in Egypt<br>• Cross-sectional using a self-administrated questionnaire<br>• 461 pharmacists took part (86.8% response rate)	• 51.2% of participating pharmacists were considered to have a good basic knowledge regarding antibiotic use and ABR.<br>• 74% of pharmacists strongly agreed that antibiotics are over-prescribed in Egypt, with 90% hoping that the Ministry of Health would publish guidelines for antibiotic dispensing in Egypt to improve future practices<br>• Male pharmacists were more likely to engage and communicate with patients or prescribers during the dispensing process compared with female colleagues
	Kandeel et al., 2019 (90)	• Assess the effectiveness of campaigns to raise the awareness of physicians, pharmacists, and the public regarding antibiotic prescribing for ARIs. This included educational courses for pharmacists<br>• Structured surveys with pharmacists<br>• 1492 participants including 596 pharmacists (39.9% of total)	• Encouragingly, the mean knowledge supporting the judicious use of antibiotics improved among participating pharmacists from 3.3 ± 0.9 to 4.0 ± 1.2<br>• Attitude scores regarding reducing antibiotic prescribing for colds, limiting the use of antibiotics to preserve their effectiveness and not giving antibiotics for nasal discharge to treat colds also improved among participating pharmacists<br>• Attitudes related to not overusing antibiotics to prevent resistant bacteria also improved among pharmacists<br>• The % of participating pharmacists who stated that they never prescribe/ dispense antibiotics for common colds increased from 16.1% to 29.7% and decreased sometimes from 58.4% to 25.2%
	Elsayed et al., 2021 (193)	• Describe antibiotic misuse and its contributing factors during COVID-19 pandemic as well as pharmacists' application of infection preventive practices<br>• Cross-sectional study using a structured questionnaire<br>• 413 community pharmacists took part (87% response rate)	• 86.2% of participating pharmacists kept regular hand sanitization<br>• 67% of surveyed pharmacists reported that patients were more likely to be given antibiotics for showing any sign or symptom of COVID-19 infection, and 82% of the dispensed antibiotics were given upon physician recommendations<br>• Azithromycin, ceftriaxone, and linezolid were the principal antibiotics dispensed to COVID-19 presumptive patients with azithromycin was given to approx. 40% of presumptive patients showing only mild or moderate symptoms for 5-10 days<br>• Antibiotic combinations were given to 74% of home-isolated patients with COVID-19 for a maximum of 2 weeks

**Pakistan**	Rehman et al., 2018 (194)	• Assess knowledge and practice of pharmacists working in various settings towards AMS in Pakistan<br>• Cross-sectional study among 181 pharmacists (72.4% of those approached) using a validated questionnaire	• 83.9% of participating pharmacists agreed that AMS is essential to improve patient care, with only 8.8% disagreeing that AMS programs reduce AMR<br>• 87.8% of pharmacists also agreed that pharmacists should be trained on the use of antimicrobials<br>• However, only close to 40% of surveyed pharmacists currently make efforts to prevent or reduce the transmission of infections within the community, only 32% communicate with prescribers if they are unsure about the appropriateness of an antibiotic prescription and only 28.7% specifically ask patients about their knowledge of any prescribed antimicrobial and its usage to improve medication use
	Sarwar et al., 2018 (195)	• Assess knowledge regarding antibiotics, AMS perception and its practice among community pharmacist<br>• Cross sectional study using self-administered questionnaire<br>• 400 pharmacist completed survey (96.6% response rate)	• Overall, good knowledge about antibiotics among surveyed pharmacists, with for instance 84.5% of participants strongly agreeing/agreeing that antibiotics are useful for bacterial infections<br>• Encouragingly, all participants strongly agreed/agreed that pharmacists have a responsibility to take a prominent role in AMS and infection-control programs in their locality, with 78% strongly agreeing/ agreeing that AMS should be incorporated at the community-pharmacy level<br>• However, only 37.8% strongly disagreed/disagreed that antibiotics are useful for viral infections such as influenza and only 31.5% disagreed/ strongly disagreed that the misuse of antibiotics can lead to a loss of sensitivity of an antibiotic to specific pathogens<br>• In addition, 53.8% of participants had never taken part in antibiotic awareness campaigns to promote the better use of antibiotics and only 6.3% currently often educate their patients on antimicrobials and AMR issues
	Waseem et al., 2019 (93)	• Assess patients, physicians, and pharmacists' knowledge, attitudes and behaviour towards AMR using a specifically designed questionnaire<br>• 160 pharmacies (37.7% of those approached) were included in the study	• Encouragingly, 73.3% of qualified pharmacists possessed good knowledge of ABR and also understood the necessity to return unwanted medicines and AMR stewardship<br>• However, a qualified pharmacist was not present in 90.6% of the pharmacies surveyed. This is a concern as 86.2% of the non-qualified pharmacy owners had poor knowledge of AMR and ASPs<br>• 74.4% agreed that patients demand antibiotics from them without a prescription, with 76.6% acknowledging the need for ASPs
	Atif et al., 2020 (196)	• Assess community pharmacists' knowledge, perceptions and current practices towards ASPs.<br>• Qualitative study via in-depth, semi-structured interviews<br>• 15 community pharmacists took part	• All participating pharmacists had good knowledge regarding irrational antibiotic use. However, for measures to reduce irrational use participating pharmacists gave different suggestions. Suggestions included the implementation of prescription monitoring systems, education of the general public, development and implementation of pharmacy practice guidelines and avoidance of broad-spectrum antibiotics<br>• Whilst AMS was a new concept for most of the participating pharmacists, after explanation they showed a significant interest in ASP activities

			• However, all respondents had concerns with the implementation of different ASP components due to absence of patient records and physicians' reluctance to cooperate – and utilization data typically only used for procurement purpose and inventory maintenance
	Saleem et al., 2020 (197)	• Assess the extent of antibiotic sales without a prescription in urban areas<br>• Cross-sectional study using the Simulated Client technique<br>• Three demand levels were used to convince pharmacy to dispense antibiotics without a prescription – key areas were RTIs and acute diarrhea with demand level 3 being insistence of an antibiotic without a prescription	• 353 pharmacies and medical stores were visited out of which 96.9% dispensed antibiotics without demanding a prescription (82.7% at demand level 1 and 14.2% at demand level 2)<br>• The most frequently dispensed antibiotic was ciprofloxacin (22.1%), with Watch antibiotics comprising 49.3% of those dispensed<br>• Of equal concern was that the Reserve group of antibiotics compromised 19.0% of all antibiotics dispensed<br>• In addition, in only 25.2% visits did pharmacy staff guide patients about the use of antibiotics, and in only 11.0% of cases they enquired about the history of other medications
	Akbar et al., 2021 (198)	• Assessment of community pharmacists' knowledge regarding antibiotics and their perception and practices regarding AMS<br>• Descriptive cross-sectional study using a self-administered questionnaire based on the literature<br>• 100 pharmacists completed questionnaire (70.9% response rate)	• Encouragingly, most surveyed pharmacists accepted they played an active role in educating patients regarding antimicrobial use and AMR.<br>• However, whilst AMS practice and perceptions were not affected by gender, age, work experience and education – response rates to AMR were highest (70.9%) among experienced pharmacist. Experienced pharmacist also had greater knowledge that AMS played a role in the emergence of AMR and ultimately enhanced the patient care<br>• A large number of surveyed pharmacists also concurred that self-medication practices among the population was the major contributing factor towards AMR development<br>• In addition, the majority stated they adhered to the prescribed duration for antibiotic dispensing although a few extend the duration beyond the prescription. Most also stated there is a need for implementation of guidelines to improve future use
	Khan et al., 2021 (199)	• Evaluate KAP of community pharmacist regarding the dispensing of antibiotics without a prescription and how to improve the rational use of antibiotics<br>• Two-phased mixed-method approach using an online validated questionnaire followed by semi-structured interviews<br>• 180 pharmacists completed the survey, followed by 21 in-depth interviews	• Encouragingly, 65.6% of those surveyed had knowledge of multi-drug resistant pathogens and their role in AMR; however, 31.1% were unable to differentiate antibiotic groups on the basis of resistance pattern<br>• 90% of participating pharmacists also identified ABR as a major health issue, with 71.7% recognizing a high ABR rate in hospital settings<br>• Encouragingly as well, 52.8% educate patients about the appropriate use of antibiotics and 47.2% reported their participation in campaigns related to appropriate antibiotic use. 48.3% also reported their engagement in practices related to infection prevention and their transmission and 41.1% considered antibiotic screening before dispensing by using local guidelines<br>• 68.3% considered the dispensing of antibiotics without a prescription a crucial issue which occurred primarily due to lack of time and financial resources to consult physicians (43.9% of respondents)<br>• The most common issues that need to be addressed to reduce ABR were effective ASPs, patient education and training of pharmacists

	Mustafa et al, 2022 (200)	• Evaluate pharmacy technicians' knowledge regarding antibiotic use, resistance and AMS programs who typically serve in ambulatory care facilities<br>• Cross sectional survey using a structured questionnaire based on previous studies and piloted<br>• 376 pharmacy technicians took part (85.8% response rate)	• 79.5% knew that antibiotics ranked among the most highly prescribed classes of medicine in Pakistan, 59.8% agreed that antibiotics did not accelerate recovery from common colds, 55.1% that antibiotics should not be prescribed for future bouts of influenza and 52.4% that antibiotics should not be used to treat influenza<br>• Encouragingly as well, 59.6% stated that inadequate duration of antibiotics and inadequate dosage (57.7% of participants) are the leading causes of AMR. In addition, 81.4% knew about terms such as 'AMR', 91.8% about 'antibiotic resistance' and 76.9% about 'drug resistance'<br>• However, 36.2% believed antibiotics were first-line treatment for sore throats and 43.1% that left-over antibiotics can be used again for similar infections<br>• In addition, awareness of the terms 'superbug', 'extensively drug resistance' and 'multi-drug resistance' was only seen among a minority at 42%, 20.7% and 25.3% of participants respectively
	Rakhshani et al, 2022 (95)	• Describe antibiotic dispensing/ prescribing practices and underlying factors associated with these practices among community-based HCWs including pharmacists<br>• Mixed-Method approach using an adapted questionnaire<br>• 49 pharmacy managers/ clerks took part	• Encouragingly, 59.2% of pharmacists/clerks were aware of the term AMR<br>• However, participating pharmacists/clerks had a high likelihood of dispensing an antibiotic for a child with a sore throat (47.9%), diarrhoea lasting more than one day (43.8%), fever (21.7%) and a cough (26.5%)<br>• High likelihood also of dispensing antibiotics for adults with a sore throat (77.6%), burning sensation when urinating (43.8%), diarrhoea lasting more than one day (70.8%), fever (36.7%) and a cough (51.0%)<br>• In addition, 44.6% of all respondents (including other HCWs) incorrectly believing antibiotics could be used to treat viral infections and 49.2% that antibiotics could be used to cure colds and influenza<br>• Only 18.8% of pharmacists/ clerks were familiar with the AWaRe categories and guidelines
	Hashmi et al, 2023 (201)	• Assess perceptions of community pharmacists regarding their role, awareness, facilitators and barriers towards instigating effective AMS practices in Pakistan<br>• Descriptive cross-sectional study using validated questionnaire<br>• 386 community pharmacists took part (100% response rate)	• 57.3% had familiarity with the term AMS with 78.0% strongly agreeing that the practice of AMS in their setting will reduce inappropriate use of antibiotics and 58.0% agreeing that AMS practice will also reduce health-related costs<br>• 92.7% believed that real-time feedback would enhance AMS activities<br>• However, barriers to AMS activities included lack of suitable personnel (90.9%), time restrictions (91.2%), lack of training (90.9%), lack of standard operating guidelines (94.3%) and limited knowledge (90.2%)
	Mustafa et al, 2023 (96)	• Assess KAP of self-medication among HCWs including pharmacists during the COVID-19 pandemic<br>• Descriptive cross-sectional study using a questionnaire principally based on previous studies<br>• 1173 HCWs took part – 11.8% were pharmacists	• There was no significant difference in the knowledge scores regarding self-medication including concerns between physicians and pharmacists; however, both had better knowledge scores than other HCWs<br>• 66% of HCWs reported they self-medicated during the pandemic with the top three most commonly purchased medicines being antipyretics (100%), antibiotics (80.4%) and vitamins (59.9%)<br>• 42% of HCWs self-medicated to prevent catching the virus, 20.7% because they suspected they had COVID-19 symptoms and 27.9% self-medicated to treat their colds and influenza

	Nabeel et al., 2024 (202)	• Assess KAP of community pharmacists regarding dispensing antibiotics without prescription<br>• Cross-sectional study using a self-administered pre-tested questionnaire based on previous studies<br>• 359 pharmacists took part (91.4% response rate)	• Encouragingly, 83.0% and 81.9% respectively of participating pharmacists strongly agreed or agreed that dispensing antibiotics without a prescription was contributing to AMR and AMR has become a public health issue<br>• Common reasons for dispensing antibiotics included unwillingness of patients to visit physicians for perceived non-serious infections and perceived good knowledge of pharmacists regarding antibiotics<br>• In addition, 51.0% of participating pharmacists stated they continually educate patients about the importance of completing the full course when dispensing antibiotics<br>• However, concerns for conditions where antibiotics are being dispensed without a prescription. These included: cold and influenza (62.1%), diarrhoea (50.7%), earache (27.9%) and rhinitis (24.3%)
	Rafi et al., 2024 (203)	• Document and compare public and private sector availability of all essential antibiotics<br>• 103 essential antibiotics surveyed - comprising 51 Access, 29 Watch, 6 Reserve, and 17 anti-tuberculosis drugs	• Mean percentage regarding on-spot availability was 23.76% ± 5.19 (14-25%) for public facilities and 59.20% ± 4.45 (54-66%) for private sector retail pharmacies. The overall percentage of available essential antibiotics varied significantly (*p*** < 0.001) in public and private sector sampling sites.<br>• Only 2.91% of surveyed essential antibiotics were available at all survey sites, whereas 30.9% antibiotics were not available at any survey site.<br>• Unlike the Watch group where none was unavailable at any surveyed site except moxifloxacin, 11 antibiotics of the Access group were found physically not available at any survey site
**Palestine**	Al-Halawa et al., 2023 (204)	• Explore Palestinian pharmacists' KAP regarding AMR<br>• Descriptive cross-sectional survey using a validated self-administered questionnaire<br>• 152 pharmacists completed the questionnaire (91.01% response rate)	• Encouragingly, 92.1% of surveyed pharmacists agreed that inappropriate use of antibiotics could lead to ineffective treatment, 72% disagreed that antibiotics could be used to alleviate pain, 86.2% disagreed that patients should stop taking antibiotics when their symptoms improved, and 66.5% agreeing that keeping leftover antibiotics for use in another time was not good practice<br>• In addition, 50.6% strongly disagreed/ disagreed that prescribing broad-spectrum antibiotics is always better, 64.5% agreed/ strongly agreed that the wrong choice of antibiotics may lead a pathogen to lose its sensitivity towards specific antibiotics, 75.6% agreed/ strongly agreed that ABR is a serious global public health issue, and 86.9% that patient's personal choice of antibiotic use and overuse contributes to AMR<br>• 63.8% also agreed/ strongly agreed that it is obligatory for them to educate patients requesting antibiotics without a prescription that they may not need one – important as only 40.1% rarely/ never dispense antibiotics without a prescription following patients' requests<br>• However, only 17.1% disagreed that antibiotics should always be used to treat URTIs
**Sudan**	Abdelrahman Hussain et al., 2022 (205)	• Investigate community pharmacists' KAP and key factors towards purchasing antibiotics without a prescription<br>• Cross-sectional study online using a semi-structured questionnaire	• Encouragingly, 96.5% of participating pharmacists believed that inappropriate use of antibiotics increases ABR, 82% that antibiotics are overused in Sudan, with 79% aware of issues of cross resistance with antibiotics<br>• In addition, 75.8% stated they talk with patients when pertinent regarding whether they need antibiotics, with only 23.8% believing that pharmacists may

		• 1217 pharmacists completed the questionnaire	advise patients to stop taking antibiotics when symptoms improve and only 24.7% that antibiotics can be dispensed as prophylaxis for future infections<br>• However, 57.8% believed ABR is mainly a problem in hospitals and 51.7% that antibiotics are indicated to relieve inflammation. 59.2% will also dispense antibiotics without prescriptions if a patient requests one by name and 51.2% will halve the course of antibiotics dispensed if patients have insufficient funds<br>• Of equal concern is that 67.2% of participating pharmacists will dispense antibiotics without a prescription for tonsilitis, 54.6% for UTIs, 44.2% for sore throats, 31.1% for diarrhoea and 20.3% for common colds, with dispensing enhanced by the low economic status of patients (51.3%) and patient pressure (37.5%)
**Upper-Middle Income countries***			
**Iraq**	Alkadhimi et al., 2020 (206)	• Ascertain dispensing practice of antibiotics in community pharmacy in Iraq<br>• Qualitative study among 20 community pharmacists using a questionnaire based on previous studies	• All the pharmacists surveyed dispense antibiotics without a prescription for conditions including URTIs, UTIs and diarrhoea - exacerbated by patients requesting antibiotics by name. This is despite most pharmacists explaining that ABR occurs due to the misuse of antibiotics<br>• Most pharmacists also dispense antibiotics to their regular customers due to fears of losing them, with dispensing antibiotics saving time and cost of a physician's consultation. In addition, typically lack of medicines and the poor quality of care in public hospitals<br>• Of equal concern is that most of the pharmacists were unaware about the concept of AMS or guidelines to optimize the use of antibiotics
**Jordan**	Haddadin et al., 2019 (207)	• Assess patterns of dispensing antibiotics among community pharmacies<br>• Cross-sectional survey 12 pharmacists working in 7 pharmacies participated along with 434 customers/patients (98% response rate)	• Of the antibiotics dispensed with/ without a prescription only 31.5% and 24.6% respectively were given appropriate doses and duration<br>• Cephalosporins were the most commonly recommended/ dispensed antibiotics by pharmacists<br>• The most common reason for requesting antibiotics were RTIs (59.5%) – 19% for colds/ influenza
	Darwish et al., 2021 (208)	• Investigate community pharmacists' KAP towards antibiotic dispensing, ABR and AMS<br>• Cross-sectional survey using a structured, validated and piloted questionnaire<br>• 114 pharmacists took part (76% response rate)	• 83.3% pf participating pharmacists perceived AMR as a global problem.<br>• Encouragingly, 59.7% also stated they educate patients about issues of inap-propriate antibiotics use where concerns<br>• In addition, 44.7% agreed that implementing AMS and ASPs would result in better outcomes for patients through reducing inappropriate use of antibiotics
	Al-Taani et al., 2022 (209)	• Assess knowledge, opportunity, motivation and behaviour of pharmacists and their sources of information regarding antibiotic use and ABR in Jordan<br>• Online cross-sectional questionnaire based on an ECDC validated survey instrument<br>• 384 pharmacists completed the survey	• Encouragingly, 91.9% of participating pharmacists believed unnecessary use of antibiotics makes them ineffective, 89.8% that antibiotics are ineffective against viruses, 87.2% that antibiotics are ineffective against colds and influenza and 52.9% that ABR can spread from person to person<br>• In addition, 88.0% strongly agreed/ agreed with the statement regarding knowledge of ABR; 84.9% that they have appropriate knowledge how to use antibiotics in their current practice, 66.4% that they have good opportunities to provide prudent advice on antibiotics and 77.3% that they have a key role in helping to control ABR in the country

			• Of concern is that only 39.8% strongly agreed/ agreed that they have easy access to materials to provide advice on antibiotics and ABR to patients<br>• Alongside this, when respondents were unable to give advice/ lack of resources when they dispense antibiotics, 45.3% stated patients were not interested in the information, 37.0% that no resources were available to help such as continual education and 27.1% that there was insufficient time to give advice<br>• However, 92.7% of participating pharmacists believed any information received such as guidelines/ information from scientific societies had an impact on changing their practice in dispensing antibiotics
**Lebanon**	ZAHREDDINE et al., 2018 (210)	• Assess the knowledge of parents and community pharmacists regarding antibiotics and ABR in the paediatric population<br>• Cross-sectional study using a structured questionnaire<br>• 202 community pharmacists took part (72.1% response rate)	• 90.1% of participating pharmacists believed inappropriate parental behaviour were the major causes of antibiotic misuse along with inappropriate behaviour of physicians (72.8%) and pharmacists (59.4%)<br>• 57.9% believed that a child <2 years, with severe painful otalgia, and fever >39°C requires an antibiotic and 42.1% that a child with an intense and sudden onset sore pharyngitis throat and fever requires antibiotics<br>• 52% believed that a low antibiotic dose would enhance ABR with 37.1% believing higher doses would do the same<br>• Significantly, higher mean number of years of experience was associated with poorer knowledge about antibiotics
	Sabra et al., 2022 (211)	• Explored Lebanese pharmacists' KAP surrounding UTIs<br>• Cross-sectional study using a structured questionnaire<br>• 450 pharmacists took part	• The majority of participating pharmacists were able to state the main cause and symptoms of UTI, with 94% aware that bacteria is the main cause of UTIs<br>• More than 90% of pharmacists believed UTIs are serious and that being unhygienic leads to UTIs (93.6%)<br>• The majority of the pharmacists believed that drinking plenty of water (96%), taking antibiotics (93.8%) and consuming foods rich in Vitamin C (56.7%) helps with UTIs<br>• 95.1% of participating pharmacists reported educating patients about preventative measures to reduce UTIs, 90.4% about the most common causes of UTIs and 88.4% about potential risk factors for developing UTIs to reduce future antibiotic use
**Libya**	Al-Shami et al., 2023 (212)	• Evaluate practices and perceptions of community pharmacists towards antibiotic use and ABR and strategies to improve future appropriate use<br>• Cross-sectional study using an online self-administered questionnaire developed from the literature<br>• 114 pharmacists completed the questionnaire	• 74.6% of participating pharmacists agreed/ strongly agreed that ease of obtaining antibiotics from community pharmacies increases ABR, 86% that it is important to supply antibiotics only when needed, 70% that it is important to supply a full course of antibiotics even when the patient says this is too expensive and 59.7% strongly disagreed/disagreed that ABR is a problem in hospitals but not the community<br>• In addition, more than two-thirds strongly agreed/agreed that AMS will reduce inappropriate antibiotic use and reduce ABR<br>• However, 48.6% of participating pharmacists believed antibiotics gave a faster cure for sore throats than non-antibiotics, only 47.3% strongly disagreed/ disagreed that antibiotics cure a patient with diarrhoea more quickly than non-antibiotics and only 43% strongly disagreed/ disagreed that they supply antibiotics

			when patients request them specifically as they do not want them go to another pharmacy. In addition, 46.5% will supply a smaller amount if the patient cannot afford a full course<br>• Of concern is that 66.7% had no previous involvement in antibiotic awareness campaigns principally because they never heard of such campaigns (48.7%). However, > 80% strongly agreed/agreed that raising awareness of rational antibiotic use including AMR among pharmacy students and pharmacists as well as educating patients will improve future antibiotic use and reduce AMR

*World Bank Classification; ABR = Antibacterial Resistance; AMR: Antimicrobial Resistance; AWaRe = Access, Watch, Reserve antibiotics; FGD = Focus Group Discussions; HCP = Healthcare Professional; HCW = Healthcare Worker; PHC = Primary Healthcare; KAP: Knowledge, Attitude and Practice; URTI: Upper Respiratory Tract Infection

Table S7: Knowledge, Attitude and Practices, antibiotics, antimicrobial resistance and antimicrobial stewardship – WHO South East Asian Region Dispensers

Country	Author and Year	Objectives, Study Design and Population	Summary of the Findings
**Low Income countries***			
**Nepal**	Nepal et al., 2019 (213)	• Investigate the pattern of antibiotic dispensing in private pharmacies<br>• Exit interviews conducted (1537 patients)	• 38.4% of patients were dispensed at least one antibiotic, with the most commonly dispensed antibiotics being cefixime (16.9%) and third-generation cephalosporins (38.0%)<br>• For several conditions, antibiotics were the most commonly dispensed medicine – these included RTIs (93.3%), diarrhoea and dysentery (91.3%), fever (70.5%) and UTIs (57.9%)<br>• Patients <15 years were more likely than all other age groups to receive antibiotics
	Goswami et al., 2020 (214)	• Understand community pharmacy personnel surrounding antibiotic-dispensing and the relationship between this understanding and their characteristics<br>• Cross-sectional survey among 312 pharmacy personnel using a structured questionnaire developed from the literature and piloted	• Encouragingly, most participants considered that dispensing antibiotics without a valid prescription is a problem (76.9%) and not legal to do so (86.9%).<br>• However, 34.9% had dispensed antibiotics without prescription and 88.1% stated that if they did not sell antibiotics without a prescription patients would go elsewhere despite 73.1% believing inappropriate dispensing increases AMR and only 34.3% that antibiotics are effective in reducing pain and inflammation<br>• Understanding about the role of antibiotics was significantly associated with age (p<0.001), work experience (p<0.001) and qualifications (p=0.017)
	Jha et al., 2020 (215)	• Evaluate antibiotic dispensing practices among community pharmacies<br>• Cross sectional study using a structured questionnaire<br>• 78 community pharmacy personnel took part	• Antibiotics were dispensed without a prescription in 85.9% of pharmacies, with brand substitution seen among 55.1% of surveyed pharmacies. 11.5% of pharmacists replaced prescribed antibiotics with cheaper brands<br>• Advice regarding completing the course of antibiotics was provided in 75.6% of pharmacies and insufficient courses of antibiotics dispensed in 29.5%<br>• Azithromycin (22.2%) was the most dispensed antibiotic followed closely by amoxycillin (21.9%)

	Koju et al., 2020 (216)	• Identify promotional activities by pharmaceutical companies in community pharmacies and medicine shops and the affordability of selected antibiotics to patients with low wages<br>• Cross-sectional study among 34 community pharmacies and medicine shops	• Financial bonus, free samples, and brochures were the most popular promotional activities by pharmaceutical companies among community pharmacies and medicine shops<br>• Top selling antibiotics had a high number of promotional activities with amoxicillin having 42 promotional activities, azithromycin 29 activities and amoxicillin/ clavulanate17 promotional activities respectively<br>• However, almost all of the most popular antibiotics for URTIs were unaffordable for unskilled workers costing them more than a day's wage
	Acharya et al., 2021 (217)	• Assess the economic and social drivers of dispensing antibiotics without prescriptions among community pharmacies<br>• Mixed-methods approach including qualitative structured interviews and a cross-sectional survey among 111 pharmacy owners and managers	• Azithromycin and amoxicillin were the most commonly dispensed antibiotics<br>• Proportion of pharmacies 'most likely' or 'likely' dispense antibiotics without prescription to adult patients ranged from 36.9% (sore throat) to 67.6% (cough) and for pediatric patients from 62.2% (sore throat) to 80.2% (cough or diarrhea) with no consistent relationship between the likelihood of dispensing antibiotics and revenues, profits or the number of patients<br>• Overall, dispensing behavior was influenced by pressure from patients with respondents were more likely to dispense antibiotics when patients specifically asked for 'an antibiotic' with 68.5% ranking 'customer satisfaction' as the most important factor motivating them
	Adhikari et al., 2021 (218)	• Explore the characteristics of sales of antibiotics without a prescription, its drivers and implications among hospital clinicians, dispensers and patients<br>• Semi-structured interviews and FGDs involving 90 participants overall	• Dispensers were the main interface between the formal and informal healthcare sectors<br>• Dispensers were aware that antibiotics were sold with little interaction with patients, that empiric treatment was common, and often entailed inappropriate dosage and overtreatment with broad-spectrum antibiotics<br>• Dispensers were also aware that these practices contravened public health recommendations; however, they were responding to patients' demands and barriers that patients face when accessing care from PHCs. A lack of time, costs, accessibility and perceived quality led patients to seek assistance outside of PGCs<br>• Only a few dispensers and patients were aware of AMR and described AMR as a potential consequence of inappropriate and overuse of antimicrobials through purchasing antibiotics without a prescription
	Rijal et al., 2021 (110)	• Explore KAP of antibiotic prescriptions and uses across sectors<br>• Cross-sectional study using a previously validated WHO questionnaire and pre-tested<br>• 33 private drug sellers participated	• 87.9% of participating drug sellers had heard about ABR and only 18% thought that antibiotics could treat all sorts of diseases<br>• 51.5% believed that antibiotics could be used to treat bacterial diseases and oral thrush<br>• However, 23.3% believed antibiotics can be used to treat viral diseases
	Zheng et al, 2021 (112)	• Explore health care-seeking patterns and source of antibiotics for fevers, ARIs and diarrhoea among children<br>• Facilities included public and private clinics and pharmacies	• Overall, antibiotic use in rural areas increased over time with 2016 data demonstrating rural antibiotic consumption for ARIs and fever surpassing urban regions which is a concern<br>• Higher levels of maternal education were generally associated with increased antibiotic use, particularly for fever

		• 5457, 5054 and 4861 children were included in the 2006, 2011 and 2016 surveys respectively.	• From 2006 to 2011, the percentage of U5s with only a cough receiving antibiotics increased from 7% to 12%, and 75% of children who received antibiotics for diarrhoea did not meet the required indication<br>• Overall, adherence to WHO-recommended antibiotics decreased over time.
	Karki et al., 2022 (219)	• Assess antibiotic dispensing knowledge and practice among dispensing staff<br>• Quantitative cross-sectional study conducted among 220 dispensing staff (100% response rate) using a structured questionnaire	• 75.9% of dispensing staff dispensed antibiotics without a prescription, with 68.2% of those surveyed reducing the number of antibiotics when the patients were not able to pay for the medicine<br>• 45.9% of dispensing staff stated they followed guidelines or manuals while dispensing antibiotics. However, only 56.4% provided counselling services on drug interactions, adverse drug reactions and drug allergies to the patients<br>• Overall, 50.0% of dispensing staff had inadequate knowledge regarding antibiotics and 46.4% had poor antibiotics dispensing practices
	Shrestha et al., 2023 (220)	• Understand KAP of pharmacy employees regarding antimicrobial dispensing<br>• Cross-sectional survey using a structured piloted questionnaire on 801 pharmacy employees	• Whilst 87% of respondents agreed AMR to be a global public health threat with misuse/overuse of antimicrobials as the most common cause of AMR, 92% agreed that demand for non-prescription antimicrobials was common in the country<br>• Suspected RTIs was the most common reason for patients demanding non-prescription antimicrobials followed by GI diseases such as diarrhoea with azithromycin the most commonly sold antimicrobial (48%) followed by amoxycillin (22%)
	Marasini et al., 2024 (221)	• Explore KAP of antimicrobials and AMR among medicine dispensers and community members in Nepal<br>• Pre-tested semi-structured interview guides were used<br>• 16 In-depth interviews with medicine dispensers and 3 FGDs with community members	• Most participating dispensers described antimicrobials as antibacterial medicines used to suppress the growth of bacteria, with most dispensers describing the current use of antimicrobials as irrational<br>• In addition, perceived primary drivers of AMR included underuse of antibiotics and non-compliance with prescribed doses, with most dispensers believing their major roles were proper counselling and promoting the rational use of antimicrobials, i.e. dispensing only when necessary<br>• However, dispensers had varying knowledge regarding AMR and often supplied antimicrobials upon patient's specific requests as competition between them
**Low-Middle Income countries***			
**Bangladesh**	Darj et al., 2019 (222)	• Evaluate retail pharmacists; perception of AMR<br>• Qualitative study comprising in depth-interviews using a purposely develop topic guide<br>• 24 retail male pharmacists were recruited	• Participating pharmacists with a diploma/ certificate were more aware of AMR than untrained ones, with untrained pharmacists exhibited greater ignorance, misuse of antibiotics and generally poor understanding about AMR than trained ones<br>• Some pharmacists considered that AMR was not a matter of concern for them although others did. This is a concern as self-treatment with antibiotics is common in Bangladesh due to concerns with costs and time with patients thinking they recognized their infections and consequently wanted the same kind of previous medication<br>• Medication without prescriptions or with old prescriptions, inadequate supervision and easy availability of antibiotics, were all identified as risk factors for AMR that need addressing

	Matin et al., 2020 (223)	• Evaluate access and utilization of antibiotics in rural areas by investigating the perception and practices of drug dispensers<br>• Mixed method of qualitative and quantitative study comprising 16 in-depth interviews.<br>• 301 drug dispensers from both public and private sectors were also included	• Out of 301 drug dispensers surveyed, 51% had no formal authorization for selling antibiotics exacerbated by drug dispensers being able to run their business without legal challenges<br>• Of concern is that drug sellers surveyed often dispense antibiotics to cure fevers, colds and coughs, and acute watery diarrhoea, often recommend antibiotics such as azithromycin for fever and RTIs and regularly sell incomplete courses of antibiotics to patients in view of financial restrictions<br>• Identified factors to tackle concerns with antibiotics include addressing the lack of physicians and inadequate healthcare facilities as well as dispenser and patient knowledge regarding self-limiting infections
	Nahar et al., 2020 (113)	• Explore HCPs' understanding of the use and functions of antibiotics, awareness of AMR and perceived patient demand<br>• Qualitative study comprising in-depth interviews with 46 HCPs from both urban and rural areas including 27 drug sellers (semi-qualified and unqualified)<br>• Interview guide based on a review of the literature combined with input from the researchers.	• There was a considerable lack of awareness about the action of antibiotics among unqualified and semi-qualified participants – exemplified by (i) most unqualified providers thinking antibiotics start working by reducing fever and not having clear knowledge about the different generations of antibiotics; (ii) a number believing that antibiotics can prevent disease<br>• Overall, unqualified or semi-qualified participants had misconceptions about target organisms of antibiotics and the effectiveness of cheaper antibiotics<br>• Encouragingly, all participating HCPs showed concern about ABR with some unqualified and semi-qualified HCPs well informed about the term and causes of ABR. However, others were completely unaware of these and many HCPs thought ABR was a side-effect of antibiotics<br>• In addition, participating HCPs typically shifted the responsibility of preventing/ reducing ABR onto others
	Nizame et al., 2021 (224)	• Explore the awareness of relevant policies and guidelines among drug shop operators and customers and identify current dispensing practices, barriers and facilitators to adherence to policies on antibiotic prescribing and dispensing<br>• Formative research to explore contextual drivers of antibiotic use and promotion among registered physicians, drug shop staff and household members<br>• This involved workshops (*n* = 4) and in-depth interviews (*n* = 24) with drug shop operators	• 14 out of 47 drug shop operators who participated in the study had no health-related training. Overall, operators typically had no clear knowledge of different antibiotic generations as well as how and for what diseases antibiotics work<br>• They also did not typically dispense a full course of antibiotics in view of the financial circumstances of patients and/ or patients did not believe a full course was needed<br>• Drug shop operators were concerned that if they advise patients against taking antibiotics/ they insist on dispensing antibiotics only with a prescription - the customer may take their business elsewhere<br>• This is an issue since for minor illnesses including cold/ coughs and fever, patients typically go to drug shops for treatment in view of the scarcity of physicians
	Orubu et al., 2021 (225)	• Mapping antimicrobial supply chains in Bangladesh<br>• This includes the number of antibiotic manufacturers and licensed antibiotics among humans in ambulatory care	• There were 138 unique, or individual, licensed antimicrobials/anti-infectives by INN available as 1,763 products in Bangladesh, with 99.5% of all licensed antibiotics locally manufactured<br>• The most common antibiotic classes were the cephalosporins (44% of licensed antibiotics), followed by the penicillins (18%); quinolone/fluoroquinolones (15%) and macrolides (15%)<br>• 54% of the top 10 licensed antimicrobials belonged to the Watch category with only 39% in the Access category

	Samir et al., 2021 (114)	• Assess the prevalence and key factors associated with antibiotic use in children under five with febrile illness<br>• Secondary analysis of Bangladesh Demographic and Health Survey data<br>• 8421 children were surveyed<br>• Sources of antibiotic prescribing/ dispensing included public and private sector physicians/ unqualified providers as well as pharmacy personnel	• 33% of children had experienced fever in the preceding two weeks. Of these 9% also had symptoms of ARI and 14% also had diarrhoea<br>• 17% of surveyed children with a fever received antibiotics<br>• The highest use of antibiotics was in children under six months of age (25%)<br>• Children of parents who had completed secondary or higher education were more likely to receive antibiotics
	Unicomb et al., 2021 (226)	• Develop of social and behavioural change communication to increase the appropriate use of antibiotics<br>• Use formative research methods/ findings and intervention design workshops with key stakeholders including drug shop staff (13 participants) to select target behaviours to improve future antibiotic use	• Drug store operators often lacked knowledge about antibiotics and ABR. This is a concern especially since patients with minor illnesses typically seek treatment/ healthcare advice from drug store sellers in view of travel costs and distances to health care facilities<br>• Alongside this, limited activities by drug store operators to address current practices that antibiotics are typically stopped when symptoms disappear<br>• In addition, antibiotics were often purchased by proxies for ill household members with underage children and adolescents as able to purchase antibiotics from drug shops – exacerbated by drug shop staff regularly selling antibiotics without a government license
	Islam et al., 2022 (227)	• Explore antibiotic dispensing patterns in pharmacies according to the WHO AWaRe classification<br>• Structured questionnaires among both drug sellers and patients purchasing antibiotics (with or without a prescription)<br>• 128 drug sellers were interviewed alongside monitoring 2686 customers/ patients	• Out of 2686 customers interviewed, 21.6% (580) had purchased antibiotics - 523 had purchased one, 52 had purchased two and 5 had purchased three courses of antibiotics (totalling 642 courses)<br>• Watch antibiotics accounted for 53.6% of all antibiotic courses dispensed followed by Access (36.4%) and Reserve (10.0%) antibiotics<br>• 50.9% were purchased without a prescription, with dispensing of non-prescribed antibiotics higher in the Access group (59.4%) followed by the Watch (46.5%) and Reserve (43.8%) groups
	Ali et al, 2023 (228)	• Assessed the impact of training on Good Pharmacy Practice (GPP) among trained pharmacists vs. not receive GPP training<br>• Explore major challenges towards achieving GPP<br>• Semi-structured among 440 pharmacists (220 in each group)	• General dispensing behavior was better among trained vs. non-trained pharmacists regarding labeling of medicines (63.2% vs 53.4%, $p = 0.038$) and counseling customers (39.1% vs 28.6%, $p = 0.021$)<br>• Bad behavior of the customers (39.5%) and lack of GPP knowledge among surveyed pharmacists (28.6%) were recognized as challenges towards achieving GPP in Bangladesh including dispensing medicines such as antibiotics without a prescription
	Bepari et al., 2023 (115)	• Identify the risk factors for developing AMR by exploring KAP of local pharmacy shopkeepers and unqualified village medical practitioners.<br>• Population based cross-sectional study through structured interviews using a questionnaire based on previous studies	• Most participating shopkeepers correctly identified ABR refers to the bacterial loss of antibiotic sensitivity and most agreed they had a role in combating ABR<br>• However, 30% of participating pharmacy shopkeepers believed they had enough sources of information regarding antibiotics and only 19.48% of pharmacy shop keepers believed that antibiotics were ineffective against viral diseases reflected by 28% providing antibiotics to treat a cold or sore throat, with patient demand exacerbating overuse (51%)

		• 149 pharmacy shopkeepers and 247 unqualified local medical practitioners took part	• Overall, most pharmacy shopkeepers had poor knowledge of antibiotic use and AMR; however, 17% strongly agreed and 51% agreed that ABR is a global problem – although not a problem in their regular practice (58%)<br>• This compares with participating unqualified local medical practitioners who had higher scores regarding antibiotics and AMR than pharmacy shop keepers, with KAP scores slightly higher for unqualified village medical practitioners (median 72.38%) than for pharmacy shopkeepers (median 66.67%)
	Rousham et al., 2023 (229)	• Examined gender dimensions of antibiotic purchases and use of prescriptions in retail drug shops<br>• Conducted customer observations in 20 drug shops in rural and urban areas<br>• Overall 582 observations took place	• 31.6% of drug shop customers were women, higher (47.1%) in urban vs. rural areas (17.2%; $p < 0.001$)<br>• 74% of antibiotics dispensed were without a prescription with antibiotic purchases were more common in urban than rural shops (21.6% versus 12.2% of all transactions, $p = 0.003$).<br>• Prescriptions for antibiotics was more likely among women than men and more likely among urban compared to rural customers
	Al Masud et al., 2024 (230)	• Audit antibiotic dispensing patterns among community pharmacies and identifying factors influencing purchasing behaviour<br>• Cross-sectional survey involving 385 antibiotic customers and structured observations of 1000 pharmacy dispensing events	• Among 1000 observed medicine dispensing events, 25.9% were antibiotics with commonly purchased antibiotics including macrolides (22.8%), third- (20.8%) and second-generation cephalosporins (16.9%) with 73.5% of antibiotics from the Watch list and 23.1% Access<br>• 56.6% antibiotics were purchased without a prescription from drug-sellers and informal healthcare providers, primarily for non-severe conditions including URTIs (37.4%), fever (31.7%), uncomplicated skin infections (20%) and GI infections (11.2%), and urinary-tract infections (7.9%) After adjusting for confounders, sex, urban-rural locations, income, education, and the number of health-symptoms did not influence prescription likelihood
**Bhutan**	Tenzin et al., 2023 (231)	• Assess the KAP of competent personnel working in community pharmacies on antimicrobial use and AMR<br>• Cross-sectional survey using a questionnaire based on published studies<br>• 58 personnel took part in 55 pharmacies	• Encouragingly, 96.55% of participants were aware that inappropriate use of antimicrobials increases AMR and 91.38% that antibiotics should not be stopped soon after symptoms resolved.70.69% were also aware that antibiotics are not effective colds or influenza<br>• In addition, 98.28% believed AMR was a global problem, reducing slightly to 94.83% for Bhutan. 94.83% also believed community pharmacy personnel play an important role in tackling AMR<br>• Of concern is that community pharmacy personnel believed 84.48% of patients visiting community pharmacies had little or no knowledge regarding AMR
**India**	Nair et al., 2019 (118)	• Assess the KAP of formal prescribers (physicians - 96), nurses (96), informal prescribers (96) and pharmacy shopkeepers (96) with respect to antibiotic use<br>• Cross-sectional study using a validated questionnaire	• 95.9% of informal health providers, 98.9% of nurses, and 94.8% of pharmacy shopkeepers claimed their knowledge of antibiotics was important to them in their role even though none of these groups are legally permitted to prescribe/ dispense antibiotics independently<br>• Appreciable differences between the groups with 76.1% of physicians either strongly disagreeing or disagreeing with the statement that antibiotics were useful for viral infections. This compares to over 60% of nurses, over 70% of informal providers and over 80% of pharmacy shopkeepers who thought antibiotics are useful for viral infections

			• Having said this, over 88% of physicians reported prescribing antibiotics for viral infections, e.g. common colds or sore throat, in reality enhanced by patient demands and as a precaution against secondary infections – similarly 85% of informal providers for some of their patients<br>• Typically, all 4 groups disagreed/ strongly disagreed with stopping antibiotics early; however, all 4 groups frequently disbursed shorter, 3-day courses
	Nair et al., 2019 (119)	• Assessing the key drivers of antibiotic use among both formal prescribers (6), nurses (5), informal prescribers (5), pharmacy shopkeepers (5) and patients (7)<br>• Qualitative study involving in-depth interviews using an adapted validated questionnaire	• Many physicians are happy to prescribe antibiotics without any clinical indication – often seeing them as a vital part of care delivery - as a result of variable follow up/ questioning, lack of testing facilities, risk of secondary infections and often unhygienic living conditions<br>• Prescribing/ dispensing of antibiotics exacerbated by patient demand – with antibiotics seen as 'quick cures' – and lack of time with physicians typically seeing 80 – 100 patients/ day in PHCs<br>• Generally limited knowledge regarding AMR with some pharmacy shopkeepers believing AMR caused by bad weather with antibiotics often combined for a range of illnesses
	Chandran et al., 2022 (232)	• Obtain an understanding of the rationale behind selling antibiotics without a prescription from the pharmacists' perception<br>• 15 pharmacists were interviewed using a validated questionnaire	• Common reasons for purchasing antibiotics without a prescription were lack of time, avoiding costly physician fees and that the same antibiotic was prescribed each time<br>• The most common infection for self-purchasing was a common cold (86.7%)<br>• Of equal concern is that only very few patients purchased a full course of antibiotics either due to lack of money or they believed they would recover after a few doses<br>• All pharmacists were aware of ABR; however, considerable misinformation about the causes of drug resistance, e.g. 46.7% believed ABR was due to a lack of immunity
	Kumar et al, 2022 (233)	• Assess knowledge and attitude of community pharmacists regarding antibiotic use, ABR and dispensing antibiotics without prescriptions<br>• Questionnaire based cross sectional survey – statistically validated<br>• 75 community pharmacists completed survey	• Encouragingly, 41% of participating pharmacists agreed that ABR has become a major health issue, with 41% agreeing dispensing antibiotics without prescription increases ABR. In addition, 60% of participating pharmacists accepted the use of antibiotics for bacterial infections<br>• However, 65% of participating pharmacists stated that antibiotics are useful for viral infections and only 9% disagreed with the comment that antibiotics are indicated to reduce any kind of pain or inflammation<br>• In addition, only 32% of pharmacists agreed that the misuse of antibiotics can lead to a loss of sensitivity of an antibiotic to a specific pathogen, with 58% of pharmacists disagreeing when asked whether a full course of antibiotics can be stopped before completion if symptoms improved<br>• Overall, poor knowledge negatively impacted on attitudes and practice towards antibiotics and ABR
	Dharanindra et al., 2023 (234)	• Assess antibiotic-dispensing patterns as well as AMR awareness among community pharmacists<br>• Cross-sectional observational study using a predesigned questionnaire	• Of concern is that 78% of pharmacist/ dispensers surveyed dispense antibiotics OTC for common ailments including common colds, coughs, sore throats, nasal congestions, diarrhoea, fever and UTIs with azithromycin (54.1% of all antibiotics) the most common antibiotic dispensed OTC

		• 389 pharmacies took part (91.1% response rate)	• Alongside this among the 303 pharmacies surveyed practicing OTC dispensing of antibiotics, 82.5% were unaware of emerging trends of AMR with only 17.5% partially aware of AMR. This compares with 57% of pharmacies surveyed who only dispense antibiotics with a prescription<br>• In addition, most pharmacists dispensing antibiotics OTC were advising inappropriate courses with only a few dispensers advising patients regarding appropriate antibiotic regimens
**Indonesia**	Ferdiana et al., 2021 (235)	• Examine antibiotic dispensing practices and associated drivers<br>• 59 in-depth interviews with a structured questionnaire including 31 personnel at pharmacies drug stores and 28 patients	• A common pattern of antibiotics dispensed without a prescription was following a direct request from patients; less common were pharmacists recommending an antibiotic often based on a brief assessment of the patient<br>• High rates exacerbated by limited knowledge of antibiotics among patients and dispensing personnel, with patients requesting antibiotics even for muscle pain alongside colds, inflammation and coughs. In addition, weak enforcement of any regulations<br>• Rarely were patients referred to health facilities – typically only for severe infections
	Wulandari et al, 2021 (236)	• Determine the extent and determinants of inappropriate dispensing of antibiotics by licensed private drug retail outlets<br>• Simulated patients (SPs) visiting 166 drug outlets presenting with either as a parent of a child at home with diarrhoea, an adult with presumptive TB or an adult with a URTI	• Antibiotic dispensing without prescription was common in 69% of SP visits - more likely in standalone pharmacies and pharmacies attached to clinics compared with drug stores<br>• Dispensing of antibiotics without prescriptions was also more likely for TB and URTI SPs than those with acute diarrhoea, with inappropriate antibiotic dispensing driven by strong patient demand for antibiotics, competition between different types of drug outlets and drug owners pushing their staff to sell medicines
**Sri Lanka**	Zawahir et al., 2019 (237)	• Evaluate community pharmacy staff's knowledge about antibiotics and identify possible factors impacting their dispensing behaviour<br>• Cross-sectional survey using a validated questionnaire<br>• 265 pharmacists/ assistants completed the questionnaires (72% response rate)	• Encouragingly only 10.2% of surveyed community pharmacy staff said they would dispense an antibiotic without a prescription for a minor viral infection (higher for pharmacists) with lower rates (4.2% overall) for a child with a viral infection. In actuality, 13.6% of community staff had dispensed an antibiotic for a sore throat in the last week, 15.8% for a cold/ cough, 8.7% for a UTI and 10.2% for patients with diarrhoea<br>• However, 44.2% of those surveyed would dispense an antibiotic without a prescription if they knew the patient (higher for assistants)<br>• Overall, pharmacists with higher ABR knowledge were less likely to dispense antibiotics without a prescription for viral infections
	Zawahir et al., 2021 (238)	• Assess community pharmacy staff's KAP towards supplying antibiotics for common colds and cough, sore throats, diarrhoea and UTIs<br>• Cross-sectional national survey using a structured questionnaire based on the literature<br>• 265 responses included in the analysis (72% response rate)	• 30.2% of surveyed staff had supplied antibiotics without a prescription for common infections including common colds (15.8%), sore throats (13.6%) and diarrhoea (10.2%)<br>• Pharmacists were less likely to supply antibiotics than non-pharmacists, with pharmacy staff with more positive beliefs about their competency more likely to supply antibiotics without a prescription for common colds and UTIs than others<br>• Of concern was that pharmacy staff who believed in the effectiveness of antibiotics against common infections were more likely to supply antibiotics for these infections

*World Bank Classification; ABR = Antibacterial Resistance; AMR: Antimicrobial Resistance; AWaRe = Access, Watch, Reserve antibiotics; FGD = Focus Group Discussions; HCP = Healthcare Professional; HCW = Healthcare Worker; PHC = Primary Healthcare; KAP: Knowledge, Attitude and Practice; URTI: Upper Respiratory Tract Infection

Table S8: Knowledge, Attitude and Practices, antibiotics, antimicrobial resistance and antimicrobial stewardship –WHO Western Pacific Region Dispensers

Country	Author and Year	Objectives, Study Design and Population	Summary of the Findings
**Low-Middle Income countries***			
**Cambodia**	Suy et al., 2019 (239)	• Investigated factors that influence community decisions on which HCPs to purchase medicines from focusing on medicine sellers and comparing different HCPs' knowledge of antibiotic use<br>• FDGs and structured interviews with 21 HCPs including 5 pharmacists, 7 medicine sellers and 5 informal medicine sellers	• Medicines purchased from sellers as typically more convenient than healthcare clinics and typically do not need to wait<br>• Overall, concerns with the level of knowledge of participants especially among informal medicine sellers, e.g. taken when sick, for a cough or diarrhoea, and tablets also broken up and placed on wounds. In addition, informal sellers more likely to sell incomplete antibiotic courses following demands from patients about available finances at the time<br>• Trained pharmacists more conservative and knowledgeable regarding antibiotics
**Lao PDR**	Sychareun et al., 2021 (133)	• Assess KAP of HCPs including physicians regarding antibiotic use and ABR related to pregnancy, childbirth and infants<br>• Mixed-methods study using a pre-tested structured questionnaire<br>• 217 HCPs took part including 34 pharmacists/ assistants and 11 drug sellers	• Encouragingly 94% of participating HCPs had heard of ABR, 91% that extensive use of antibiotics increases the risk of ABR, 89% that unnecessary use of antibiotics can make them ineffective and 76% that antibiotic courses should not be interrupted even when symptoms of sick patients are improving.<br>• Overall, 80% agreed that ABR is an appreciable problem in their practice with 67% believing ABR is a problem in Laos. In addition, 100% of participants also correctly classified ampicillin, amoxicillin, and penicillin as antibiotics<br>• However, only 68% stated cortisone is not an antibiotic. 39% would also recommend an antibiotic to an adult with a runny nose, cough or fever to hasten recovery – reflected by antibiotics being prescribed/dispensed for fever (69%), sore throats (63%) and coughs (51%)
**Vietnam**	Nhi et al., 2018 (240)	• Assess antimicrobial access and usage in for childhood diarrhea in an urban setting using a simulated patient study<br>• Interviews among caregivers (396) using a structured questionnaire	• 8% and 22% of outlets sold antimicrobials for pediatric watery and mucoid diarrhea, respectively.<br>• 59% of caregivers reported their children had received medication in the last month - 47% of which was an antimicrobial<br>• Concerns with knowledge among surveyed caregivers with 46% believing antimicrobials can be used to treat coughs, 35% fever, 30% colds, 21% headaches and19% diarrhea (19%), with 85% stating they had antimicrobials within the last 30 days prior to the interview
	Nguyen et al., 2019 (241)	• Understand key issues regarding access and use of antibiotic among drug sellers and patients	• Of concern is that a high percentage of participating drug sellers do sell antibiotics without a prescription (94%) – and it is common for customers to request specific antibiotics based on previous experiences<br>• Common illnesses that antibiotics were sold for included sore throats, cold, and inflammation – 81% of suppliers

		• In-depth interviews with drug suppliers FGDs with community members using structured guides<br>• 16 in-depth interviews with drug suppliers	• Suppliers often sold incomplete courses – 94%; with 69% believing that patients do not complete their courses of antibiotics<br>• In addition – 94% of suppliers had unsure knowledge regarding antibiotics and ABR<br>• Common misperceptions among suppliers included effectiveness of antibiotics for viral infections and URTIs. Alongside this, whilst all suppliers had heard about ABR they were typically unsure what it is and how this develops
	Nguyen et al., 2020 (242)	• Review community-level utilisation of antibiotics in rural Vietnam 1404 antibiotic encounters were included in this study - of which 1342 were for human use	• Antibiotics were purchased without prescription in 57.6% of cases<br>• Antibiotics were dispensed for a number of infectious diseases including coughs (53.9% of encounters where an antibiotic was dispensed), sore throats (48.0% of encounters), fever (33.3% of encounters) and runny noses (28.2% of encounters)<br>• The Access group of antibiotics constituted 59.0% of all antibiotics dispensed and the Watch group 39.3%<br>• Factors associated with a higher likelihood of a Watch antibiotic being dispensed were a private pharmacy, a non-prescription sale or a child
**Upper-Middle Income countries***			
**China**	Hyat et al., 2019 (243)	• Investigate the perspective of pharmacists on community-based ASPs in China<br>• Multicentre cross-sectional study<br>• 416 pharmacists took part (87.4% response rate)	• Encouragingly 74.0% of respondents believed ASPs are vital to improving patient care<br>• 34.1% always ask patients about their knowledge relating to antimicrobials when dispensing these<br>• In addition, 30.5% of respondents always or often collaborated with other HCPs to improve patient care
	Feng et al, 2021 (244)	• Investigate the KAP of pharmacy staff toward antimicrobials and ASPs<br>• Cross-sectional survey using a questionnaire developed from the literature and validated<br>• 394 completed questionnaires (98.5% response rate)	• Encouragingly, 91.9% of participants knew that common colds and influenza are caused by viruses, and 93.4% knew that antimicrobials could not cure all disease.<br>• 99.5% of participants also knew that antimicrobials are used to treat bacterial infection.<br>• 73.9% thought that ASPs should be incorporated at the community pharmacy as ASPs reduce the problem of AMR.<br>• In addition, 40.6% strongly agreed that adequate training should be provided to community pharmacists on antimicrobial use<br>• However, concerns with actual practices regarding ASPs were poor. Only 15.7% of pharmacy staff reported often providing medication consultations to patients who bought antimicrobials and only 69.5% of participants stating they often told patients about the adverse effects of using antimicrobials.<br>• Participants' age, level of education, occupation and experience showed a statistically significant difference with the median score of practices toward ASPs ($p < 0.05$).<br>• A pharmacy of independent type ($p < 0.001$) was a key factor associated with high levels of antimicrobial knowledge among staff because of pharmacy-sponsored training programs

	Zhang et al., 2022 (245)	• Analysis published studies to examine the impact of 9 policies on antibiotic sales in retail pharmacies in China and how tensions between ‘excess’ and ‘access’ are managed<br>• Review of policy documents (25) and 9 studies relating to controlling antibiotic sales in retail pharmacies in China.	• Inappropriate sales of antibiotics are still common in retail pharmacies, which can be linked to the lack of consistency and enforcement of published policies, the profit-driven nature of retail pharmacies, and the displacement of the demand for antibiotics from clinical into less regulated settings<br>• Additional measures are needed to reduce inappropriate dispensing of antibiotics in pharmacies in China with increasing utilisation of antibiotics now coming from retail pharmacies in China

*World Bank Classification; ABR = Antibacterial Resistance; AMR: Antimicrobial Resistance; ASPs = Antimicrobial Stewardship Programmes; AWaRe = Access, Watch, Reserve antibiotics; FGD = Focus Group Discussions; HCP = Healthcare Professional; HCW = Healthcare Worker; PHC = Primary Healthcare; KAP: Knowledge, Attitude and Practice; URTI: Upper Respiratory Tract Infection

Table S9: Knowledge, Attitude and Practices, antibiotics, antimicrobial resistance and antimicrobial stewardship – WHO African Region Students

Country	Author and Year	Objectives, Study Design and Population	Summary of the Findings
**African countries**	Xu et al., 2019 (246)	• Literature search to assess the extent of self-medication with antibiotics among university students in low and middle-income countries including African countries<br>• Random-effects model was applied to calculate the pooled effect size	• The practice of self-medication with antibiotics was widespread among university students in LMICs and frequently associated with inappropriate use<br>• Africa had the highest pooled prevalence of self-medication among university students at 55.30% vs. Asia at 43.8% and South America at 38.3%<br>• The highest prevalence was in the Congo (90.7%) vs. Brazil at 11.1%
**Low Income countries***			
**Ethiopia**	Seid et al., 2018 (247)	• Assess knowledge and attitude of para-medic students regarding AMR<br>• Cross-sectional study using a structured questionnaire based on a review of the literature<br>• 323 paramedic students took part (90.0% response rate)	• Overall, 55% of participating students had a poor level of knowledge of AMR with 33.1% moderate level of knowledge<br>• However, 98.2% believed that inappropriate use of antibiotics puts their patients at risk and more than 50% knew the causes of AMR and consequence of overuse of antibiotics<br>• In addition, more than 70.0% of students agreed positively with all attitude questions, 87.3% about the consequences of AMR and 70.6% that AMR is a major problem globally and in Ethiopia that needs urgent addressing
	Fetensa et al., 2020 (248)	• Assess knowledge and attitudes towards AMR among health science students<br>• Cross-sectional study design using a semi-structured tool taken from a previous study<br>• 232 students took part (93.6% response rate)	• 84.9% of participating students agreed that penicillin or amoxicillin were antibiotics, with 73.3% disagreeing that aspirin is an antibiotic<br>• Encouragingly 86.6% of students also knew that irrational consumption of antimicrobials increases<br>• However, 32.4% agreed or strongly agreed that it is acceptable to discontinue antimicrobials when patients are feeling better,<br>• In addition, 60.3% believed it acceptable to take antibiotics for cold and 53.9% for a fever. 31.9% also believed it acceptable to utilize left over antibiotics

**Mali**	Chen et al., 2021 (249)	• Primarily to assess antibiotic knowledge and utilization among medical students in the country<br>• Cross-sectional survey using a questionnaire derived from other LMICs<br>• 446 students completed the questionnaire (89.2% response rate)	• Encouragingly, 61.7% of students recognized that the overuse of antibiotics would lead to difficulties in treating bacterial infections and 57.6% agreed that inappropriate use of antibiotics would increase ABR resulting in fewer available antibiotics<br>• However, only a minority of participating students correctly answered that antibiotics do not work for viral infections (39.9%), influenza (32.1%), sore throats (19.7%) and common diarrhoea<br>• In addition, 72% of students falsely believed that newer antibiotics were always more effective than older ones
**Malawi**	Kamoto et al., 2020 (250)	• Determine knowledge, attitude and perception on antimicrobial use and AMR among final year medical students<br>• Cross-sectional study using a self-administered questionnaire based on previous studies<br>• 74 students took part (95% response rate)	• Encouragingly over 88% of the respondents answered more than half of the knowledge questions correctly<br>• In addition, only 13.9% of participants believed AMR was not a problem nationally, with 69.4%believing antibiotics are being over used in the country, with the better use of antibiotics helping to reduce AMR<br>• Variable knowledge though with 98.6% not recommending antibiotics for children with diarrhoea; however, only 22.2% would not prescribe antibiotics in children with purulent rhinitis and a sore throat
**Rwanda**	Tuyishimire et al., 2019 (251)	• Assess self-medication practices with antibiotics among students at the University of Rwanda and the rationale including students from the College of Medicine and Health Sciences<br>• Structured survey using a structured questionnaire<br>• 570 students participated	• Self-medication was more frequent among female versus male student participants<br>• Principal reasons for self-medication were that the students believed their illness not serious (50.7%) and assumed medical knowledge (14.5%)<br>• The principal infections treated with self-purchased antibiotics were common cold/influenza/cough (47.8%), sore throat (14.5) and diarrhoea (13.4%)
	Nisabwe et al., 2020 (252)	• Appraise attitudes and knowledge of AMR among healthcare students<br>• Cross-sectional study using a questionnaire adapted from previous studies<br>• 282 medical, dental and pharmacy students took part	• 94.3% of participating students responded correctly than penicillin and amoxycillin were antibiotics with only16.6% stating that aspirin is an antibiotic and only 5.7% that paracetamol is an antibiotic<br>• In addition, only 18.8% agreed/ strongly agreed that antibiotics can treat viral infections such as influenza and 21.8% that antibiotics are indicated to reduce any kind of pain or inflammation<br>• Encouragingly as well, 98.7% had heard about AMR with 78.6% discussing AMR during their course. In addition, 95.2% of students believed that inappropriate use of antimicrobials increases AMR<br>• However, only 14.8% knew what AMS means
**Tanzania**	Chuwa et al., 2021 (253)	• Assess the prevalence and factors associated with self-medication with antibiotics among university students<br>• Cross-sectional study utilizing a self-administered questionnaire<br>• 374 students took part (100% response rate) – 187 medical and 187 non-medical students<br>•	• Prevalence of self-medication with antibiotics overall was 57% - lower at 51% among medical students<br>• The commonest reasons of self-medication reported to be emergency illness (38.77%) and delaying of hospital services (24.33%)<br>• Common diseases for self-medication with antibiotics included (n=331) – headache - 31.02%, malaria – 15.24%, coughs – 10.96%, fever – 10.43% and diarrhoea – 5.61%<br>• Most common antibiotics used for self-medication were amoxicillin - 32.08%, and metronidazole – 8.82%

	Lubwama et al., 2021 (254)	• Evaluate the KAP of final year medical and pharmacy students on antimicrobial use and AMR at universities including Tanzania as well as assess how prepared they are to use antibiotics appropriately<br>• Cross-sectional survey was undertaken with a self-administered questionnaire<br>• 328 students took part including 178 from Tanzania	• Only 36.6% of final year students had good overall total knowledge – greater in students from Kenya (72%) vs. Uganda (40%) or Tanzania (20.2%)<br>• In addition, 72.9% of participating students had good a good attitude and perception on antibiotic use, highest in Uganda<br>• However, only 44.8% of students had good knowledge about AMR and only 17.1% had good knowledge about antibiotic use in various clinical scenarios<br>• Having said this, 97.6% knew that inappropriate use of antibiotics can lead to ABR and amoxycillin is an antibiotic. In addition, 97.6% disagreed that paracetamol and aspirin are antibiotics<br>• Of concern is that 29.9% wrongly agreed that prescribing broad spectrum antibiotics is always better even when narrower spectrum antibiotics are available and effective. In addition, whilst 68.2% of students reported knowing what AMR means only 26.8% of students were aware of the meaning of AMS
	Shitindi et al., 2023 (255)	• Compare KAP and factors influencing self-medication with antibiotics among medical and non-medical students<br>• Cross-sectional study using self-administered semi-structured questionnaires which were piloted<br>• Overall, 829 participants (89.2% response rate) - 48.2% medical students	• Prevalence of self-medication among medical students was 49.1% and 59.2% among non-medical students<br>• The main factors influencing self-medication were the availability of antibiotics without a prescription, easy access to pharmacies, and a lack of knowledge about the risks of SMA.<br>• Medical students were 1.6 times more likely to self-medicate with antibiotics than non-medical students (AOR: 1.6; 95%CI: 1.2–2.3, p=0.004)<br>• Most common illnesses for self-medicating with antibiotics were diarrhoea (20.8% - higher prevalence among non-medical students at 24.8% vs. medical students - 15.7%), UTIs (12.9%), sore throats (12.0%) and tonsillitis (10.4%)
**Uganda**	Lubwama et al., 2021 (254)	• Evaluate the KAP of final year medical and pharmacy students on antimicrobial use and AMR at universities including Uganda as well as assess how prepared they are to use antibiotics appropriately<br>• Cross-sectional survey was undertaken with a self-administered questionnaire<br>• 328 students took part including 75 from Uganda	• Only 36.6% of final year students had good overall total knowledge, greater among those from Kenya (72%) vs. Uganda (40%) or Tanzania (20.2%)<br>• In addition, 72.9% of participating students had good a good attitude and perception on antibiotic use, highest in Uganda<br>• However, only 44.8% of students had good knowledge about AMR and only 17.1% had good knowledge about antibiotic use in various clinical scenarios<br>• Having said this, 97.6% knew that inappropriate use of antibiotics can lead to ABR and amoxycillin is an antibiotic. In addition, 97.6% disagreed that paracetamol and aspirin are antibiotics<br>• Of concern is that 29.9% wrongly agreed that prescribing broad spectrum antibiotics is always better even when narrower spectrum antibiotics are available and effective. In addition, whilst 68.2% of students reported knowing what AMR means only 26.8% of students were aware of the meaning of AMS

	Kanyike et al., 2022 (256)	• Determine knowledge and perceptions of healthcare students towards AMR and confidence in prescribing antimicrobials<br>• Cross sectional survey using a data collection tool developed from similar validated<br>• questionnaires<br>• 681 students participated (55% response rate)	• 87.5% of surveyed students had sufficient knowledge about AMR - higher among female versus male students as well as e.g. fourth versus third year students<br>• 54.5% agreed that antimicrobials were being overused and 46.7% that poor infection control practices by HCPs causes AMR<br>• 66.9% of surveyed students stated they were confident with making an accurate diagnosis of infection/ sepsis and 71.1% stated they were confident in choosing the correct antimicrobial to prescribed<br>• However, 97.5% agreed they needed more training on antimicrobial selection, with 66.2% also wishing a separate course on AMR to improve their knowledge – similar to Kasujja et al (2024) advocating a multi-faceted approach to advance student knowledge of antibiotics and AMR (257)
	Nakato et al., 2023 (258)	• Determine the prevalence and factors associated with self-medication with antibiotics among undergraduate healthcare students<br>• Cross-sectional survey using a self-administered questionnaire based on the literature<br>• 326 students participated	• 93.8% of students stated that they self-medicate with antibiotics despite 72.1% stating they believed this was not an acceptable practice<br>• Amoxicillin (65% of responder students) and metronidazole (64.1%) were the most common self-medicated antibiotics among students<br>• Most common conditions for self-medication included diarrhoea (51.3%), peptic ulcers (35.3%) and fever (27%)<br>• Reasons for self-medication/ purchasing of antibiotics included the illness was seen as minor (55.2%), prior use of the same antibiotic (52.5%)/ perceived knowledge of the right antibiotic for their condition (44.2%), lack of services in the hospital (33.1%), high costs of consultations (25.8%) and self-medication saves time (29.1%)
**Zambia**	Zulu et al., 2020 (259)	• Assess KAP regarding ABR among undergraduate medical students at the University of Zambia<br>• Cross-sectional study using a structured questionnaire<br>• 260 undergraduate medical students participated.	• 87.3% of participating medical students had good knowledge on antibiotic use and AMR, e.g. 99.2% believed antibiotics can cure bacterial infections and 93.5% that antibiotics cannot cure viral infections<br>• In addition, 97.3% had heard of ABR and 93.1% believed that the frequent use of antibiotics can lead to ABR and reduce their effectiveness of treat-ment<br>• Overall, 96.9% had positive attitudes and 75% had good practices towards AMR<br>• However, there was a significant difference between the year of study and the level of knowledge, with final year medical students having higher levels of knowledge and attitude but lower levels of practice compared to other years
	Mudenda et al., 2023 (260)	• Assess non-health professional students' KAP towards antimicrobial use and AMR<br>• Cross-sectional study using a structured questionnaire adapted from previous studies<br>• 433 undergraduate students participated	• 55.2%, 63.5% and 45% of participating students had moderate KAP scores regarding antibiotic utilisation and AMR, with 76.7% self-medicating with an-tibiotics<br>• Students who were studying Engineering and Mining fields were more likely to have good knowledge of AMR compared with those in Social Sciences.<br>• Students who were in their fourth and fifth years were also more likely to have positive attitudes towards antibiotic utilisation and AMR<br>• Students who practised self-medication were less likely to have good self-reported practices towards AMR

	Nowbuth et al., 2023 (261)	• Assess antimicrobial use and resistance knowledge, attitude and perceived quality of education relating to AMR in Zambian medical schools<br>• Cross-sectional anonymous survey with the questionnaire based on previous studies and piloted<br>• 180 students took part	• Encouragingly, 91% of participating students thought antibiotics were currently overused in Zambia, and 88% thought AMR was a problem in Zambia.<br>• However, only 47% believed they were adequately trained on antibiotic prescribing, and only 43% felt confident in choosing the correct antibiotic for specific infections<br>• In addition, only 2% felt prepared interpreting antibiograms, only 3% were adequately trained to de-escalate to narrow-spectrum antibiotics and only 6% knew how to transition from IV to oral antibiotics. Alongside this, only 14% understood the spectrum of activity of prescribed antibiotics
**Lower-Middle Income countries***			
**Ghana**	Owusu-Ofori et al., 2021 (262)	• Determine healthcare students' self-medication practices and attitudes regarding AMR<br>• Cross-sectional study using a pretested questionnaire<br>• 264 students participated (94.3% response rate)	• 95.5% of students knew about antibiotics with 56.2% purchasing antibiotics without a prescription<br>• Encouragingly, 94.1% of students correctly answered that antibiotics can cure bacterial infections, with 66.9% correctly stating that antibiotics cannot cure viral infections<br>• Knowledge of ABR was known among 73.0% of participating students, with 69.7% agreeing that frequent use of antibiotic decrease their efficacy<br>• However, 63.4% used antibiotics to treat sore throats, 47.6% to treat common colds and 48.2% to treat diarrhoea
	Amponsah et al., 2022 (263)	• Investigate relevant aspects of self-medication among pharmacy and non-pharmacy students<br>• Cross-sectional study using a structured questionnaire based on previous studies<br>• 337 students (163 pharmacy and 174 nonpharmacy) participated (95% response rate)	• Whilst self-medication was common among students (55.2% among participating pharmacy students and 51.1% among non-pharmacy students), self-medication with antibiotics was appreciably lower<br>• Overall - self-medication with antibiotics was 10.6% among non-pharmacy students vs. 6.1% among pharmacy students<br>• Reasons for self-medication included long queues at hospitals, fast relief of illness, previous experience with the same medicine and illness not severe enough to warrant a physician visit
	Sefah et al., 2022 (264)	• Assess healthcare student's knowledge on antibiotic use, AMR and AMS<br>• Cross-sectional survey using a structured questionnaire based on the literature<br>• 160 students participated (100% response rate)	• Good knowledge of antibiotic use, AMR, and AMS was associated with the study course ($p = 0.001$) as well as the number of years of study ($p < 0.001$)<br>• Overall, healthcare students' knowledge on antibiotics was approximately six times more likely to be good knowledge if they were in their fifth year as opposed to earlier years
**Kenya**	Lubwama et al., 2021 (254)	• Evaluate the KAP of final year medical and pharmacy students on antimicrobial use and AMR at universities including Tanzania as well as assess how prepared they are to use antibiotics appropriately<br>• Cross-sectional survey was undertaken with a self-administered questionnaire<br>• 328 students took part including 75 from Kenya	• Only 36.6% of final year students had good overall total knowledge – greater among those from Kenya (72%) vs. Uganda (40%) or Tanzania (20.2%)<br>• In addition, 72.9% of participating students had good a good attitude and perception on antibiotic use, highest in Uganda<br>• However, only 44.8% of students had good knowledge about AMR and only 17.1% had good knowledge about antibiotic use in various clinical scenarios

			• Having said this, 97.6% knew that inappropriate use of antibiotics can lead to ABR and amoxycillin is an antibiotic. In addition, 97.6% disagreed that paracetamol and aspirin are antibiotics<br>• Of concern is that 29.9% wrongly agreed that prescribing broad spectrum antibiotics is always better even when narrower spectrum antibiotics are available and effective. In addition, whilst 68.2% of students reported knowing what AMR means only 26.8% of students were aware of the meaning of AMS
**Nigeria**	Ajibola et al., 2018 (265)	• Evaluate self-medication with antibiotics and knowledge of AMR among community members and undergraduate students<br>• Cross-sectional study using a structured pre-tested questionnaire<br>• 1230 people took part (84.8% response rate) – 872 community members and 358 students	• Weekly usage of self-medication with antibiotics was common – more common among undergraduate students (43%) than community members (26%)<br>• Prescription of antibiotics for use by a clinician, nurse, or pharmacist was 33.5%, 29%, and 25% respectively among undergraduate students vs. 57%, 20.4%, and 15.5% among the community members<br>• Antibiotics more likely to be purchased in patent medicine stores (40%) among students vs. local chemists/ pharmacies (48.4%) among community members<br>• Common conditions for self-medication with antibiotics among students were malaria (14.5%), typhoid (13.1%), stomach pains (12.7%) and diarrhoea (11.9%) and among community members dysentery/ diarrhoea (19%), infection (17%), typhoid (13%) and ear and throat pain (3.2%)<br>• Principal reasons for self-medication among undergraduate students and community members were long delays in the hospital (46% and 35%), cheaper not to go to hospital (26% and 19%) and the distance to the hospital (15% and 21%)
	Ayepola et al., 2018 (266)	• Understanding the levels of awareness regarding antibiotics among university students<br>• Structured questionnaire used and administered to 355 students	• Encouragingly, 75.2% stated they adhere to the dosage guidelines, 73.3% that it is important to complete the full course and 74.8% that improper use of antibiotics can be harmful<br>• However, 60.6% admitted taking antibiotics in the past 6 months, 64.5% that they take antibiotics without a prescription (usually from parents, guardians and pharmacists) and 56.9% that they keep antibiotics for future use. Most used antibiotics were ampicillin and penicillin
	Okedo-Alex et al., 2019 (267)	• Assess knowledge regarding antibiotic use and ABR among medical school students<br>• Cross-sectional study using a structured questionnaire conducted among 184 medical students (100% response rate)	• Encouragingly, 98.4% of participating students knew that AMR was an important global public health issue, with 96.2% correctly linking indiscriminate use of antimicrobials with the development of AMR. In addition, 96.7% believed that ineffective treatment with antibiotics can occur due to their indiscriminate use<br>• However, 39.1% did not know that common colds and influenza were not due to bacteria, i.e. viral in origin; 36.6% sometimes and 13.1% usually stopped taking their antibiotics when they felt better; and 37.2% of respondents never discarded leftover antibiotics and 35.5% sometimes<br>• In addition, 87.0% stated they wanted more education on antimicrobial use and AMR

	Augie et al., 2021 (268)	• Assess the knowledge and perceptions medical students about ABR and antibiotic use<br>• Cross-sectional survey using an electronic questionnaire developed from a previous study with four main categories on AMS<br>• 276 students participated - 104 from South Africa, 172 from Nigeria (overall 50.4% response rate)	• Encouraging 94.8% of Nigerian students surveyed agreed that antibiotics are overused in their country.<br>• However, South African students scored an average of 62.4% on knowledge question compared with 31.9% for Nigerian students<br>• In addition, 99.0% of South African students believed inappropriate antibiotic use causes ABR vs. 93% among Nigerian students although 99.0% of the respondents in both countries agreed that better antibiotic use could reduce ABR.<br>• 91.4% of South African students vs. only 84.3% of Nigerian students also agreed that using broad-spectrum antibiotics when equally effective narrow-spectrum antibiotics are available encourages ABR<br>• 34.6% of South African students were also very familiar and 64.4% familiar with AMS whilst 43.8% of Nigerian respondents were not familiar and 16.6% not at all familiar with AMS<br>• However, Nigerian students were significantly more confident in knowing when and how to go from IV to oral antibiotics vs. South African students.<br>• 98.3% of Nigerian students also indicated a desire for more education on appropriate antibiotic use.
	Akande-Sholabi et al., 2021 (269)	• Assess the use of antibiotics and knowledge of AMR among future HCPs<br>• Cross-sectional study using a self-administered questionnaire based on the literature and piloted<br>• 866 questionnaires were completed (92.2 % response rate)	• Encouragingly, 88.3 % of respondents were aware that AMR makes it harder to eliminate the infection<br>• However, 95.2 % had used antibiotics in the past 12 months to treat malaria (16.9%), cough (16.3%), sore throat (15.9%) and common cold (12.1%)<br>• 52.4 % purchased their antibiotics from community pharmacies, while others obtained their antibiotics from hospitals (23.3 %), patent medicine stores (18.2 %) and friends and family (6.1 %)<br>• In addition,10.6% strongly agreed and 30.0% agreed that antibiotics will improve the outcome of patients with common colds<br>• Amoxicillin (27.5%) and ciprofloxacin (17.2%) were the most used antibiotics<br>• Students in their 3rd to 6th years had greater knowledge of AMR than others
	Akande-Sholabi et al., 2021 (270)	• Assess prevalence, knowledge and perception of self-medication among healthcare students<br>• Cross-sectional study using a questionnaire based on the literature<br>• 866 students participated (90.2% response rate)	• 55.3% had ‘good’ knowledge, with females 1.4 times more knowledgeable about self-medication<br>• 32.4% self-medicated for minor ailments, with 22.5% believing they had the necessary medical knowledge for this and 11.1% did not want to waste time at the clinic<br>• Analgesics (30.1%), antimalarials (30.0%), antibiotics (15.5%) and multivitamins 12.1% were the most common medicines used for self-medication<br>• The most frequently treated conditions for self-medication among respondents were headaches (18.4%), malaria (16.9%), coughs (9.3%), menstrual pain (8.6%) and colds/influenza (8.0%).

	Abdu-Aguye et al., 2022 (271)	• Assess knowledge of antibiotics, AMR and AMS among fifth year pharmacy students<br>• Descriptive cross-sectional using a structured questionnaire based on previous studies and piloted<br>• 164 questionnaires were completed (76.3% response rate)	• Most students had some knowledge of antibiotics and AMR; however, several misconceptions were identified<br>• Only 23.8% of respondents were able to correctly identify that both antimicrobial resistance and AMR referred to the same concept<br>• The most common conditions identified by respondents for antibiotics were skin infections (91.5%), UTIs (89%), diarrhea (64.6%), sore throats (62.2%) and measles (39%)<br>• Encouragingly interventions to reduce AMR included 'Developing national and institutional antimicrobial usage policies (90.9%)' and 'reducing antimicrobial use in humans (64%)'<br>• In addition, over 90% of participating students stated they would like more education about these topics before graduation
**Upper-Middle Income countries***			
**South Africa**	Augie et al, 2021 (268)	• Assess the knowledge and perceptions medical students about ABR and antibiotic use<br>• Cross-sectional survey using an electronic questionnaire developed from a previous study with four main categories on AMS<br>• 276 students participated - 104 from South Africa, 172 from Nigeria (overall 50.4% response rate)	• 91.4% of South African students surveyed agreed that antibiotics are overused in their country.<br>• South African students scored an average of 62.4% on knowledge questions vs. 31.9% for Nigerian students<br>• 99.0% of South African students believed inappropriate antibiotic use causes ABR vs. 93% among Nigerian students although 99.0% in both countries agreed that better antibiotic use could reduce ABR.<br>• 91.4% of South African students vs. 84.3% of Nigerian students also agreed that using broad-spectrum antibiotics when equally effective narrow-spectrum antibiotics are available may encourage ABR<br>• Encouragingly, 34.6% of South African students were very familiar and 64.4% familiar with AMS whilst 43.8% of Nigerian respondents were not familiar and 16.6% not at all familiar with AMS<br>• However, Nigerian students were significantly more confident in knowing when and how to go from IV to oral antibiotics vs. South African students.<br>• All South African respondents (100%) indicated a desire for more education on appropriate antibiotic use.
	Teague et al, 2023 (272)	• Assess knowledge and perceptions of final-year nursing students regarding antimicrobials and AMS<br>• Quantitative descriptive study using a self-administered online questionnaire<br>• 124 final-year students participated (51.2% response rate)	• Overall mixed knowledge with 84.7% disagreeing that antibiotics can be stopped when patients feel better, 80.6% disagreeing that antibiotics are useful for viral infections and 74.2% disagreeing that antibiotics can be used to treat colds and influenza. However, 43.6% agreed than antibiotics can reduce any kind of pain and inflammation<br>• In addition, only 15.3% of students knew that ceftazidime is not a fourth-generation cephalosporin and only 16.1% were aware that clavulanic acid does not decrease inflammation at the infection site.<br>• Encouragingly, 58.9% agreed that prescribing broad-spectrum antibiotics contributes to AMR and 83.1% that ABR can be minimised by using narrow-spectrum therapy where pertinent. Additionally, 67.7% recognized that poor infection control practices increase AMR.

			• However, AMS was not a well-known concept among participating students, indicating a significant gap in their education regarding strategies to mitigate AMR.

*World Bank Classification; ABR = Antibacterial Resistance; AMR: Antimicrobial Resistance; AMS: Antimicrobial Stewardship; HCW = Healthcare Worker; PHC = Primary Healthcare; RTIs = Respiratory Tract Infections; UTIs = Urinary Tract Infections; URTIs = Upper Respiratory Tract Infections

Table S10: Knowledge, Attitude and Practices, antibiotics, antimicrobial resistance and antimicrobial stewardship – WHO Eastern Mediterranean Region Students

Country	Author and Year	Objectives, Study Design and Population	Summary of the Findings
**Low-Middle Income countries***			
**Egypt**	Assar et al., 2020 (273)	• Identify knowledge and practice gaps in AMS among undergraduate medical students<br>• Cross-sectional using a questionnaire based on previous studies and validated<br>• 963 students completed the questionnaire	• Encouragingly, 95.7% of participating students believed indiscriminate antimicrobial use leads to AMR, with 90.6% stating that AMR means antimicrobials are less likely to work in the future (90.6%). In addition, 94.5% of participating students believed AMR is a serious global issue<br>• 72% of those surveyed also said they complete the full course of antibiotics prescribed; however, 36.2% admitted often saving antibiotics for the next time<br>• Of concern is that 35.7% of those surveyed believed bacteria cause common colds and influenza, with 38.8% stating that antibiotics are first line treatment for coughs and sore throats. In addition, only 37.3% stated they seldom prefer to take an antibiotic for a cough and sore throat<br>• Male students also had frequently lower knowledge scores than female medical students
	Mostafa et al, 2021 (274)	• Examined health literacy and association with antibiotic use, knowledge of antibiotics and awareness of ABR among university students in Egypt<br>• Cross-sectional study using a questionnaire from the literature including WHO Awareness Survey<br>• 508 non-medical students participated	• 38.0% of surveyed students reported using antibiotics in the past month and 62.3% in the last 12 months<br>• Of concern, 73.7% of surveyed students believed antibiotics can be used to treat sore throats, 72.8% to treat colds and influenza, 41.4% diarrhoea and 36.9% body aches<br>• 38% of surveyed students also thought they should stop taking antibiotics once they felt better<br>• In addition, 39.5% of participating students did not know the term ABR with only 30.6% stating they had heard the term AMR<br>• 57.4% of students also incorrectly thought ABR occurs when their body becomes resistant to antibiotics, with 43.3% incorrectly believing ABR is only a problem for people who take antibiotics regularly
	Alsayed et al., 2022 (275)	• Explore KAP regarding antibiotic use among key stakeholders in Arabic countries including medical students<br>• Cross-sectional descriptive study using a pre-validated survey instrument<br>• 2322 (23% of the total survey population) were medical students	• Confidence levels when prescribing or recommending antibiotics among surveyed medical students was relatively low for almost all of the studied aspects<br>• Of concern is that 44.5% of medical students perceived antibiotics as effective against common colds, coughs and nasal congestion with 44.1% believing antibiotics are effective against fevers and 29.5% against viral infections<br>• Encouragingly though, 73% thought ABR was a global problem with 61.5% a problem in their country. In addition, medical students perceived important

		• These included students from Egypt (7.1% of total students)	causes of ABR were using antibiotics when not necessary, not completing the full course and self-medication of antibiotics<br>• Potential interventions to reduce ABR included ready availability of national resistance data and tools to aid appropriate prescribing of antibiotics
	Naser et al., 2024 (276)	• Assess 4265 pharmacy students' KAP regarding ABR among 7 Middle Eastern countries including Egypt (52.7% of students), Jordan (16.5%) and Lebanon (9.4%)<br>• The structured questionnaire was evaluated and validated by clinical pharmacists before use	• The median knowledge score of participating pharmacy students was 5.00 out of 7, with 4th/ 5th year students and bachelor of pharmacy program students have higher odds and more knowledgeable about ABR, with 89.2% agreeing that ABR is increasing. Students from Egypt also had higher odds of being knowledgeable (*P* < .001)<br>• 93.5% agreed they should be more concerned regarding antibiotic consumption and that authorities should create more awareness of ABR<br>• 58.4% agreed that physicians often prescribe antibiotics unnecessarily<br>• Having said this, 51.7% of participating pharmacy students reported they took antibiotics to manage their fever
**Pakistan**	Abubakar et al., 2020 (277)	• Evaluate knowledge and self-confidence regarding ABR, appropriate antibiotic therapy, and AMS among final year pharmacy undergraduate students in three Asian countries including Pakistan<br>• Cross-sectional study using a 59-item questionnaire developed after a literature review and validated<br>• 211 students took part (response rate 77.8%).<br>•	• 80.1% of participating students recognised that inappropriate to treat diarrhoea with antibiotics, and 75.8% strongly disagreed/ disagreed that AMR is not a serious problem<br>• However, only 23.2% were able to select appropriate antibiotics to treat UTIs and only 26.1% that inappropriate to treat URTIs with antibiotics<br>• Approximately 70% of students understood the role of pharmacists in ASPs. However, 95% stated they needed more training in antibiotics and AMR, with only 23.2% having formal training in AMS and 88.1% stating that their knowledge of AMS was only poor to average
	Hussain et al., 2021 (278)	• Assess knowledge and perception regarding AMR and AMS among students<br>• Cross sectional study using online self-administered questionnaire<br>• 496 students completed the questionnaire (86.5% response rate)	• 85.7% of the participating students were familiar with the term AMR, with 79.4% agreeing that a contributing factor for AMR was poorly designed dosing regimens<br>• 87.3% considered the use of broad vs. narrow spectrum antibiotics enhanced AMR (although 66.5% believed broad-spectrum antibiotics are being used inappropriately in Pakistan), with 59.4% identifying that antibiotics are not effective against viruses and 86% that irrational use of antibiotics can harm patients<br>• Pharmacy students were more aware of the inappropriate use of antibiotics in Pakistan than other students, e.g. those from biological sciences<br>• The term AMS was known to 57.9% of the participants, with 74.4% believing that knowledge regarding antimicrobial usage is important to improve patient care and 86.5% that this role can be played by the pharmacist
	Hayat et al, 2021 (279)	• Evaluate understanding about ABR, antibiotic use and AMS programmes among final year pharmacy students<br>• Cross sectional study using self-administered questionnaire based on previous studies and validate	• Regarding antibiotic use, 59.9% of participating students had average understanding with 22.6% good understanding. However, concerns that antibiotics are being frequently prescribed in both public (88.2%) and private healthcare settings (74.0%).

		• 296 students took part in study (85.8% response rate)<br>•	• Of concern is that only 57.4% replied that patients should complete the antibiotic course even when symptoms improve and 51.4% that antibiotics can be given as a preventive medicine to tackle future infections<br>• In addition, 50.3% were unable to identify that diphenhydramine is not an antibiotic, 46.6% that antibiotics can treat influenza and 41.6% that antibiotics are first line treatment for sore throats<br>• 80.1% were aware of the term ABR as this was taught to them. However, 64.9% were not familiar with the term ASP and 59.8% had not been taught about AMS
	Khan et al., 2021 (280)	• Assess awareness of pharmacy students regarding the appropriate use of antibiotics, ABR, and AMS alongside faculty members<br>• Cross sectional study involving 414 undergraduate pharmacy students using a validated questionnaire<br>• 20 faculty members were also interviewed	• Overall, students had good knowledge about antibiotics use and the majority purchased antibiotics through prescription (66.9%) in the last month<br>• Most students had heard the terminologies related to AMR through social media although a minority (25.1%) were unaware of the Pakistan NAP against AMR<br>• Overall, respondents had a somewhat good understanding of the ABR, with 60.9% believing regular use of antibiotics without consultation of a physician can lead to ABR<br>• The majority of the students and teachers believed the current pharmacy syllabus must be updated with new subjects related to ABR and AMS
	Shah et al, 2022 (281)	• Assess KAP of students regarding the use of antibiotics<br>• 525 medical and non-medical students took part in the study	• Only 14% of agreed about the appropriateness of antibiotics for viral infections<br>• However, 65.7% of students took antibiotics only when prescribed by a doctor<br>• 54% though bought antibiotics without prescription and 15% of students stopped taking antibiotics when symptoms subsided<br>• Statistically significant results were found among students who had heard about AMR
	Yasmin et al., 2022 (282)	• Determine the prevalence of self-medication practices among medical and pharmacy students during COVID-19 pandemic<br>• Cross-sectional study using a structured questionnaire<br>• 374 medical and 115 pharmacy students participated (61% response rate)	• 83.0% of students practiced self-medication and reasons included treating cold/flu or preventive measures for COVID-19<br>• Common symptoms for self-medication included fever (67.9%), muscle pain (54.0%), fatigue (51.7%), sore throat (46.6%) and coughs (44.4%)<br>• The most commonly utilized medicines were paracetamol (65.2%), ibuprofen (29%) and azithromycin (25.6%) and multivitamins (56.0%)<br>• Being female, in 3rd year of medical studies, and those with good self-reported health were more frequent users of self-medication practices
	Ahmed et al, 2024 (283)	• Assess knowledge of healthcare students regarding AMR and AMS and impact of training session on their knowledge<br>• Mixed method study<br>• 226 health-care students were enrolled	• A significant difference in mean AMR knowledge score was observed among student nurses, those undertaking a degree in blood transfusion medicine and medical student groups<br>• However, no statistical difference was observed between nursing and medical students<br>• Encouragingly, there was a significant increase in mean knowledge scores regarding AMS and AMR following training sessions<br>• Overall, awareness regarding AMR and AMS can be improved among healthcare students by the introduction of curriculum related to AMR and AMS

**Palestine**	Alsayed et al., 2022 (275)	• Explore KAP regarding antibiotic use among key stakeholders in Arabic countries including medical students<br>• Cross-sectional descriptive study using a pre-validated survey instrument<br>• 2322 (23% of the total survey population) were medical students<br>• These included students from Palestine (2.1% of total students)	• Confidence levels when prescribing or recommending antibiotics among medical students was seen as relatively low for almost all of the studied aspects<br>• Of concern is that 44.5% perceived antibiotics as effective against common colds, coughs and nasal congestion with 44.1% effective against fevers and 29.5% against viral infections<br>• Encouragingly, 73% thought ABR was a global problem with 61.5% a problem in their country. In addition, medical students perceived important causes of ABR were using antibiotics when not necessary, not completing the full course, and self-medication of antibiotics<br>• Potential interventions to help reduce ABR include ready availability of national resistance data and tools to aid appropriate prescribing of antibiotics
	Abuawad et al., 2024 (284)	• Investigate KAP of medical students towards AMR and AMS<br>• Cross-sectional study using a structured questionnaire<br>• 384 medical students completed the study.	• Overall, surveyed medical students demonstrated high awareness/ knowledge about AMR with 97.4% knowing about AMR and 86.7% that the use of broad-spectrum antibiotics increases AMR. In addition, only 9.9% believed that antibiotics kill viruses<br>• In addition, 94.5% of surveyed students believed it is important to follow the appropriate duration of antibiotics to reduce ABR, 84.4% that poor patient adherence to antibiotics increases AMR and 76.0% that broad-spectrum antibiotics are unnecessary when narrow-spectrum antibiotics are available and will increase AMR. Alongside this, 81.8% that AMS seeks to optimize antimicrobial use<br>• However, 66.1% of surveyed students were not confident about their knowledge regarding AMS
**Sudan**	Elmahi et al., 2022 (285)	• Evaluate the knowledge and attitude of medical students in Sudan towards antibiotics including the prevalence and rationale of any self-medication with antibiotics<br>• Cross-sectional survey using a pre-validated and pretested questionnaire<br>• 1100 medical students participated	• Encouragingly, 84.9% of participating students were aware that repetitive non-compliance with antibiotics leads to ABR<br>• In addition, 80.9% strongly disagreed or disagreed that the same antibiotics will always be effective in the future, with 78.5%, 72.5% and 63.8% respectively strongly disagreeing or disagreeing that antibiotics should be stopped when symptoms improve, antibiotics can be used for the same symptoms as paracetamol and antibiotics can be used to treat colds or influenza<br>• However, only 41.2% strongly disagreed/ disagreed that sore throats can be treated with antibiotics and 56.3% that broad spectrum antibiotics are preferable under all circumstances<br>• In addition, 60.8% had self-medicated with antibiotics within the previous 12 months with azithromycin (29.9%) and amoxicillin/clavulanic acid (26.8%) the most commonly purchased<br>• The principal infections for self-medication with antibiotics were RTIs (38.1%), coughs (30.4%) and common colds (26.2%)
	Abdelkarim et al., 2024 (286)	• Assess the knowledge and confidence in antibiotic therapy, ABR and AMS among final-year undergraduate pharmacy students	• Encouragingly more than 70% of surveyed students demonstrated good knowledge of AMR in terms of defining it and explaining its mechanisms, and that AMR is a global threat; 62.4% disagreed or strongly disagreed with the notion that ABR is not a serious issue and 68% strongly agreed/agreed that comprehensive knowledge of ABR is essential in their pharmacy career

		• Cross-sectional study using an online questionnaire developed following a review of the literature<br>• 109 students responded (36% response rate)	• In addition, 75.2% could identify AMS interventions and 57.8% define the goals of ASPs<br>• However, only 24.8% could identify the daily dose of gentamicin, only 29.4% could identify key factors contributing to the spread of ABR, and only 37.6% that URTIs do not potentially need antibiotics for treatment<br>• In addition, 41.3% believed the problem of ABR is being exaggerated, only 31.2% could identify a situation where AMS is not necessary and one-third of students lacked confidence in interpreting microbiological results
**Upper-Middle Income countries***			
**Iran**	Abdi et al., 2018 (287)	• Determine the prevalence of self-medication and its related factors among the health sciences students<br>• Cross-sectional study with a structured questionnaire<br>• 250 students took part	• Self-medication was common at 89.6% of participating students. Prior experience about the illness, non-seriousness of the illness and the ready availability of medicines were the most prevalent reasons for self-medication<br>• The most commonly used medications included medicines for common colds drugs, analgesics and antibiotics, with the most frequently used medications being cold pills, acetaminophen and amoxicillin capsule<br>• Most students obtained their pharmaceutical information from pharmacists, physician or online sources
**Iraq**	Alsayed et al., 2022 (275)	• Explore KAP regarding antibiotic use among key stakeholders in Arabic countries including medical students<br>• Cross-sectional descriptive study using a pre-validated survey instrument<br>• 2322 (23% of the total survey population) were medical students<br>• These included students from Iraq (18.2% of total students)	• Confidence levels when prescribing or recommending antibiotics among surveyed medical students was seen as relatively low for almost all of the studied aspects<br>• Of concern is that 44.5% of medical students perceived antibiotics as effective against common colds, coughs and nasal congestion with 44.1% believing antibiotics were effective against fevers and 29.5% against viral infections<br>• Encouragingly, 73% thought ABR was a global problem with 61.5% a problem in their country. In addition, medical students perceived important causes of ABR were using antibiotics when not necessary, not completing the full course, and self-medication of antibiotics<br>• Potential interventions to help reduce ABR included ready availability of national resistance data and tools to aid appropriate prescribing of antibiotics
**Jordan**	Najjar et al., 2021 (288)	• Assess the prevalence, knowledge and reasons for self-treatment with antibiotics among undergraduate university students<br>• Cross-sectional study using a structured questionnaire developed with validated questions from previously studies and piloted<br>• 201 students participated (84% response rate) with 64.2% from health-related subjects	• Encouragingly, 67.2% of participating students stated they knew about ABR, with 69.8% of students taking health-related subjects believing repeated and unnecessary use of antibiotics increases ABR making them less effective (20.8% non health-related students) and 64.3% believing ABR means antibiotics are no longer active to eliminate bacteria (31.9% non health-related students)<br>• In addition, 53.5% of students in health-related subjects stated that they can differentiate between viral and bacterial infections vs. 36.1% in non health-related subjects with 74.4% correctly identifying that viruses are the main cause of colds and cough (77.8% non-health related students)<br>• However, when antibiotics were used – they were used to treat more than one health problem (50.2% of participating students), cold/ influenza/ tonsilitis (23.9%), pains (9%) or a fever (7%)<br>• In addition, 55.8% of health-related students would stop antibiotics when symptoms are relieved (27.8% non health-related students)

	Al-Qerem et al., 2022 (289)	• Assess KAP towards antibiotics and ABR among pharmacy students<br>• Cross-sectional online study using a questionnaire adapted from previous studies and piloted<br>• 890 students completed the questionnaire.	• Encouragingly, 86.85% of surveyed students believed indiscriminate use of antibiotics can lead to ineffective treatments and 91.46% to ABR; 92.03% also believed that if antibiotics are taken too often, they are less likely to work in the future<br>• In addition, 67.52% believed is an important and serious public health issue globally and 62.29% in Jordan<br>• However, 35.39% believed bacteria cause colds and influenza although only 15% agreed/ strongly agreed with the statement that patients should take antibiotics for a common cold to prevent getting worse. In addition, 28.6% agreed/ strongly agreed that taking antibiotics for a fever helps patients get better more quickly
	Alsayed et al., 2022 (275)	• Explore KAP regarding antibiotic use among key stakeholders in Arabic countries including medical students<br>• Cross-sectional descriptive study using a pre-validated survey instrument<br>• 2322 (23% of the total survey population) were medical students. These included students from Jordan (60.3% of total participating students)	• Confidence levels when prescribing or recommending antibiotics among surveyed medical students was seen as relatively low for almost all of the studied aspects<br>• Encouragingly, 73% thought ABR was a global problem with 61.5% that ABR was a problem in their country. In addition, medical students perceived important causes of ABR were due to using antibiotics when not necessary, not completing the full prescribed course, and self-medication with antibiotics<br>• Of concern is that 44.5% of medical students perceived antibiotics as effective against common colds, coughs and nasal congestion with 44.1% believing antibiotics were effective against fevers and 29.5% against viral infections<br>• Potential interventions to help reduce ABR included ready availability of national resistance data and tools to aid appropriate prescribing of antibiotics
	Al-Taani et al, 2022 (290)	• Assess survey medical, nursing and pharmacy students' KAP regarding antimicrobial use and AMR<br>• cross sectional online survey using a questionnaire adapted from an ECDC validated instrument<br>• 716 undergraduate students participated	• Encouragingly, 93.0% of surveyed students believed unnecessary use of antibiotics makes them ineffective, 85.5% that antibiotics are ineffective against viruses, 76.7% ineffective against colds and influenza<br>• In addition, 85.5% strongly agreed/ agreed what ABR is; 63.8% that they have a key role in preventing ABR and 55.2% that they have good opportunities to provide advice on prudent antibiotic use to others<br>• However, 34.6% were not aware of any initiatives in their country regarding antibiotic awareness and AMR
	Naser et al., 2024 (276)	• Assess 4265 pharmacy students' KAP regarding ABR among 7 Middle Eastern countries including Egypt (52.7% of students), Jordan (16.5%) and Lebanon (9.4%)<br>• The structured questionnaire was evaluated and validated by clinical pharmacists before use	• The median knowledge score of participating pharmacy students was 5.00 out of 7, with 4th/ 5th year students and bachelor of pharmacy program students have higher odds and more knowledgeable about ABR, with 89.2% agreeing that ABR is increasing. Students from Egypt also had higher odds of being knowledgeable (*P* < .001)<br>• 93.5% agreed they should be more concerned regarding antibiotic consumption and that authorities should create more awareness of ABR<br>• 58.4% agreed that physicians often prescribe antibiotics unnecessarily<br>• Having said this, 51.7% of participating pharmacy students reported they took antibiotics to manage their fever
**Lebanon**	Sakr et al., 2020 (291)	• Evaluate the KAP towards antibiotics and ABR among Lebanese university students	• 78% of respondents from the health-related subjects scored high knowledge regarding antibiotics and ABR compared to only 41% of non-health related subjects including the effectiveness of antibiotics to treat bacterial/viral

		• Cross-sectional study - questionnaire-based survey with the questionnaire based on published studies and piloted<br>• 1250 students participated	infections (80.2% vs. 36.9%) and familiarity with terms related to ABR – 93.3% vs. 56.6%<br>• However – concerns regarding knowledge to of antibiotics to treat fevers (only 40.3% correct among students studying health-related subjects vs. 37.2% for the others) and headaches – only 11.6% correct among students studying health- related subjects vs. 17.7% among the others<br>• The attitude scores among students studying health-related subjects were also more positive and satisfactory than others, e.g. 'Is antibiotic resistance an important and serious global public health issue?' – 94.6% among health-related students vs. 83.6% others and 94.4% vs 89.2% for 'Do you think that antibiotic resistance can result from inappropriate use of antibiotics?'
	Alsayed et al., 2022 (275)	• Explore KAP regarding antibiotic use among key stakeholders in Arabic countries including medical students<br>• Cross-sectional descriptive study using a pre-validated survey instrument<br>• 2322 (23% of the total survey population) were medical students<br>• These included students from Lebanon (0.5% of total students)	• Confidence levels when prescribing or recommending antibiotics among surveyed medical students was seen as relatively low for almost all of the studied aspects<br>• Of concern is that 44.5% of medical students perceived antibiotics as effective against common colds, coughs and nasal congestion with 44.1% believing antibiotics were effective against fevers and 29.5% against viral infections<br>• Encouragingly, 73% thought ABR was a global problem with 61.5% a problem in their country. In addition, medical students perceived important causes of ABR were using antibiotics when not necessary, not completing the full course, and self-medication of antibiotics<br>• Potential interventions to help reduce ABR included ready availability of national resistance data and tools to aid appropriate prescribing of antibiotics
	Atallah et al., 2023 (292)	• Evaluate the impact of social media education on spreading AMS awareness among principally healthcare students<br>• Self-completed questionnaire online<br>• 125 students took part	• Overall, 85.6% of surveyed students believed that antibiotics are overused with 26.4% admitting that they overuse antibiotics<br>• Encouragingly, 88.8% confirmed the importance of having ASPs to help improve future antibiotic use.<br>• 76.8% regularly use social media for educational purposes with a significant 36.2% improvement between pre and post questioning following social media education
	Naser et al., 2024 (276)	• Assess 4265 pharmacy students' KAP regarding ABR among 7 Middle Eastern countries including Egypt (52.7% of students), Jordan (16.5%) and Lebanon (9.4%)<br>• The structured questionnaire was evaluated and validated by clinical pharmacists before use	• The median knowledge score of participating pharmacy students was 5.00 out of 7, with 4th/ 5th year students and bachelor of pharmacy program students have higher odds and more knowledgeable about ABR, with 89.2% agreeing that ABR is increasing. Students from Egypt also had higher odds of being knowledgeable ($P < .001$)<br>• 93.5% agreed they should be more concerned regarding antibiotic consumption and that authorities should create more awareness of ABR<br>• 58.4% agreed that physicians often prescribe antibiotics unnecessarily<br>• Having said this, 51.7% of participating pharmacy students reported they took antibiotics to manage their fever

*World Bank Classification; ABR = Antibacterial Resistance; AMR: Antimicrobial Resistance; AWaRe = Access, Watch, Reserve antibiotics; FGD = Focus Group Discussions; HCP = Healthcare Professional; HCW = Healthcare Worker; PHC = Primary Healthcare; KAP: Knowledge, Attitude and Practice; URTI: Upper Respiratory Tract Infection

Table S11: Knowledge, Attitude and Practices, antibiotics, antimicrobial resistance and antimicrobial stewardship – WHO South East Asian Region Students

Country	Author and Year	Objectives, Study Design and Population	Summary of the Findings
**Low Income countries***			
**Nepal**	Shah et al., 2019 (293)	• Assess KAP associated with antibiotic use among both medical and non-medical students<br>• Cross-sectional study using a self-administered questionnaire based on previous studies and piloted<br>• 1222 students took part - 609 medical and 613 non-medical (99.1% response rate)	• The overall knowledge scores were significantly higher for participating medical vs. non-medical students (mean: 10.98 vs. 8.60 - p < 0.001), with more medical students having heard of AMR (97.7%) vs. non-medical students (50.2%) – with higher levels as well for attitude and practice<br>• In addition, 85.5% of medical students did not think frequent use of antibiotics decreases the occurrence of infections vs. 72.2% for non-medical students. 82.9% of medical students also thought AMR was a problem in Nepal (vs. 41.6% for non-medical students) and 90.6% that over use of antibiotics causes AMR (46.0% for non-medical students)<br>• Alongside this, 99.7% of students believed it was necessary to know more about the rational use of antibiotics (vs. 95.2% for non-medical students)<br>• However, concerns that 73.7% of participating medical students thought that antibiotics can be used to cure infections caused by viruses (vs. only 34.2% for non-medical students), 94.1% thought it acceptable to stop taking antibiotics when feeling better (vs. 76.3% for non-medical students) and 39.4% of medical students thought that antibiotics speed up the recovery from colds and coughs (23.8% for non-medical students)
	Shrestha 2019 (294)	• Assess KAP towards antibiotics use and ABR among undergraduate medical students<br>• Descriptive cross-sectional study using a questionnaire based on published studies<br>• 228 students fully participated (78.6% response rate)	• Encouragingly, 83.3% of participating students agreed that ABR means that if antibiotics are taken too often they are less likely to work in the future - leading to ineffective treatment (92.5% of students) – with 88.6% believing that ABR is a problem globally<br>• In addition, only 25.9% agreed that antibiotics are useful for viral infections, 75% would never stop taking antibiotics when they felt better if prescribed by physicians and 71.1% of participating students stated they never save antibiotics for the next illness<br>• However, 46.1% believed that antibiotics would speed up recovery from colds and influenza
	Mandal et al., 2020 (295)	• Ascertain the prevalence of self-medication practice of antibiotics among medical and dental undergraduate students<br>• Descriptive cross-sectional study using a pre-tested self-administered questionnaire<br>• 558 students participated	• Self-medication with antibiotics was higher in medical students (61.7% of participating medical students) than dental students (38.3%)<br>• Infectious diseases self-medicated with antibiotics included sore throats with runny nose (45.3% of self-medication), fever (31.6%) and tonsil infections (20.4%)<br>• 17.9% of students obtained antibiotics from family members/ relatives, 17.5% from friends/ seniors and 17.2% used leftover antibiotics

	Shah et al., 2021 (296)	• Assess the perceptions, knowledge, and awareness of self-medication practice among university students<br>• Descriptive cross-sectional, question-naire-based study using a validate ques-tionnaire<br>• 620 students participated	• 95.4% of students self-medicated - the majority of surveyed students took analgesic/ antipyretics (66.1%) as well as anti-ulcer medicines (35.3%) and antibiotics (33.9%) - principally because of long-waiting times to see pro-fessionals in hospitals/ clinics (46.7%) followed by time and expenditure (approx. 20%)<br>• The most common conditions for self-medication were colds, headache and sore throats<br>• The principal factor influencing the use of antibiotics was re-use of old medicines (approx. 50%)
**Low-Middle Income countries***			
**Bangladesh**	Seam et al, 2018 (297)	• Assessing the perceptions regarding self-medication practice among the pharmacy students<br>• Study was conducted by using both qualitative and quantitative data<br>• 250 students took part	• Whilst the practice of self-medication was high among surveyed students: headache (71.20%); cough, cold/flu (61.20%); diarrhoea (47.60%); pain (42.80%); and stomach ache (32.80%) – use of antibiotics was low (15.6%)<br>• Most common medicines for self-medication were antipyretics (58.40%); analgesics (49.20%); anti-diarrhoeals (39.20%); and antacids (38.8%)<br>• Self-medication was typically practiced as students felt they did not need to see a doctor for a minor illness
	Marzan et al. 2021 (298)	• Assess and compare knowledge and attitude regarding antibiotics among public university biology and non-biology students.<br>• A cross-sectional design using a questionnaire based on the literature<br>• 210 responded (53% response rate)	• 42.4% of surveyed students had good knowledge and 34.1% had poor knowledge regarding antibiotics<br>• The knowledge gap was more prevalent in non-biology students (62.8%) than biology students (32.6%), with biology students more than four times as likely to have higher levels of knowledge about antibiotics than non-biology students<br>• However, 44.4% of students held misconceptions that antibiotics could prevent viral infections, higher among non-biology students. In addition, 28.3% biology students vs. 31.9% non-biology students that antibiotics are effective for treating viral infections<br>• 91.7% of students reported that antibiotic consumption without a physician's prescription and overuse (82.0%) could promote ABR, with higher rates among biology students<br>• 96.6% of surveyed students also thought the government should create more awareness to prevent ABR
	Paul et al., 2022 (299)	• Evaluate KAP towards AMR and practice of self-medication among University students<br>• Cross-sectional study using a structured questionnaire<br>• 200 students took part in the study	• 42.2% of surveyed had previously used antibiotics for fever, 10.1% for a common cold and 8.4% for diarrhoea<br>• Of concern is that 54.5% of respondents thought that "sometimes" self-medication with an antibiotic is not a problem and 32.5% kept antibiotics for future use<br>• Encouragingly only 6% disagreed/ strongly disagreed that the unnecessary use of antibiotic makes them inactive and 7% that ABR would arise if a full course of antibiotic was not completed
	Wahab et al., 2023 (300)	• To identify the prevalence and patterns of self-medication with antibiotics among university students to shed light on the	• 61.0% of participants had self-medicated with antibiotics in the last six months exacerbated by 60.0% of students exhibiting a substandard understanding of ABR and appropriate antibiotic usage

		extent of antibiotic misuse and risks as well as understand key factors driving self-medication<br>• A structured questionnaire was used consisting of three sections<br>• 951 students took part	• Male students (61.7%) were found to self-medicate more often than female students (38.3%)<br>• The most common reasons for self-medication among surveyed students were previous experience with the illness (40.9%) and belief that the illness was not serious (36.2%)<br>• The most common infections for self-medication were a fever (40.9%) and a cough/ cold (29.3%)<br>• Age, sex, parents' occupation, maintaining diet chart and exercising regularly were related to higher tendency for self-medication
**India**	Gupta et al, 2019 (301)	• Assess KAP among medical students re-garding antibiotic use and AMR<br>• Cross sectional study using structured questionnaire based on previous studies and validated via a pilot study<br>• 474 students completed the forms	• 91.8% of surveyed students were aware that antibiotics are useful for bacterial infections, with significant knowledge regarding antibiotics observed among students except first year students. 84.7% disagreed regarding the use of antibiotics to alleviate pain and inflammation in body<br>• Knowledge about the side-effects of antibiotics ranged from 78%-86.5%; however, there was unsatisfactory knowledge about the type of antibiotics used for MRSA<br>• Of concern was that 64.3% of participating students keep leftover antibiotics for future use, 27.6% stopped taking antibiotics when they feel better, 23.2% took antibiotics for colds and sore throats and 17.9% for fevers<br>• Overall, whilst knowledge was satisfactory the attitude and practices of students need to be improved
	Ritchie et al., 2020 (302)	• Assess knowledge, attitude and factors associated with confidence with intentions to prescribe antibiotics<br>• Cross-sectional study using a questionnaire adapted from previous studies<br>• 347 medical students took part (approximate 98% response rate)	• Encouragingly, 85.3% of surveyed students believed antibiotics are being overused nationally, 84.1% their inappropriate use causes AMR, 81.6% that better use of antibiotics will reduce AMR and prescribing broad-spectrum antibiotics when equally effective narrower spectrum ones are available increases AMR<br>• In addition, 82.4% thought patients' incomplete antibiotic treatment courses the most common factor contributing to AMR, with 58% reporting excessive number of antibiotic prescriptions. Alongside this, only 9.2% of students disagreed that AMR was a non-significant problem in hospitals in their rotations<br>• Of concern is that only 13.2% of participating students had knowledge of ASPs. 64% also believed knowledge of antimicrobials is essential for their medical career; however, 41% were unaware of their hospital's current antibiotic guidelines and only 15.9% had a copy of their hospital's antibiotic guidelines. In addition, only 35% of students were confident in their capability to prescribe antibiotics<br>• Overall, 88.2% of students would like more education on antibiotic selection at medical school and 59% believed that education on AMR would be beneficial for them
	Agarwal et al., 2022 (303)	• Assess the knowledge and attitude of undergraduate, interns, and postgraduate medical students	• 80.67% of participating students correctly identified that bacterial infections require antibiotics; however, 19.33% believed antibiotics could be used for both viral and bacterial infections. 53.3% believed narrow spectrum

		• concerning antimicrobials and ABR and associated factors.<br>• Completed a validated self-administered questionnaire<br>• 150 students took part -50 final year students, 50 interns and 50 resident doctors	antibiotics are preferred with 44.7% believing broad spectrum ones are preferred<br>• 74% had knowledge that the choice of antibiotic should cover all pathogenic bacteria at the site of infection<br>• In addition, 88.67% disagreed that the use of antimicrobials for patients even when they do not need them does not cause significant damage and 88% believed antimicrobials were being over used in theirs and other hospitals in the country<br>• Most common contributing factors for antibiotic prescriptions were a purulent discharge presence (92%), prevent serious complications (82.67%), presence of fever (56.67%) and when diagnosis is uncertain (39.33%). Encouragingly only 9.33% said a self-limiting condition and only 3.33% patient request<br>• Encouragingly as well, 68% disagreed that antimicrobials are required in watery diarrhoea and 63.33% that antimicrobials are needed in rhinitis<br>• However, 56% preferred fluroquinolones for UTIs and 31.33% prescribed third generation cephalosporins for surgical site infections. In addition, only 33.3% were aware of the antibiotic policies in their hospitals and only 56.67% were aware of the Infection Control Programme in their hospitals<br>• Overall, 96.67% thought AMR a worldwide problem while 79.33% considered it a significant problem in India which can be addressed by formulating learning objectives and implementing competency-based curriculum for undergraduate students
	Nabi et al., 2022 (304)	• Explore trends in self-medication with antibiotics and risk factors among medical undergraduates<br>• Cross-sectional study with a structured questionnaire among 360 medical students	• 67.78% of students had self-medicated with antibiotics, higher among females<br>• 74.59% of students self-medicating had experienced adverse events<br>• Convenience (86.1%) and past experiences (24.6%) were the principal reasons of practicing self-medication - with antibiotics readily available for sale among community pharmacies<br>• Fever (47.5%) and RTIs (39.3%) principal indications for self-medication with extended-spectrum penicillins (60.7%) and the quinolones (33.6%) the most commonly purchased class of antibiotics. Of equal concern was that 41.0% of students stop taking antibiotics when they feel better
**Indonesia**	Abubakar et al., 2020 (277)	• Evaluated knowledge and self-confi-dence regarding ABR, appropriate anti-biotic therapy, and AMS among final year pharmacy undergraduate students in three Asian countries including Indo-nesia<br>• Cross-sectional study using a 59-item questionnaire developed after a litera-ture review and validated<br>• 211 students took part (response rate 77.8%).	• Encouragingly, 80.1% of participating students recognised that it was inap-propriate to treat diarrhoea with antibiotics, and 75.8% strongly disagreed/ disagreed that AMR is not a serious problem<br>• However, only 23.2% were able to select appropriate antibiotics to treat UTIs and only 26.1% stated that it was inappropriate to treat URTIs with antibiotics<br>• Approximately 70% of students understood the role of pharmacists in ASPs. However, 95% stated they needed more training in antibiotics and AMR, with only 23.2% having formal training in AMS and 88.1% stating that their knowledge of AMS was only poor to average

		•	
**Sri Lanka**	Sakeena et al., 2018 (305)	• Assess and compare knowledge of antibiotics and AMR between pharmacy and other allied health sciences students<br>• Cross-sectional study using a questionnaire modified from the WHO<br>• 386 students took part (69% response rate)	• Encouragingly, the majority of participating students were familiar with terms such as ABR (pharmacy students - 90%, others - 85%), AMR (pharmacy 80%, others 52%), drug resistance (pharmacy 73%, others 74%) and antibiotic resistant bacteria (pharmacy 7%, others 67%)<br>• Overall, participating pharmacy students exhibited greater knowledge regarding appropriate antibiotic use than non-pharmacy ones<br>• However, both student sets had misconceptions about some antibiotic use, e.g. believing the following could be cured by antibiotics: cold / flu (pharmacy - 63%, others 59%), body-aches (pharmacy - 13%, others - 18%) and headaches (pharmacy - 4%, others 18%)<br>• In addition, 36% of non-pharmacy students and 22% of pharmacy students believed antibiotics should be stopped when patients feel better
	Sakeena et al., 2018 (306)	• Investigate antibiotic use, knowledge of antibiotics and AMR among undergraduate pharmacy students at Sri Lankan universities<br>• Cross-sectional study using a WHO based questionnaire<br>• 466 students took part (typically response rates above 80% for participating universities)	• Encouragingly, 100% of senior pharmacy students were knowledgeable of the term ABR (90% for junior students), 90% for AMR (65% for junior students) and 93% for drug resistance (70% for junior students)<br>• In addition, 80% of senior students believed infections are becoming increasingly resistant to antibiotics (57% for juniors), with 82% of seniors believing ABR is an issue that could affect them and their families (64% for juniors). Alongside this, 77% disagreed that ABR is only a problem for people who take antibiotics regularly (64% for juniors)<br>• However, the proportion of participating pharmacy students reporting that antibiotics can be used to treat UTIs were 76%, sore throats - 57%, cold and influenza - 51%, diarrhoea - 49% and fever - 41%, with typically higher scores for senior students
	Jayaweerasingham et al., 2019 (307)	• Identify KAP on antibiotic use and ABR among student nurses<br>• Cross-sectional study using a pre-validated, self-administered questionnaire<br>• 199 nursing students participated	• 97% of surveyed nurses had heard about ABR and 73.9% believed their inappropriate use leads to ABR.<br>• In addition, 88.5% believed that if antibiotics are taken for long time, bacteria become more resistant to them<br>• However, 40.2% believed antibiotics were indicated for common colds with 37.2% and 39.7% respectively believing that taking antibiotics during a cold will prevent it from worsening or help patients recover faster<br>• Alongside this, 34.7% believed antibiotics were active against viruses and 72.2% would stop taking antibiotics if they felt better
	Sakeena et al., 2019 (308)	• Investigate and compare antibiotic use and knowledge of antibiotics and AMR among undergraduate pharmacy students in Australian and Sri Lankan universities<br>• Cross-sectional study using a WHO based questionnaire<br>• 466 undergraduate pharmacy students from Sri Lanka (63% response rate)	• Encouragingly the majority of pharmacy students from Sri Lanka were familiar with the following terms related to AMR: ABR - 94% of participating students AMR - 76%, drug resistance - 80%, and antibiotic resistant bacteria 88% - Generally though appreciably greater knowledge among Australian students (*p* = 0.001)<br>• In addition, 67% of Sri Lankan students believed many infections are becoming increasingly resistant to treatment by antibiotics with 72% believing ABR is an issue that could affect me or my family<br>• However, a significantly higher percentage of Sri Lankan pharmacy students incorrectly believed antibiotics were appropriate for colds/

			influenza - 51%, body aches - 11% and headaches - 6%) when compared to Australian students<br>• In addition, only 28% believed ABR occurs when your body becomes resistant to antibiotics and they no longer work

*World Bank Classification; ABR = Antibacterial Resistance; AMR: Antimicrobial Resistance; AWaRe = Access, Watch, Reserve antibiotics; FGD = Focus Group Discussions; HCP = Healthcare Professional; HCW = Healthcare Worker; PHC = Primary Healthcare; KAP: Knowledge, Attitude and Practice; URTI: Upper Respiratory Tract Infection

Table S12: Knowledge, Attitude and Practices, antibiotics, antimicrobial resistance and antimicrobial stewardship – WHO Western Pacific Region Students

Country	Author and Year	Objectives, Study Design and Population	Summary of the Findings
**Upper-Middle Income countries***			
**China**	Hu et al., 2018 (309)	• Assess KAP among medical students with respect to antibiotic use for self-limiting conditions<br>• Online cross-sectional survey using a structured questionnaire<br>• 1819 medical students participated	• 29.1% of medical students reported at least one self-limiting illness in the prior month. Of these, 54.0% self-medicated, with 27.0% using antibiotics; 21.0% saw a physician with 58.0%prescribed antibiotics<br>• In the past year, 15.0% had used antibiotics as prophylaxis, and 15.0% of had demanded an antibiotic from a doctor. In addition, 64.0% of respondents kept a personal stock of antibiotics<br>• Students with high KAP scores with respect to antibiotics were significantly less likely to self-medicate with antibiotics ($p = 0.031$), use antibiotics for prophylaxis ($p < 0.0001$), or demand an antibiotic ($p = 0.007$) from the doctor<br>• More than 85.0% of the medical students were aware that the overuse of antibiotics in China as a serious problem that could cause AMR in the future and lead to difficulties in treating bacterial infections.<br>• More than 60.0% of the medical students answered correctly that the common cold was a self-limiting disease not requiring antibiotics, with 92.0% agreeing that antibiotics were effective for treating bacterial infections<br>• However, only 47.0% did not agree that antibiotics could reduce the symptoms of the common cold
	Peng et al., 2018 (310)	• Explore the antibiotic misuse among university students<br>• Questionnaire based study with the questionnaire adapted from previous studies<br>• 3995 students took part with medical students accounting for 22.1% of these. 2073 university students from Guizhou (rural) and 1922 from Zhejiang (urban) completed the questionnaire	• Compared with students in Zhejiang, students in Guizhou were more likely to see a doctor (35.0% vs. 28.3%) when they were ill; more likely to buy over-the-counter antibiotics (73.9% vs 63.4%); ask for antibiotics from doctors (21.4% vs 15.6%); use antibiotics prophylactically (29.9% vs 15.7%); less likely to select primary care health facilities (66.7% vs. 77.1%); have higher antibiotic use prescribed by doctors (79.8% vs 56.2%) and self-medicate with antibiotics (33.0% vs 16.1%).<br>• Students with a medical background were significantly associated with better antibiotic use behaviours, i.e. less likely to ask for antibiotics from doctors ($p < 0.01$), and take antibiotics prophylactically ($p < 0.01$).<br>• However, students whose parents had medical backgrounds demonstrated poorer antibiotic use behaviours, i.e. more likely to self-treat with antibiotics ($p < 0.001$), keep antibiotics in their dorm/home ($p < 0.01$), and take antibiotics prophylactically ($p < 0.05$)

	Lin et al., 2020 (311)	• Investigate the decision-making process of Chinese university students regarding antibiotic use for self-diagnosed URTIs<br>• Cross-sectional study using a systematically developed questionnaire<br>• 2834 university students participated	• 33.4% self-reported having taken antibiotics and 48.8% used non-prescription antibiotics<br>• 20.4% of university students with self-diagnosed URTIs decided against treatment, 54.5% decided to self-treat, and 25.1% sought formal care. 17.3% admitted to pressuring their doctors for antibiotics, with a 100% success rate.<br>• Having said this, 74.2%–88.5% reported being aware of the dangers posed by overuse of antibiotics. However, 63.0% of participating students had trouble distinguishing cold medicines from antibiotics; 36.5% were unaware that antibiotics were not anti-inflammatory drugs and more than 60% had an incorrect perception of antibiotic efficacy for URTIs<br>• Surprisingly, high ability to identify antibiotics was linked to higher likelihood of antibiotic use, especially antibiotic prescriptions<br>• At health facilities, approximately 70% of clinicians prescribed antibiotics for URTI symptoms with practically no barriers to accessing antibiotics from a doctor.
	Wang et al., 2020 (312)	• Assess KAP among public health undergraduates regarding AMR<br>• Cross-sectional survey using a structured questionnaire based on a WHO questionnaire<br>• 1122 questionnaires were completed (94.4% response rate)	• 75.2% of students had performed incorrect antimicrobial practices with only 37.4% of students correctly answering the question about the use of antimicrobials for common colds<br>• 31.7% of public health students perceived their AMR-related knowledge to be either limited or very limited, with 84.2% of students surveyed believing they did not receive enough knowledge about antimicrobials and were willing to learn more<br>• In addition, 60% of students reported a lack of specific lectures about AMR, while more than one-half did not consider AMR to be a key point in their exams
	Min et al., 2022 (313)	• Describing KAP concerning AMR among medical students<br>• Questionnaire-based survey using a questionnaire based on previously validated instruments<br>• 1066 respondents were enrolled into the study (88.83%) – 564 in 2017 and 502 in 2022	• The undergraduates had a significant increase in their knowledge and awareness of AMR during the 5 years from 2017 to 2022 ($p < 0.001$), e.g. 97.0% in 2022 vs 79.1% in 2017 strongly agreed/agreed that doctors should only prescribe antimicrobials when they are needed<br>• A majority of respondents (76.8% in 2022 and 77.5% in 2017) agreed on the current abuse of antimicrobials and would like to take responsibility for reducing them. Overall, suggesting that public education on knowledge could influence the behaviours among the medical students<br>• Encouragingly, only a small proportion of medical students (4.8% in 2022; 6.2% in 2017) stated they would use antimicrobials to treat common colds. In addition, 67.3% of students in 2022 correctly answered as false that AMR occurs when your body becomes resistant to antimicrobials, and they no longer work as well (vs. 45.6% in 2017), with only 25.3% of students in 2022 saying they would buy the same antimicrobials, or request these from a doctor, if they were sick and they helped before vs. 69.3% in 201<br>• Alongside this, only 28.9% of students in 2022 agreed they would use antimicrobials that were given to a friend or family as long as they were used to treat the same illness vs 66.8% in 2017
	Yang et al., 2024 (314)	• Evaluate the levels and explore the associated factors with KAP regarding antibiotic use and AMR among nursing students in China	• Of concern is that 54.3% of participants were unaware that antibiotics were ineffective against viral infections, only 41.6% agreeing that every person treated with antibiotics is at an increased risk of antibiotic-resistant infection, and only 41.0% agreeing that AMR can affect their and their family's health

		• Cross-sectional survey using a self-administered questionnaire based on a WHO questionnaire<br>• 1959 students returned completed survey (95.9% response rate)	• In addition, only 36% agreed that current antibiotic abuse existed, with 23.1% of participants stating they sometimes took antibiotics when they have a fever, and 24.3% sometimes using antibiotics when they have a cold.<br>• However, only 31.5% of participants believed they had adequate knowledge of how to use antibiotics appropriately in current practice. 79.5% of students stated there was a discussion on AMR in their courses; however, 96.2% thought it necessary to set up special course on antibiotics<br>• Nursing students who had discussions about AMR in their courses had better KAP and nursing students with higher average scores had better knowledge and were more aware of high-risk practices.
**Malaysia**	Haque et al., 2019 (315)	• Assess university students' knowledge and beliefs about and their use of antibiotics<br>• Cross-sectional study among medical and non-medical students using a pre-validated instrument.<br>• 674 students took part - response rate 97.73% of medical students and 100% of students from nonmedical faculties	• Significantly higher levels of knowledge and more up-to-date beliefs was seen among surveyed medical compared to non-medical students (p-value of <0.001). However, only 3.3% had a high level of knowledge about antibiotics and only 43.9% held up-to-date beliefs regarding antibiotic usage<br>• There were concerns that participants did not compete the course of prescribed antibiotics (36.5%) with 20.1% not remembering – 18.1% because they felt better<br>• Self-medication with antibiotics was practiced among 14.1% of participants principally for common colds (31.7%), coughs (33.7%), genitourinary infections (5.8%) and sore throats (1.5%)
	Haque et al., 2019 (316)	• Gain information on antibiotics among medical students<br>• Cross-sectional study using a validated questionnaire<br>• 204 questionnaires were completed (99.0% response rate)	• Perceived knowledge regarding antibiotics was seen as good among participating students with more than 88% of students totally agreeing or agreeing that penicillin or amoxicillin are antibiotics and antibiotics are useful for bacterial infections<br>• In addition. 81.4% totally disagreed or disagreed that 'Aspirin is an antibiotic', 84.8% that paracetamol is an antibiotic, 65.6% that antibiotics are useful for viral infections and 70.6% that antibiotics are indicated to reduce any kind of pain and inflammation"<br>• 77.5% also stated that they do not take antibiotics to treat a cold or sore throat and 60.8% for a fever<br>• 87.7% had also heard about ABR, with 87.3% totally agreeing or agreeing that ABR is a phenomenon for which bacteria lose their sensitivity to an antibiotic, and 85.8% that misuse of antibiotics can lead to a loss of sensitivity of an antibiotic<br>• Overall, senior (nearing graduation) medical students had better knowledge than junior colleagues
	Abubakar et al., 2020 (277)	• Evaluated knowledge and self-confidence regarding ABR, appropriate antibiotic therapy, and AMS among final year pharmacy undergraduate students in three Asian countries including Malaysia<br>• Cross-sectional study using a 59-item questionnaire developed after a literature review and validated<br>• 211 students took part (response rate 77.8%).	• 80.1% of participating students recognised that it was inappropriate to treat diarrhoea with antibiotics, and 75.8% strongly disagreed/ disagreed that AMR is not a serious problem<br>• However, only 23.2% were able to select appropriate antibiotics to treat UTIs and only 26.1% felt that it was inappropriate to treat URTIs with antibiotics<br>• Approximately 70% of students understood the role of pharmacists in ASPs. However, 95% stated they needed more training in antibiotics and AMR, with only 23.2% having formal training in AMS and 88.1% stating that their knowledge of AMS was only poor to average

	Haque et al., 2022 (317)	• Assess the relative effectiveness of various instruction/teaching delivery modes among medical students in gaining and retaining knowledge of medicines and prescribing including antibiotics<br>• Cross sectional study using a validated questionnaire<br>• 140 students took part in the principal study (90.3% response rate)	• Year-IV and Year-V Medical participating students had higher knowledge than Year-III students regarding confidence in antibiotic prescribing with Year-V students having significantly more knowledge than Year-IV students<br>• Almost all knowledge and attitudes towards AMR were lower among Year-III vs. Year-IV and Year-V students<br>• Overall, students felt that the trust in antimicrobial prescribing guidelines and adherence to these guidelines was essential to reduce the risk of AMR

*World Bank Classification; ABR = Antibacterial Resistance; AMR: Antimicrobial Resistance; AWaRe = Access, Watch, Reserve antibiotics; FGD = Focus Group Discussions; HCP = Healthcare Professional; HCW = Healthcare Worker; PHC = Primary Healthcare; KAP: Knowledge, Attitude and Practice; URTI: Upper Respiratory Tract Infection

Table S13: Knowledge, Attitude and Practices, antibiotics, antimicrobial resistance and antimicrobial stewardship – WHO African Region Patients/ Public

Country	Author and Year	Objectives, Study Design and Population	Summary of the Findings
**Low Income countries***			
**Burkina Faso**	Sariola et al., 2022 (318)	• Focus on key areas surrounding the implementation of the NAP in Burkina Faso<br>• Interviews and FGDs with key stakeholders including patients/ public<br>• 'Drug bag' method regarding identifying antibiotics	• No details of the number of households approached<br>• Sources of antibiotics among households to treat infections included PHCs (34%), informal sellers (27%), private sellers including pharmacies (29%) and hospital drug stores (12%)<br>• To conserve costs, it was common for patients when purchasing antibiotics to stop taking them when they recovered ready for use the next time
**Central African Republic**	Eibs et al, 2020 (19)	• Address the knowledge gap regarding antibiotic use through exploring antibiotic prescription and consumption habits alongside key drivers<br>• Interviews with 24 MSF staff and patients as well as 81 personnel in FGDs using a standardized questionnaire	• Marginalised groups/ members of Muslim communities sought 'powerful' medicines vs. other groups<br>• Mobile populations, e.g. cattle herders, typically visited pharmacies for medicines finding it difficult to leave their animals alone for a whole day to seek healthcare<br>• However, concerns with the knowledge level of drug sellers, who easily adapted their prescribing/ dispensing habits to meet patients' demands for 'powerful' medicines<br>• Self-medication was also driven by a desire for quick relief and easy access to medicines, e.g. antibiotics to treat RTIs
**Democratic Republic of Congo**	Eibs et al, 2020 (19)	• Address the knowledge gap regarding antibiotic use through exploring antibiotic prescription and consumption habits alongside key drivers<br>• Interviews with 40 MSF staff and patients as well as 41 personnel in FGDs using a standardized questionnaire	• Patients typically sought antibiotics based on previous experiences to avoid costs and lengthy admittance procedures, with all pharmacies studied admitting to dispensing antibiotics without prescriptions – exacerbated by certain medicines typically not available in health centres<br>• Self-medication was prevalent driven by a desire for quick relief, easy access to antibiotics, with antibiotics typically cheaper from community providers than hospital pharmacies

			• However, concerns that patients are only dispensed the quantity of antibiotics they could afford at that time – potentially purchasing additional antibiotics later<br>• Concerns also with lack of knowledge of the prescriptions provided
	Shembo et al., 2022 (319)	• Explore community knowledge of antibiotics and practices regarding their use<br>• Qualitative study with in-depth interviews using a semi-structured interview guide developed following a literature review<br>• 18 participants were interviewed	• All participants were familiar with the term “antibiotics”, with most describing antibiotics as medicines that were used to treat infections<br>• However, inappropriate use of antibiotics was common – with antibiotics being used to treat a range of conditions including fever, coughs, profuse sweating and pain during urination, enhanced by unsanitary living conditions which exposed patients to various types of infections<br>• Of concern as well is that antibiotics were typically stopped when symptoms disappeared
**Eritrea**	Ateshim et al., 2019 (320)	• Ascertain the current status of purchasing of antibiotics without a prescription and the rationale among the community members<br>• Face-to-face interviews with 577 participants (99.5% response rate) using a structured questionnaire based on a similar previous study	• 45.1% prevalence rate of self-medication with antibiotics among interviewed participants<br>• Principal conditions for self-medication included wound infections - 17.9%, sore throats - 13.9%, aches and pains - 12.5%, tonsillitis - 12.41% and coughs - 9.2%<br>• Principal reasons for self-medication included previous successful experience with antibiotics (34.4%), the illness seen as too minor to seek medical attention (25.7%) and the need for quick relief (25%)<br>• Of concern is that only 42.6% of participants completed their course of treatment as recommended and 23.9% discontinued their antibiotics when symptoms disappeared. Overall, 84.7% of participants were seen to have inadequate knowledge regarding antibiotics
	Russom et al., 2021 (321)	• Measure KAP of antibiotics and ABR and their determinants in an urban population to provide future direction to key stakeholder groups<br>• Population-based cross-sectional study using a structured questionnaire based on previous studies<br>• 2477 patients/ public were successfully inter-viewed (97.4% response rate)	• Only 36.6%–49.5% of the study population correctly reported the indication of antibiotics for common illnesses<br>• 63.4% believed that antibiotics can be used to treat viral infections, 61.8% for watery diarrhoea, 58.9% to treat a common cold and 54.2% to treat a dry cough<br>• 18.8% who used antibiotics did not complete the full course principally because their condition had improved<br>• There were also concerns with the overall knowledge regarding antibiotics among the public/ patients. However, 78.2% of the study population correctly re-ported that ABR is an issue that needs addressing
**Ethiopia**	Bogale et al., 2019 (322)	• Assess KAP regarding self-medication with antibiotics among community residents<br>• Cross-sectional study using a questionnaire developed from previous validated question-naires<br>• 595 residents participated (98.3% response rate)	• 58.7% of participating residents had no knowledge regarding the antibiotics they had used and 63.4% were unaware that antibiotic overuse can cause ABR<br>• 45.7% of residents incorrectly believed that antibiotics and anti-inflammatory drugs were the same, 49.9% incorrectly believed that antibiotics can be used to treat common colds and 36.0% believed it is acceptable to stop taking antibiotic when symptoms improve<br>• 53.3% of participating residents also incorrectly believed that antibiotics can be purchased without a prescription
	Mengesha et al, 2020 (323)	• Evaluate public awareness, attitude, and practice concerning antimicrobial use and AMR<br>• Cross-sectional study using a standardized questionnaire	• Encouragingly, 72.5% of respondents finished the full course of antibiotics prescribed, with the majority of the remainder stopping when symptoms improved<br>• 59.4% had heard the term AMR and of these 50.4% were aware of the problems of ABR

		• 374 participants took part (97% response rate)	• Overall, 47.6% of respondents realized key risk factors for AMR including overuse/underuse of antibiotics, failure to complete the full course and sharing antibiotics with others
	Dejene et al., 2022 (324)	• Assess the KAP of residents regarding antimicrobial use and AMR<br>• Cross-sectional study using a semi-structured questionnaire developed following a literature review and validated<br>• 400 randomly selected residents took part	• Encouragingly, 48% of participating residents had moderate knowledge, 35% high knowledge and only 17% had low knowledge about AMR and antibiotic usage<br>• However, 51.5% agreed that antimicrobials help with the recovery of a cold and 46.8% that antimicrobials are effective against the virus<br>• In addition, 43.3% prefer to use antimicrobials when they have a minor illness to feel better more quickly and 39.5% stated they do not complete the full course of antibiotics if they feel better
	Demissie et al., 2022 (325)	• Assess the prevalence of self-medication with antibiotics, and its associated factors among the community<br>• 826 respondents took part using a pre-tested questionnaire	• Prevalence of self-medication with antibiotics in the past 12 months among community members was 38.9%<br>• Principal conditions for self-medication with antibiotics were aches and pains (15.5%), typhoid and typhus (8.1%), coughs (6.3%) and diarrhoea (4.3%<br>• Principal antibiotics were amoxicillin (22.1%), ciprofloxacin (9.7%), doxycycline and ciprofloxacin (8.7%)<br>• Principal reasons for self-medication were prolonged waiting times to get seen in clinics (39.9%), treatment of previous similar symptoms (19.8%), and lack of time to visit health institutions in the first place/ busy day program (16.4%)
	Simegn et al., 2022 (326)	• Assess the level of awareness and knowledge of AMR and key factors associated with knowledge among adults<br>• Community-based cross-sectional study using a semi-structured interview-based questionnaire adapted from the WHO level of AMR public awareness survey and another study<br>• 407 participants took part (99.5% response rate)	• 58.2% of respondents were aware of the existence of ABR, 39.8% were aware of the existence of AMR, 36.6% were aware of the existence of ABR, with only 15.7% unaware of the existence of such terms<br>• 70.8% of respondents were aware that sharing antibiotics with others was a risk factor for AMR, with AMR heightened by failure to complete the course of antibiotics (61.4% of respondents) as well as over/ under use of antibiotics (43.7%)<br>• Good knowledge regarding AMR was associated with a number of factors including college and above educational level and getting advice from HCPs about how to take antibiotics
	Jifar et al., 2024 (327)	• Assess key factors associated with self-medication practices during the recent COVID-19 pandemic<br>• Community-based cross-sectional study design among 415 patients (98.3% response rate) visiting community pharmacies using a piloted structured questionnaire based on the literature	• 50.6% reported engaging in self-medication for symptoms of COVID-19 with 49% seeking self-medication information from the internet or social media<br>• The most frequently used classes of medicines for COVID-19 treatment or prevention were analgesics (42.4%), cold medicines (29.5%) or antibiotics (26.7%), with antibiotics principally used for RTIs (14.3% of patients), coughs (5.8%) or sore throats (5.5%)<br>• Fear of contracting the virus was the principal reason for self-medication (31.5%) with a minority stating concerns with extended waiting times at clinics
	Muhummed et al., 2024 (328)	• Examined KAP regarding AMR in a rural community<br>• Mixed method approach using surveys and FGDs with a semi-structured interview guide and survey questionnaire	• 95.9% of participants correctly agreed with the definition of antibiotics<br>• However, 89.5% of participants believed that antibiotics can be used to treat watery diarrhoea, 71% a fever, 68.2% a common cold and 57.7% viral infections<br>• 39% of participants had heard of AMR. However, 74.3% believed that AMR could not be transmitted between humans and only 4% had heard of ASPs

		• 362 people participated in the survey and there were 12 FGDs	• 52.4% of participants had used antibiotics in the past month and 17.1% in the past 6 months. Common infectious diseases included URTIs (75.5%), UTIs (72.7%), diarrhoea (71.5%) and a common cold (52.5%)<br>• In the FGDs, most participants had a limited knowledge of AMR as well as factors involved in the spread of AMR. In addition, most found it difficult to adhere to the prescribed antibiotic course
**Malawi**	Sambakunsi et al., 2019 (329)	• Describe the KAP of residents regarding self-medication with antimicrobials<br>• Mixed method study with two FGDs and a survey using structured questionnaires<br>• 15 participants overall in the focus groups and 105 in the KAP survey (95% response rate)	• Concerns with knowledge with 92.4% of participants believing antimicrobials could stop a fever, 74% unable to differentiate antimicrobials from other categories of medicines and over 54% wrongly believing that antimicrobials are effective in treating common colds<br>• Concerns also with practice, with 53% stating they would use antimicrobials to treat URTIs and only 41% agreeing they would complete a course of antibiotics even if they symptoms were improving. In addition, 67.6% of respondents indicated they would keep antimicrobials at home for emergencies<br>• However, 51% agreed that the unnecessary use of antimicrobials would reduce their future usefulness and 54.3% correctly stated that resistant bacteria could affect them as well as other patients.
	Dixon et al., 2021 (21)	• Increase understanding regarding the patterns of antibiotic prescribing and use and the reasons behind this<br>• Data collection involved household surveys using a structured questionnaire<br>• 1811 households participated of which 51.0% were in Malawi, 24,8% in Uganda and 24.0% in Zimbabwe and 24.8%	• Amoxicillin was overall the most prescribed antibiotic (range: 13.5% in Uganda – 53.0% in Zimbabwe), followed by cotrimoxazole (range: 8.1% in Uganda – 37.9% in Malawi) and metronidazole (range: 6.1% in Malawi –28.5% in Uganda), with a greater proportion of households frequently using one or more antibiotics from the 'Access' group (range: 68.8% in Zimbabwe –94% in Uganda) than the 'Watch' group (up to 25.0% in Zimbabwe).<br>• Catastrophic healthcare costs could be mitigated through borrowing medicines to treat acute illness and reciprocating at a later date with leftover antibiotics<br>• Purchasing antibiotics apart from cotrimoxazole (typically prescribed for a range of illness in Malawi apart from HIV as provided by donors) was common from private pharmacies/ informal sellers as stockouts in clinics were common even though medicines provided free in public facilities
	Machongo et al., 2022 (330)	• Explore the experiences of caregivers of children under 5 years regarding antibiotic use<br>• In-depth interviews using a structured questionnaire<br>• 16 caregivers participated	• Most caregivers did not know about the name "antibiotics" with limited knowledge about ABR. However, some were aware that at some point antibiot-ics would stop working if used inappropriately<br>• Caregivers typically indulged in self-medication with antibiotics, with antibiotics typically used to treat coughs, sore throats and diarrhoea.<br>• Sources of antibiotics included buying antibiotics from a store (hawker, drug store or pharmacy), using leftover antibiotics at home and sharing antibiotics with friends and relatives – the latter enhanced by the high costs of antibiotics versus average wages. As a result, some caregivers stop administering antibiot-ics when a child's condition improves
	Limwado et al., 2024 (331)	• Assess the prevalence of antibiotic self-medication and knowledge of ABR among community members	• 71.2% reported self-medicating in the past 6 months, with 69.5% of these partic-ipants self-medicating at least twice. Of concern is that 60% of patients pre-emptively asked for antibiotics, subsequently sold without a prescription<br>• Amoxicillin (61.1%) and cotrimoxazole (29.6%) were the most used antibiotics, with 53.1% of participants reusing leftover antibiotics from health facilities, with

		• Cross-sectional study utilizing quantitative survey data collection methods with interviews<br>• 531 participants responded to household questionnaire and 39 drug retailers were interviewed	employed participants significantly more likely to self-medicate. Of concern is that 57.7% who self-medicated stopped their antibiotics when their symptoms improved<br>• Common symptoms for self-medication included a cough (29.9%), sore throat (28.6%), and aches and pain (28.6%), with convenience (31.5%) and confidence (26.7%) the principal reasons for self-medication with antibiotic<br>• Of concern was that awareness of ABR was low (16.1%), with 61.5% of drug retailers declining to reveal their source of antibiotics although 97.4% recognizing it is illegal to sell antibiotics without a prescription
**Mozambique**	Mate et al., 2019 (332)	• Investigate the KAP antibiotic use among adults living in peri-urban areas<br>• Cross sectional and semi-quantitative study using a semi-structured questionnaire<br>• 1091 participants took part	• 52.4% of participants had poor knowledge regarding the use of antibiotics with only 46.4% having reasonable knowledge<br>• 28.7% of participants did not complete their course of antibiotics<br>• Principal reasons for non-prescribed antibiotics were a perception that there was no need to attend a health facility as only a minor illness (26.8%), 7.7% someone else's advice (7.7%) and 6.2% that symptoms were similar to a previous infectious disease episode
	Cambaco et al., 2020 (333)	• Describe knowledge and practices of antibiotic use among the general population<br>• Mixed method approach with in-depth interviews and FGDs using a structured questionnaire<br>• 16 in-depth interviews and 4 FGDs	• Mixed responses from participants regarding familiarization with antibiotics; however, the majority had heard of the term<br>• Encouragingly, participants recognized the importance of taking the full course of medications and finalizing treatment as instructed with only a few respondents reported quitting the medication before the prescribed treatment was completed<br>• However, concerns that participants believed antibiotics could be used to treat a variety of infections including coughs, fever, body pain, pain in the uterus, headaches and illnesses related to bones<br>• In addition, participants consistently stated they had never heard of ABR. Among the few that did recognize the term, they believed ABR happens when the disease or body resists the effects of antibiotics
	Torres et al., 2020 (162)	• Identifying the commonly used antibiotics dispensed without a prescription and the principal health conditions<br>• Cross-sectional study involving both individual and FGDs using a structured questionnaire<br>• 32 patients visiting pharmacies and 17 pharmacists participated	• 93.75% of surveyed patients admitted to purchasing antibiotics without a prescription exacerbated by the long waiting times at clinics especially when symptoms are mild<br>• The most commonly purchased antibiotics were amoxycillin (also known as 'two colours medicine'), cotrimoxazole and co-amoxiclav<br>• Sore throats, fever, coughs, influenza, RTIs, UTIs and vaginal discharge were common conditions where antibiotics were purchased, with requests for antibiotics for STIs increasing
**Uganda**	Lanyero et al., 2020 (163)	• Determined the prevalence and factors associated with antibiotic use in managing ARIs in children in rural communities<br>• Cross-sectional survey among households using a questionnaire adapted from previous studies<br>• 856 households took part (98.9% response rate)	• 52.7% of caregivers visited health centres for their children with 47.3% self-medicating their children<br>• 60.2% of children were treated with antibacterials; the symptoms most commonly associated with antibiotics included runny noses (58%), cough (67%), fever (61%), fast breathing (79%), having symptoms of ARIs with diarrhoea (54%)<br>• The most commonly used antibiotics were amoxicillin (55.2%), cotrimoxazole (15.4%) and metronidazole (11.4%)

			• Determinants of antibiotic use included getting treatment from a health facility, households located in peri-urban area and a child having a cough
	Dixon et al., 2021 (21)	• Increase understanding regarding the patterns of antibiotic prescribing and use and the reasons behind this<br>• Data collection involved household surveys using a structured questionnaire<br>• 1811 households participated of which 51.0% were in Malawi, 24,8% in Uganda and 24.0% in Zimbabwe and 24.8%	• Amoxicillin was overall the most prescribed/used antibiotic (range: 13.5% in Uganda – 53.0% in Zimbabwe) - followed by cotrimoxazole (range: 8.1% in Uganda –37.9% in Malawi) and metronidazole (range: 6.1% in Malawi –28.5% in Uganda), with a greater proportion of households frequently using one or more antibiotics from the 'Access' group (range: 68.8% in Zimbabwe –94% in Uganda) than the 'Watch' group (up to 25.0% in Zimbabwe)<br>• Use of metronidazole was common in Uganda as could be purchased relatively cheaply by patients and made symptoms, if not completely better, at least tolerable<br>• Catastrophic healthcare costs could be mitigated through borrowing medicines to treat acute illness and reciprocating at a later date with leftover antibiotics, with purchasing of antibiotics common from private pharmacies/ informal sellers as stockouts in clinics common and high co-payments
	Lanyero et al., 2021 (334)	• Describe and validate caregivers' reported use of antibacterials by their children prior to seeking care<br>• Cross sectional study among children under five years seeking care using a researcher administered questionnaire<br>• 355 study participants with a complete set of data	• 4.4% of participants reported giving children antibacterials prior to visiting the healthcare facility. However, LC-MS/MS detected antibacterials in blood and urine samples in 63.7% of the children<br>• The most common antibacterials detected were cotrimoxazole (29%), ciprofloxacin (13%), and metronidazole (9.9%)<br>• Overall, low validity of caregivers' reports on prior intake of antibacterials among their children
	Nyeko et al., 2022 (335)	• Investigate pre-hospital antibiotic use among febrile children presenting to a tertiary health facility<br>• Cross-sectional study using a pre-tested questionnaire<br>• 210 children were recruited	• 39.5% of the children with fever had taken antibiotics prior to the hospital visit, 55.4% of which were on a self-medication basis<br>• The most commonly used antibiotics were amoxicillin (39.8%) and erythromycin (21.7%)<br>• Key factors associated with pre-hospital antibiotic use included residence; distance from the nearest health facility; caregivers' gender; cough; duration of fever and caregivers' perception that fever, cough, diarrhoea and any infection are indications for antibiotics.
	Kiragga et al., 2023 (165)	• Assess the extent of self-purchasing of medicines, especially antibiotics, pre- and during COVID-19<br>• 19,285 customers were surveyed using electronic sales data	• Self-purchasing of antibiotics was high pre-COVID-19. Out of 19,285 customers surveyed, antibiotics purchased included azithromycin (n = 6077), ciprofloxacin (n = 6066) and erythromycin (n = 997)<br>• Antibiotics purchased to treat COVID-19 included azithromycin, erythromycin and ciprofloxacin as well as supplements including Zinc and vitamin C and dexamethasone<br>• The number of patients purchasing azithromycin during the COVID-19 pandemic increased (19.7%) but decreased for erythromycin (-35.8%) and ciprofloxacin (-58.8%)
**Zambia**	Wildbret et al., 2023 (336)	• Define infant and illness characteristics associated with self-purchasing of antibiotics from the informal sector for infants up to fifteen months of age	• Most illnesses where medicines were purchased were for respiratory disease (n = 1123), gastrointestinal disease (n = 391) and general malaise, fever, or and headache (n= 185), with medicines purchased in the informal sector most frequent for 'malaise, fever, or headache'

		• Weekly in-person surveys were conducted<br>• Overall, the analysis included 439 participants accounting for 1927 illness episodes over 14 months	• Medicines mostly acquired in pharmacies (62.4%) or over the counter/chemical shops (29.1%), with the proportion of illnesses where medicines were acquired in the informal health sector increasing with increasing distance from the study clinic<br>• Antibiotic usage was common - used in 50.8% of all illnesses
	Mudenda et al., 2024 (337)	• Assess KAP regarding antimicrobial use and AMR among community members<br>• Cross-sectional study using a structured questionnaire<br>• 369 participants completed the questionnaire	• 38% had good knowledge in terms of for instance disagreeing they kept antibiotics for future use, 58% had positive attitudes in terms of for instance agreeing it is important that patients are advised about complying with antibiotics when they are prescribe/ dispensed, and 52% good practices regarding antibiotic use and AMR<br>• 66.7% had heard about antibiotics; however, only 33.6% had heard about AMR and 48.2% were neutral about AMR being a public health issue<br>• In addition, 52.8% indicated they did not take antibiotics without consulting a doctor or pharmacist
	Kampamba et al., 2024 (338)	• Evaluate knowledge and practices towards short-term antibiotic use on self-reported adherence among patients visiting hospitals<br>• Cross-sectional study using a questionnaire based on previous studies<br>• 335 patients took part (87.2% response rate)	• Overall, variable knowledge regarding antibiotics. 72.2% knew amoxycillin was an antibiotic and 66.0% that antibiotics are effective against bacterial infections; however, only 45.4% knew antibiotics were ineffective against viral infections an 43.6% that antibiotics are not indicated to reduce pain. Inflammation<br>• However, 58.2% had taken an antibiotic for a common cold, 70.5% had used leftover antibiotics and 74.0% had bought antibiotics without a prescription<br>• Alongside this, 57.6% displayed low adherence towards antibiotic use, e.g. 66.9% stopped taking antibiotics when they felt better
	Ngoma et al., 2024 (339)	• Assessing antibiotic consumption and associated factors among residents in selected communities<br>• Cross-sectional study using a structured questionnaire adapted following a pilot study<br>• 2038 residents took part	• Overall, 45.6% of participating residents used antibiotics correctly; however, 52.6% stopped taking antibiotics when they felt better<br>• Overall, the proportion of appropriate use of antibiotics was estimated at 40.9%, i.e. those who obtained antibiotics using prescriptions and consumed them as guided, while 59.1% utilized antibiotics inappropriately<br>• The appropriate use of antibiotics was associated with a number of factors. These included being female, being aged 35 years and above, attaining secondary school or tertiary education and being aware that antibiotics are not the same as painkillers
**Zimbabwe**	Dixon et al., 2021 (21)	• Increase understanding regarding the patterns of antibiotic prescribing and use and the reasons behind this<br>• Data collection involved household surveys using a structured questionnaire<br>• 1811 households participated of which 51.0% were in Malawi, 24,8% in Uganda and 24.0% in Zimbabwe and 24.8%	• Amoxicillin was overall the most prescribed/used antibiotic (range: 13.5% in Uganda – 53.0% in Zimbabwe) - followed by cotrimoxazole (range: 8.1% in Uganda –37.9% in Malawi) and metronidazole (range: 6.1% in Malawi –28.5% in Uganda), with a greater proportion of households frequently using one or more antibiotics from the 'Access' group (range: 68.8% in Zimbabwe –94% in Uganda) than the 'Watch' group (up to 25.0% in Zimbabwe)<br>• Amoxicillin was typically seen as the 'drug of choice' for a range of conditions including pneumonia, sinusitis, otitis media and UTIs, with expectations that antibiotics would be prescribed during consultations<br>• Catastrophic healthcare costs could be mitigated through borrowing medicines to treat acute illness and reciprocating at a later date with leftover antibiotics, with purchasing of antibiotics common from informal sellers due to the high costs in pharmacies

Lower-Middle Income countries*			
**Benin**	Aika et al., 2023 (340)	• Evaluate and compare the impact of two educational interventions on knowledge of antibiotics and their use among paediatric home caregivers<br>• Cross-sectional study conducted in the paediatric out-patient department using a structured questionnaire administered to 60 paediatric caregivers (30 in each group)<br>• The intervention included one-to-one educational inputs or Group inputs	• Many paediatric caregivers had poor knowledge regarding antibiotics and their use which improved significantly after education<br>• One-on-one education had more of an impact than group education<br>• Improvements seen in key issues including the following:<br>○ Before education - 81.7% of participants believed that antibiotics could treat malaria infection – reduced to 13.3% after education)]<br>○ Before education - 71.7% of respondents agreed that antibiotics could be used to treat all kinds of diarrhoea and 65% that antibiotics in powder form can be reconstituted with hot or warm water before use – reduced to 11.7% after education<br>○ Overall, mean knowledge scores regarding antibiotics improved significantly after educational initiatives among the sixty participants – greater for one-to-one education
**Cameroon**	Ngu et al., 2018 (341)	• Assess the prevalence of antibiotic self-medication and identify key factors contributing to self-medication among adults with RTIs<br>• Cross-sectional study using a validated questionnaire<br>• 308 participants	• 41.9% patients with RTIs reported taking antibiotics prior to visiting hospital – with most antibiotics obtained from pharmacies/ chemists without a prescription (81.4%)<br>• Cotrimoxazole (38.8% of participants) and amoxicillin (26.4%) were the most common antibiotics purchased<br>• Patients with a history of pulmonary TB were significantly less likely to self-medicate with antibiotics
	Penda et al., 2018 (342)	• Characterize the use of non-prescription medicines and describe illnesses of sick children before admission to the hospital<br>• Semi-structured interview guide used<br>• 295 children/adolescents too part (58.88% response rate)	• Self-medication was the initial most widely used option (74.1%) followed by a medical consultation (16.9%). Medical consultations (80.2%) and pharmaceutical advice (16.9%) were frequent 2nd recourses<br>• The most frequently used medicines were analgesics/antipyretics (47.6%), antimalarials (15.0%) and antibiotics (10.2%), with the main symptoms among children/ adolescents being fever (76.6%), vomiting (24.7%) and diarrhoea (22.7%).
	Elong Ekambi et al., 2019 (343)	• Determine KAP towards antibiotics use among patients visiting pharmacies<br>• Cross-sectional study using a structured questionnaire<br>• 1192 patients took part	• 33.7% of interviewees had recently purchased antibiotics, and of these 47% had purchased without a prescription<br>• Encouragingly, 88.3% of those surveyed said antibiotics treat microbes and only 11.8% that they treat viral diseases and only 10.6% that treat pain<br>• Principal reason for self-medication was the treatment of a similar previous infection
**Ghana**	Ahiabu et al., 2018 (344)	• Document treatment of acute illnesses and antibiotic dispensing practices among medicine sales outlets<br>• Recall visits to 12 households with detailed notes taken<br>• Dispensing practices observed in 3 rural and 3 urban medicine outlets	• Fever, abdominal and respiratory symptoms the most common causes of ill-health. 65% medicine-use events involved self-treatment - 40% of which involved antibiotics often without a prescription<br>• Purchasing of medicines without a prescription higher in rural vs. urban settings<br>• Penicillins were the most commonly dispensed antibiotics - 30% of antibiotic sales in urban outlets vs. 46% in rural ones
	Afari-Asiedu et al., 2018 (167)	• Assess differences between regulatory and community demands on the sale of antibiotics and explore how these differences could	• Abuse of antibiotics, including extensive self-medication is common, often for conditions that do not warrant antibiotics, e.g. stomach pains, hernias, wounds/sores, boils and headache with amoxicillin, tetracycline, metronidazole and chloramphenicol generally used to treat stomach aches

		be resolved to facilitate appropriate use of antibiotics in rural Ghana<br>• Mixed method approach involving 72 dispensers and community members in in-depth interviews and FGDs using structured questionnaires	• Generally, community members did not know what antibiotics are and the diseases they should be used for with for instance tetracycline and metronidazole poured into "akpeteshie" (local gin) to treat hernia and perceived stomach sores<br>• Overall, inappropriate antibiotic use was influenced by a general lack of knowledge on antibiotics among key stakeholders and identification of antibiotics by colours leads to confusion following requests and could lead to inappropriate antibiotic use which needs addressing
	Mensah et al., 2019 (345)	• Assesses the extent of self-medication among people in a rural setting<br>• Cross-sectional study using a questionnaire adapted from a previous study<br>• 361 participants completed the questionnaire (99.4% response rate)	• Antibiotics (32.1%) and analgesics (21.0%) were the most common medicines purchased without a prescription<br>• Antibiotics were mostly obtained from licensed chemical sellers (32.5%)<br>• One of the main reasons for self-medication was the influence of family and friends (32.7%). Other reasons included current bureaucracy (23%) and cost saving (22%)
	Afari-Asiedu et al., 2020 (346)	• Examine determinants of inappropriate antibiotic use at the community level in rural Ghana<br>• Mixed method approach involving 1100 randomly selected households over one year<br>• Four in-depth interviews and 2 FDGs to help explain survey results	• 53.2% of households had used antibiotics in the month prior to the survey<br>• 64.3% of antibiotics used were without a prescription and 45.3% treatment courses were not completed – with those without insurance more likely to use antibiotics inappropriately. This included those with lower income (and no insurance) buying incomplete courses initially on the understanding that they will come back and purchase the remaining course when they had funds again<br>• Distance to health facility (65.83%), trust in the medicine supplier visited (77.22%) and disease severity (76.8%) were important factors influencing the type of drug suppliers visited for healthcare
	Afari-Asiedu et al., 2020 (347)	• Explore inappropriate antibiotic use and current confusion regarding antibiotics with other medicines<br>• Mixed method with 15 in-depth interviews with HCPs and private dispensers and 8 FGDs involving 55 community members	• Antibiotics are were used to treat a range of diseases - including stomach pain, diarrhoea, gonorrhoeae, wounds/sores and headache - many indications are not caused by bacteria<br>• Where patients self-medicate with antibiotics – typically based on recommendations of family relatives, colleagues and friends<br>• Drug peddlers who move from house-to-house also teach people about specific antibiotics<br>• Of equal concern is that locally, antibiotics were often identified/ described with colours as patients do not know the names of these medicines.
	Efah et al., 2020 (348)	• Assess the awareness and knowledge of antibiotic usage and ABR among the general public<br>• Population-based survey using a structured questionnaire<br>• 632 people completed the questionnaire (74.3% response rate)	• Almost half of the respondents had ample knowledge that antibiotics are ineffective against colds, with a statistically significant difference between the various age groups and professions<br>• In addition, 75.9%, of the respondents had knowledge on bacteria's ability to become resistant to antibiotics. However, only 34.8% knew that ABR is transmissible among persons and only a few disagreed that leftover antibiotics can be saved for future use
	Jimah et al, 2020 (349)	• Assess the level of antibiotic knowledge, attitudes and consumption as well as ABR among community members<br>• Cross-sectional survey using a questionnaire adapted from the WHO<br>• 400 personnel took part	• Two-thirds and one-fourth of respondents respectively were not aware that antibiotics were inactive against viruses including colds/influenza and HIV/AIDS. 46% also answered incorrectly that antibiotics were effective against malaria<br>• Only 37% had heard of the term ABR and less than 10% had heard of "superbugs" or "AMR".

			• Encouragingly though 69.5% strongly agreed and 7.25% slightly agreed that patients should not keep left over antibiotics. However, 27.5% strongly agreed and 20.5% sightly agreed that there is not much they can do to stop ABR
	Kretchy et al., 2021 (350)	• Investigate the prevalence and risks of antibiotic self-medication among residents in a rural community<br>• Cross-sectional survey using a semi-structured questionnaire based on the literature<br>• 350 adults participated	• Overall prevalence of purchasing of antibiotics without a prescription was 36% among surveyed adults - helped by the ease of purchasing, problems seen as minor and seeking quick relief<br>• The predominant reasons for self-purchasing were GI problems (46%), fever (11%), skin problems (9%), GU tract problems (7%), URTIs (3%)<br>• Amoxicillin was the predominant antibiotic purchased (53.4% of occasions) followed by ampicillin - 23.3%, and tetracycline – 21%
	Otieku et al., 2023 (351)	• Evaluate knowledge of AMR and if access to knowledge helps mitigate against AMR<br>• Interview-based study with an intervention to improve knowledge<br>• 800 participants took part	• Most participants had a general knowledge of the health and economic implications of antibiotic use and AMR.<br>• However, 71% disagreed or disagreed to some extent that AMR may lead to reduced productivity/indirect costs with 87% disagreeing that AMR increases provider costs and 59% the costs for carers, patients/societal costs<br>• Encouragingly, participants exposed to the intervention were more likely to recommend restrictive access to antibiotics ($p<0.01$) and pay slightly more for a strategy to reduce their risk of AMR
	Vicar et al., 2023 (352)	• Assess the relationship between KAP and antibiotic use among households in urban informal settlements<br>• Survey study using a questionnaire based on the literature<br>• 660 households took part	• 44.1% of households had used antibiotics in the previous month, with 70.1% obtained without a prescription. Information on purchased antibiotics was principally obtained from either a friend/family member (24.7%) or a previous prescription (22.5%)<br>• Encouragingly, 53.6% had a good knowledge score regarding antibiotics and ABR with 92.4% knowing that antibiotics are used to kill germs and 59.7% that antibiotics will not help them recover quickly from a fever<br>• 82.4% were aware that the misuse of antibiotics is a major cause of ABR, 74.4% that ABR is a failure of antibiotics to kill germs and 63.8% that ABR is a serious health challenge in Ghana. 60.8% also stated that antibiotics should not be shared with household members for the same conditions<br>• However, 77.0% believed antibiotics can be used to cure influenza, 56.8% that antibiotics can be purchased with a prescription, 42.4% that antibiotics can be used to treat headaches/ coughs
	Hackman et al., 2024 (353)	• Determining the prevalence of antibiotic self-medication and the possible associated factors during the peak of the COVID-19 pandemic among adults<br>• Cross-sectional survey using a structured questionnaire<br>• 400 participants	• 76% of the surveyed participants had self-medicated with antibiotics in the previous 12 months during the COVID-19 pandemic – higher among males<br>• Convenience, avoiding long hospital queues, easy access, previous experiences and costs were some of the primary reasons for antibiotic self-medication<br>• Commonly self-administered antibiotics were azithromycin (34%), amoxicillin/clavulanic acid (22%), and metronidazole (16%) for perceived RTIs and GI tract infections.
**Kenya**	Kimathi et al., 2022 (172)	• Assess the extent of self-purchasing of antibiotics among patients with COVID-19 as well as KAP on self-medication<br>• Telephone-based survey using a coded questionnaire	• 23.6% self-medicated with antibiotics - this compares with feedback from 6 pharmacies allied to the University of Nairobi in the study of Opanga et al. where no pharmacy allied to the university dispensed antibiotics for patients with COVID-19 suggesting symptomatic relief (173)

		• 280 participants took part	• High levels of self-medication despite 92% of those interviewed thinking self-medication is unacceptable and associated with health risks<br>• However, 96.8% of participants believed that a full course of antibiotics should be completed as advised by HCPs
**Nigeria**	Ajibola et al., 2018 (265)	• Evaluate self-medication with antibiotics and knowledge of AMR among community members and undergraduate students<br>• Cross-sectional study using a structured pre-tested questionnaire<br>• 1230 people took part (84.8% response rate) – 872 community members and 358 students	• Weekly usage of self-medication with antibiotics was common – more common among undergraduate students (43%) than community members (26%)<br>• Prescription of antibiotics for use by a clinician, nurse, or pharmacist was 33.5%, 29%, and 25% respectively among undergraduate students vs. 57%, 20.4%, and 15.5% among the community members<br>• Antibiotics more likely to be purchased in patent medicine stores (40%) among students vs. local chemists/ pharmacies (48.4%) among community members<br>• Common conditions for self-medication with antibiotics among students were malaria (14.5%), typhoid (13.1%), stomach pains (12.7%) and diarrhoea (11.9%) and among community members dysentery/ diarrhoea (19%), infection (17%), typhoid (13%) and ear and throat pain (3.2%)<br>• Principal reasons for self-medication among undergraduate students and community members were long delays in the hospital (46% and 35%), cheaper not to go to hospital (26% and 19%) and the distance to the hospital (15% and 21%)
	Badger-Emeka et al., 2018 (354)	• Determine factors driving self-purchasing of antibiotics and the extent<br>• Cross-sectional study using a semi-structured self-administered questionnaire<br>• 400 adults participated	• 86.5% of participants had frequently taken non-prescribed antibiotics in the last 2–3 months<br>• 35.3% of participants stated they used antibiotics for treatment of their infectious disease with 39.8% for both treatment and prevention<br>• Principal conditions for antibiotic use were a cold/ cough (42.7%), sore throat (13.8%) or fever (16%)<br>• Penicillins (58%), quinolones (22%) and tetracycline (20.75%) were the most frequently purchased antibiotics<br>• Based on their personal experiences, 82% would recommend antibiotics to their family members and friend for similar infectious diseases
	Chukwu et al., 2020 (355)	• Assess the current level of awareness and knowledge of the Nigerian public regarding AMR<br>• A pre-tested and validated questionnaire was used among 482 participants	• Encouragingly, 76.7% of respondents believed that ABR is one of the biggest problems globally and 56.5% were familiar with the term “antibiotic resistance”,<br>• However, 66.8% had taken antibiotics in the last six months out of which 31.3% were without prescription with skin/ wound infections (77.2%) and bladder infection (68.9%) the most common conditions<br>• In addition, 51.5%, 48.5% and 44.4% of respondents erroneously identified measles, catarrh, colds/ influenza as infectious conditions requiring antibiotics.<br>• 26.1% of respondents also believed they didn’t need to complete the course as long as they feel better
	Aika et al., 2023 (340)	• Evaluate and compare the impact of two educational interventions (one-to-one and Group Education) on knowledge of antibiotics and their uses among paediatric home caregivers<br>• 60 caregivers took part – 30 in each group	• Overall positive responses with significant reductions in the number of caregivers who thought bacteria cause colds, fever, sore throats and catarrh in children, that antibiotics can treat all kinds of diarrhoea and dysentery and that antibiotics can treat malaria<br>• In addition, a significant reduction in those asking their physicians or pharmacist to prescribe antibiotics for their child even if they do not warrant these

			• Similar combined improvements with both educational programmes although greater improvement from one-to-one education in the 3 identified infections
	Akande-Sholabi, 2023 (356)	• Assess the knowledge, perceptions and factors associated with antibiotic misuse among consumers visiting selected community pharmacies<br>• Cross-sectional study using a questionnaire based on the literature and piloted<br>• 509 questionnaires were completed (97.9% response rate)	• 95.9% believed antibiotics can prevent bacterial growth<br>• However, 60.7% believed they can treat all infections including viral infections, 57.4% were unaware of ABR and only 14.7% had adequate knowledge regarding antibiotics<br>• 72.5% had used antibiotics in the last 12 months, with 28.3% obtaining these without a prescription<br>• Amoxicillin was the most used antibiotic (42.4%) followed by ampicillin (20.2%) and ciprofloxacin (19.4%), with antibiotics used to treat malaria (38.9%), colds/catarrh (19.8%), sore throats (18.5%) and fever/headaches (15.1%)<br>• Key factors influencing antibiotics misuse included delays in test reports, belief in antibiotics' quick relief and proximity of pharmacies to their house/ workplace
	Idoko et al., 2023 (357)	• Assess the knowledge of AMR among hospital out-patients<br>• Cross-sectional study among 400 outpatients using a structured questionnaire	• Overall concerns with the knowledge of outpatients, e.g.:<br>o Only 9.6% of females and 9.9% of males knew that ABR occurs when bacteria become resistant to antibiotics and they no longer work as well.<br>o Only 50.4% of females and 55.2% of males knew that infections are becoming increasingly resistant to current antimicrobials<br>o Only 26.8% of females and 27.9% of males gave a correct answer to 'AMR is only a problem for people who take antibiotics regularly'<br>• However, 55.3% of females and 57.0% of males gave a correct response to 'Over or underuse of antibiotic is a risk factor for AMR'<br>• Marital status, education and age impacted on knowledge scores
	Isah et al., 2023 (358)	• Determined predictors of antibiotic knowledge and attitudes toward antibiotic use and ABR among patients visiting community pharmacies<br>• Cross-sectional using a questionnaire revised from previous studies<br>• 964 patients took part	• Encouragingly, 76.6% of patients stated that bacteria would become less resistant to antibiotics after prolonged use<br>• However, concerns with their knowledge regarding antibiotics, e.g.:<br>o 50.9% believed antibiotics can relieve fever and pain<br>o Only 68% stated they used antibiotics on the recommendation of a health professional, with 68.3% stating they had used an antibiotic in the last 6 months<br>o 30% expected antibiotics to be concomitantly prescribed with an antimalarial when the need arises<br>o 22.1% believed antibiotics cure their cold/sore throat faster with 27.9% believing using leftover antibiotics was permissible when they had a cold or similar symptoms
**Senegal**	Bassoum et al., 2018 (359)	• Assess people's knowledge and opinions about antibiotic use and ABR in an urban setting<br>• Cross-sectional study using a questionnaire developed by the co-authors and extensively reviewed and piloted<br>• 400 participants took part (100% response rate)	• Encouragingly 83.8% of participants felt that high consumption of antibiotics can lead to ABR, with 78.3% believing the population of Senegal currently overuses antibiotics and 45.8% that they can play a considerable role in fighting ABR<br>• Of concern is that 72.3% of respondents believed that antibiotics are effective against a cough, 69.8% that antibiotics are effective against colds/influenza, 64.4% against a sore throat, 50.8% for fatigue and 41.8% for a fever<br>• 42.8% of participants would also stop taking antibiotics as soon they feel better

**Tanzania**	Horumpende et al., 2018 (360)	• Investigated the prevalence, determinants and knowledge of self-medication among residents of Siha District<br>• A cross-sectional study using pretested a semi-structured questionnaire<br>• 300 residents were surveyed	• 58% of surveyed residents admitted to self-medication with antibiotics, with the most commonly utilized antibiotics being amoxycillin (43%) and metronidazole (10%).<br>• The most common reasons for self-medication were emergency illness (24.00%), health facility charges (20.33%), proximity of pharmacy to their home (17.00%) and no reason (16.66%), with pharmacies commonly used as the first point of medical care (92% of respondents).<br>• However, 99.67% of respondents reported believed self-medication is not better than seeking medical consultation, 96.33% that self-medication with antibiotics increases ABR and 89.67% that this can result in complications<br>• Of equal concern is that the most common symptoms for self-medication with antibiotics were a cough (51.14%), headache/fever/malaria (25.57%) and diarrhoea (21.59%)
	Mboya et al., 2018 (361)	• Determine the prevalence of, and examine factors associated with irrational use of antibiotics among patients<br>• Cross-sectional study using a structured questionnaire adapted from the WHO<br>• 152 adults took part in the study	• 76.3% of purchases of antibiotics were without a prescription, 23% of participants buying incomplete course of antibiotic of less than 5 days.<br>• URTIs including coughs and influenza were the most common infection treated with antibiotics (48% of participants) followed by UTIs (17.8% of participants) and diarrhoea (9.9%)<br>• Participants with poor knowledge regarding how to use antibiotics had 5.5 higher odds of using antibiotics irrationally versus those with good knowledge
	Mboya et al., 2020 (362)	• Assessing knowledge of appropriate antibiotic use among patients in the Moshi municipality<br>• Cross-sectional study using exit interviews, with the questionnaire adapted from a validated WHO questionnaire<br>• 152 adults were interviewed	• Only 25% of surveyed adults had adequate knowledge about the use of antibiotics.<br>• 58.6% believed they should stop antibiotics only after finishing the dose as directed. However, 38.2% would stop antibiotics when they felt better and 50.7% thought it acceptable to share antibiotics with others<br>• Common conditions that were identified as treatable with antibiotics included sore throats - 62.5% of adults interviewed, influenza - 46.1% and fever - 40.1%. 48% of participants also agreed that antibiotics can be used to treat diarrhoea with only 17.1% disagreeing with this<br>• In addition, 65.1% felt they should request the same antibiotic if used previously to treat a similar illness<br>• Higher levels of education and having health insurance were associated with better levels of knowledge concerning antibiotic use
	Simon et al., 2020 (363)	• Assess the knowledge of parents/guardians of children under five regarding their knowledge of the appropriate use of antibiot-ics<br>• Cross-sectional study using a pre-tested structured questionnaire<br>• 730 pairs of parents/ caregivers and under-five age children took part	• 54.6% of parents/caregivers had a low level of knowledge regarding antibiotics<br>• 32.9% reported that they had stopped taking a full course of the antibiotic if symptoms improved<br>• In addition, only 22.7% of participating parents/ caregivers believed that their purchasing and use of antibiotics without a prescription/medical consultation could potentially enhance AMR

	Sindato et al., 2020 (364)	• Determine KAP regarding antimicrobial use and AMR among three districts in Tanzania<br>• Cross-sectional study using a semi-structured questionnaire<br>• 828 participants from the three districts were surveyed	• 98.6% of those surveyed were aware of antimicrobials and 99% reported to have used them, with awareness tending to increase with increased education<br>• Between 35.9% and 56.3% of participants strongly agreed that AMR occurs when antimicrobials are no longer able to treat infections, with between 34.0% and 54.7% strongly agreeing that if the disease-causing germs are resistant to antimicrobials, it can be very difficult or impossible to treat the infection<br>• In addition, between 34.1% and 55.6% of participants strongly agreed that AMR is mainly a problem for people who take antimicrobials frequently and between 38.4% and 64.1% strongly agreed that Tanzania is among the countries facing the challenges of AMR.<br>• However, antimicrobials were mainly used to treat coughs - 68.0% of those surveyed, UTIs - 53.4% and diarrhoea - 48.5%<br>• Overall, KAP scores were significantly influenced by increased participant's age and level of education
	Gabriel et al., 2021 (365)	• Assess the knowledge of rational use of antibiotics among consumers in Dar es Salaam<br>• Cross-sectional study using a structured questionnaire<br>• 960 participants surveyed	• 20.4% and 52.4% of participants respectively demonstrated good knowledge of rational antibiotic use and conditions that can be treated with antibiotics<br>• Encouragingly, 70.6% responded that they stopped using antibiotics after dose completion with only 26.2% saying they would stop antibiotics when they felt better<br>• However, 53.6% would request the same antibiotic if it had helped to treat a similar condition in the past and 42.3% are willing to use the same antibiotic if a friend or family member used the medication previously to treat similar signs and symptoms<br>• Alongside this, infections seen as treatable with antibiotics included influenza - 50.7% of participants, sore throats - 61.4% and UTIs - 60.5% and fever - 38.2%
	Mutagonda et al., 2022 (366)	• Determine KAP among parents/guardians as well as factors associated with inappropriate use of antibiotics among children in Tanzania<br>• Questionnaire design using a structured questionnaire based on the literature and piloted<br>• 2802 parents/guardians took part	• Overall, only 10.9% of participating parents/ guardians had good knowledge about antibiotics with 82.0% of respondents having poor practices regarding the appropriate use of antibiotics, e.g., only 20% felt that infectious diseases are becoming more difficult to treat with antibiotics, only 20% felt that antibiotics are not useful in treating diarrhoea, and only 30% felt that antibiotics are not useful in treating influenza or cough<br>• In addition, 30.3% of parents/ guardians would stop giving antibiotics to their children when there were improvements, with approximately 33% giving antibiotics to their children when they had a cough<br>• Having a university degree, good knowledge and positive attitudes towards antibiotics were significantly associated with appropriate use of antibiotics in children
**Upper-Middle Income countries***			
**South Africa**	Anstey Watkins et al., 2019 (190)	• Ascertain key issues regarding antibiotics including knowledge of antibiotics and AMR<br>• Semi-structured interviews<br>• 60 rural village residents participated (95.3% response rate)	• Antibiotics were used to treat a variety of bacterial infections including tonsillitis, UTIs and respiratory tract infections; however, none was obtained without a prescription. This is different to the findings of Mokwele *et al.* and Sono *et al.* where there was self-purchasing of antibiotics in community pharmacies driven by issues of affordabiliy and time taken to see a physician or their assistant in PHCs (186, 188)

			• Most interviewees though had not heard of the term ‘antibiotics’ before and had limited understanding of their purpose; however, when described using relevant Xitsonga words most participants were able to relate to this term.<br>• Some interviewees admitted not finishing their prescribed course when they felt better and either disposed of unused antibiotics into outdoor latrines or used them for the next infection<br>• The term AMR was unfamiliar to all interviewees; however, a number did understand the concept when translated into Xitsonga words
	Farley et al., 2019 (367)	• Describe the KAP of antibiotic use and ABR among patients attending PHCs - comparing public and private practice respondents.<br>• Cross-sectional survey using a structured questionnaire based on previous studies<br>• 782 patients took part – 379 public and private 403	• Mean knowledge scores were lower in public sector respondents compared to private sector (45% vs 60%) with higher knowledge scores associated with some protective attitudes and behaviour (e.g. must complete the full course) but also some negative ones particularly among public sector patients, e.g. I feel happy when prescribed antibiotics<br>• Encouragingly, 61% were concerned about ABR and believed antibiotics work less well in the future if overuse<br>• However, 72% believed incorrectly that the human body becomes resistant to antibiotics and 66% incorrectly stated antibiotics are good for treating viruses<br>• Public sector patients were more likely to believe antibiotics should be given on demand (39% vs 12%)
	Mokoena et al, 2021 (368)	• Assess knowledge and understanding of antibiotics and ABR among the minibus-taxi community, and to document indigenous antibiotic terminology used<br>• Semi-structured questionnaire adapted from the WHO and translated into commonly spoken languages<br>• 83 members of the minibus-taxi community took part	• 71% of participants understood the importance of taking antibiotics as directed<br>• However, 64% incorrectly believed it was acceptable to share antibiotics and 75% that ABR occurred in the human body rather than in bacteria<br>• There was also a common misconception that antibiotics could treat colds, influenza and fever<br>• Over 80% of participants were unfamiliar with antibiotic-related terminology as well as a significant variation in the indigenous terms used for antibiotics, indicating a need for standardized terminology. This is similar to the recent findings of Sono et al. (369)

*World Bank Classification; ABR = Antibacterial Resistance; AMR: Antimicrobial Resistance; AMS: Antimicrobial Stewardship; HCW = Healthcare Worker; FGDs = Focus Group Discussions; KAP: Knowledge, Attitude and Practice; PHC = Primary Healthcare; RTIs = respiratory Tract Infections; UTIs = Urinary Tract Infections; URTIs = Upper Respiratory Tract Infections

Table S14: Knowledge, Attitude and Practices, antibiotics, antimicrobial resistance and antimicrobial stewardship – WHO Eastern Mediterranean Region Patients/ Public

Country	Author and Year	Objectives, Study Design and Population	Summary of the Findings
**Low Income countries***			
**Yemen**	Alshakka et al., 2023 (370)	• Assess KAP towards antibiotic usage among the general public<br>• Cross-sectional study using a validated questionnaire and piloted<br>• 400 questionnaires were completed	• Whilst more than 77.5% of participants thought that antibiotics for common colds are not necessary and 81.5% that the overuse of antibiotics increases ABR, 88.8% of participants used antibiotics in all cases of fever, 58.3% thought that antibiotics could cure viral infections, and 65.5% believed antibiotics should be stopped as soon as the problem disappears<br>• In addition, 46.5% incorrectly thought that the early use of antibiotics in patients with a cough, running nose, or sore throat could be cured more quickly with antibiotics, with 62.7% admitting purchasing antibiotics for infections without a prescription in the last six months<br>• Unnecessary purchasing/ use of antibiotics in pharmacies is not helped by limited AMS practices among community pharmacies (371)
**Low-Middle Income countries***			
**Afghanistan**	Burtscher et al., 2021 (372)	• Principally to explore the perceptions and attitudes towards antibiotics among patients<br>• In-depth interviews with patients and caretakers using a structured questionnaire<br>• 351 participants completed the survey (100% response rate)	• Overall, poor knowledge among surveyed participants regarding antibiotics, with 56% believing antibiotics can cure illness quickly. Participants also commonly thought that antibiotics protect and strengthen the body, with physicians confirming patients often want antibiotics for their illnesses<br>• Surveyed patients typically saw antibiotics as the 'desired medication', and expected them to be prescribed by physicians for their infections, etc., whatever the cause<br>• Of equal concern is the 23% of participants said they sometimes stopped taking antibiotics before the course was finished. 20% of those surveyed used antibiotics to treat pain, 15% to treat a sore throat, and 14% an RTI<br>• In addition, patients who could not afford antibiotics did not purchase the full course, with 23% of those surveyed admitting they sometimes stopped antibiotics early. Participants also admitted to storing leftover antibiotics for the next infectious disease
**Egypt**	Kandeel et al., 2019 (90)	• Assess the effectiveness of campaigns to raise the awareness of physicians, pharmacists, and the public regarding antibiotic prescribing for ARIs<br>• This included an intensive educational campaign to reduce antibiotic use for URTIs as well as a targeted campaign for the youth on social media platforms<br>• Structured surveys with patients<br>• 1492 participants including 607 adults and parents of children (40.7% of the total)	• There was an overall improvement in the mean attitude scores for both surveyed parents of children and adults between the pre-and post-intervention periods - both patient groups improved their mean scores regarding persuading a doctor to prescribe antibiotics from 4.1 (pre-intervention) to 4.4 (post intervention), i.e. less likely to persuade doctors to prescribe antibiotics for ARIs<br>• Overall knowledge and attitude scores increased among parents of children and adult patients surveyed post the educational campaigns from 2.3 to 2.5 and from 2.4 to 2.6, respectively<br>• In addition, their understanding that many people are unnecessarily treated with antibiotics improved - mean scores increased from 2.9 to 3.3 and from

			2.7 to 3.4, respectively, e.g. parents of children gained knowledge that antibiotics are unnecessary when nasal discharge turns from yellow to green
	Alsayed et al., 2022 (275)	• Explore KAP regarding antibiotic use among key stakeholders in Arabic countries including adults in the community<br>• Cross-sectional descriptive study using a pre-validated survey instrument<br>• 6746 (23% of the total survey population) were adults<br>• These included adults from Egypt (7.6% of total adults)	• Encouragingly, 54.5% of adults thought ABR was a global problem reducing to 44.7% in their country, with 21.5% believing 21% - 50% of prescriptions for antibiotics are unnecessary<br>• Of concern is 58.8% of adults thought antibiotics are effective against sore throats, 57.6% against common colds, coughs and nasal congestion<br>• In addition, 48.3% believed that antibiotics are effective against fevers, 41.7% viral infections with a fever and 39.4% against viral infections<br>• Alongside this, approximately one-quarter of respondents stated that antibiotics will always be effective against the same infection in the future
	Maarouf et al., 2023 (373)	• Assess KAP towards antibiotic use as well as the effectiveness of a campaign to increase awareness regarding the safe use of antibiotics<br>• Structured questionnaires for both aspects and piloted<br>• 626 people participated (100% response rate)	• Encouragingly, the number of participants who believed more expensive antibiotics are more effective decreased post campaign from 34.7% to 18.6%, and the number believing they should take an antibiotic for a cold also decreased from 22.3% to 7%<br>• In addition, the number of participants who do not take antibiotics for a cold also increased post campaign from 14.1% to 25%, and the number stopping antibiotics when they feel better decreased from 32.5% of participants to 11.6%<br>• The number of participants who would ask a physician to prescribe an antibiotic even if they believed they were not needed also decreased from 17.5% to 7%<br>• Overall, the campaign made participants recognize that unwise antibiotic use is harmful to them or others and more participants would spread the word about ABR
	Ali et al., 2024 (374)	• Assess the KAP of the general population towards self-medication<br>• Cross-sectional study using a questionnaire developed from previous studies and piloted<br>• 1630 participants satisfactorily completed the questionnaire	• Painkillers were the most frequently self-medicated medicines in 60.74% of participants who practiced self-medication followed by antibiotics (32.13%) and antipyretics (28.61%)<br>• Self-medication was practiced typically either due to previous similar symptoms (59.08%), experienced with their health condition (39.48%), need a quick response (33.69%), lack of time (25.2%) or resources (economics) – 23.73%
**Morocco**	Elhaddadi et al., 2024 (375)	• Document parents' self-medication practices with antibiotics, determine key factors regarding their use in the paediatric population, and assess parents' knowledge of antibiotics and AMR<br>• Cross-sectional study using a structured questionnaire<br>• 460 families took part	• Encouragingly, 64% of parents had heard of ABR and multidrug-resistant infections, and half stated they would minimize the use of antibiotics by self-medication/ only take them when necessary<br>• However, self-medication with antibiotics was common (68% of families), encouraged more by mothers than fathers, with self-medication with antibiotic use more frequent in two to five year-old children than older children<br>• In addition, 78% believed RTIs should be treated with antibiotics, with self-medication with antibiotics used to treat a number of infections including coughs (43%), fever (24%), sore throats (9%) and diarrhoea (9%). Betalactams were the most used class of antibiotics (72%), followed by trimethoprim-sulfamethoxazole (15%)

			• Principal reasons for self-medication included saving consultation costs and time as well as the ease of obtaining treatment without a prescription
**Pakistan**	Akhund et al., 2019 (376)	• Evaluate the knowledge and attitude of the Pakistani population towards AMR<br>• Observational cross-sectional survey using a specifically designed on-line form<br>• 1132 forms completed	• Mixed findings with 56.3% feeling they knew they could contribute to decreasing AMR, 44.6% that frequent and unnecessary use of antibiotics decreases their effectiveness and 61.8% that bacteria could become resistant to antibiotics over time<br>• However, 73.9% felt it was acceptable to stop antibiotics when they felt better and 18.4% that it was correct to take antibiotics for colds and influenza<br>• There was a similar picture regarding attitudes towards antibiotics with only 13.9% stating they would not follow the recommended duration of treatment and only 4.4% would change their antibiotic dose without doctor consultation<br>• However, 41.3% used leftover antibiotics from their previous prescription, 61.8% suggested to their physician to prescribe antibiotics and 33.4% had purchased antibiotics without any prescription during the last year
	Atif et al., 2019 (377)	• Investigate KAP of the general public regarding the use of antibiotics in community pharmacy<br>• Mixed method study using a validated questionnaire<br>• 400 respondents (86.6%) agreed to take part	• 61.5% of respondents had a moderate level of knowledge regarding antibiotics, with 12% demonstrating poor knowledge<br>• However, 50.3% had poor attitudes towards antibiotics, with 56.6% believing that antibiotics could cure all types of infections and 92.3% did not complete their prescribed course, stopping when they felt better<br>• Identified inappropriate antibiotic practices included (i) lack of consultation with an HCP; (ii) purchasing of antibiotics without a prescription/ refilling a previous prescription; (iii) using left over antibiotics/ sharing of antibiotics with others; and (iv) improper dosage regimens/ early cessation of antibiotics
	Waseem et al., 2019 (93)	• Assess patients, physicians, and pharmacists' knowledge, attitudes and behaviour towards AMR in Pakistan using a specifically designed questionnaire<br>• 385 patients (community members) were included in the study	• 55.6% of approached community members possessed low knowledge about AMR, with 44.4% categorized as having high knowledge<br>• 61.8% of respondents had a bad attitude towards antibiotics and AMR, 35.1% had average knowledge and only 3.1% had a good attitude towards antibiotics<br>• Age and education impacted on knowledge scores
	Khan et al., 2020 (378)	• Assess KAP toward antibiotics and ABR among consumers<br>• 399 patients visiting community pharmacies took part using a validated questionnaire	• Typically, poor to moderate knowledge of antibiotics with 67.4% of participants having heard of the term 'antibiotics', and all participants aware of commonly used antibiotics by their trade names<br>• 61.7% had good knowledge regarding antibiotics and AMR as well as the fact that antibiotics kill bacteria<br>• However, 83.7% didn't know the term ABR and 71.4% were hearing it for the first time<br>• In addition, the majority of participants believed antibiotics can treat viral infections and that all types of infection can be cured with the help of antibiotics<br>• Poor practices regarding antibiotic use were also observed with 72.9% of participants stopping their antibiotics after feeling better and 57.6% storing left over antibiotics for the next infection
	Vaidya et al., 2020 (379)	• Evaluate the accuracy of self-reported antibiotic use among patients coming to hospital with suspected enteric fever for 3 or	• Antibiotics were detected in 39% of urine samples<br>• The correlation between measured and reported antibiotic use was modest

		more days in Bangladesh, Pakistan and Nepal vs. culture results from urine samples<br>• 2939 patients with suspected enteric fever were enrolled across 3 countries	• In Bangladesh and Nepal, reported antibiotic use was higher than the detected antibiotics across all age groups<br>• After adjusting for disease severity, patients with antibiotics in their urine were slightly more likely to be blood culture positive for enteric fever; however, the effect was not statistically significant<br>• Interestingly, only 2.4% of participants sought care at a pharmacy in Pakistan before coming to hospital vs. 56.7% in Bangladesh and 59.6% in Nepal
	Gillani et al., 2021 (380)	• Evaluate the KAP regarding antibiotics among the general public<br>• Tailored pre-validated and pre-tested Questionnaire<br>• 2106 members of the public (87.9%) completed the questionnaire	• Overall, 60.2% of participants had low levels of knowledge regarding antibiotic use - 35.4% believed antibiotics could cure viral infections and only 47.8% disagreeing that antibiotics are effective against colds and influenza with only 46.3% agreeing that it is not necessary to treat a cold with antibiotics<br>• In addition, only 32.6% disagreed that antibiotics should be discontinued as soon as symptoms disappear and only 46.7% that overuse of antibiotics causes AMR; however, 61.5% disagreed that antibiotics and anti-inflammatory medicines are the same<br>• Females had significantly higher levels of knowledge than males and city vs. village dwellers<br>• Overall, 68.4% of participants were classified as having a negative attitude towards antibiotic use with 59.6% reporting self-medication with antibiotics<br>• Pharmacists (46.2%) were the primary source of information regarding antibiotics
	Arshad et al., 2022 (381)	• Evaluate KAP related to antibiotic use and multi-drug resistant (MDR) pathogens among the general population<br>• Cross-sectional survey using specifically designed questionnaire<br>• 6,684 participants (87.6% of those approached) completed questionnaire	• 39.8% of participants scored above the mean score on knowledge. However, relatively few provided correct answers to the questions regarding antibiotics – greater among urban residents and males, e.g. only 42.4% of males and 26.5% of females disagreed/ strongly disagreed that antibiotics are effective against viruses and 34.2% of males and 21.3% of females that paracetamol is an antibiotic<br>• The mean MDR knowledge score was 1.98 (maximum of 3) with only 32.7% correctly answering the question whether MDR pathogens could only infect them in a hospital – higher scores again among urban residents and male<br>• Approximately half of the respondents correctly answered the question about ABR
	Khan et al., 2022 (382)	• Investigate KAP on antibiotic consumption, ABR and related suggestions among residents in conflicted zones in Pakistan<br>• Qualitative study with 20 in-depth interviews among patients seeking antibiotics in pharmacies using a specifically developed questionnaire	• All respondents replied they had heard the word ‘antibiotic’, with most interviewees defining ‘antibiotics’ correctly<br>• However, overall knowledge regarding ABR was poor<br>• Most participants were optimistic about antibiotics with 13/20 believing that antibiotics had a faster effect than other medicines.<br>• Most participants wanted to recover quickly for their daily activities and believed that antibiotics gave them extra strength, especially with infections<br>• Participants believed that every pharmacy should have a pharmacist present who can educate them regarding when and how to appropriately use antibiotics

	Saif et al., 2024 (383)	• Assess KAP regarding antibiotics, prevalence of self-medication and non-adherence as well as assess the impact of a video-based intervention program on those with poor knowledge<br>• Mixed method study involving structured questionnaires and 493 participants	• 78.4% had taken antibiotics in the past 6 months for a range of infections including ARIs and UTIs - 39.8% of antibiotics as self-medication with 42% (n=207) non-adherent to antibiotic treatment plan<br>• Lack of proper information from HCPs was seen as the most important barrier to antibiotic treatment adherence<br>• 68.4% had better knowledge of antibiotics than others with 50.7% having a positive attitude towards antibiotics<br>• The interventional video was shown to 31.64% of participants who lacked proper knowledge about antibiotic use and its associated problems. There appeared to be a significant improvement post-intervention in terms of KAP and adherence related to antibiotics which was encouraging
**Palestine**	Alsayed et al., 2022 (275)	• Explore KAP regarding antibiotic use among key stakeholders in Arabic countries including adults in the community<br>• Cross-sectional descriptive study using a pre-validated survey instrument<br>• 6746 (23% of the total survey population) were adults<br>• These included adults from Palestine (7.6% of total adults)	• Encouragingly, 54.5% of adults thought ABR was a global problem reducing to 44.7% in their country, with 21.5% believing 21% - 50% of prescriptions for antibiotics are unnecessary<br>• Of concern is 58.8% of adults thought antibiotics are effective against sore throats, 57.6% against common colds, coughs and nasal congestion<br>• In addition, 48.3% believed that antibiotics are effective against fevers, 41.7% against viral infections with a fever and 39.4% against viral infections generally<br>• Alongside this, approximately one-quarter of respondents stated that antibiotics will always be effective against the same infection in the future
**Sudan**	Eibs et al., 2020 (19)	• Address the knowledge gap regarding antibiotic use through exploring antibiotic prescription and consumption habits alongside key drivers<br>• Interviews with 38 MSF staff and patients as well as 148 personnel in FGDs using a standardized questionnaire	• Prescribers typically prescribed antibiotics immediately rather than adopt a ‘wait and see’ policy due to circumstances exacerbated by low levels of hygiene and sanitation<br>• All antibiotics were available without a prescription<br>• Concerns that patients stopped taking their antibiotics when they felt better and then either kept them for later or used them as shopping guidance<br>• In addition, some mothers shared their antibiotics with their children with similar symptoms exacerbated by affordability issues<br>• Most community members were also disinterested in how medicines work or even fear of knowing how they worked.
	Hussain et al., 2023 (384)	• Estimate the prevalence and predictors of purchasing antibiotics without a prescription among patients in Sudan<br>• Cross-sectional study design using a semi-structured questionnaire<br>• 1492 participants were surveyed	• The most common indications for self-purchasing were tonsilitis (55% - 56.1%), cough (45.5% - 44.6%), runny nose (34.5% - 41.6%) and nasal congestion (33.3% - 33.6%)<br>• In addition, 52% always or sometimes deliberately changed doses during treatment and 51% stop taking antibiotics when symptoms disappear<br>• 57% also stopped taking their antibiotics if they experience an adverse event
**Tunisia**	Ben Mabrouk et al., 2022 (385)	• Evaluate parents' knowledge concerning antibiotic use in children, and identify key factors associated with this<br>• Cross-sectional study using a structured questionnaire<br>• 354 parents took part	• 61.6% of participating parents had poor knowledge about antibiotics<br>• The frequency of non-prescription antibiotic use among children was 20.6%, with amoxicillin was the most used antibiotic (72.6%), and the main symptoms for non-prescription antibiotic use were sore throats (60.3% of cases), fever (34.2%) and flu-like symptoms (23.3%)<br>• The principal reason for self-medication was previous experience for the same symptoms (58.9%), with key parental factors associated with self-

			medication with antibiotics were advanced parent's age, the ability to name an antibiotic and low knowledge scores regarding antibiotics and AMR
**Upper-Middle Income countries***			
**Iran**	Nazari et al., 2022 (386)	• Investigate the frequency of self-medication with antibiotics in children under 6<br>• Population-based cross-sectional study using a structured questionnaire	• 1483 households took part (84.5% response rate)<br>• Annually self-medication with antibiotics was estimated 61.6%<br>• Girls were associated with less self-medication than boys'<br>• Older mothers instigated lower self-medication of antibiotics than younger mothers
**Iraq**	Al-Taie et al., 2021 (387)	• Assess KAP regarding the rational and self-medication use of antibiotics in the population<br>• Cross-sectional study using a questionnaire developed from the literature and piloted<br>• 384 participants	• RTIs were the infectious disease most treated with antibiotics (by prescription - 64.4% of the total), with UTIs constituting 10.1% of antibiotic prescriptions<br>• With respect to self-medication with antibiotics (45.8% of the participants), the most common infectious disease was influenza/ common cold (44.9% of conditions) followed by a sore throat (31.3%) and a cough (17.6%). High rates of self-medication with antibiotics (90%) were also seen in the study of Muhammed et al. (2021) (388)<br>• This was reflected in responses to knowledge and attitude questions where 57% agreed that antibiotics were useful for sore throats and fever, 54.7% against viruses such as influenza/ cold and 49.2% against a cough that has lasted more than a week, similar to the findings of Al-Yasseri et al (2019) (389)<br>• Of equal concern is that 49.2% of participants stated they discontinued antibiotics once they felt better and 40.9% kept antibiotics at home for future use<br>• In addition, only 34.4% agreed that the misuse of antibiotics increases ABR, which is an issue to society
	Alsayed et al., 2022 (275)	• Explore KAP regarding antibiotic use among key stakeholders in Arabic countries including adults in the community<br>• Cross-sectional descriptive study using a pre-validated survey instrument<br>• 6746 (23% of the total survey population) were adults<br>• These included adults from Iraq (29.4% of total adults)	• Encouragingly, 54.5% of adults thought ABR was a global problem reducing to 44.7% in their country, with 21.5% believing 21% - 50% of prescriptions for antibiotics are unnecessary<br>• Of concern is 58.8% of adults thought antibiotics are effective against sore throats, 57.6% against common colds, coughs and nasal congestion<br>• In addition, 48.3% believed that antibiotics are effective against fevers, 41.7% viral infections with a fever and 39.4% against viral infections<br>• Alongside this, approximately one-quarter of respondents stated that antibiotics will always be effective against the same infection in the future
**Jordan**	Hammour et al., 2018 (390)	• Evaluate the parents' KAP towards the use of antibiotics for children with URTIs<br>• Cross-sectional study using a validated questionnaire<br>• 1301 parents completed the questionnaire	• Encouragingly, 88.3% of participating parents agreed that antibiotics are being excessively used to treat URTIs<br>• In addition, 68.3% of parents strongly agreed/ agreed that if antibiotics are given for no reason, their effectiveness will decrease and bacteria will become resistant and 60% of parents strongly agreed/ agreed that since upper URTIs such as influenza, common colds and sore throats are typically viral in origin, antibiotics should not be given to cure them<br>• However, 72.4% strongly agreed/ agreed that once a child develops a fever, they should be given antibiotics regardless of the cause and 63.5% strongly

			agreed/ agreed that any complications of URTIs can be minimized by giving antibiotics
	Yusef et al., 2018 (391)	• Evaluate the areas of defects knowledge and practices about antibiotic use<br>• Cross-sectional study using a pre-tested and pre-validated questionnaire<br>• 1060 questionnaires were completed (98% response rate)	• 57% of surveyed patients stated they had received oral antibiotics in the past six months (or given them to their children) and 41% in the past two months, with antibiotics prescribed for a number of infections including URTIs (74%) and UTIs (over 10%)<br>• 53.8% of participants also believed every illness accompanied with a fever necessitates antibiotics and 39.4% believed they can stop taking antibiotics when their symptoms improve<br>• Alongside this, 70% of the respondents either answered incorrectly or not known about/ ever heard of the term AMR<br>• Whilst participants with higher education/ higher income were generally more knowledgeable about appropriate antibiotic use and AMR, some responses showed no statistical difference between the different groups
	Haddadin et al., 2019 (207)	• Assess patterns of dispensing antibiotics among community pharmacies<br>• Cross-sectional survey 12 pharmacists working in 7 pharmacies participated along with 434 customers/patients (98% response rate)	• Of the antibiotics dispensed with/ without a prescription only 31.5% and 24.6% respectively were given appropriate doses and duration<br>• Cephalosporins were the most commonly recommended/ dispensed antibiotics by pharmacists<br>• The most common reason for requesting antibiotics were RTIs (59.5%) – 19% for colds/ influenza
	Abdel-Qader et al., 2020 (392)	• Assess the awareness, attitude, and knowledge about antibiotics and ABR<br>• Cross-sectional study using a questionnaire based on previously validated questionnaire<br>• 620 householders completed the questionnaire (87.2% response rate) including those in affluent vs. deprived areas	• Encouragingly, participants in both the affluent and deprived regions believed the over use of antibiotics increases ABR (93.9% affluent vs. 90.6% deprived). In addition, 82.3% in the affluent region believed that ABR occurs when bacteria change in some way that reduces or eliminates the effectiveness of antibiotics (31.9% deprived region)<br>• In addition, in the affluent region, 92.6% of participating householder believed antibiotics are ineffective against common colds vs. only 5.8% in deprived regions<br>• However, 22% of surveyed householders who used antibiotics used them to treat a common cold, and 44.2% who used them stopped their antibiotics after no improvement in the initial days<br>• Alongside this, only 14.2% of surveyed households in the affluent region did not believe antibiotics work on most coughs and colds and 2.9% in deprived regions. Only 8.1% in the affluent region and 2.6% in the deprived region also did not believe antibiotics speed up a recovery from a cold, with similar levels of lack of knowledge regarding why it is essential to complete a course of antibiotics (i.e. to reduce ABR)
	Abdelmalek et al., 2021 (393)	• Assess knowledge and perceptions about antibiotic use and ABR in Jordan over an 8-year period<br>• Two cross sectional studies were performed 8 years apart using the same structured questionnaire based on available literature	• 83% of participants in 2010 knew the name of an antibiotic vs. 95.5% in 2018 with 58% in 2018 knowing that antibiotics are effective against bacteria vs. only 33% in 2010 ($p < 0.001$).<br>• In addition, only 27% of participants in 2018 believed antibiotics can be used to treat both bacteria and viruses vs. 35.4% in 2010; and in 2018 there was a significant ($p < 0.01$) increase in the % of patients who disagreed that antibiotics accelerate recovery from a common cold

		• 711 participants took part in 2010 and 436 in 2018	• Overall, the percentage of participants who believed that antibiotics can be used to treat common colds, toothache, a cough longer than a week or a sore throat also decreased. There was also a significant increase in those who believed that ABR leads to the use of more expensive antibiotics with more side-effects<br>• Of concern is that 69.5% of participants in 2010 disagreed that it is good to keep remaining antibiotic doses at home for later vs. only 57.4% in 2018 and the percentage who believed antibiotics can be used to treat UTIs, fever or bronchitis increased<br>• There was also a significant decrease in answers to the statement that ABR leads to an increase in the duration of illness
	Alsayed et al., 2022 (275)	• Explore KAP regarding antibiotic use among key stakeholders in Arabic countries including adults in the community<br>• Cross-sectional descriptive study using a pre-validated survey instrument<br>• 6746 (23% of the total survey population) were adults<br>• These included adults from Jordan (42.6% of total adults surveyed)	• Encouragingly, 54.5% of adults thought ABR was a global problem reducing to 44.7% in their country, with 21.5% believing 21% - 50% of prescriptions for antibiotics are unnecessary<br>• Of concern is 58.8% of adults believed antibiotics are effective against sore throats, 57.6% against common colds, coughs and nasal congestion<br>• In addition, 48.3% believed that antibiotics are effective against fevers, 41.7% viral infections with a fever and 39.4% against viral infections<br>• Alongside this, approximately one-quarter of respondents stated that antibiotics will always be effective against the same infection in the future
	Muflih et al. 2023 (394)	• Assess public understanding of antibiotics, self-medication, and drug disposal practices<br>• Cross-sectional survey using a self-administered questionnaire<br>• 1,105 participants completed the questionnaire	• Encouragingly only 16% of participants believed they should discontinue antibiotics once they felt better<br>• However, 44% practiced self-medication with antibiotics principally due to prior experience, costs and pharmacy<br>• In addition, only 6.4% of unneeded antibiotics were returned to a pharmacy with 60% kept at home<br>• Almost half of the participants who kept antibiotics said they would use them again with one-third saying they would give them to friends and family when needed
**Lebanon**	El Khoury et al., 2018 (395)	• Document parents' perspectives and assess their practices toward antibiotics used to treat URTIs in children<br>• Cross-sectional study using a structured questionnaire<br>• 1,037 parents participated in both public and private schools across Lebanon	• Appreciable misconceptions and malpractices among parents, e.g. 33.9% of parents considered antibiotics are helpful in treating common colds among children and 36.2% that antibiotics expedited recovery of their children from common colds<br>• There was also a lack of knowledge concerning antibiotic coverage with 37.9% of parents believed antibiotics could treat viral infections and 21.5% were neutral<br>• Approximately 20% of parents also believed they could reduce the dose of antibiotics once their child gets better
	Zahreddine et al., 2018 (210)	• Assess the knowledge of parents and community pharmacists regarding antibiotics and ABR in the paediatric population<br>• Cross-sectional study using a structured questionnaire<br>• 204 parents took part	• Encouragingly, 58.8% believed antibiotic misuse would lead to loss of immunity, 44.6% to recurrent infections and 38.7% to treatment failure<br>• Half of participating parents also believed antibiotic misuse is due to a bad choice/ indication with 40.2% and 39.7% respectively believing due to a bad dose or lack of adherence

			• However, 55.9% of participating parents believe antibiotics should be given to treat a fever, 49.5% a sore throat, 29.4% diarrhoea and 26% a cold<br>• 56.4% of parents blamed other parents for antibiotic misuse, with 52.5% and 37.3% respectively blaming physicians and pharmacists for misuse [this contrasts with 90.1% of pharmacists surveyed believing inappropriate parental behaviour was a major cause of antibiotic misuse along with inappropriate behaviour of physicians (72.8%)]<br>• Parents with university level education or a master's degree had significantly better knowledge compared to illiterate parents
	Mallah et al., 2020 (396)	• Assess the association between the level of knowledge and beliefs about antibiotics/ antibiotic misuse in Lebanon<br>• Cross-sectional study among parents of schoolchildren using a self-administered questionnaire based on published studies<br>• 1,460 parents took part (32.7% response rate)	• Antibiotic misuse behaviour included keeping leftover antibiotics for future use/ sharing them with others (22%) and doubling doses when doses were skipped (10.6%)<br>• Participating parents who believed that antibiotics were effective in treating viruses including colds/ sore throat infections were twice as likely to misuse antibiotics<br>• Parents who thought that antibiotics can treat colds were at substantially higher odds of misuse behaviour than others<br>• The odds of misusing antibiotics were also more than twice higher among parents who usually take antibiotics when they suffer from sore throats
	Henaine et al., 2021 (397)	• Evaluate the association between socio-demographic characteristics and knowledge about antibiotics in the Lebanese population<br>• Cross-sectional study with a questionnaire based on the WHO questionnaire<br>• 906 participate (90.6% response)	• The mean antibiotic knowledge index was 12.5 (minimum score: 3 and maximum score: 19) with greater knowledge inversely correlated with age<br>• Statistically significant differences were also found with type of residence, level of educational and total household income among participants<br>• Overall, a higher knowledge index was significantly associated with higher income levels combined with higher education levels

*World Bank Classification; ABR = Antibacterial Resistance; AMR: Antimicrobial Resistance; AWaRe = Access, Watch, Reserve antibiotics; FGD = Focus Group Discussions; HCP = Healthcare Professional; HCW = Healthcare Worker; PHC = Primary Healthcare; KAP: Knowledge, Attitude and Practice; URTI: Upper Respiratory Tract Infection

Table S15: Knowledge, Attitude and Practices, antibiotics, antimicrobial resistance and antimicrobial stewardship – WHO South East Asian Region Patients/ Public

Country	Author and Year	Objectives, Study Design and Population	Summary of the Findings
**Low Income countries***			
**Nepal**	Nepal et al., 2019 (398)	• Explore KAP towards antibiotic use among adults in Nepal<br>• ross sectional quantitative survey using an adapted and piloted questionnaire<br>• 220 households took part (97% response rate)	• Respondents had relatively good knowledge about the role of antibiotics, side-effects of antibiotics and ABR<br>• Encouragingly, 94.1% answered correctly that antibiotics are useful for killing germs, 84.1% that antibiotics are often not needed for treating colds/ influenza and 71.5% knew paracetamol was not an antibiotic<br>• In addition, 77.3% of participants stated they did not need to take antibiotics for a cold to prevent them getting worse and 78.2% and did not want to take an antibiotic if they did not need one. 81.3% also stated they completed the full course of treatment when prescribed<br>• However, 67.7% did not answer correctly that amoxicillin is an antibiotic and 32.7% did not know that antacids are not antibiotics<br>• Alongside this, 84.6% reported sometimes preferring an antibiotic when they have a cough or sore throat and 31.8% using antibiotics as a prophylaxis. 47.7% also thought antibiotics would help them recover more quickly if they had a fever
	Gyanwali et al, 2020 (108)	• Assess the medicine prescribing patterns and patients' knowledge about medicine use at different levels of health care<br>• Cross sectional study involving 88 health facilities in Nepal<br>• In addition, 2200 patients interviewed using a structured questionnaire	• Out of total drugs prescribed, 68.91% were essential drugs and 23.74 % were antibiotics, with prevalence rates ranging from 20.18% to 26.63% depending on the facility – greatest for health posts<br>• Among patients receiving medicines, adequate knowledge on medicine use was seen among 41% of patients surveyed, while individual correct responses regarding antibiotics were seen among 63% of patients (right medicine), 82% (right dose), 87% (right frequency), 75% (right time) and 59% (right duration)
	Vaidya et al., 2020 (379)	• Evaluate the accuracy of self-reported antibiotic use among patients coming to hospital with suspected enteric fever for 3 or more days in Bangladesh, Pakistan and Nepal vs. culture results from urine samples<br>• 2939 patients with suspected enteric fever were enrolled across 3 countries	• Antibiotics were detected in 39% of urine samples<br>• The correlation between measured and reported antibiotic use was modest<br>• In Bangladesh and Nepal, reported antibiotic use was higher than the detected antibiotics across all age groups<br>• After adjusting for disease severity, patients with antibiotics in their urine were slightly more likely to be blood culture positive for enteric fever; however, the effect was not statistically significant<br>• Interestingly, only 2.4% of participants sought care at a pharmacy in Pakistan before coming to hospital vs. 56.7% in Bangladesh and 59.6% in Nepal
	Adhikari et al., 2021 (218)	• Explore the characteristics of sales of antibiotics without a prescription, its drivers and implications among hospital clinicians, dispensers and patients<br>• Semi-structured interviews and FGDs involving 90 participants overall	• Dispensers were the main interface between the formal and informal healthcare sectors<br>• Dispensers were aware that antibiotics were sold with little interaction with patients, that empiric treatment was common, and often entailed inappropriate dosage and overtreatment with broad-spectrum antibiotics

			• Dispensers were also aware that these practices contravened public health recommendations; however, they were responding to patients' demands and barriers that patients face when accessing care from PHCs. A lack of time, costs, accessibility and perceived quality led patients to seek assistance outside of PGCs<br>• Only a few dispensers and patients were aware of AMR and described AMR as a potential consequence of inappropriate and overuse of antimicrobials through purchasing antibiotics without a prescription
	Rijal et al., 2021 (110)	• Explore KAP of antibiotic prescriptions and uses across sectors<br>• Cross-sectional study using a previously validated WHO questionnaire and pre-tested<br>• 324 patients were interviewed	• 42.9% of surveyed patients thought a fever could be treated with antibiotics, 35.2% that antibiotics can be used to treat colds and influenza; and 17.3% to treat a sore throat<br>• Of equal concern is that 84% of those surveyed had never heard anything about ABR and only 16% had heard about different terms for antibiotics<br>• In addition, 75% were unaware on anything related to ABR issues and 46.3% admitted they discontinued antibiotics after they felt better
	Marasini et al., 2024 (221)	• Explore knowledge, perceptions, and practices of antimicrobials and AMR among medicine dispensers and community members in Nepal<br>• Pre-tested semi-structured interview guides were used<br>• 16 In-depth interviews with medicine dispensers and 3 FGDs with community members	• Some participants described antimicrobials as comparatively strong and expensive medicines, and were seen as effective medicines resulting in fast recovery of diseases<br>• Overall, participants believed antimicrobials helped them to get better quickly<br>• However, of concern is that participants typically demonstrated a low level of knowledge concerning AMR
**Low-Middle Income countries***			
**Bangladesh**	Chowdhury et al., 2019 (399)	• Explore factors and practices regarding access and use of antibiotic use understanding on ABR<br>• Qualitative study with 6 FGDs and 16 in-depth interviews with a structured questionnaire	• Local pharmacies were chosen over public sectors as reliable source of antibiotics even though most medicines are free of charge in government-run facilities as they could obtain them quickly over-the-counter<br>• Antibiotics were perceived as useful for treating a variety of infections as well as non-infectious conditions including hypertension and lethargy<br>• Some male and female FGD participants volunteered views on ABR, i.e. heard about it on the radio along with potential ban on certain antibiotics
	Lucas et al., 2019 (400)	• Explore how households were accessing antimicrobials for themselves<br>• In-depth interviews using a semi-structured and piloted interview guide with 48 households in one urban and one rural area	• People accessed antibiotics through five pathways: drugs shops, private clinics, government/charitable hospitals, community/family planning clinics, and specialized/private hospitals<br>• Drug shops provided direct access to antibiotics for common, less serious and acute illnesses, with several pathways for persistent or serious illnesses. However, households often relied on drug stores for treatment<br>• Multiple and incomplete dosing of antibiotics were common even when prescribed by a qualified doctor in view of the high cost of antibiotics vs. other medicines<br>• The cost of antibiotics was often reported as a barrier to purchasing a full course, which is a concern

	Vaidya et al., 2020 (379)	• Evaluate the accuracy of self-reported antibiotic use among patients coming to hospital with suspected enteric fever for 3 or more days in Bangladesh, Pakistan and Nepal vs. culture results from urine samples<br>• 2939 patients with suspected enteric fever were enrolled across 3 countries	• Antibiotics were detected in 39% of urine samples<br>• The correlation between measured and reported antibiotic use was modest<br>• In Bangladesh and Nepal, reported antibiotic use was higher than the detected antibiotics across all age groups<br>• After adjusting for disease severity, patients with antibiotics in their urine were slightly more likely to be blood culture positive for enteric fever; however, the effect was not statistically significant<br>• Interestingly, only 2.4% of participants sought care at a pharmacy in Pakistan before coming to hospital vs. 56.7% in Bangladesh and 59.6% in Nepal
	Akhtar et al., 2021 (401)	• Assess antibiotic use for any reported illnesses in the preceding four weeks and knowledge regarding antibiotics during the COVID-19 pandemic<br>• Cross-sectional mobile-phone-based survey among people above 18 years of age using a structured questionnaire<br>• Total respondents were 1854 (85% response rate)	• 86.3% of participants had herd the names of various antibiotics with 12.1% having knowledge of the side-effects without consulting a doctor<br>• However, 53.1% did not know the duration of antibiotic treatment<br>• 46.7% participating patients trusted local pharmacies for treatment, 12.1% consulted with formal doctors and 16% went to private clinics
	Nizame et al., 2021 (224)	• Explore awareness of relevant policies and guidelines among drug shop operators and customers; identify current dispensing practices, barriers and facilitators to adherence to policies on antibiotic prescribing and dispensing<br>• Formative research to explore contextual drivers of antibiotic use and promotion among registered physicians, drug shop staff and household members	• Drug store operators typically did not dispense a full course of antibiotics for patients' infectious diseases in view of the financial circumstances of patients and/ or patients did not believe a full course was needed<br>• They were concerned that if they advise patients against taking antibiotics for minor illnesses including colds/ coughs and fever or insist on dispensing antibiotics only with a prescription – patients may take their business elsewhere<br>• Such activities are exacerbated by the scarcity of physicians in the locality
	Unicomb et al., 2021 (226)	• Develop of social and behavioural change communication to increase the appropriate use of antibiotics<br>• Use formative research methods/ findings and intervention design workshops with key stakeholders including patients to select target behaviours to improve future antibiotic use	• Participating patients typically seek healthcare advice from drug store sellers in view of travel costs and distances to health care facilities<br>• However, concerns that drug store operators often lacked knowledge about antibiotics and ABR – exemplified by limited activities among them to address current patient practices that antibiotics are typically stopped when symptoms disappear<br>• Of equal concern is that antibiotics are often purchased in drug stores by proxies for ill household members with underage children and adolescents readily able to purchase these – exacerbated by drug shop staff regularly selling antibiotics without a government license
	Islam et al., 2022 (227)	• Explore antibiotic dispensing patterns in pharmacies according to the WHO AWaRe classification<br>• Structured questionnaires among both drug sellers and patients purchasing antibiotics (with or without a prescription)	• Out of 2686 customers interviewed, 21.6% (580) had purchased antibiotics - 523 had purchased one, 52 had purchased two and 5 had purchased three courses of antibiotics (totalling 642 courses)<br>• Watch antibiotics accounted for 53.6% of antibiotic courses dispensed followed by Access (36.4%) and Reserve (10.0%) antibiotics

		• 128 drug sellers were interviewed alongside monitoring 2686 customers/ patients	• 50.9% were purchased without a prescription, with dispensing of non-prescribed antibiotics higher in the Access group (59.4%) followed by the Watch (46.5%) and Reserve (43.8%) groups
	Saha et al., 2022 (402)	• Determine the prevalence of self-medication and analyse key factors associated with it<br>• Cross-sectional study using a pre-tested and semi-structured questionnaire<br>• 1350 people participated	• Coughs, colds and fever were highly prevalent common diseases among the target population, with 80.9% self-medicating with antibiotics when they believed they were needed<br>• Overall, 34.5% of people surveyed self-medicated with antibiotics with 66.7% self-medicating for coughs, colds and fever (including with antibiotics)<br>• Self-medication was exacerbated by long distances to hospitals, with males more likely to self-medicate than females
	Azim et al, 2023 (403)	• Evaluate KAP of antibiotic use and AMR among Bangladeshi population<br>• Cross sectional study through a structured questionnaire developed following a thorough literature review<br>• 656 respondents were included.	• Overall, 52.29% of respondents demonstrated average knowledge (10.82% high knowledge), 67.84% moderate attitudes and 50.61% good practices regarding antibiotic use and AMR<br>• Encouragingly, 80.49% believed antibiotics were effective in treating bacterial diseases, 71.95% that ABR increases when doses are missed and 58.08% that ABR can develop from the misuse of antibiotics<br>• In addition, 67.84% of respondents showed moderate attitude with 77.9% accepted that antibiotics should not be consumed on suggestions from a medicine seller<br>• However, 90.7% believed antibiotics were effective in treating viral infections with 84.76% believing that antibiotics speed up recovery from most coughs/ colds and 83.23% from a fever
	Islam et al., 2024 (404)	• Investigate parents' KAP regarding antibiotic misuse and ABR among school-going children<br>• Community based cross sectional study among parents of school going students using valid and reliable questionnaires<br>• 704 parents participated	• An appreciable proportion of participating parents showed a lack of knowledge in recognizing basic antibiotics - 63% and 56% respectively did not know that amoxicillin and azithromycin were antibiotics<br>• Encouragingly, 79% knew that paracetamol was not an antibiotic, 75% knew that misuse of antibiotics could exacerbate ABR and 47% believed that antibiotic-resistant bacteria are difficult to treat<br>• In addition, more than 80% had positive attitudes toward non-antibiotic prescriptions, and were satisfied with the doctor's prescription<br>• However, 63% of participating parents believed that antibiotics could be used to treat colds and improve a fever and cold, 26% were ready to stop antibiotics among their children when symptoms improved and 27% reused the same antibiotics for similar symptoms
	Mannan et al., 2024 (405)	• Determine the prevalence of self-medication of antibiotics and contributing factors<br>• Cross sectional survey through a semi-structured questionnaire.<br>• 1537 participants took part in the study	• The top three reasons for self-medication with antibiotics were previous experiences (36.5%), urgency of the problem (22.6%) and economic constraints (16.9%) – economics include high consultation fees and long distance/ costs to travel to healthcare clinics<br>• Top three diseases for antibiotic use were a cough, cold, and fevers (70.1%), headache (13.8%) and dysentery, diarrhoea<br>• Family earnings, educational level, residential area and chronic morbidity significantly influenced knowledge about antibiotic use.
**India**	Nair et al., 2019 (119)	• Assessing the key drivers of antibiotic use among both formal prescribers (6), nurses	• Many physicians are happy to prescribe antibiotics without any clinical indication – often seeing them as a vital part of care delivery - as a result of

		(5), informal prescribers (5), pharmacy shopkeepers (5) and patients (7)<br>• Qualitative study involving in-depth interviews using an adapted validated questionnaire	variable follow up/ questioning, lack of testing facilities, risk of secondary infections and often unhygienic living conditions<br>• Prescribing/ dispensing of antibiotics is exacerbated by patient demand – with antibiotics seen as 'quick cures' – and lack of time with physicians typically seeing 80 – 100 patients/ day in PHCs<br>• Generally limited knowledge regarding AMR with some pharmacy shopkeepers believing AMR caused by bad weather with antibiotics often combined for a range of illnesses
	Tamhankar et al, 2019 (406)	• Describe characteristics of voluntary ABR awareness campaigns and future recommendations and approach for resource limited settings<br>• Questionnaire based study<br>• 19 respondents completed questionnaire (28% response rate)	• Over 85% of coordinators thought the voluntary campaign effective as it raised the awareness regarding rational antibiotic use and resistance<br>• Consequently, implications for settings with limited resources and for low-middle income countries
	Chatterjee et al, 2021 (407)	• Assess KAP in the community regarding antimicrobial use and resistance<br>• Questionnaire based cross sectional survey<br>• 502 respondents completed the survey and were analysed	• Overall, respondents had a fairly good knowledge about antibiotic use and AMR with 78.7% of responders agreeing with the statement suggesting that improper use of antibiotics contributes to ABR and 77.5% agreeing that non-adherence to a prescribed regimens enhances ABR<br>• However, approximately 47% agreed or strongly agreed that antibiotics were needed for a cough and cold and approximately 55% for watery diarrhoea<br>• In addition, 34.9% admitted to not completing the full course of antibiotics and 45.8% admitted to retaining left over antibiotics for future use<br>• Higher educational and socioeconomic levels were associated with better attitude scores; however, knowledge levels were comparable between groups
	Kotwani et al., 2021 (408)	• Explore knowledge, practice and, behaviour of consumers towards antibiotics and their use, AMR and their purchasing behaviour regarding antibiotics<br>• 72 in-depth interviews using a semi-structured interview guide	• Whilst 76% stated they had heard the term 'antibiotics'; overall, there was minimal understanding of the term<br>• Of concern was that incomplete consumption of full courses of antibiotics was common combined with not purchasing a full course in the first place - enhanced by financial constraints and lack of knowledge<br>• Less than one third of participating consumers had heard of the term AMR, neither were they able to define it. In addition, a misconception that the body develops resistance against antibiotics, with very few knowing that the misuse of antibiotics enhances AMR
	Pattnaik et al, 2023 (409)	• Evaluate KAP of rural communities regarding antimicrobial use and AMR<br>• Questionnaire based cross sectional survey<br>• 1003 participants took part	• 44.47% of respondents were aware of antibiotics, with 14.75% buying non-prescription antibiotics over the counter<br>• Of concern is that 28.03% of participants took antibiotics for a cold, 5.83% for a sore throat, 6.95% for watery diarrhoea, and 2.91% for malaria.<br>• In addition, 20.14% did not complete their full courses of antibiotics
	Turankar et al, 2023 (410)	• Assess difference of KAP among HCPs and laypersons regarding usage and resistance of antimicrobials<br>• Self-administered WHO questionnaire based cross sectional-based survey	• Encouragingly, only 3.1% of lay people thought the statement 'ABR occurs when your body becomes resistant to antibiotics and they no longer work' as false, with only 12.6% of laypeople stating as false that that it may be highly challenging or perhaps impossible to cure the diseases caused by bacteria if they are resistant to antibiotics

		• Study included 97 laypersons	• In addition, 89.7% agreed that it is the obligation of everyone to use antimicrobials responsibly to reduce AMR – especially with 83.5% stating that 'AMR worries me about how it may damage my family's health and myself'<br>• However, 52.1% answered incorrectly that in some countries AMR is a problem but not in ours<br>• Alongside this, 50.5% of laypeople thought that it was acceptable to use antibiotics that were given to a friend or family member, as long as they were used to treat the same illness and 46.4% that it is OK to buy the same antibiotic/ request the same antibiotic if you are sick with the same symptoms and this helped before
	Gautham et al, 2024 (411)	• Explored individual, community and health system-level factors influencing community antibiotic practices<br>• Mixed method including FGDs and in-depth interviews using semi-structured topic guides<br>• 98 community members took part in FGDs and 16 community leaders were interviewed - including teachers, elected village representatives and social workers	• At individual level, patients typically go to informal providers due to economic constraints, e.g. loose a day's wage going to clinics alongside co-pays for physicians. However, coughs and colds are treated with antibiotics (3 to 5 days), with people generally stopping antibiotics when they feel better<br>• Other major factors that influence antibiotic practices among surveyed participants were sociodemographic as well as cognitive and affective factors alongside economic constraints<br>• Lack of knowledge and empowerment were the major challenges faced by community leaders and healthcare workers while addressing people's doubts and a barrier was also created due to inaccessibility to doctors
**Indonesia**	Herawati et al., 2021 (412)	• Assess the effectiveness of an educational video about antibiotics and antibiotic use to improve outpatients' knowledge in these areas<br>• Pre-test—post-test quasi-experimental design with 98 outpatients in one hospital and 96 in another	• Overall, a significant improvement in knowledge scores ($p < 0.05$) in both hospitals<br>• Significant improvements in key areas including 'Antibiotics are remedies for diseases with symptoms of fever, runny nose, and sore throat' and 'If the disease has the same symptoms as a relative or a friend, the patient can use the antibiotics left over by the relative or friend<br>• In addition, improvement in 'Stopping the use of antibiotics before completing the course of treatment recommended by the doctor can cause the bacteria to become resistant'
	Karuniawati et al., 2021 (413)	• Investigate the levels and associated factors of KAP regarding antibiotic use among the public<br>• Cross-sectional study using a purposely designed and validated questionnaire<br>• 575 respondents (99.96% response rate)	• Encouragingly 67.7% of participants correctly stated that inappropriate use of antibiotics will cause ABR<br>• However, 73.1% stated that antibiotics could be used to treat infections due to viruses and 63.35% that antibiotics could reduce fever, with 50% considered stopping antibiotics when symptoms improve<br>• In addition, more than 50% of respondents incorrectly answered the question that antibiotics need to be stored in case of illness in the future and more than 45% considered taking antibiotics to speed up recovery from a cold with respondents hoping the doctor would give them antibiotics
	Naurita et al. 2021 (414)	• Identify information on antibiotics provided by pharmacy staff and recalled by patients<br>• A checklist used to obtain information provided by pharmacy staff with interviews conducted among patients to determine key information they recalled	• Almost all patients could recall information on administration directions - "route of administration" (97.0%), "frequency" (95.2%), "dosage" (92.6%), "hour of administration" (85.7%), "administration before/after a meal" (89.1%) and "duration of use" (90.9%)<br>• Fewer patients could recall "antibiotic identification" (76.5%) and "indication" (77.0%)

		• 230 patients took part (79.9% response rate)	
	Yunita et al., 2022 (415)	• Examine knowledge and practices of female visitors to health centres<br>• Cross-sectional study using a structured and piloted questionnaire<br>• 677 women took part	• Encouragingly, 82.7% of those interviewed believed that antibiotics are medicines used to kill bacteria; 80.8% that amoxicillin and ampicillin are examples of antibiotics, 68.6% that patients must take a complete course of antibiotics even if symptoms disappear and 55.6% that bacteria do become resistant to antibiotics<br>• In addition, only 12.5% believed antibiotics are effective against viruses, 22.3% that antibiotics work on most common colds and only 27.8% that antibiotics must be taken as soon as a fever develops<br>• Women with higher education, previous antibiotic experience, and easy access to primary care doctors were more likely to have good antibiotic knowledge
	Sinuraya et al., 2023 (416)	• Evaluate the level of knowledge and behaviour regarding antibiotic usage in the Indonesian population<br>• Observational study using a validated questionnaire<br>• 582 residents took part	• 63.5% of respondents were aware that antibiotics are used for treating bacterial infections, 60% understanding that antibiotics are not indicated for coughs or headaches<br>• In addition, 80.9% agreed that antibiotics should not be saved for later use with 79% understanding that antibiotics should not be shared with others,<br>• However, nearly 50% believed antibiotics can be used for viral infections<br>• Encouragingly, 75% of participants were committed to completing their prescribed course of antibiotics; however, 29% admitted to stopping antibiotics once they start feeling better<br>• Employment status, trust in physicians, and possessing sound knowledge of antibiotics were significant predictors of good behaviour in utilizing antibiotics
**Myanmar**	Miyano et al., 2022 (417)	• Assess KAP regarding antibiotics and AMR among adults<br>• Structured interview methodology using interactive voice responses<br>• 2045 adults participated (36% response rate)	• 89.5% of participants recognized antibiotics especially when names were mentioned and 60.6% believed unnecessary or inappropriate use of antibiotics could result in ineffective treatment/ ABR. In addition, 71.6% knew that antibiotics were not the same as anti-inflammatory medicines and 56.3% of participants recognized the concept of AMR<br>• However, 72.6% of participants believed that antibiotics could kill viruses and 73.5% that antibiotics are effective against colds and influenza - significantly greater among those in urban areas and 18–29 years of age<br>• In addition, 62.5% of participants stopped taking antibiotics when they felt better
**Sri Lanka**	Zawahir et al., 2021 (418)	• Evaluate Sri Lankan public's KAP about antibiotic use and self-medication<br>• Cross-sectional using a structured questionnaire<br>• 998 households took part (91% response rate)	• Overall, knowledge about antibiotics was poor (mean = 12.5; SD = 3.5; (scale 0-27))<br>• 11% had self-medicated the last time they took antibiotics<br>• Respondents were less likely to self-medicate if they did not support ease of access to antibiotics from pharmacies ($p < 0.001$) and had greater knowledge about prescription requirements for antibiotics ($p = 0.004$)

*World Bank Classification; ABR = Antibacterial Resistance; AMR: Antimicrobial Resistance; AWaRe = Access, Watch, Reserve antibiotics; FGD = Focus Group Discussions; HCP = Healthcare Professional; HCW = Healthcare Worker; PHC = Primary Healthcare; KAP: Knowledge, Attitude and Practice; URTI: Upper Respiratory Tract Infection

Table S16: Knowledge, Attitude and Practices, antibiotics, antimicrobial resistance and antimicrobial stewardship – WHO Western Pacific Region Patients/ Public

Country	Author and Year	Objectives, Study Design and Population	Summary of the Findings
**Low-Middle Income countries***			
**Cambodia**	Miyazaki et al., 2020 (419)	• Assessing the frequency of reported illness, common symptoms and associated health-seeking behavior of infants < 12 months of age and their caregivers<br>• Cross-sectional study using a structured and piloted questionnaire combined with a longitudinal survey	• 149 infants were enrolled, 51.4% reported symptoms of diarrhea, fever or cough in the previous 14 days, with use of antibiotics reported in 14.8%<br>• Among 47 infants enrolled in the longitudinal surveillance, there were 141 reported episodes of illness in 94% of infants with 45% of these reported to have received antibiotics<br>• Amoxicillin was the most commonly reported antibiotic in both surveys (68%) principally for fevers and coughs, with cefadroxil also used for similar indications (10.2%)
	Lim et al., 2021 (420)	• Measure public KAP regarding antibiotics and ABR<br>• Interview-based questionnaire, with the questionnaire adapted from various sources including the WHO<br>• 2005 participants completed the survey (response rates varied between 79% and 86% depending on the Province)	• Encouragingly, 69.7% of participants agreed that AMR could make medical procedures more dangerous, 74.8% that AMR could make infections more difficult to treat, and 70% that AMR will make many infections increasingly resistant to antibiotics<br>• However, 44% believed that antibiotics could help them recover faster from a common cold and only 6.3% of participants were able to correctly identify that viruses cause common colds and influenza<br>• Overall, 28.1% of participants believed antibiotics are effective against pain, 25.6% against sore throats, 24.5% against gastric ulcers, 21.7% against common colds and 12.7% for a fever, with only 1.2% correctly identified that antibiotics are effective specifically against bacteria<br>• Responses were reflected by conditions that participants used antibiotics for - including pain (38.7%), UTIs (20.3%), fever (17.2%), cough (12.4%), a runny nose (12.3%) and diarrhoea (5.3%)
**Lao PDR**	Haenssgen et al., 2018 (421)	• Assess the impact of educational activities on people's behaviours and perceptions<br>• The intervention involved sharing information and ideas regarding antibiotics and AMR in line with messages from the WHO over six sessions and a control group	• The educational activity influenced the awareness and understanding of "drug resistance" - The average recognition of the term rose from 27.6% to 91.4% in the active group vs. a rise from 36.2% to 58.8% among the unexposed group<br>• However, the evidence regarding the behavioural impacts was sparse and mixed – with the range of possible consequences included a disproportionate uptake of antibiotics from formal healthcare providers<br>• The findings casts doubt on the continued dominance of awareness raising as a behavioural tool to limit inappropriate antibiotic requests/ use among patients
	Haenssgen et al., 2019 (422)	• Assess antibiotic-related KAP among the general population in two LMICs including LAO<br>• Cross-sectional study using a questionnaire from a previous study<br>• 2141 adults took part	• Villagers more knowledgeable/ aware of antibiotics in Thailand (95.7% of participants) than Lao (86.4%; p<0.001) as well as ABR (Thailand 74.8% vs. 62.5% in Lao; p<0.001)<br>• In Lao, 38.6% used the official term for antibiotics (translated as germ resister) and also colloquial expressions for specific types of antibiotics including '*Ampi'* (75.6%) and '*Amok'* (35.3%)

			• However, common uses of antibiotics included external wounds (44.4%), coughs (30.5%), fevers (30.5%), sore throats (28.9%) and inflammation of the body (18.6%)
	Kounnavong et al., 2022 (423)	• Assess KAP of pregnant women regarding antibiotic use and ABR<br>• Structured interviews using a question-naire based on previously used instru-ments<br>• 539 women took part	• Only 23.9% of participating women stated that antibiotics are effective against bacterial infections, with 23.2% stating they are effective against viruses<br>• However, only 23.6% knew that a cold is caused by viruses<br>• In addition, only 16.5% of women had heard the term ABR, only 15.8% that bacteria can become resistant to antibiotics and only 22.5% that ABR is a big problem in Lao
	Sychareun et al., 2022 (424)	• Explore perceptions and reported practices of pregnant women and mothers with children under two years regarding correct antibiotic use and ABR<br>• Qualitative research design using FGDs<br>• 55 women took part in 6 FGDs	• Encouragingly, over half of the participants had heard the term ABR, with some mothers cited a link between ABR and superbugs<br>• In addition, most recognized ABR could harm health and making it harder to treat some infectious diseases<br>• However, participants felt that children should be given antibiotics to hasten recovery, with common conditions for antibiotics being fever, coughs, sore throats and pneumonia
**Vietnam**	Nhi et al., 2018 (240)	• Assess antimicrobial access and usage in for childhood diarrhea in an urban setting using a simulated patient study<br>• Interviews among caregivers (396) using a structured questionnaire	• 8% and 22% of outlets sold antimicrobials for pediatric watery and mucoid diarrhea, respectively.<br>• 59% of caregivers reported their children had received medication in the last month - 47% of which was an antimicrobial<br>• Concerns with knowledge among surveyed caregivers with 46% believing antimicrobials can be used to treat coughs, 35% fever, 30% colds, 21% headaches and 19% diarrhea, with 85% stating they had taken antimicrobials within the 30 days prior to the interview
	Ha et al., 2019 (425)	• Explore the awareness of antibiotic use and ABR among general population in highland provinces<br>• Cross-sectional study using a piloted structured questionnaire<br>• Information from 1000 households was used for data analysis (97.2% response rate).	• Encouragingly, 83.5% of household participants understood that using antibiotics required prescriptions and of these, 94.9% were aware that antibiotics had to be prescribed by physicians at the medical facility.<br>• 64.2% of people were aware of prescription drugs; 67.4% were aware of antibiotic use.<br>• Of concern is that only 18.8% of participants knew that ABR has a negative effect of antibiotic use, and only 55.8% were aware of ABR<br>• In addition, 53.9% of participants would re-examine their health if antibiotics were not working 21.8% would replace current antibiotics with other antibiotics.<br>• Higher age, education, and family income were positively associated with being aware of prescription medicines, antibiotics and ABR.
	Nguyen et al., 2019 (241)	• Understand key issues regarding access and use of antibiotic among drug sellers and patients<br>• In-depth interviews with drug suppliers FGDs with community members using structured guides<br>• 16 in-depth interviews with suppliers; 16 in-depth interviews with community	• Common illnesses that antibiotics used for (sore throats, cold, inflammation) – 31% members in interviews and 16% in FGDs<br>• Community members not completing the full course of antibiotics – 75% among members in interviews and 35% in FGDs<br>• Of equal concern is that participants in FGDs reported they took antibiotics 2 to 3 days and stopped once felt better to prevent side-effects

		members and 6 FGDs with 49 members in total	• In addition - selling antibiotics as customer preference – 25% in interviews and 14% in FGDs. However, suppliers stated that it was common for customers to request specific antibiotics based on previous experiences<br>• Antibiotics were often identified by shape and colour, with some confusing antibiotics with antipyretics such as paracetamol and anti-inflammatories when shown pictures
	McKinn et al. 2021 (426)	• Ascertain how people in Vietnam use antibiotics in community settings and factors that impact on this<br>• Conducted 43 qualitative in-depth interviews with 50 community members using a structured guide and subsequently conducting an iterative inductive thematic analysis alongside data collection	• There was frequent and indiscriminate use of antibiotics among intervened community members - driven by the powerful appeal that antibiotics held<br>• Consumers' decisions were affected by perceptions of what constitutes high-quality medicine especially surrounding antibiotics (effective, strong, accessible and affordable) with consumers more interested in symptom control vs. diagnosis with antibiotics seen as a trusted remedy across a range of infectious diseases building on their previous efficacy<br>• Generally incomplete understanding of the concept of ABR and its implications for individuals and for public health
	Cai et al., 2022 (427)	• Explore challenges and lessons learned from researching an approach to improve antibiotic utilisation across sectors<br>• Part of a pilot study involving multiple phases<br>• 26 participants took part in the pilot across 4 villages	• Gathering local information is key to improving understanding and potential future interventions to improve future use were identified as important among participants<br>• Local terminology was also identified as important for conveying concepts such as AMR<br>• Participants were familiar with the general concept of drug resistance rather than ABR, and felt worried about it. However, overall limited understanding of what either term really means among participants<br>• Of concern as well is that participants did not think AMR was an issue affecting their community
	Di et al., 2022 (428)	• Investigate key issues with adults regarding antibiotic use and ABR<br>• Cross-sectional Computer Assisted Telephone interviews using a structured validated and piloted questionnaire<br>• 1306 households provided a complete response (42.5% response rate)	• Whilst 67.2% of participants agreed that antibiotics were used to treat infections caused by bacteria, 67.9% also stated that antibiotics were used to treat infections caused by viruses – with respondents who bought antibiotics at pharmacies without a prescription accounting for a high proportion of these (39.0% strongly agreed, 20.7% agreed).<br>• Respondents with higher education levels (college and above) had 2.66 times higher knowledge scores than those with lower education levels ($p < 0.001$). High-income respondents also possessed more knowledge regarding antibiotics and ABR than low-income respondents ($p = 0.024$).<br>• In addition, respondents with higher/adequate knowledge scores had higher practice scores than those with inadequate knowledge scores ($p < 0.05$).
	Nguyen et al., 2022 (429)	• Undertake a survey of current antibiotic dispensing practices across Vietnam as well as ascertain customers' knowledge regarding antibiotics and factors affecting AMR<br>• Cross-sectional study using a questionnaire based on previous studies	• 61.4% of antibiotics were sold to treat RTIs, with the 'Access' antibiotics (amoxicillin and cephalexin) being the most frequently sold.<br>• Freelancer occupation and a lower educational level were factors related to purchasing antibiotics without a prescription.<br>• Participants purchased antibiotics without a prescription as they thought that their symptoms were mild (50.5%) as well as saving time (30.6%) and money (12.3%) by not visiting physicians

		• 1626 participants were observed and interviewed. Out of these, 487 purchased antibiotics and of these 81.7% (398 out of 487) purchased these without a prescription	• Participants with a prescription showed a significantly better awareness than those without a prescription about treatment duration (50.6% versus 37.6%, respectively; p = 0.026).<br>• Regarding AMR, over 50% of participants in both groups had heard of, or knew about, ABR. Despite this, only 48.7% knew that not taking antibiotics for long enough leads to AMR. However, 52.8% agreed that AMR was a severe public health problem
	Ulaya et al., 2022 (430)	• Assess levels of awareness and knowledge regarding antibiotics and ABR in a rural community<br>• Cross-sectional survey using a structured questionnaire<br>• 324 households participated (83.3% response rate)	• 71.8% of participants had heard of antibiotics, but only 21.1% could name any antibiotic and 17.7% had heard of ABR<br>• Antibiotic awareness was lower among those who lived further from health facilities but higher among those who used interpersonal sources for health information<br>• ABR awareness was lower among those who used private providers or pharmacies as their usual health facility but higher among those with high media use frequency and those who sought health information from official sources<br>• The most well-known antibiotic was ampicillin/ amoxicillin, with only 4.2% having never heard of it. Those who used private clinics, pharmacies, or drug stores were familiar with more antibiotics than those who used government facilities; however, less aware of ABR and scored lower on the ABR knowledge questions
**Upper-Middle Income countries***			
**China**	Cheng et al., 2018 (431)	• Examine antibiotic-related knowledge and behaviour among residents in rural China, identify factors associated with knowledge, and explore relationships between knowledge and antibiotic use<br>• Cross-sectional study using a structured questionnaire principally based on the literature<br>• 2611 were interviewed (94.6% response rate)	• Encouragingly, 71.5% of respondents reported that they would comply with the physician's decision not to prescribe an antibiotic. However, 28.5% reported they would feel unhappy and see another doctor or buy over-the-counter medicines if no antibiotic was prescribed. 14.3% of respondents also reported that they asked their doctor to prescribe a specific drug and 4.5% had requested multiple prescriptions.<br>• Of concern is that 91.6% of participants believed that antibiotics can control viruses and 77.5% that a combination of antibiotics is more effective than a single class. In addition, 44.7% of respondents said they would cease taking antibiotics with relief of symptoms.<br>• Self- medication with antibiotics was common with 46.3% of participants using over-the-counter (32.0%) or left-over medicines (14.8%) for common infections in the past three months.<br>• Those with a higher educational level and younger age group had greater knowledge of antibiotics. However, greater knowledge was associated with using left-over medication.
	Diao et al., 2018 (432)	• Investigate occurrence of RTIs and their effect on use of self/ professional care among patients in the community<br>• Cross-sectional household survey using a structured questionnaire	• 80% of participating households reported RTIs within last 9 months with a dry cough (58.9%), rhinorrhoea without pus (51.7%) and a sore throat (49.8%) the most common infections<br>• 81.3% of participants reported having used antibiotics to treat their RTIs, while purchasing antibiotics without a prescription was positively linked with a productive cough

		• 1968 completed the survey (91.1% re-sponse rate)	• Prescribing of antibiotics at clinics for RTIs increased from 77.7% for patients with one symptom to 96.4% for those with over seven symptoms
	Cheng et al., 2019 (433)	• Assess antibiotic using behaviors and associations of caregivers' variables and children's symptoms with URTIs<br>• Retrospective cross-sectional study	• Children with URTIs had a high consultation rate and a high prescription rate of antibiotics<br>• Some caregivers adopted without rationale 'self-medication' with antibiotics for their children<br>• Children with a fever were more likely to be taken to a doctor; among those, children with particular symptoms were more likely to receive a prescription for antibiotics<br>• Disseminating knowledge on antibiotics and URTI symptoms will be important targets for caregiver and physician education going forward
	Sun et al., 2019 (434)	• investigate leftover antibiotics and their influence on self-medication with antibiotics for Chinese children, and further explore different influences of leftover antibiotics<br>• Cross-sectional study using a structured questionnaire based on a literature review and piloted<br>• 9526 parents participated (88.7% response rate)	• 48.1% of parents reported keeping antibiotics at home for their children. Among those who had leftovers, 63.1% reported their leftover antibiotics came from previous prescriptions and 35.3% reported these came from pharmaceutical purchases<br>• Mothers, older age of child, higher household income, higher education level and medical background were significantly associated with keeping antibiotics at home – which was significantly associated with self-medication of antibiotics for their children
	Wang et al., 2019 (435)	• Assess KAP towards antibiotics among young parents and identify contributing factors of antibiotic usage<br>• Cross-sectional study using a structured questionnaire<br>• 1368 questionnaires were completed	• In the past 6 months, 68.9% of the respondents stated their doctor had prescribed antibiotics for their children, with 27.2% stating they had purchased antibiotics themselves in the past12 months<br>• Whilst 83.5% said antibiotics were not necessary for common colds, and 70.2% that overuse of antibiotics increased ABR, 81.2% believed antibiotics should always be prescribed for a fever, 70.8% antibiotics protect against colds, and 46.5% that antibiotics are the same as anti-inflammatory drugs<br>• Parents who deny the existence of antibiotic abuse in China, who have lower incomes, and who have antibiotics prescribed by doctors (requests), were more associated with incorrect practices than other parents
	Li et al., 2020 (436)	• Investigate KAP of antibiotics and ABR among customers visiting community pharmacies<br>• Multicentred survey-based study using a questionnaire developed from the literature<br>• 1800 customers participated (66.7% re-sponse rate)	• The knowledge of most of the participants was poor (22.3%) or average (69.4%) with only 9.7% having good knowledge about antibiotics<br>• 52.7% of participants believed antibiotics can be used to treat common colds and 45.6% were unable to differentiate between antibiotics and anti-inflammatory drugs<br>• However, 77.5% believed the unnecessary use of antibiotics is dangerous for health<br>• Encouragingly as well, 69.3% showed a good attitude towards antibiotic use with 72.3% strongly disagreeing/ disagreeing that double doses of antibiotics speed up cures; 63.7% strongly disagreeing/ disagreeing that taking multiple antibiotics is more effective than taking one and 49.8% strongly disagreeing/ disagreeing that costly antibiotics are more effective/ have fewer side-effects

			• In addition, 58.0% strongly agreed/ agreed that the effectiveness of antibiotics will be reduced if a full course is not completed and only 24.6% strongly agreeing/ agreeing that leftover antibiotics can be saved in case of future need
	Xu et al., 2020 (437)	• Assess antibiotic misuse among children and its variation in two provinces in China<br>• Cross-sectional study with data of 2924 parents in Zhejiang and 3355 parents in Shaanxi whose children were 0-13 years old were collected via a self-administrated questionnaire	• Compared to parents in Zhejiang, those in Shaanxi were more likely to keep antibiotics for children at home, to engage in self-medication with antibiotics and to make their children take antibiotics prophylactically<br>• While there were no significant differences between parents' requests for antibiotics during consultations, parents in Shaanxi province were more likely to receive prescribed antibiotics<br>• Overall, children in less developed provinces in China face higher risks of antibiotic misuse at home and when seeing physicians
	Duan et al. 2021 (438)	• Estimate the prevalence of the general population's irrational use of antibiotics and identify potential reasons<br>• A systematic review and meta-analysis were performed concerning four main behaviours relevant to access, irrational use and common misunderstandings involving four databases<br>• 78 studies were eventually included	Key findings included:<br>• The public can easily obtain antibiotics in China - with an estimated prevalence of 37% (95% CI: 29–46) of the population typically demanding antibiotics from physicians and 47% (95% CI: 38–57) purchasing non-prescription antibiotics from pharmacies – greatest in Western China<br>• People also commonly did not follow prescription advice (pooled estimate: 48%, 95% CI: 41–55) and used antibiotics for infections/ disease areas where not indicated (pooled estimate: 35%, 95% CI: 29–42)<br>• Misunderstanding of antibiotic use including possible indications and adverse outcomes was also common among patients
	Lin et al, 2021 (439)	• Investigate parents' decision-making processes with respect to treatment choices and antibiotic use for pediatric URTIs<br>• Data were collected from a random cluster sample of 3188 parents of children aged 0-13 years across three Chinese provinces<br>• Risk factors of parents' treatment choices and antibiotic use were assessed using binary and multinomial logistic regressions	• 46.0% of children whose parents had self-diagnosed their children with a URTI were given antibiotics, with or without prescription<br>• 40.5% were self-medicated with antibiotics by their parents and 56.1% obtained further antibiotic prescriptions at healthcare facilities.<br>• Approximately 70% of children with URTI symptoms sought care; of these, 54.8% obtained antibiotic prescriptions and 7.7% asked for antibiotic prescriptions, with 79.4% successfully obtaining them<br>• Those perceiving antibiotics as effective for treating common colds and fever, who had access to non-prescription antibiotics, and with greater perceived severity of infection, were more likely to use antibiotics.
	Luo et al., 2021 (440)	• Investigate non-prescription antibiotic use for coughs and explore to what extent antibiotic use knowledge was correctly instructed in communities<br>• Cross-sectional survey using a structured questionnaire among 3034 respondents	• 79.1% of respondents has experienced a cough in the past 12 months with a medium age of 36.5 years (IQR: 26–49)<br>• 12.21% had used non-prescription antibiotics, with the proportion of non-prescription antibiotic use for coughs peaking at approximately 16% among people aged 30–39 years<br>• The major sources of antibiotics were pharmacy (77.70%) and/or family storage (43.92%).
	Wu et al., 2021 (441)	• Assess the prevalence of self-medication with antibiotics among children 0-5 years and related factors	• Among 1188 participants, 14.32% had self-medicated their children with antibiotics in the past 6 months<br>• The higher the degree of the perceived threat of the infection and self-efficacy, the less likely parents were to self-medicate their children

		• Cross-sectional study using a structured questionnaire to collect data from 1188 parents of children aged 0-5 years	• The higher the degree of perceived barriers, the more likely parents were to self-medicate their children with antibiotics
	Yin et al., 2021 (442)	• Assess the prevalence of self-medication with antibiotics in China and related factors<br>• Cross sectional study using a structured questionnaire<br>• 3206 participants took part	• Of the 3206 participants, 10.32% reported self-medication with antibiotics in the past 6 months<br>• Only 12.04% of participants had a higher antibiotic knowledge level<br>• Participants who with middle or high perceived barriers to seeking health care services also had a higher likelihood of self-medication with antibiotics<br>• Measures to increase public health education and improve the accessibility of health services are crucial to decreasing self-medication in China
	Zhu et al., 2021 (443)	• Investigate the use of antibiotics for coughs among children < 5 years<br>• Community based survey using a structured questionnaire<br>• 3012 respondents took part	• 40.2% received antibiotics, out of which 18.7% were not prescribed (self-purchasing)<br>• Cephalosporins were the most frequently used antibiotic (52.8%) followed by followed by penicillins (30.3%) and macrolides (16.8%)<br>• Of concern is that > 60% of respondents failed to distinguish between antibiotics and anti-inflammatories, 16.5% that antibiotics should be used as soon as possible for coughs and 12.5% that patients have the right to decide on antibiotic use and they can request the doctor to prescribe antibiotics
	Zhou et al., 2021 (444)	• Ascertain nationality differences in KAP toward antibiotic use and understand the practices of self-medication with antibiotics among parents of various nationalities in China<br>• Cross-sectional study using a structured questionnaire based on the literature and piloted survey<br>• 296 individuals were eventually analysed (84.6% response rate)	• Chinese citizens surveyed were more likely to practice self-medication with antibiotic and have worse knowledge, attitudes and practices towards antibiotic use, compared to their Occidental counterparts<br>• Common symptoms for self-medication included a fever (60.98%), cough (58.54%), bronchitis (43.90%) and a sore throat (34.15%)<br>• The principal reasons for self-medication were previous medication experiences (80.49%) and same ailments with no need to see a doctor (39.02%)<br>• Purchasing antibiotics at pharmacies (92.08%) and using leftover antibiotics (26.83%) were usual approaches to obtaining antibiotics
	Yuan et al., 2022 (445)	• Estimate the prevalence of self-medication by parents among children under 12 years<br>• Cross-sectional study using a questionnaire based on previous studies<br>• 4,608 parents took part	• 24.21% of respondents reported self-medication in the previous year. Among these, 75.45% reported the use of cold and cough medicine, 54.21% reported self-use of respiratory medications and 46.51% gastrointestinal medicines<br>• 29.57% of parents reported self-medication with antibiotics<br>• Odds of self-medication were associated with being a father, living in Northern China and having a child aged 6–11 years
	Lin et al., 2023 (446)	• Identify public's behavioural patterns of antibiotic use for URTIs and their influencing factors<br>• Mixed method approach using a literature review and semi-structured interviews with a piloted questionnaire<br>• 815 respondents were enrolled	• 22.94% of participants saw URTIs as a serious illness with 53.87% stating they were able to distinguish severe from mild URTIs<br>• Encouragingly, self-medication without antibiotics was the most common approach by participants to treating URTIs (56.93%), followed by home remedies (51.04%) and formal health care seeking (44.29%). Self-medication with antibiotics for URTIs was seen among 39.63% of respondents<br>• However, 76.56% reported stopping antibiotics early. In addition, in post-use evaluation 55.95% that antibiotics are effective against URTIs and 51.17% that more effective than other treatments

			• Four behavioural patterns regarding antibiotic use for URTIs were identified: antibiotic self-medicators (20.25%), formal health care seekers (26.50%), various treatment users (24.20%) and self-medication without antibiotics (28.96%).<br>• Respondents perceived strong social influence to use antibiotics irrationally with 42.46% receiving recommendations to use antibiotics from their family and friends for URTIs and 48.59% from retail pharmacies
	Qu et al., 2023 (447)	• Estimate the prevalence and associated factors of self-medication with antibiotics among children in China, including parents' KAP towards antibiotic use<br>• Cross-sectional study using a structured questionnaire<br>• 1699 respondents participated	• 23.31% had practiced self-medication with antibiotics to their children in the past year<br>• A cough (59.6%) was the most common symptom leading to self-medication, with penicillins (85.4%) the most commonly used antibiotics<br>• Storing antibiotics at home resulted in an increased likelihood of self-medication in urban areas but not in rural areas<br>• There was also a higher probability of non-prescribed antibiotics to treat infectious diseases in children without chronic diseases
	Wang et al., 2023 (448)	• Explore the impact of knowledge and attitude on antibiotic use to treat a common cold based on the expanding KAP model<br>• Cross-sectional study among patients attending community health centres using a self-administered questionnaire<br>• 815 completed questionnaires (85.3% response rate)	• Encouragingly, 62.8% of participating patients believed over use of antibiotics leads to ABR and 41.8% that bacteria can develop resistance to antibiotics<br>• However, only 18.7% gave a correct answer to 'antibiotics can effectively treat common colds', 15.5% that 'antibiotics and anti-inflammatory medicines are the same' and 12.5% that antibiotics can treat viral colds<br>• On average, respondents who lived in high-income level areas had higher scores for antibiotic knowledge than others ($p < 0.05$), with knowledge identified as a significant predictor regarding attitudes and behaviour/ use of antibiotics<br>• Respondents with a medical background also had higher knowledge scores and better attitude scores than other patients
	Yan et al., 2023 (449)	• Investigate the moderating effect of parental skills on the link between parental skills for antibiotic use and inappropriate antibiotic use among children<br>• Cross-sectional study using a self-administered finalized following a pilot<br>• 9526 parents with children took part. Subsequently, data from 1944 parents who self-medicated their children and 2478 respondents whose children sought care were analysed	• Participating parents with higher education attainment and higher incomes were less likely to self-medicate their children with antibiotics<br>• Highly educated parents and those whose children were younger were also less likely to ask physicians for antibiotics when seeking care<br>• Parents with a medium level of skills for antibiotic identification as well as high level of skills for antibiotic use, and those with a high level of both skills, were less likely to ask physicians for antibiotics when seeking care
	Zhang et al., 2023 (450)	• Investigate the influences regarding decisions on treatment and antibiotic use for common illnesses among patients in Eastern China<br>• Semi-structured interviews in localities where concerns with antibiotic use and ABR	• A previous household survey including 1,379 participants 41.7% and 41.1% respectively reported using antibiotics for sore throats and fever<br>• In this study, most surveyed patients stated they would not self-medicate with antibiotics until their illness got worse or lasted longer than expected, with antibiotics considered as anti-inflammatory medicines for prolonged viral infections, with others seeing antibiotics as able to provide fast relief for their symptoms

		• Qualitative sample of 29 patients	• Patients will typically self-medicate for perceived mild infections such as coughs and sore throats with difficulties with accessing healthcare facilities
**Malaysia**	Choo et al., 2018 (451)	• Explore beliefs, knowledge, and practice regarding antibiotic patients attending hospitals and PHCs<br>• Cross-sectional study using an adapted questionnaire from previous studies<br>• Respondents were excluded from the study if they had no experience in consuming antibiotics<br>• 2632 patients were eventually included in the final analysis (94.9% usable response)	• Whilst 51% of respondents self-evaluated their knowledge regarding antibiotics usage as good, 67% had not heard about ABR<br>• In addition, 52.7% believed viral infections can be cured by antibiotics, 37.8% that antibiotic can be stopped once symptoms improved (with 48% admitting to this in practice) and 35.8% that taking low doses of antibiotics is better than not taking any<br>• 70.1% had high expectations that their physicians would prescribe antibiotics for their infections, with 66.0% in practice taking antibiotics to prevent illness from getting worse<br>• Lack of awareness regarding ABR was a significant factor associated with in-appropriate antibiotic use that needs to be addressed
	Halim et al., 2018 (452)	• Assess knowledge and attitudes concern-ing antibiotic use and ABR among the general public<br>• Cross-sectional survey using a validated self-administered questionnaire<br>• 326 were completed by members of the public (92.9% response rate).	• Concerns with general public's knowledge regarding antibiotics with 83.7% of the surveyed public believing that antibiotics could treat viral infections, 76% that antibiotics could alleviate a viral fever and 52.6% that antibiotics could treat all types of infections<br>• In addition, 63.7% admitted taking antibiotics in order to accelerate their re-covery from illness, 49.8% expect their physician to prescribe antibiotics if they feel ill and 34.8% stopped taking antibiotics when their symptoms disap-peared<br>• Typically, inadequate knowledge of about ABR with 72.9% of respondents not agreeing that resistant bacteria can spread from humans or animals to hu-mans and 32% unaware that bacteria can develop resistance to antibiotics.
	Irawati et al., 2019 (453)	• Explore residents' KAP regarding antibiotics and ABR<br>• 22 residents (aged ≥18 years) were interviewed using a semi-structured interview guide based on an extensive literature review<br>• The responses were thematically ana-lysed	• Most participating residents believed antibiotics can kill viruses and hasten recovery from viral infections, and were unaware that antibiotics have side-effects. A few participants also believed that antibiotics relieve pain and reduce inflammation<br>• Most residents also stopped taking antibiotics when their symptoms improved<br>• Most residents had heard of the term 'ABR'; however, only 9% understood it as a condition where bacteria become resistant to antibiotics. In addition, most were unaware of the consequences of ABR and how it can be prevented. In addition, only a few believed they were responsible for preventing ABR.<br>• Encouragingly most participants stated they would abide by their physician's decisions regarding their infection and would not request antibiotics specifi-cally as they trust their physician
	Aslam et al., 2021 (454)	• Estimate the prevalence rate self-medication with antibiotics and the rationale among the lay population<br>• Cross-sectional study using a validated questionnaire<br>• 480 lay persons were surveyed	• Prevalence of self-medication with antibiotics was 15.1% among those surveyed – principally to save money as well as lack of time for a check-up/ consultation<br>• Among the 480 participants, 20.6% stated they always use co-amoxiclav for a cough, cold, runny nose, influenza, sore throat, fever or diarrhoea followed by ampicillin/cloxacillin (14.2%) and levofloxacin (8.3%)

			• 31.7% of participants stated they stopped taking antibiotics when they felt better, with 57.7%, 51.5% and 41.5% of participants respectively believing that fevers, colds/ influenza and sore throats can be treated with antibiotics
	Chang et al., 2021 (455)	• Assess the knowledge of the Malaysian public regarding COVID-19 and antibiotics<br>• Web-based study using a validated and piloted questionnaire<br>• 2117 respondents took part	• Encouragingly, 59.6% of respondents believed that the misuse of antibiotics accelerates the resistance process with 58.6% believing ABR can cause mortality<br>• Of concern is that only 49% of respondents believed that antibiotics are effective against bacterial infections only
	Kong et al., 2021 (456)	• Explore knowledge, expectations, and practices regarding antibiotic use among the general public.<br>• Nation-wise cross-sectional survey using a self-administered questionnaire based on validated questionnaires from previous studies<br>• 1971 adults from each state of Malaysia	• The main reasons for using antibiotics among participating adults were fever (35.5%), RTIs (22.4%) and pain/ inflammation (14.5%)<br>• One inappropriate practice regarding antibiotics was observed in 56.6% responders, with 48.8% not keen to complete a course of antibiotics (most common reason)<br>• Of concern is that only 20.6% knew that antibiotics cannot be used to treat viral infections and 22.3% that antibiotics do not work on coughs and colds. 56.9% also believed it is OK to stop taking antibiotics when symptoms improve<br>• In addition, 62.9% of respondents expected an antibiotic to be prescribed for a fever, 57.2% for a sore throat, 50.9% for a cold or influenza, 49.5% for a runny nose with green mucus and 47.9% for a cough<br>• Of additional concern is that only 9.5% of respondents answered correctly that ABR occurs when your body becomes resistant to antibiotics and they no longer work as well<br>• Patients who had better knowledge were less likely to use antibiotics inappropriately.
	Thong et al., 2021 (457)	• Determine the impact of targeted educational interventions on public knowledge and perception of antibiotic use and ABR<br>• Experimental study design, with data collected via validated self-administered questionnaires<br>• 234 respondents (78% response rate) took part in the study	• The educational programme was effective improving knowledge and practice<br>• % response to 'ABR happens when the antibiotic loses its ability to treat bacterial infections' increased among respondents from 68.4% of responders to 94.4% (immediate after intervention) and 90.6% (2 weeks later)<br>• % response to 'Treatment may fail if you do not finish antibiotics as instructed' also increased from 79.9% to 97%<br>• Correct responses to 'Antibiotics can treat fever and sore throats; fever with running nose; common cough and wheezing and influenza' increased from 9.8% to 50.4% (immediate) and 43.2% (2 weeks after). In addition, correct responses to 'viral infections can be treated by taking antibiotics' increased from 25.6% to 88.0% (immediate) and 80.3% (after 2 weeks) and correct responses to 'ABR bacteria can be spread from one person to another' from 47.8% to 85.9% (immediate) and 81.2% (after 2 weeks)
	Wong et al., 2021 (458)	• Evaluate knowledge and sociodemographic factors that may influence inappropriate use of antibiotics<br>• Nationwide cross-sectional survey through telephone interviews using a computer assisted telephone interview system. The	• The majority of respondents had good knowledge of antibiotic usage and ABR problems<br>• However, only 23.8% of respondents were aware that antibiotics are not effective against viruses, only 23% knew that antibiotics cannot speed up recovery from a cough and 28.7% that colds and influenza cannot be treated with antibiotics

		questionnaire was based on previous studies and guidelines<br>• 1005 adult participated out of 11,036 randomised numbers (9% response rate)	• Of equal concern is that 25% of respondents admitted to not adhering to recommended doses, 19.4% reused antibiotics and 14.4% shared them with others.<br>• Awareness of terms 'drug resistance, 'ABR' and 'superbug' were also low at 12.2%, 6.0% and 5.9% respondents respectively<br>• Educational status, higher income and high antibiotic knowledge score were associated with higher practice scores

*World Bank Classification; ABR = Antibacterial Resistance; AMR: Antimicrobial Resistance; AWaRe = Access, Watch, Reserve antibiotics; FGD = Focus Group Discussions; HCP = Healthcare Professional; HCW = Healthcare Worker; PHC = Primary Healthcare; KAP: Knowledge, Attitude and Practice; URTI: Upper Respiratory Tract Infection

Table S17 – Consolidated Findings among Prescribers in the 4 WHO Regions

African Region	Eastern Mediterranean Region	South East Asian Region	Western Pacific Region
a) Prescribing Behaviour<br>• Typically excessive prescribing of antibiotics for essentially self-limiting conditions including URTIs, fever of unknown origin/ undifferentiated fever and acute diarrhoea. Excessive prescribing generally higher for children than adults, those in rural vs. urban settings and among physicians in private vs. public clinics. Lower rates of antibiotic prescribing were typically seen in mothers with higher education<br>• Lack of time with patients and concerns with knowledge of antibiotics and AMR amongst prescribers, alongside patient demand/ expectations, exacerbates excessive prescribing of antibiotics with typically high rates of empiric vs. targeted prescribing. Overall, where indicated, high patient expectations that antibiotics will be prescribed even for URTIs<br>• In some countries higher rates of inappropriate antibiotic prescribing in nurses/ clinical officers vs. physicians; however, the reverse in other African countries<br>• Variable adherence to local STGs among studied African countries, with often poor adherence to current guidelines<br>• Penicillins typically the most prescribed antibiotics among studied countries. However, do see prescribing of cephalosporins (e.g. ceftriaxone) and quinolones (ciprofloxacin). High rates of the prescribing of penicillins reflected in high prescribing rates of 'Access' antibiotics versus other the other categories when antibiotics are prescribed in a number of African countries, e.g. 85.0% - 94% of antibiotics prescribed [e.g. Burkina Faso, Kenya and Uganda - (8, 21, 54)]. However – high rates of 'Watch' antibiotics prescribed in some, e.g. 30.3% - 55.3% in DR Congo, Ethiopia and Tanzania (11, 15, 62)	*a) Prescribing behaviour*<br>• Variable practices with prescribers in a number of the low- and middle-income countries in the Region believing that antibiotics are not effective against viral infections and that their overuse will increase AMR. In addition, the body is well able to fight mild infections without the need for antibiotics<br>• However, in others prescribers believe antibiotics are effective in reducing the severity/ can be prescribed for URTIs/ treating fevers and that broad spectrum antibiotics are preferred to narrow ones for the treatment of URTIs<br>• In addition, do see excessive antibiotic prescribing in patients where an indication/ diagnosis is lacking – with the belief that antibiotics do not cause harm<br>• High patient demand in some countries for Physicians/ HCPs to prescribe antibiotics even for colds/ URTIs. However, in others Prescribers have initiated effective communication campaigns to educate patients when antibiotics are necessary, e.g. Jordan, as well as advise patients to complete the full course of prescribed antibiotics.<br>*b) Knowledge*<br>• Generally greater awareness of key issues surrounding AMR among physicians vs. nurses as well as physicians working in outpatients vs. GPs, with GPs more	*a) Prescribing behaviour*<br>• Typically high rates of prescribing of antibiotics for URTIs and diarrhoea, with high rates of prescribing of 3rd generation cephalosporins in some followed by penicillins.<br>• In urban areas in India do see 'Watch' antibiotics accounting for up to 54.9% of antibiotics prescribed – although generally antibiotics more likely to be prescribed in rural vs. urban areas<br>• High rates of antibiotic prescribing exacerbated by patient demand – driving up AMR<br>*b) Knowledge*<br>• Generally good knowledge regarding AMR and that excessive use of antibiotics will increase AMR – although variable between countries<br>• Typically qualified prescribes/ clinicians had greater knowledge of antibiotics/ AMR than unqualified prescribers<br>• Whilst in some studies local prescribers had reasonable knowledge of antibiotics/ AMR – small number believed antibiotics were effective against viral infections<br>*c) Training*<br>• In some countries, over 50% of prescribers currently unaware of the AWaRe classification for antibiotics<br>• Training needed to address knowledge gaps especially as in some countries prescribers currently find it hard to select	*a) Prescribing behaviour*<br>• Typically high rates of prescribing of antibiotics for URTIs and gastroenteritis despite in some cases prescribes knowing that URTIs/ common cold caused by viruses. However, not always the case, e.g. Malaysia with limited prescribing of antibiotics for URTIs<br>• Higher rates of antibiotic prescribing among neonates/ children vs adults, in rural vs. urban areas, in prescribers with less clinical experience and among prescribers in private practice vs. public clinics. In addition, have seen lower rates of prescribing of 'Access' antibiotics in private clinics (64.2% - 68.3%) versus public clinics (over 90%), with greater prescribing of 'Watch' antibiotics in young children/ follow-up visits<br>• Variable adherence to guidelines. However, where adherence occurs do see increased rates of appropriate prescribing<br>• Lack of CST/ resistance data and activities of Pharma companies influences higher prescribing rates of antibiotics alongside lack of regular training and updates<br>*b) Knowledge*<br>• Generally good knowledge regarding antibiotics and AMR and that excessive use of antibiotics will increase AMR – although variable between countries as see in some high rates of prescribing of antibiotics for URTIs and belief that cortisone is an antibiotic (Laos)

• Encouragingly, general awareness that AMR is a problem as well as increased awareness and usage of the AWaRe classification when describing and reviewing prescribing habits.<br>*b) Knowledge*<br>• Variable knowledge and awareness regarding antibiotics, AMR and AMS with generally concerns across most of the African countries<br>• There are also issues of terminology surrounding antibiotics and AMR in some countries with these terms not readily translatable in local languages<br>• Differences seen in knowledge scores regarding antibiotics and the reality in terms of e.g. believing that antibiotics are effective against viral infections. In addition, concerns with the length of the course of antibiotics prescribed – although variable<br>*c) Training*<br>• General lack of training regarding antibiotics/ classes, AMR and ASPs – particular the latter<br>• Lack of training regarding STGs with generally high rates of non-adherence – but not universal<br>d) No obvious differences in prescribing behaviour/ knowledge/ training by income levels between documented countries	likely to prescribe antibiotics generally<br>• However do see limited use of sensitivity testing unless no improvement in patients<br>*c) Training*<br>• Seen as important to help identify resistance patterns and react to these when prescribing<br>• Currently limited training on antibiotics/ AMR in a number of the studied countries<br>• In addition, can play a role in ASPs with additional training as limited ASP activity currently – although to see these in some, e.g. Egypt<br>d) No obvious differences in prescribing behaviour/ knowledge/ training by income levels between documented countries	the correct antibiotic for the condition<br>d) No obvious differences in prescribing behaviour/ knowledge/ training by income levels between documented countries	• Encouragingly, where see high levels of knowledge of antibiotics/ AMR do see low interest to prescribe antibiotics/ high intensity to reduce antibiotic prescribing (China)<br>*c) Training*<br>• Concerns with the lack of training regarding antibiotics/ AMR enhancing inappropriate prescribing/ AMR<br>• Appropriate training can address concerns with influence of Pharma Companies<br>d) No obvious differences in prescribing behaviour/ knowledge/ training by income levels between documented countries although less prescribing of antibiotics for viral infections in public clinics in Malaysia

Table S18 – Consolidated Findings among Dispensers in the 4 WHO Regions

African Region	Eastern Mediterranean Region	South East Asian Region	Western Pacific Region
a) Dispensing practices<br>• Generally high rates of dispensing of antibiotics without a prescription seen across African countries – lower among some countries/ locations e.g. South Africa. Typically for self-limiting conditions including URTIs, gastroenteritis, stomach aches/ pain and UTIs<br>• Typically, penicillins most dispensed antibiotics although see high rates of azithromycin and cephalosporins in some (Ghana and Nigeria). As a result, see high rates of 'Access' antibiotics dispensed, e.g. 84.6% in Burkina Faso (15.4% 'Watch') – although 25.6% 'Watch' antibiotics in medicine outlets in DRC<br>• Higher rates of antibiotic dispensing in children vs. adults and those with less experience/ assistants (Ghana) – although not always the case (Tanzania)<br>• Generally considerable influence of patients – with requests for antibiotics enhanced by beliefs with their curative powers for self-limiting conditions such as URTIs as well as fevers, etc.<br>*b) Knowledge*<br>• Generally good knowledge with beliefs that antibiotics should be used more prudently although not always reflected in practice, e.g. antibiotics are effective against viral infections such as URTIs<br>• However, knowledge gaps remain (although variable), with generally greater knowledge regarding antibiotics than AMR<br>*c) Training*	*a) Dispensing practices*<br>• Typically, high rates of dispensing of antibiotics without a prescription across the Region. This includes for common colds and typically driven by patient demand as well as lack of time/ resources to see physicians<br>• This includes high rates of dispensing of cephalosporins in some countries (Jordan)<br>• Variable education of patients including whether needing antibiotics, length of course and duration as well as how to prevent UTIs (e.g. Jordan and Sudan)<br>• In addition, variation about the extent of asking patients about current symptoms<br>• Seen that improved education of pharmacists (following courses) increases the number of pharmacists that would not dispense antibiotics for common colds (Egypt)<br>*b) Knowledge*<br>• Generally reasonable knowledge of antibiotics and AMR (especially basic knowledge); however, in practice can be concerns, e.g. in Egypt 97.6% subsequently dispensed antibiotics for acute bronchitis and 99.1% for common colds. In addition, concerns that leftover antibiotics can be used to treat similar infections in the future<br>• However, generally low awareness of AWaRe classification/ guidelines when mentioned<br>• In addition, poorer knowledge generally among assistants vs. pharmacists. Greater knowledge of antibiotics/ AMR with more experience<br>• Belief that MOHs could issue more up-to-date guidelines to improve future care, with AMS/ ASPs encouraged to improve future care<br>*c) Training*<br>• Concerns in some countries that reasonable percentage of dispensers had not taken part in awareness campaigns/ courses to reduce unnecessary antibiotic dispensing/ AMR	*a) Dispensing practices*<br>• Typically, high rates of dispensing of antibiotics generally across countries (although variable) driven by key issues including high patient co-payments and ease of access to community pharmacists/ drug sellers<br>• Antibiotics typically dispensed for self-limiting conditions including URTIs, acute diarrhoea, dysentery and fever (unknown origin)<br>• Higher rates of dispensing of antibiotics without a prescription from unqualified personnel/ those with limited education vs. more knowledgeable pharmacists (although concerns here as well)<br>• Where documented – patients are often a key driver (through requests) for the dispensing of antibiotics without a prescription<br>• Concerns that in a number of situations often incomplete courses of antibiotics dispensed – driven by affordability issues<br>• No real recording of antibiotics dispensed according to their AWaRe classification<br>*b) Knowledge*<br>• Encouragingly, reasonable knowledge that AMR is a global concern – however, often limited knowledge of its causes<br>• Variable knowledge of antibiotics, AMR and AMS – and concerns that often believed that antibiotics will be effective against viral infections. Typically, more limited knowledge of antibiotics and	*a) Dispensing practices*<br>• Typically high rates of dispensing of antibiotics without a prescription driven often by patient demands. However, concerns that incomplete courses may be dispensed and patients not completing their full course<br>• In Vietnam – seen high rates of dispensing of 'Watch' antibiotics (39.3%) vs. 'Access' antibiotics (59.0%) – with higher rates of 'Watch' antibiotics dispensed in children<br>• However in China – have seen 34.1% of pharmacists/ dispensers asking patients about their knowledge of antibiotics when discussing treatment of their infectious disease, with 30.5% always/ often collaborating with other HCPs to improve patient care<br>*b) Knowledge*<br>• Variable knowledge about antibiotics and AMR – worse among drug sellers with considerable beliefs that antibiotics are effective against viral infections whilst concurrently unsure about AMR. Trained pharmacists more knowledgeable<br>• Whilst knowledge of a number was reasonably good regarding antibiotics/ AMR – also believed/ dispensed antibiotics for URTIs and believed cortisone was an antibiotic<br>*c) Training*<br>• Generally recognised need for training especially surrounding

• Identified need to address knowledge gaps regarding antibiotics/ AMR and associated skills to reduce inappropriate dispensing of antibiotics<br>• Believed that training would help with diagnosis/ appropriate management of infectious diseases seen in ambulatory care (including surrounding the AWaRe book/ classification) and reduce the extent of antibiotics dispensed as recognised that not educating patients sufficiently regarding antibiotics/ AMR<br>• Peer supervising seen to enhance appropriateness (Uganda)<br>d) No obvious differences by country income levels	• Acknowledged need to train on information regarding resistance patterns and what this means for antibiotics recommended/ dispensed<br>• Acknowledged that improved training for pharmacists regarding communication skills with patients would help to reduce inappropriate dispensing of antibiotics. In addition, training on current guidelines to encourage more appropriate communication/ behaviour from patients<br>• Acknowledged need for training for AMS/ ASPs as only a minority fully aware of these issues including terms such as 'superbugs'. However, acknowledged barriers to AMS activities including lack of time/ personnel, training and resources<br>• In the meantime, real time feedback on dispensing behaviour would be useful to improve future activities<br>d) No obvious differences by country income levels	AMR among non-qualified dispensers<br>*c) Training*<br>• Where mentioned, additional training welcomed to address concerns with current dispenser knowledge of antibiotics, AMR and AMS<br>d) No obvious differences by country income levels	ASPs – although the general need for training surrounding antibiotics, AMR and ASPs identified<br>• Training also required on counselling patients regarding antibiotics and potential adverse events to improve future use<br>d) No obvious differences by country income levels

Table S19 – Consolidated Findings among Students in the 4 WHO Regions

African Region	Eastern Mediterranean Region	South East Asian Region	Western Pacific Region
a) Knowledge/ Attitude<br>• Variable knowledge regarding antibiotics, AMR and its causes, with generally greater knowledge among final-year students as well as Medical/ Pharmacy students vs. other healthcare students<br>• Most believed that inappropriate and high use of antibiotics contribute to AMR<br>• However, concerns that despite often reasonable/ good knowledge of antibiotics and AMR, students (e.g. in Ethiopia and other countries) found it acceptable that patients stop taking antibiotics when they felt better, used left over antibiotics, and believed/ had used antibiotics (including self-purchasing) for managing URTIs (especially among children) and diarrhoea (can be lower with Medical Students e.g. Tanzania)<br>• Overall – see relatively high self-purchasing of medicines to treat self-limiting conditions such as URTIs/ diarrhoea in view of costs and time pressures. In addition, believe sufficiently knowledgeable and avoid physician costs (especially among Medical students and also females vs. males)<br>• Concerns also among some students that broad spectrum antibiotics are better than narrow ones, and newer ones better than older ones<br>• Overall greater knowledge regarding antibiotics than AMR. Often limited knowledge regarding AMS/ ASPs<br>*b) Training needs*<br>• Recognised that more training needed regarding antibiotics in clinical settings including the spectrum of different antibiotics<br>• Appreciable training needed regarding AMS – what is this/ meaning – as well as ASPs to help improve future antibiotic use once qualified	*a) Knowledge/ Attitude*<br>• Variable knowledge of antibiotics and AMR with generally reasonable/ good knowledge of the causes of AMR, i.e. excessive use of antibiotics, non-compliance with doses prescribed/ dispensed and storing antibiotics for future use. However, concerns that despite ongoing knowledge do recommend/ use antibiotics for viral infections including URTIs with some stopping their antibiotics when feeling better. In addition, beliefs among some students that antibiotics are effective against URTIs/ fever and viral infections generally<br>• Concerns also with the terminology – with some only believing AMR is a problem if people take antibiotics regularly<br>• Typically, greater knowledge of antibiotics/ AMR among Pharmacy/ Medical students than other healthcare students and among healthcare vs. non-healthcare students. Some studies also showed that female students had better knowledge of antibiotics/ AMR than male students<br>*b) Training needs*<br>• Recognised training needs regarding antibiotics, AMR, AMS and ASPs to help improve future antibiotic use as confidence levels regarding prescribing/ dispensing of antibiotics low among some students. This is important as studies have shown that awareness regarding AMR and AMS can be improved by pertinent curricula activities (Pakistan)<br>• Recognised need for tools to be available to improve future prescribing/ dispensing of antibiotics as well as providing rapid information on local resistance patterns and the implications for prescribing/ dispensing	*a) Knowledge/ Attitude*<br>• Variable scores regarding antibiotics and AMR – with better scores among medical vs. non-medical students, healthcare vs. non healthcare students, pharmacy vs. non-pharmacy students and among nurses<br>• However – even with reasonable/ good scores – do see beliefs that antibiotics are effective against viruses/ URTIs, fever and diarrhoea and hasten recovery. In addition, stop taking antibiotics when feeling better and using left over antibiotics including from family/ friends<br>• Often high degree of self-medication including antibiotics for viral infections, etc., for convenience, long waiting times to see a physician and costs. In addition, believe no need to see a physician in view of their knowledge. In some studies – see greater self-medication among male vs. female students<br>• Finaly year students typically greater knowledge than others on antibiotics/ AMR but not always<br>*b) Training needs*<br>• Recognised need to know more about the rational use of antibiotics and improved attitudes generally towards antibiotics<br>• This includes knowledge surrounding guidelines/ appropriate antibiotic selection to reduce AMR<br>• Greater need also for training regarding AMR, AMS and ASPs –	*a) Knowledge/ Attitude*<br>• Variable knowledge across countries regarding antibiotics and AMR – with good knowledge in a number of countries that over use of antibiotics increases AMR and that antibiotics are not effective against antibiotics<br>• Having said this – do see self-purchasing of antibiotics for viral infections and beliefs that antibiotics are effective against viral infections<br>• Generally better knowledge of antibiotics/ AMR among Medical vs. non-medical students, and final year medical students vs. lower years. In addition better vs. nurses<br>• Do see that among Medical students with higher KAP scores – limited use of antibiotics to treat colds. However, those students with parents with a medical background seen to have poorer antibiotic behaviour than other students<br>• Typically poor to average knowledge regarding AMS/ ASPs<br>*b) Training needs*<br>• Recognised need for training regarding antibiotics, AMR and ASPs, wit students with better KAPs towards antibiotics/ AMR more aware of high risk practices leading to AMR<br>• Training also on robust guidelines as recognised that improve adherence to guidelines

c) No obvious differences in responses among students by country income levels although perhaps greater knowledge of antibiotics/ AMR among students from South Africa	• Training also on government/ health authority activities to improve future prescribing/ dispensing as well as improved use of social media to provide pertinent information to improve prescribing/ dispensing and reduce AMR<br>c) No obvious differences with student replies/ knowledge by country income levels	especially AMS and ASPs – with poor to average knowledge of these and AMR<br>c) No obvious differences with student replies/ knowledge by country income levels	improves appropriate antibiotic use<br>c) No obvious differences by country income levels

Table S20 – Consolidated Findings among Patients/ Public in the 4 WHO Regions

African Region	Eastern Mediterranean Region	South East Asian Region	Western Pacific Region
a) Knowledge/ Attitude<br>• Limited knowledge generally regarding antibiotics and AMR with patients typically believing antibiotics are effective against viral infections such as URTIs, fever and acute diarrhoea<br>• Even when demonstrating good knowledge regarding antibiotics/ AMR or concerns with AMR – typically believe/ consume antibiotics to treat e.g. URTIs, acute diarrhoea and fevers, e.g. DRC, Ethiopia, Ghana, Malawi, Tanzania and South Africa. In addition, belief generally that AMR cannot be transmitted between humans<br>• Generally better knowledge of antibiotics and AMR in more educated patients and those in urban vs. rural settings. This is an issue as patients with poorer knowledge regarding antibiotics/ AMR have 5.5 times higher odds of irrationally using antibiotics than those with greater knowledge (Tanzania) and more educated patients more likely to recommend restrictive antibiotic practices<br>• In addition, generally greater knowledge about antibiotics than AMR – although concerns with both<br>• Concerns in countries such as Ghana that education regarding antibiotics typically provided by drug pedlars<br>*b) Practice*<br>• Typically high rates of purchasing antibiotics without a prescription exacerbated by patient pressure - driven by issues of convenience with pharmacies/ drug sellers, costs and only minor disease (so no need to see a physician/ clinic) with antibiotics seen as 'powerful medicines' offering quick relief for e.g. URTIs<br>• Typically also 'Access' antibiotics most used, e.g. penicillins – although higher use of 'Watch' antibiotics reported in Zimbabwe (up to 25% of total). In addition, have seen higher use of azithromycin and ciprofloxacin in some countries and in Uganda see comparatively higher	*a) Knowledge/ Attitude*<br>• Variable knowledge of antibiotics and AMR among studied patients. Knowledge generally better in urban vs. rural settings and among more educated families. Variable findings whether knowledge better among males vs. females<br>• Typically, greater knowledge generally regarding antibiotics than AMR. However – even when professing good knowledge of AMR typically beliefs that antibiotics are effective against self-limiting conditions such as URTIs, diarrhoea and fever alongside pain/ inflammation. In addition – also generally poor practices towards antibiotics<br>• Lack of knowledge generally about the reasons behind necessarily completing the course of prescribed/ dispensed antibiotics (reflected in their documented practices)<br>*b) Practice*<br>• Generally high rates of purchasing antibiotics without a prescription<br>• Typically (when mentioned) pressure on physicians/ pharmacists to prescribe/ dispense antibiotics even for self-limiting conditions such as URTIs and diarrhoea in the belief of their effectiveness – some also mentioned that antibiotics gives them strength<br>• Concerns also that an appreciable number of patients admitted to stopping antibiotics when they felt better and storing left over antibiotics for their next infectious disease (typically themselves/ family member) especially if affordability was an issue<br>c) No obvious differences by country income levels	*a) Knowledge/ Attitude*<br>• Variable knowledge and attitudes regarding antibiotics and AMR (better for antibiotics) – with often seen as having reasonable knowledge in a number of countries but poor attitudes towards antibiotics in terms of believing them effective/ accelerating recovery in self-limiting conditions including URTIs as well as dysentery/ diarrhoea, e.g. Bangladesh, Indonesia and Nepal<br>• Better attitudes towards antibiotics with higher education and less likely to self-medicate with antibiotics<br>*b) Practice*<br>• Generally high rates of purchasing of antibiotics without a prescription even for self-limiting conditions such as URTIs, fever and acute diarrhoea in view of costs to see a physician, convenience of pharmacists/ drug sellers and previous experiences – overall typically see pharmacists/ drug sellers as trusted outlets for treatments<br>• Typically patient demand for antibiotics based on previous experiences, etc., with drug sellers believing patients will take their business elsewhere if suggested antibiotics not dispensed<br>• Concerns that patients stop taking antibiotics when feeling better, use leftover antibiotics for similar infections in the future and will only purchase part courses of antibiotics if insufficient funds	*a) Knowledge/ Attitude*<br>• Variable knowledge regarding antibiotics and AMR with participants in a number of studied countries believing antibiotics are effective against URTIs, fever and diarrhoea and will hasten recovery. In addition, concerns in some that patients may not always complete the full course, e.g. Vietnam<br>• However – some acknowledging that inappropriate and high use of antibiotics increases AMR<br>• Generally strong social influence to use/ request antibiotics and high expectations that antibiotics will be prescribed/ dispensed even for e.g. URTIs<br>• Raising awareness/ knowledge regarding antibiotics/ AMR including whether antibiotics effective in treating URTIs and whether AMR can be spread between people can help limit requests for antibiotics. However, concerns about the effectiveness of education campaigns in practice (e.g. Lao)<br>• More educated families/ those with higher incomes or those patients with a medical background greater awareness of antibiotics/ AMR and less likely to self-medicate with antibiotics/ request these from physicians<br>• Language can be an issue when discussing key issues surrounding antibiotics/ AMR

use of metronidazole across a range of infections as seen as working and inexpensive vs. other antibiotics<br>• Concerns that patients in a number of African countries stop taking antibiotics when they feel better, will only purchase amounts they can afford and use/ share left over antibiotics – however, not universal e.g. Madagascar<br>• In addition, concerns from patients regarding what they can do in practice to reduce AMR (although not universal)<br>c) No obvious differences in responses from patients by country income levels		c) No obvious differences by country income levels	with concerns with terminology in some countries/ dialects<br>*b) Practice*<br>• Typically high rates of self-purchasing of antibiotics across countries/ asking for antibiotics – although in some only when symptoms get worse, e.g. China. This includes for URTIs, fever and diarrhoea to save costs and time<br>• Generally, 'Access' antibiotics most used – although higher use of cephalosporins in some countries<br>c) No obvious differences by country income levels

Table S21 – Suggested Activities among Physicians, Nurses and their Associations

**Short to Medium term (1 – 5 years)**	• Work with Governments/ Health Authorities to continue to ascertain/ research the KAP of prescribers regarding antibiotics, AMR, and ASPs, especially where there are existing concerns as part of NAPs to help attain UN GA goals. This can include working with Universities to instigate CPD activities where current knowledge is a concern<br>• Physicians, Nurses and their associations to work closely with Governments/ Health Authorities to refine national guidelines such as the WHO AWaRe book guidance taking account of local AMR patterns, and incorporate any refinements into any agreed quality indicators based on AWaRe guidance. This necessarily includes encouraging facilities across countries to monitor resistance patterns where concerns, as well as encouraging Governments/ Health Authorities to fund more surveillance facilities, as well as culture testing where co-payments are a concern<br>• Physician and Nursing Associations to work closely with prescribers to monitor current antibiotic prescribing patterns through audits as part of ASPs. This includes helping to develop and instigate agreed-upon quality indicators based on refined WHO AWaRe book guidance – ensuring that prescribers are not overwhelmed with the number of instigated quality indicators<br>• Monitoring activities across LMICs will be facilitated by the introduction and routine availability of electronic systems within primary care clinics, which is a concern currently with an appreciable amount of paper-based systems across the 4 WHO Regions<br>• Physician and Nursing Associations to work closely with Universities across the 4 WHO Regions to ensure HCPs are fully aware of AMS and ASPs and are confident with undertaking ASPs in their practice. Academicians in universities can also help with initiating and undertaking research to monitor the impact of any ASP, and their cost-effectiveness, to help refine future activities potentially with the help of groups such as BSAC in the UK (459)<br>• Alongside this, Physician and Nursing Associations to work closely with universities to ensure prescribers have the necessary communication skills when discussing the management of infectious diseases with patients given the level of misinformation among patients/ the public, and the pressures they apply to encourage prescribers and dispensers to provide antibiotics for them or their children for often self-limiting conditions such as URTIs (Table 13 – 16)
**Longer Term**	• Keep engaging with key stakeholder groups to continually monitor the KAP of prescribers, and instigate appropriate educational and ASP activities where concerns along with Universities. This includes the routine provision of antimicrobial resistance surveillance systems where concerns<br>• Physician and Nursing Associations to continually update and refine treatment guidance and quality indicators where concerns exist. This necessarily means the routine availability of electronic medical records within primary healthcare centres<br>• Physician and Nursing Associations to keep working with Universities and others to ensure good communication skills with patients given ongoing concerns, which can be part of ongoing CPD activities

Table S22 – Suggested Activities among Community Pharmacists and their Associations

**Short to Medium term (1 – 5 years)**	• Pharmacy Associations to work with Governments/ Health Authorities to continue to ascertain/ research the KAP of dispensers regarding antibiotics, AMR, and ASPs, especially where there are existing concerns as part of NAPs to help attain UNGA goals. This can include working with universities to instigate CPD activities where current knowledge is a concern<br>• Pharmacy Associations to work closely with dispensers to monitor current antibiotic dispensing patterns through audits as part of any planned ASP. This includes helping to develop and instigate agreed-upon quality indicators based on refined WHO AWaRe book guidance – ensuring that dispensers are not overwhelmed with the number of instigated quality indicators<br>• Monitoring activities across LMICs will necessarily include the introduction and routine availability of electronic systems as opposed to paper-based systems within pharmacies - potentially starting with mobile technologies<br>• Pharmacy Associations to work closely with Universities to ensure dispensers have the necessary communication skills when discussing the management of infectious diseases with patients given the level of misinformation among patients/ the public regarding the effectiveness of antibiotics to treat viral infections, and the pressures they apply to encourage prescribers and dispensers to provide antibiotics for them or their children for often self-limiting conditions such as URTIs (Table 13 – 16). This also includes ensuring that dispensers are aware of any language difficulties with patients in their understanding of key terms such as antibiotics and AMR – especially in LMICs where there are multiple local languages where translation can be a problem such as South Africa (369)<br>• Pharmacy Associations and Universities to explore the potential of working with groups such as the Commonwealth Pharmacy Association regarding possible educational activities including ASPs building on current programmes (460)
**Longer Term**	• Keep engaging with key stakeholder groups to continually monitor the KAP of dispensers, and instigate appropriate educational and ASP activities where concerns along with universities<br>• Pharmacy Associations to continually help update and refine treatment guidance and quality indicators. This necessarily means the routine availability of electronic surveillance systems among community pharmacies<br>• Pharmacy Associations to keep working with universities and others to ensure good communication skills with patients given ongoing concerns, which can be part of ongoing CPD activities with community pharmacists and dispensers

Table S23 – Suggested Activities among Universities

**Short to Medium term (1 – 5 years)**	• Universities to revise and update their curricula and teaching methods to ensure as far as possible that no student HCP leaves the University without a thorough knowledge regarding antibiotics, AMR and ASPs as well as the WHO AWaRe classification and guidance given current concerns as well as the new UN GA goals. This is important given the problems lockdown measures caused the education of HCP students (461); however, this is now changing. As part of this, regularly conduct KAP research among HCP students of all years to assess the effectiveness of current curricula activities<br>• Universities to work with Governments/ Health Authorities as well as Physician, Nurse and Pharmacy Associations to ensure their courses meet the needs of HCPs once qualified and continued with CPD activities post-qualification<br>• Universities/ Academics to also work with key groups to undertake regular research to monitor the KAP of all key stakeholders as well as help them undertake ASPs to improve future antibiotic utilisation. This includes assessing the cost-effectiveness of implemented ASPs to provide future direction to all key stakeholder groups<br>• Alongside this, work with key groups to help refine future quality indicators as well as their impact in reality to improve future prescribing and dispensing to help attain UN GA goals<br>• Universities to work with Patient Associations to assess the impact and cost-effectiveness of any educational campaign targeted at patients/ public, as well as research into social media and their impact given the lack of studies to date in this area in LMICs. This also includes potential ways to address misinformation regarding antibiotics and AMR emanating from key social media platforms
**Longer Term**	• Universities to continually assess the KAP of their students regarding antibiotics, AMR and AMS as well as continue with others to monitor the KAP of other key stakeholder groups

	• Continue to work with Physician, Nurse and Pharmacy Associations to conduct research to monitor the KAP of their groups as well as help them with researching to assess the effectiveness and cost-effectiveness of any ASP activities<br>• Similarly with Patient Associations and others to monitor the effectiveness and cost-effectiveness of future educational programmes

Table S24 – Suggested Activities among Patients and Patient Associations

Short to Medium term (1 – 5 years)	• National Patient Associations to work with Governments/ Health Authorities to continue to ascertain/ research the KAP of patients and the public regarding antibiotics, AMR, and ASPs, especially given the extent of existing concerns (Tables 9 – 12) to help attain NAP and UN GA goals. This can include working with universities to undertake necessary research activities<br>• National Patient Associations to work alongside Governments/ Health Authorities, Physician/ Nursing Associations and Pharmacy Associations to help improve effective communication with patients to enhance their understanding of antibiotics and AMR as well as reduce the level of inappropriate requests. This includes the potential use of animations for children and others to reduce unnecessary requests for antibiotics (462)<br>• Alongside this, work with Governments/ Health Authorities and Universities to assess the impact of any national or local educational campaign on subsequent knowledge and activities of patients/ public regarding knowledge and practice concerning antibiotics and AMR including their cost-effectiveness. Subsequently, use the findings to guide future activities<br>• In addition, work with these key groups when assessing and refining possible quality indicators that directly affect patients and their care<br>• National Patient Associations to instigate programmes with the help of Governments/ Health Authorities to encourage more members of the public, including influential persons to become Antibiotic Guardians where such programmes exist. Alternatively, work with Governments/ Health Authorities to instigate such programmes where these are currently lacking to help improve future utilisation<br>• National Patient Associations and others to work with universities to continue to research the role of social media in spreading misinformation regarding antibiotics as well as instigate research with universities and others to assess the most appropriate social media platforms within countries to dispel myths regarding the effectiveness of antibiotics to treat viral diseases
Longer Term	• National Patient Associations and others continue to monitor the KAP of patients/ public with the help of others with respect to antibiotics and AMR<br>• Similarly, regarding any educational campaign with patients, their effectiveness and cost-effectiveness, and the media platforms used, to help guide future activities<br>• Continue to promote programmes such as Antibiotic Guardians as well as work with Governments/ Health Authorities and others concerning possible quality indicators to improve future antibiotic use among patients to achieve UNGA goals

References

1. Bulabula ANH, Jenkins A, Mehtar S, Nathwani D. Education and management of antimicrobials amongst nurses in Africa-a situation analysis: an Infection Control Africa Network (ICAN)/BSAC online survey. J Antimicrob Chemother. 2018;73(5):1408-15.
2. Allwell-Brown G, Hussain-Alkhateeb L, Kitutu FE, Strömdahl S, Mårtensson A, Johansson EW. Trends in reported antibiotic use among children under 5 years of age with fever, diarrhoea, or cough with fast or difficult breathing across low-income and middle-income countries in 2005-17: a systematic analysis of 132 national surveys from 73 countries. Lancet Glob Health. 2020;8(6):e799-e807.
3. Knowles R, Sharland M, Hsia Y, Magrini N, Moja L, Siyam A, et al. Measuring antibiotic availability and use in 20 low- and middle-income countries. Bull World Health Organ. 2020;98(3):177-87c.
4. Tesema GA, Biney GK, Wang VQ, Ameyaw EK, Yaya S. Antibiotic prescription sources and use among under-5 children with fever/cough in sub-Saharan Africa. Int Health. 2024.
5. Bezie MM, Asmare ZA, Asebe HA, Lombebo AA, Fentie BM, Asnake AA, et al. Factors associated with the use of antibiotics for children presenting with illnesses with fever and cough obtained from prescription and non-prescription sources: a cross-sectional study of data for 37 sub-Saharan African countries. BMC Public Health. 2024;24(1):1089.
6. Sié A, Coulibaly B, Adama S, Ouermi L, Dah C, Tapsoba C, et al. Antibiotic Prescription Patterns among Children Younger than 5 Years in Nouna District, Burkina Faso. Am J Trop Med Hyg. 2019;100(5):1121-4.
7. Sié A, Ouattara M, Bountogo M, Dah C, Compaoré G, Boudo V, et al. Indication for Antibiotic Prescription Among Children Attending Primary Healthcare Services in Rural Burkina Faso. Clin Infect Dis. 2021;73(7):1288-91.
8. Valia D, Ingelbeen B, Nassa GJW, Kaboré B, Kiemdé F, Rouamba T, et al. Antibiotic use by clinical presentation across all healthcare providers in rural Burkina Faso: a healthcare visit exit survey. J Antimicrob Chemother. 2024.
9. Wieters I, Johnstone S, Makiala-Mandanda S, Poda A, Akoua-Koffi C, Abu Sin M, et al. Reported antibiotic use among patients in the multicenter ANDEMIA infectious diseases surveillance study in sub-saharan Africa. Antimicrob Resist Infect Control. 2024;13(1):9.
10. Ingelbeen B, Phanzu DM, Phoba MF, Budiongo MYN, Berhe NM, Kamba FK, et al. Antibiotic use from formal and informal healthcare providers in the Democratic Republic of Congo: a population-based study in two health zones. Clin Microbiol Infect. 2022;28(9):1272-7.
11. Kakumba JM, Kindenge JM, Kapepula PM, Iyamba JL, Mashi ML, Mulwahali JW, et al. Evaluation of Antibiotic Prescribing Pattern Using WHO Access, Watch and Reserve Classification in Kinshasa, Democratic Republic of Congo. Antibiotics. 2023;12(8).
12. Abdu N, Idrisnur S, Tewelde T, Tesfamariam EH. Antibiotic prescribing practice using WHO Access, Watch and Reserve classification and its determinants among outpatient prescriptions dispensed to elderly population in six community chain pharmacies in Asmara, Eritrea: a cross-sectional study. BMJ Open. 2024;14(6):e085743.
13. Ness TE, Streatfield AE, Simelane T, Korsa A, Dlamini S, Guffey D, et al. Evaluating antibiotic use and developing a tool to optimize prescribing in a family-centered HIV clinic in Eswatini. PLoS One. 2021;16(1):e0244247.
14. Wendie TF, Ahmed A, Mohammed SA. Drug use pattern using WHO core drug use indicators in public health centers of Dessie, North-East Ethiopia. BMC Med Inform Decis Mak. 2021;21(1):197.
15. Dereje B, Workneh A, Megersa A, Yibabie S. Prescribing Pattern and Associated Factors in Community Pharmacies: A Cross-Sectional Study Using AWaRe Classification and WHO Antibiotic Prescribing Indicators in Dire Dawa, Ethiopia. Drugs Real World Outcomes. 2023;10(3):459-469.
16. Abebe RB, Ayal BM, Alemu MA, Zeleke TK. Antibiotic appropriateness at outpatient settings in Ethiopia: the need for an antibiotic stewardship programme. Drugs Context. 2024;13.
17. Abejew AA, Wubetu GY, Fenta TG. Antibiotic Prescribing Behavior of Physicians in Outpatient Departments in Hospitals in Northwest Ethiopia: Structural Equation Modeling Approach. Interact J Med Res. 2024;13:e57285.
18. Altaye FW, Thupayagale-Tshweneagae G, Mfidi FH. Qualitative enquiry on factors affecting antibiotic prescribing at primary healthcare facilities in Addis Ababa, Ethiopia. Front Med. 2024;11:1308699.
19. Eibs T, Koscalova A, Nair M, Grohma P, Kohler G, Bakhit RG, et al. Qualitative study of antibiotic prescription patterns and associated drivers in Sudan, Guinea-Bissau, Central African Republic and Democratic Republic of Congo. BMJ Open. 2020;10(9):e036530.

20. Ardillon A, Ramblière L, Kermorvant-Duchemin E, Sok T, Zo AZ, Diouf JB, et al. Inappropriate antibiotic prescribing and its determinants among outpatient children in 3 low- and middle-income countries: A multicentric community-based cohort study. PLoS Med. 2023;20(6):e1004211.
21. Dixon J, MacPherson EE, Nayiga S, Manyau S, Nabirye C, Kayendeke M, et al. Antibiotic stories: a mixed-methods, multi-country analysis of household antibiotic use in Malawi, Uganda and Zimbabwe. BMJ Glob Health. 2021;6(11).
22. MacPherson EE, Reynolds J, Sanudi E, Nkaombe A, Phiri C, Mankhomwa J, et al. Understanding antimicrobial resistance through the lens of antibiotic vulnerabilities in primary health care in rural Malawi. Glob Public Health. 2021:1-17.
23. Kabba JA, Tadesse N, James PB, Kallon H, Kitchen C, Atif N, et al. Knowledge, attitude and antibiotic prescribing patterns of medical doctors providing free healthcare in the outpatient departments of public hospitals in Sierra Leone: a national cross-sectional study. Trans R Soc Trop Med Hyg. 2020;114(6):448-58.
24. Koroma AT, Bundu PM, Sheriff M, Baryon B, Gamaga B, Sillah F, et al. Behavioral practices towards antibiotic use among health care workers - Sierra Leone, 2021: a facility-based cross-sectional study. Pan Afr Med J. 2024;47:63.
25. Lyus R, Pollock A, Ocan M, Brhlikova P. Registration of antimicrobials, Kenya, Uganda and United Republic of Tanzania, 2018. Bull World Health Organ. 2020;98(8):530-8.
26. Bonniface M, Nambatya W, Rajab K. An Evaluation of Antibiotic Prescribing Practices in a Rural Refugee Settlement District in Uganda. Antibiotics. 2021;10(2).
27. Kagoya EK, Royen KV, Waako P, Royen PV, Iramiot JS, Obakiro SB, et al. Experiences and views of healthcare professionals on the prescription of antibiotics in Eastern Uganda: A qualitative study. J Glob Antimicrob Resist. 2021;25:66-71.
28. Allwell-Brown G, Namugambe JS, Ssanyu JN, Johansson EW, Hussain-Alkhateeb L, Strömdahl S, et al. Patterns and contextual determinants of antibiotic prescribing for febrile under-five outpatients at primary and secondary healthcare facilities in Bugisu, Eastern Uganda. JAC Antimicrob Resist. 2022;4(5):dlac091.
29. Obakiro SB, Napyo A, Wilberforce MJ, Adongo P, Kiyimba K, Anthierens S, et al. Are antibiotic prescription practices in Eastern Uganda concordant with the national standard treatment guidelines? A cross-sectional retrospective study. J Glob Antimicrob Resist. 2022;29:513-9.
30. Mambula G, Nanjebe D, Munene A, Guindo O, Salifou A, Mamaty AA, et al. Practices and challenges related to antibiotic use in paediatric treatment in hospitals and health centres in Niger and Uganda: a mixed methods study. Antimicrob Resist Infect Control. 2023;12(1):67.
31. Murungi M, Ndagije HB, Kiggundu R, Kesi DN, Waswa JP, Rajab K, et al. Antimicrobial consumption surveillance in Uganda: Results from an analysis of national import data for the human health sector, 2018-2021. J Infect Public Health. 2023;16 Suppl 1:45-51.
32. Igirikwayo ZK, Migisha R, Mukaga H, Kabakyenga J. Prescription patterns of antibiotics and associated factors among outpatients diagnosed with respiratory tract infections in Jinja city, Uganda, June 2022-May 2023. BMC Pulm Med. 2024;24(1):446.
33. Lundin V, Lam F, Akinjeji A, Kabunga L, Eriksen J. Prevalence of antibiotic use for childhood diarrhoea in Uganda after an ORS scale-up intervention: a repeated cross-sectional study. BMC Public Health. 2024;24(1):2084.
34. Olamijuwon E, Keenan K, Mushi MF, Kansiime C, Konje ET, Kesby M, et al. Treatment seeking and antibiotic use for urinary tract infection symptoms in the time of COVID-19 in Tanzania and Uganda. J Glob Health. 2024;14:05007.
35. Kalungia AC, Mwambula H, Munkombwe D, Marshall S, Schellack N, May C, et al. Antimicrobial stewardship knowledge and perception among physicians and pharmacists at leading tertiary teaching hospitals in Zambia: implications for future policy and practice. J Chemother. 2019;31(7-8):378-87.
36. Mudenda S, Chabalenge B, Daka V, Jere E, Sefah I, Wesangula E, et al. Knowledge, awareness and practices of healthcare workers regarding antimicrobial use, resistance and stewardship in Zambia: a multi-facility cross-sectional study. JAC Antimicrob Resist. 2024;6(3):dlae076.
37. Yamba K, Mudenda S, Mpabalwani E, Mainda G, Mukuma M, Samutela MT, et al. Antibiotic prescribing patterns and carriage of antibiotic-resistant Escherichia coli and Enterococcus species in healthy individuals from selected communities in Lusaka and Ndola districts, Zambia. JAC Antimicrob Resist. 2024;6(2):dlae027.
38. Olaru ID, Ferrand RA, Yeung S, Chingono R, Chonzi P, Masunda KPE, et al. Knowledge, attitudes and practices relating to antibiotic use and resistance among prescribers from public primary healthcare facilities in Harare, Zimbabwe. Wellcome Open Res. 2021;6:72.

39. Olaru ID, Chisenga M, Yeung S, Mabey D, Marks M, Chonzi P, et al. Sexually transmitted infections and prior antibiotic use as important causes for negative urine cultures among adults presenting with urinary tract infection symptoms to primary care clinics in Zimbabwe: a cross-sectional study. BMJ Open. 2021;11(8):e050407.
40. Olaru ID, Elamin W, Chisenga M, Malou N, Piton J, Yeung S, et al. Evaluation of the InTray and Compact Dry culture systems for the diagnosis of urinary tract infections in patients presenting to primary health clinics in Harare, Zimbabwe. Eur J Clin Microbiol Infect Dis. 2021;40(12):2543-50.
41. Olaru ID, Chisenga M, Yeung S, Chonzi P, Masunda KPE, Ferrand RA, et al. Clinical and bacteriological outcomes in patients with urinary tract infections presenting to primary care in Harare, Zimbabwe: a cohort study. Wellcome Open Res. 2021;6:135.
42. Chem ED, Anong DN, Akoachere JKT. Prescribing patterns and associated factors of antibiotic prescription in primary health care facilities of Kumbo East and Kumbo West Health Districts, North West Cameroon. PLoS One. 2018;13(3):e0193353.
43. Menkem EZ, Labo Nanfah A, Takang T, Ryan Awah L, Awah Achua K, Ekane Akume S, et al. Attitudes and Practices of the Use of Third-Generation Cephalosporins among Medical Doctors Practicing in Cameroon. Int J Clin Pract. 2023;2023:8074413.
44. Ghebrehewet S, Shepherd W, Panford-Quainoo E, Shantikumar S, Decraene V, Rajendran R, et al. Implementation of a Delayed Prescribing Model to Reduce Antibiotic Prescribing for Suspected Upper Respiratory Tract Infections in a Hospital Outpatient Department, Ghana. Antibiotics. 2020;9(11).
45. Opoku MM, Bonful HA, Koram KA. Antibiotic prescription for febrile outpatients: a health facility-based secondary data analysis for the Greater Accra region of Ghana. BMC Health Serv Res. 2020;20(1):978.
46. Sefah IA, Essah DO, Kurdi A, Sneddon J, Alalbila TM, Kordorwu H, et al. Assessment of adherence to pneumonia guidelines and its determinants in an ambulatory care clinic in Ghana: findings and implications for the future. JAC Antimicrob Resist. 2021;3(2):dlab080.
47. Owusu H, Thekkur P, Ashubwe-Jalemba J, Hedidor GK, Corquaye O, Aggor A, et al. Compliance to Guidelines in Prescribing Empirical Antibiotics for Individuals with Uncomplicated Urinary Tract Infection in a Primary Health Facility of Ghana, 2019-2021. Int J Environ Res Public Health. 2022;19(19).
48. Pinto Jimenez C, Pearson M, Hennessey M, Nkereuwem E, Crocker C, Egbujo U, et al. Awareness of antibiotic resistance: a tool for measurement among human and animal health care professionals in LMICs and UMICs. J Antimicrob Chemother. 2023;78(3):620-35.
49. Sefah IA, Chetty S, Yamoah P, Meyer JC, Chigome A, Godman B, et al. A Multicenter Cross-Sectional Survey of Knowledge, Attitude, and Practices of Healthcare Professionals towards Antimicrobial Stewardship in Ghana: Findings and Implications. Antibiotics. 2023;12(10):1497.
50. Sefah IA, Quagraine AM, Kurdi A, Mudenda S, Godman B. Audit of antibiotic utilization patterns and practice for common eye infections at the ambulatory clinic of a teaching hospital in Ghana: Findings and implications. PLoS One. 2024;19(10):e0313019.
51. Kleczka B, Kumar P, Njeru MK, Musiega A, Wekesa P, Rabut G, et al. Using rubber stamps and mobile phones to help understand and change antibiotic prescribing behaviour in private sector primary healthcare clinics in Kenya. BMJ Glob Health. 2019;4(5):e001422.
52. Mekuria LA, de Wit TF, Spieker N, Koech R, Nyarango R, Ndwiga S, et al. Analyzing data from the digital healthcare exchange platform for surveillance of antibiotic prescriptions in primary care in urban Kenya: A mixed-methods study. PLoS One. 2019;14(9):e0222651.
53. Rhee C, Aol G, Ouma A, Audi A, Muema S, Auko J, et al. Inappropriate use of antibiotics for childhood diarrhea case management — Kenya, 2009–2016. BMC Public Health. 2019;19(3):468.
54. Sulis G, Daniels B, Kwan A, Gandra S, Daftary A, Das J, et al. Antibiotic overuse in the primary health care setting: a secondary data analysis of standardised patient studies from India, China and Kenya. BMJ Glob Health. 2020;5(9).
55. Hooft AM, Ndenga B, Mutuku F, Otuka V, Ronga C, Chebii PK, et al. High Frequency of Antibiotic Prescription in Children With Undifferentiated Febrile Illness in Kenya. Clin Infect Dis. 2021;73(7):e2399-e406.
56. Kwan A, Boone CE, Sulis G, Gertler PJ. Do private providers give patients what they demand, even if it is inappropriate? A randomised study using unannounced standardised patients in Kenya. BMJ Open. 2022;12(3):e058746.
57. Chukwu EE, Oladele DA, Enwuru CA, Gogwan PL, Abuh D, Audu RA, et al. Antimicrobial resistance awareness and antibiotic prescribing behavior among healthcare workers in Nigeria: a national survey. BMC Infect Dis. 2021;21(1):22.

58. Ogaji DS, Nwaejike D, Ebiekuraju O. Quality of Drug Prescribing and Dispensing Practices in Primary Healthcare Centres in an Urban Local Government Area in Nigeria. West Afr J Med. 2023;40(9):925-34.
59. Chukwu EE, Abuh D, Idigbe IE, Osuolale KA, Chuka-Ebene V, Awoderu O, et al. Implementation of antimicrobial stewardship programs: A study of prescribers' perspective of facilitators and barriers. PLoS One. 2024;19(1):e0297472.
60. Lyimo SR, Sigalla GN, Emidi B, Mgabo MR, Kajeguka DC. Cross-sectional Survey on Antibiotic Prescription Practices Among Health Care Providers in Rombo District, Northern Tanzania. East Afr Health Res J. 2018;2(1):10-7.
61. Emgård M, Mwangi R, Mayo C, Mshana E, Nkini G, Andersson R, et al. Tanzanian primary healthcare workers' experiences of antibiotic prescription and understanding of antibiotic resistance in common childhood infections: a qualitative phenomenographic study. Antimicrob Resist Infect Control. 2021;10(1):94.
62. Khalfan MA, Sasi PG, Mugusi SF. The prevalence and pattern of antibiotic prescription among insured patients in Dar es Salaam Tanzania. Pan Afr Med J. 2021;40:140.
63. Kilipamwambu A, Bwire GM, Myemba DT, Njiro BJ, Majigo MV. WHO/INRUD core prescribing indicators and antibiotic utilization patterns among primary health care facilities in Ilala district, Tanzania. JAC Antimicrob Resist. 2021;3(2):dlab049.
64. van de Maat J, De Santis O, Luwanda L, Tan R, Keitel K. Primary Care Case Management of Febrile Children: Insights From the ePOCT Routine Care Cohort in Dar es Salaam, Tanzania. Front Pediatr. 2021;9:626386.
65. Wiedenmayer K, Ombaka E, Kabudi B, Canavan R, Rajkumar S, Chilunda F, et al. Adherence to standard treatment guidelines among prescribers in primary healthcare facilities in the Dodoma region of Tanzania. BMC Health Serv Res. 2021;21(1):272.
66. Khalfan MA, Sasi P, Mugusi S. Factors influencing receipt of an antibiotic prescription among insured patients in Tanzania: a cross-sectional study. BMJ Open. 2022;12(11):e062147.
67. Mabilika RJ, Shirima G, Mpolya E. Prevalence and Predictors of Antibiotic Prescriptions at Primary Healthcare Facilities in the Dodoma Region, Central Tanzania: A Retrospective, Cross-Sectional Study. Antibiotics. 2022;11(8):1035.
68. Virhia J, Gilmour M, Russell C, Mutua E, Nasuwa F, Mmbaga BT, et al. “If You Do Not Take the Medicine and Complete the Dose…It Could Cause You More Trouble”: Bringing Awareness, Local Knowledge and Experience into Antimicrobial Stewardship in Tanzania. Antibiotics. 2023;12(2):243.
69. Lamshöft MM, Liheluka E, Ginski G, Lusingu JPA, Minja D, Gesase S, et al. Understanding pre-hospital disease management of fever and diarrhoea in children-Care pathways in rural Tanzania. Trop Med Int Health. 2024;29(8):706-14.
70. Hadley MB, Beard J. Is 'Health for All' synonymous with 'antibiotics for all': changes in antibiotic prescribing in a performance-based financing pilot in Zanzibar. Health Policy Plan. 2019;34(Supplement_2):ii28-ii35.
71. Adegbite BR, Edoa JR, Schaumburg F, Alabi AS, Adegnika AA, Grobusch MP. Knowledge and perception on antimicrobial resistance and antibiotics prescribing attitude among physicians and nurses in Lambaréné region, Gabon: a call for setting-up an antimicrobial stewardship program. Antimicrob Resist Infect Control. 2022;11(1):44.
72. Niaz Q, Godman B, Campbell S, Kibuule D. Compliance to prescribing guidelines among public health care facilities in Namibia; findings and implications. Int J Clin Pharm. 2020;42(4):1227-36.
73. Farley E, Stewart A, Davies MA, Govind M, Van den Bergh D, Boyles TH. Antibiotic use and resistance: Knowledge, attitudes and perceptions among primary care prescribers in South Africa. S Afr Med J. 2018;108(9):763-71.
74. Gasson J, Blockman M, Willems B. Antibiotic prescribing practice and adherence to guidelines in primary care in the Cape Town Metro District, South Africa. S Afr Med J. 2018;108(4):304-10.
75. Truter I, Knoesen BC. Perceptions towards the prescribing of antibiotics by pharmacists and the use of antibiotics in primary care in South Africa. J Infect Dev Ctries. 2018;12(2):115-9.
76. van Hecke O, Butler C, Mendelson M, Tonkin-Crine S. Introducing new point-of-care tests for common infections in publicly funded clinics in South Africa: a qualitative study with primary care clinicians. BMJ Open. 2019;9(11):e029260.
77. Sharma S, Tandlich R, Docrat M, Srinivas S. Antibiotic procurement and ABC analysis for a comprehensive primary health care clinic in the Eastern Cape province, South Africa. S Afr J Infect Dis. 2020;35(1):134.

78. Balliram R, Sibanda W, Essack SY. The knowledge, attitudes and practices of doctors, pharmacists and nurses on antimicrobials, antimicrobial resistance and antimicrobial stewardship in South Africa. S Afr J Infect Dis. 2021;36(1):262.
79. Engler D, Meyer JC, Schellack N, Kurdi A, Godman B. Antimicrobial Stewardship Activities in Public Healthcare Facilities in South Africa: A Baseline for Future Direction. Antibiotics. 2021;10(8).
80. Govender T, Suleman F, Perumal-Pillay VA. Evaluating the implementation of the standard treatment guidelines (STGs) and essential medicines list (EML) at a public South African tertiary institution and its associated primary health care (PHC) facilities. J Pharm Policy Pract. 2021;14(1):105.
81. Alabi ME, Essack SY. Antibiotic prescribing amongst South African general practitioners in private practice: an analysis of a health insurance database. JAC Antimicrob Resist. 2022;4(5):dlac101.
82. De Vries E, Johnson Y, Willems B, Bedeker W, Ras T, Coetzee R, et al. Improving primary care antimicrobial stewardship by implementing a peer audit and feedback intervention in Cape Town community healthcare centres. S Afr Med J. 2022;112(10):812-8.
83. Guma SP, Godman B, Campbell SM, Mahomed O. Determinants of the Empiric Use of Antibiotics by General practitioners in South Africa: Observational, Analytic, Cross-Sectional Study. Antibiotics. 2022;11(10):1423.
84. Keuler N, Johnson Y, Coetzee R. Treating urinary tract infections in public sector primary healthcare facilities in Cape Town, South Africa: A pharmaceutical perspective. S Afr Med J. 2022;112(7):487-93.
85. Lagarde M, Blaauw D. Levels and determinants of overprescribing of antibiotics in the public and private primary care sectors in South Africa. BMJ Glob Health. 2023;8(7).
86. Blaauw D, Lagarde M. New study finds very high rate of unnecessary antibiotic prescribing in SA. 2019. Available at URL: https://www.wits.ac.za/news/latest-news/research-news/2019/2019-03/new-study-finds-very-high-rate-of-unnecessary-antibiotic-prescribing-in-sa-.html
87. Maraqa B, Nazzal Z, Hamshari S, Matani N, Assi Y, Aabed M, et al. Palestinian physicians' self-reported practice regarding antibiotic use for upper respiratory tract infections in primary healthcare. Front Med. 2023;10:1139871.
88. Orubu ESF, Al-Dheeb N, Ching C, Bu Jawdeh S, Anderson J, Sheikh R, et al. Assessing Antimicrobial Resistance, Utilization, and Stewardship in Yemen: An Exploratory Mixed-Methods Study. Am J Trop Med Hyg. 2021;105(5):1404-12.
89. Othman G, Halboup A, Alzoubi K. Awareness of Bacterial Resistance to Antibiotics among Healthcare Providers in Sana'a City, Yemen. Yemeni Journal for Medical Sciences. 2018;18.
90. Kandeel A, Palms DL, Afifi S, Kandeel Y, Etman A, Hicks LA, et al. An educational intervention to promote appropriate antibiotic use for acute respiratory infections in a district in Egypt- pilot study. BMC Public Health. 2019;19(Suppl 3):498.
91. El-Sokkary R, Kishk R, Mohy El-Din S, Nemr N, Mahrous N, Alfishawy M, et al. Antibiotic Use and Resistance Among Prescribers: Current Status of Knowledge, Attitude, and Practice in Egypt. Infect Drug Resist. 2021;14:1209-18.
92. Amin MT, Abd El Aty MA, Ahmed SM, Elsedfy GO, Hassanin ES, El-Gazzar AF. Over prescription of antibiotics in children with acute upper respiratory tract infections: A study on the knowledge, attitude and practices of non-specialized physicians in Egypt. PLoS One. 2022;17(11):e0277308.
93. Waseem H, Ali J, Sarwar F, Khan A, Rehman HSU, Choudri M, et al. Assessment of knowledge and attitude trends towards antimicrobial resistance (AMR) among the community members, pharmacists/pharmacy owners and physicians in district Sialkot, Pakistan. Antimicrob Resist Infect Control. 2019;8:67.
94. Hashmi H, Sasoli NA, Sadiq A, Raziq A, Batool F, Raza S, et al. Prescribing Patterns for Upper Respiratory Tract Infections: A Prescription-Review of Primary Care Practice in Quetta, Pakistan and the Implications. Frontiers in Public Health. 2021;9.
95. Rakhshani NS, Kaljee LM, Khan MI, Prentiss T, Turab A, Mustafa A, et al. A Formative Assessment of Antibiotic Dispensing/Prescribing Practices and Knowledge and Perceptions of Antimicrobial Resistance (AMR) among Healthcare Workers in Lahore Pakistan. Antibiotics. 2022;11(10).
96. Mustafa ZU, Iqbal S, Asif HR, Salman M, Jabbar S, Mallhi TH, et al. Knowledge, Attitude and Practices of Self-Medication Including Antibiotics among Health Care Professionals during the COVID-19 Pandemic in Pakistan: Findings and Implications. Antibiotics. 2023;12(3):481.
97. Vakili-Arki H, Aalaei S, Farrokhi M, Nabovati E, Saberi MR, Eslami S. A survey of perceptions, attitudes and practices regarding the antibiotic prescription among Iranian physicians. Expert Review of Anti-infective Therapy. 2019;17(9):741-8.

98. Sami R, Sadegh R, Fani F, Atashi V, Solgi H. Assessing the knowledge, attitudes and practices of physicians on antibiotic use and antimicrobial resistance in Iran: a cross-sectional survey. J Pharm Policy Pract. 2022;15(1):82.
99. Aeenparast A, Haeri Mehrizi AA, Maftoon F, Farzadi F. Drug Prescription Indicators in Outpatient Services in Social Security Organization Facilities in Iran. J Prev Med Public Health. 2024;57(3):298-303.
100. Karasneh RA, Al-Azzam SI, Ababneh M, Al-Azzeh O, Al-Batayneh OB, Muflih SM, et al. Prescribers' Knowledge, Attitudes and Behaviors on Antibiotics, Antibiotic Use and Antibiotic Resistance in Jordan. Antibiotics. 2021;10(7).
101. Karasneh RA, Al-Azzam SI, Ababneh MA, Basheti IA, Al-Azzeh O, Al Sharie S, et al. Exploring Information Available to and Used by Physicians on Antibiotic Use and Antibiotic Resistance in Jordan. Antibiotics. 2021;10(8).
102. Orubu ESF, Albeik S, Ching C, Hussein R, Mousa A, Horino M, et al. A Survey Assessing Antimicrobial Prescribing at United Nations Relief and Works Agency Primary Health Care Centers in Jordan. Am J Trop Med Hyg. 2022;107(2):474-83.
103. Ababneh MA, Abujuma H, Altawalbeh S, Al Demour S. Evaluation of Antimicrobial Stewardship Programs and antibiotic prescribing patterns among physicians in ambulatory care settings in Jordan. Expert Rev Pharmacoecon Outcomes Res. 2024;24(3):405-12.
104. Abu-Farha R, Gharaibeh L, Alzoubi KH, Nazal R, Zawiah M, Binsaleh AY, et al. Awareness, perspectives and practices of antibiotics deprescribing among physicians in Jordan: a cross-sectional study. J Pharm Policy Pract. 2024;17(1):2378484.
105. Shrestha R, Prajapati S. Assessment of prescription pattern and prescription error in outpatient Department at Tertiary Care District Hospital, Central Nepal. J Pharm Policy Pract. 2019;12:16.
106. Aryal A, Dahal A, Shrestha R. Study on drug use pattern in primary healthcare centers of Kathmandu valley. SAGE Open Med. 2020;8:2050312120926437.
107. Ban B, Hodgins S, Thapa P, Thapa S, Joshi D, Dhungana A, et al. A national survey of private-sector outpatient care of sick infants and young children in Nepal. BMC Health Serv Res. 2020;20(1):545.
108. Gyanwali P, Dhakal N, Humagain B, Karki KB. Medicine Prescribing Pattern and Knowledge on Medicine Use at Different Level of Health Care Settings in Nepal. J Nepal Health Res Counc. 2020;18(3):520-4.
109. Nepal A, Hendrie D, Robinson S, Selvey LA. Analysis of patterns of antibiotic prescribing in public health facilities in Nepal. J Infect Dev Ctries. 2020;14(1):18-27.
110. Rijal KR, Banjara MR, Dhungel B, Kafle S, Gautam K, Ghimire B, et al. Use of antimicrobials and antimicrobial resistance in Nepal: a nationwide survey. Sci Rep. 2021;11(1):11554.
111. Shrestha JTM, Tiwari S, Kushwaha DK, Bhattarai P, Shrestha R. Drug Prescription in the Department of Medicine of a Tertiary Care Hospital according to the World Health Organization/International Network for Rational Use of Drugs Core Indicators: A Descriptive Cross-sectional Survey. J Nepal Med Assoc. 2021;59(240):745-8.
112. Zheng C, Karkey A, Wang T, Makuka G, van Doorn HR, Lewycka S. Determinants and patterns of antibiotic consumption for children under five in Nepal: analysis and modelling of Demographic Health Survey data from 2006 to 2016. Trop Med Int Health. 2021;26(4):397-409.
113. Nahar P, Unicomb L, Lucas PJ, Uddin MR, Islam MA, Nizame FA, et al. What contributes to inappropriate antibiotic dispensing among qualified and unqualified healthcare providers in Bangladesh? A qualitative study. BMC Health Serv Res. 2020;20(1):656.
114. Samir N, Hassan MZ, Biswas M, Chowdhury F, Akhtar Z, Lingam R, et al. Antibiotic Use for Febrile Illness among Under-5 Children in Bangladesh: A Nationally Representative Sample Survey. Antibiotics. 2021;10(10).
115. Bepari AK, Rabbi G, Shaon HR, Khan SI, Zahid ZI, Dalal K, et al. Factors Driving Antimicrobial Resistance in Rural Bangladesh: A Cross-Sectional Study on Antibiotic Use-Related Knowledge, Attitude, and Practice Among Unqualified Village Medical Practitioners and Pharmacy Shopkeepers. Adv Ther. 2023;40(8):3478-94.
116. Hanson OR, Khan, II, Khan ZH, Amin MA, Biswas D, Islam MT, et al. Identification, mapping, and self-reported practice patterns of village doctors in Sitakunda subdistrict, Bangladesh. J Glob Health. 2024;14:04185.
117. Mahmudul Islam AFM, Raihan MA, Ahmed KT, Islam MS, Nusrat NA, Hasan MA, et al. Prevalence of inappropriate antibiotic doses among pediatric patients of inpatient, outpatient, and emergency care units in Bangladesh: A cross-sectional study. PLOS Glob Public Health. 2024;4(9):e0003657.

118. Nair M, Tripathi S, Mazumdar S, Mahajan R, Harshana A, Pereira A, et al. Knowledge, attitudes, and practices related to antibiotic use in Paschim Bardhaman District: A survey of healthcare providers in West Bengal, India. PLoS One. 2019;14(5):e0217818.
119. Nair M, Tripathi S, Mazumdar S, Mahajan R, Harshana A, Pereira A, et al. "Without antibiotics, I cannot treat": A qualitative study of antibiotic use in Paschim Bardhaman district of West Bengal, India. PLoS One. 2019;14(6):e0219002.
120. Gautham M, Spicer N, Chatterjee S, Goodman C. What are the challenges for antibiotic stewardship at the community level? An analysis of the drivers of antibiotic provision by informal healthcare providers in rural India. Soc Sci Med. 2021;275:113813.
121. Chatterjee S, Hazra A, Chakraverty R, Shafiq N, Pathak A, Trivedi N, et al. Knowledge, attitude, and practice survey on antimicrobial use and resistance among Indian clinicians: A multicentric, cross-sectional study. Perspectives in clinical research. 2022;13(2):99-105.
122. Gautham M, Miller R, Rego S, Goodman C. Availability, Prices and Affordability of Antibiotics Stocked by Informal Providers in Rural India: A Cross-Sectional Survey. Antibiotics. 2022;11(4).
123. Mittal N, Goel P, Goel K, Sharma R, Nath B, Singh S, et al. Awareness Regarding Antimicrobial Resistance and Antibiotic Prescribing Behavior among Physicians: Results from a Nationwide Cross-Sectional Survey in India. Antibiotics. 2023;12(10):1496.
124. Debnath F, De RG, Chakraborty D, Majumdar A, Mukhopadhyay S, Sarkar MD, et al. Antimicrobial stewardship implementation in primary and secondary tier hospitals in India: interim findings from a need assessment study using mixed method design. Sci Rep. 2024;14(1):28068.
125. Alkaff RN, Kamigaki T, Saito M, Ariyanti F, Iriani DU, Oshitani H. Use of antibiotics for common illnesses among children aged under 5 years in a rural community in Indonesia: a cross-sectional study. Trop Med Health. 2019;47:45.
126. Christanti JV, Setiadi AP, Wibowo YI, Presley B, Halim SV, Setiawan E, et al. A cross-sectional assessment of Indonesian female health cadres' knowledge and attitude towards antibiotics. J Infect Dev Ctries. 2021;15(10):1453-61.
127. Wardani RL, Suharjono, Kuntaman, Widjaja A. Antibiotic use on acute respiratory tract infection nonpneumonia and nonspecific diarrhea in Primary Health Care Centre in Banjarbaru City, South Kalimantan, Indonesia. J Basic Clin Physiol Pharmacol. 2021;32(4):729-35.
128. Althaus T, Greer RC, Swe MMM, Cohen J, Tun NN, Heaton J, et al. Effect of point-of-care C-reactive protein testing on antibiotic prescription in febrile patients attending primary care in Thailand and Myanmar: an open-label, randomised, controlled trial. Lancet Glob Health. 2019;7(1):e119-e31.
129. Swe MMM, Ashley EA, Althaus T, Lubell Y, Smithuis F, McLean ARD. Inter-prescriber variability in the decision to prescribe antibiotics to febrile patients attending primary care in Myanmar. JAC Antimicrob Resist. 2021;3(1):dlaa118.
130. Shu G, Jayawardena K, Jayaweera Patabandige D, Tennegedara A, Liyanapathirana V. Knowledge, perceptions and practices on antibiotic use among Sri Lankan doctors. PLoS One. 2022;17(2):e0263167.
131. Harris L, Bongers A, Yan J, Francis JR, Marr I, Lake S, et al. Estimates of Antibacterial Consumption in Timor-Leste Using Distribution Data and Variation in Municipality Usage Patterns. Antibiotics. 2021;10(12).
132. Keohavong B, Vonglokham M, Phoummalaysith B, Louangpradith V, Inthaphatha S, Kariya T, et al. Antibiotic prescription for under-fives with common cold or upper respiratory tract infection in Savannakhet Province, Lao PDR. Trop Med Health. 2019;47:16.
133. Sychareun V, Sihavong A, Machowska A, Onthongdee X, Chaleunvong K, Keohavong B, et al. Knowledge, Attitudes, Perception and Reported Practices of Healthcare Providers on Antibiotic Use and Resistance in Pregnancy, Childbirth and Children under Two in Lao PDR: A Mixed Methods Study. Antibiotics. 2021;10(12).
134. Zamunu A, Pameh W, Ripa P, Vince J, Duke T. Antibiotic use in the management of children with the common cold at a provincial hospital in Papua New Guinea: a point-prevalence study. Paediatr Int Child Health. 2018;38(4):261-5.
135. Berdida DJE, Grande RAN, Lopez V, Ramirez SH, Manting MME, Berdida MME, et al. A national online survey of Filipinos' knowledge, attitude, and awareness of antibiotic use and resistance: A cross-sectional study. Nurs Forum. 2022;57(6):1299-313.
136. Di KN, Tay ST, Sri La Sri Ponnampalavanar S, Pham DT, Wong LP. Physician's Perspectives on Factors Influencing Antibiotic Resistance: A Qualitative Study in Vietnam. Healthcare. 2022;11(1).
137. Nguyen NV, Do NTT, Vu DTV, Greer RC, Dittrich S, Vandendorpe M, et al. Outpatient antibiotic prescribing for acute respiratory infections in Vietnamese primary care settings by the WHO AWaRe (Access,

Watch and Reserve) classification: An analysis using routinely collected electronic prescription data. Lancet Reg Health West Pac. 2023;30:100611.
138. Thi TVL, Canh Pham E, Dang-Nguyen DT. Evaluation of children's antibiotics use for outpatient pneumonia treatment in Vietnam. Braz J Infect Dis. 2024;28(4):103839.
139. Vu Minh D, Nguyen Thi Hong Y, Nagraj S, Do Thi Thuy N, Vu Thi Lan H, Nguyen Vinh N, et al. Determinants of antibiotic prescribing in primary care in Vietnam: a qualitative study using the Theoretical Domains Framework. Antimicrob Resist Infect Control. 2024;13(1):115.
140. Chang Y, Chusri S, Sangthong R, McNeil E, Hu J, Du W, et al. Clinical pattern of antibiotic overuse and misuse in primary healthcare hospitals in the southwest of China. PLoS One. 2019;14(6):e0214779.
141. Ding G, Vinturache A, Lu M. Addressing inappropriate antibiotic prescribing in China. Cmaj. 2019;191(6):E149-e50.
142. Liu C, Liu C, Wang D, Zhang X. Knowledge, Attitudes and Intentions to Prescribe Antibiotics: A Structural Equation Modeling Study of Primary Care Institutions in Hubei, China. Int J Environ Res Public Health. 2019;16(13).
143. Wushouer H, Wang Z, Tian Y, Zhou Y, Zhu D, Vuillermin D, et al. The impact of physicians' knowledge on outpatient antibiotic use: Evidence from China's county hospitals. Medicine. 2020;99(3):e18852.
144. Xu J, Wang X, Sun KS, Lin L, Zhou X. Parental self-medication with antibiotics for children promotes antibiotic over-prescribing in clinical settings in China. Antimicrob Resist Infect Control. 2020;9(1):150.
145. Xu R, Mu T, Jian W, Xu C, Shi J. Knowledge, Attitude, and Prescription Practice on Antimicrobials Use Among Physicians: A Cross-Sectional Study in Eastern China. Inquiry. 2021;58:469580211059984.
146. Wang W, Yu S, Zhou X, Wang L, He X, Zhou H, et al. Antibiotic prescribing patterns at children's outpatient departments of primary care institutions in Southwest China. BMC Prim Care. 2022;23(1):269.
147. Xu X, Zhang K, Ma H, Shen X, Chai J, Tang M, et al. Differences in service and antibiotics use following symptomatic respiratory tract infections between 2016 and 2021 in rural Anhui, China. Epidemiol Infect. 2022;150:e117.
148. Xue T, Liu C, Li Z, Liu J, Tang Y. Weighing patient attributes in antibiotic prescribing for upper respiratory tract infections: A discrete choice experiment on primary care physicians in Hubei Province, China. Front Public Health. 2022;10:1008217.
149. Fu M, Gong Z, Zhu Y, Li C, Zhou Y, Hu L, et al. Inappropriate antibiotic prescribing in primary healthcare facilities in China: a nationwide survey, 2017-2019. Clin Microbiol Infect. 2023;29(5):602-9.
150. He D, Li F, Wang J, Zhuo C, Zou G. Antibiotic prescription for children with acute respiratory tract infections in rural primary healthcare in Guangdong province, China: a cross-sectional study. BMJ Open. 2023;13(11):e068545.
151. Li C, Cui Z, Wei D, Zhang Q, Yang J, Wang W, et al. Trends and Patterns of Antibiotic Prescriptions in Primary Care Institutions in Southwest China, 2017-2022. Infect Drug Resist. 2023;16:5833-54.
152. Shen L, Wang T, Yin J, Sun Q, Dyar OJ. Clinical Uncertainty Influences Antibiotic Prescribing for Upper Respiratory Tract Infections: A Qualitative Study of Township Hospital Physicians and Village Doctors in Rural Shandong Province, China. Antibiotics. 2023;12(6).
153. Ooi ZY, Mohd Ghazali NA, Nik Zahari NJ, Chan HK, Md Noor N, Harun NL, et al. Patient profile and antibiotic use in a dedicated upper respiratory tract infection clinic based in a primary healthcare setting during COVID-19 pandemic in Malaysia: A cross sectional study. Malays Fam Physician. 2022;17(3):74-83.
154. Lim AH, Ab Rahman N, Nasarudin SNS, Velvanathan T, Fong MCC, Mohamad Yahaya AH, et al. A comparison between antibiotic utilisation in public and private community healthcare in Malaysia. BMC Public Health. 2024;24(1):79.
155. Lim AH, Ab Rahman N, Ong SM, Mohamad Azman SR, Mohd Rathi FZ, Ismail M, et al. Impact evaluation of guidelines on antibiotic utilisation & appropriateness in Malaysian public primary care: an interrupted time series analysis. J Pharm Policy Pract. 2024;17(1):2355666.
156. Ayele AA, Mekuria AB, Tegegn HG, Gebresillassie BM, Mekonnen AB, Erku DA. Management of minor ailments in a community pharmacy setting: Findings from simulated visits and qualitative study in Gondar town, Ethiopia. PLoS One. 2018;13(1):e0190583.
157. Erku DA, Aberra SY. Non-prescribed sale of antibiotics for acute childhood diarrhea and upper respiratory tract infection in community pharmacies: a 2 phase mixed-methods study. Antimicrob Resist Infect Control. 2018;7:92.
158. Belachew SA, Hall L, Selvey LA. Community drug retail outlet staff's knowledge, attitudes and practices towards non-prescription antibiotics use and antibiotic resistance in the Amhara region, Ethiopia with a focus on non-urban towns. Antimicrob Resist Infect Control. 2022;11(1):64.

159. Ayenew W, Tessema TA, Anagaw YK, Siraj EA, Zewdie S, Simegn W, et al. Prevalence and predictors of self-medication with antibiotics in Ethiopia: a systematic review and meta-analysis. Antimicrob Resist Infect Control. 2024;13(1):61.
160. Edessa D, Kumsa FA, Dinsa G, Oljira L. Drug providers’ perspectives on antibiotic misuse practices in eastern Ethiopia: a qualitative study. BMJ Open. 2024;14(8):e085352.
161. Torres NF, Solomon VP, Middleton LE. Pharmacists' practices for non-prescribed antibiotic dispensing in Mozambique. Pharm Pract. 2020;18(3):1965.
162. Torres NF, Solomon VP, Middleton LE. Identifying the commonly used antibiotics for self-medication in urban Mozambique: a qualitative study. BMJ Open. 2020;10(12):e041323.
163. Lanyero H, Eriksen J, Obua C, Stålsby Lundborg C, Nanzigu S, Katureebe A, et al. Use of antibacterials in the management of symptoms of acute respiratory tract infections among children under five years in Gulu, northern Uganda: Prevalence and determinants. PLoS One. 2020;15(6):e0235164.
164. Bagonza A, Kitutu FE, Peterson S, Mårtensson A, Mutto M, Awor P, et al. Effectiveness of peer-supervision on pediatric fever illness treatment among registered private drug sellers in East-Central Uganda: An interrupted time series analysis. Health Sci Rep. 2021;4(2):e284.
165. Kiragga AN, Najjemba L, Galiwango R, Banturaki G, Munyiwra G, Iwumbwe I, et al. Community purchases of antimicrobials during the COVID-19 pandemic in Uganda: An increased risk for antimicrobial resistance. PLOS Glob Public Health. 2023;3(2):e0001579.
166. Mudenda S HM, Saleem Z, Sadiq MJ, Banda M, Munkombwe D, Mwila C, Kasanga M, Zulu AC, Hangoma JM, Mufwambi W, Muungo LT, Kampamba RM, Bambala AM, Abdulrahman NM, Akram M, Hikaambo CN, Muma JB. Knowledge, Attitude, and Practices of Community Pharmacists on Antibiotic Resistance and Antimicrobial Stewardship in Lusaka, Zambia. J Biomed Res Environ Sci. 2021; 2(10):1005-14.
167. Afari-Asiedu S, Kinsman J, Boamah-Kaali E, Abdulai MA, Gyapong M, Sankoh O, et al. To sell or not to sell; the differences between regulatory and community demands regarding access to antibiotics in rural Ghana. J Pharm Policy Pract. 2018;11:30.
168. Afari-Asiedu S, Hulscher M, Abdulai MA, Boamah-Kaali E, Wertheim HFL, Asante KP. Stakeholders' perspectives on training over the counter medicine sellers and Community-based Health Planning and Services facilities to dispense antibiotics in Ghana. J Pharm Policy Pract. 2021;14(1):62.
169. Ngyedu EK, Acolatse J, Akafity G, Incoom R, Rauf A, Seaton RA, et al. Selling antibiotics without prescriptions among community pharmacies and drug outlets: a simulated client study from Ghana. Expert Review of Anti-infective Therapy. 2023;21(12):1373-82.
170. Greene HC, Makovi K, Abdul-Mumin R, Bansal A, Frimpong JA. Challenges in the distribution of antimicrobial medications in community dispensaries in Accra, Ghana. PLoS One. 2024;19(5):e0281699.
171. Muloi D, Fèvre EM, Bettridge J, Rono R, Ong'are D, Hassell JM, et al. A cross-sectional survey of practices and knowledge among antibiotic retailers in Nairobi, Kenya. J Glob Health. 2019;9(2):010412.
172. Kimathi G, Kiarie J, Njarambah L, Onditi J, Ojakaa D. A cross-sectional study of antimicrobial use among self-medicating COVID-19 cases in Nyeri County, Kenya. Antimicrob Resist Infect Control. 2022;11(1):111.
173. Opanga S, Rizvi N, Wamaitha A, Abebrese Sefah I, Godman BB. Availability of Medicines in Community Pharmacy to Manage Patients with COVID-19 in Kenya; Pilot Study and Implications. Sch Acad J Pharm. 2021;3:36-42.
174. Mukokinya MMA, Opanga S, Oluka M, Godman B. Dispensing of Antimicrobials in Kenya: A Cross-sectional Pilot Study and Its Implications. J Res Pharm Pract. 2018;7(2):77-82.
175. Gacheri J, Hamilton KA, Munywoki P, Wakahiu S, Kiambi K, Fèvre EM, et al. Antibiotic prescribing practices in community and clinical settings during the COVID-19 pandemic in Nairobi, Kenya. PLOS Glob Public Health. 2024;4(4):e0003046.
176. Abubakar U, Tangiisuran B. Knowledge and practices of community pharmacists towards non-prescription dispensing of antibiotics in Northern Nigeria. Int J Clin Pharm. 2020;42(2):756-64.
177. Akpan RM, Udoh EI, Akpan SE, Ozuluoha CC. Community pharmacists' management of self-limiting infections: a simulation study in Akwa Ibom State, South-South Nigeria. Afr Health Sci. 2021;21(2):576-84.
178. Poyongo BP, Sangeda RZ. Pharmacists' Knowledge, Attitude and Practice Regarding the Dispensing of Antibiotics without Prescription in Tanzania: An Explorative Cross-Sectional Study. Pharmacy. 2020;8(4).
179. Ndaki PM, Mushi MF, Mwanga JR, Konje ET, Ntinginya NE, Mmbaga BT, et al. Dispensing Antibiotics without Prescription at Community Pharmacies and Accredited Drug Dispensing Outlets in Tanzania: A Cross-Sectional Study. Antibiotics. 2021;10(8).

180. Myemba DT, Maganda BA, Kibwana UO, Nkinda L, Ndayishimiye P, Kilonzi M, et al. Profiling of antimicrobial dispensing practices in accredited drug dispensing outlets in Tanzania: a mixed-method cross-sectional study focusing on pediatric patients. BMC Health Serv Res. 2022;22(1):1575.
181. Ndaki PM, Mushi MF, Mwanga JR, Konje ET, Mugassa S, Manyiri MW, et al. Non-prescribed antibiotic dispensing practices for symptoms of urinary tract infection in community pharmacies and accredited drug dispensing outlets in Tanzania: a simulated clients approach. BMC Prim Care. 2022;23(1):287.
182. Ndaki PM, Mwanga JR, Mushi MF, Konje ET, Fredricks KJ, Kesby M, et al. Practices and motives behind antibiotics provision in drug outlets in Tanzania: A qualitative study. PLoS One. 2023;18(8):e0290638.
183. Sono TM, Yeika E, Cook A, Kalungia A, Opanga SA, Acolatse JEE, et al. Current rates of purchasing of antibiotics without a prescription across sub-Saharan Africa; rationale and potential programmes to reduce inappropriate dispensing and resistance. Expert Rev Anti Infect Ther. 2023;21(10):1025-55.
184. Kamati M, Godman B, Kibuule D. Prevalence of Self-Medication for Acute Respiratory Infections in Young Children in Namibia: Findings and Implications. J Res Pharm Pract. 2019;8(4):220-4.
185. Kibuule D, Nambahu L, Sefah IA, Kurdi A, Phuong TNT, Kwon H-Y, Godman B. Activities in Namibia to limit the prevalence and mortality from COVID-19 including community pharmacy activities and the implications. Sch Acad J Pharm. 2021;5:82-92.
186. Mokwele RN, Schellack N, Bronkhorst E, Brink AJ, Schweickerdt L, Godman B. Using mystery shoppers to determine practices pertaining to antibiotic dispensing without a prescription among community pharmacies in South Africa—a pilot survey. JAC-Antimicrobial Resistance. 2022;4(1).
187. Sono TM, Maluleke MT, Jelić AG, Campbell S, Marković-Peković V, Schellack N, et al. Potential Strategies to Limit Inappropriate Purchasing of Antibiotics without a Prescription in a Rural Province in South Africa: Pilot Study and the Implications. Advances in Human Biology. 2024;14(1):60-7.
188. Sono TM, Maluleke MT, Ramdas N, Jelic AG, Campbell S, Markovic-Pekovic V, et al. Pilot Study to Evaluate the Feasibility of a Patient Questionnaire for the Purpose of Investigating the Extent of Purchasing Antibiotics without a Prescription in a Rural Province in South Africa: Rationale and Implications. Advances in Human Biology. 2024;14(2):138-47.
189. Do NTT, Vu HTL, Nguyen CTK, Punpuing S, Khan WA, Gyapong M, et al. Community-based antibiotic access and use in six low-income and middle-income countries: a mixed-method approach. Lancet Glob Health. 2021;9(5):e610-e9.
190. Anstey Watkins J, Wagner F, Xavier Gómez-Olivé F, Wertheim H, Sankoh O, Kinsman J. Rural South African Community Perceptions of Antibiotic Access and Use: Qualitative Evidence from a Health and Demographic Surveillance System Site. Am J Trop Med Hyg. 2019;100(6):1378-90.
191. Abdelaziz AI, Tawfik AG, Rabie KA, Omran M, Hussein M, Abou-Ali A, et al. Quality of Community Pharmacy Practice in Antibiotic Self-Medication Encounters: A Simulated Patient Study in Upper Egypt. Antibiotics. 2019;8(2).
192. Zakaa El-Din M, Samy F, Mohamed A, Hamdy F, Yasser S, Ehab M. Egyptian community pharmacists' attitudes and practices towards antibiotic dispensing and antibiotic resistance; a cross-sectional survey in Greater Cairo. Curr Med Res Opin. 2019;35(6):939-46.
193. Elsayed AA, Darwish SF, Zewail MB, Mohammed M, Saeed H, Rabea H. Antibiotic misuse and compliance with infection control measures during COVID-19 pandemic in community pharmacies in Egypt. Int J Clin Pract. 2021;75(6):e14081.
194. Rehman IU, Asad MM, Bukhsh A, Ali Z, Ata H, Dujaili JA, et al. Knowledge and Practice of Pharmacists toward Antimicrobial Stewardship in Pakistan. Pharmacy. 2018;6(4).
195. Sarwar MR, Saqib A, Iftikhar S, Sadiq T. Knowledge of community pharmacists about antibiotics, and their perceptions and practices regarding antimicrobial stewardship: a cross-sectional study in Punjab, Pakistan. Infect Drug Resist. 2018;11:133-45.
196. Atif M, Asghar S, Mushtaq I, Malik I. Community pharmacists as antibiotic stewards: A qualitative study exploring the current status of Antibiotic Stewardship Program in Bahawalpur, Pakistan. Journal of Infection and Public Health. 2020;13(1):118-24.
197. Saleem Z, Hassali MA, Godman B, Fatima M, Ahmad Z, Sajid A, et al. Sale of WHO AWaRe groups antibiotics without a prescription in Pakistan: a simulated client study. J Pharm Policy Pract. 2020;13:26.
198. Akbar Z, Saleem Z, Shaukat A, Farrukh MJ. Perception and practices of community pharmacists towards antimicrobial stewardship in Lahore, Pakistan. J Glob Antimicrob Resist. 2021;25:157-61.
199. Khan FU, Khan FU, Hayat K, Ahmad T, Khan A, Chang J, et al. Knowledge, Attitude, and Practice on Antibiotics and Its Resistance: A Two-Phase Mixed-Methods Online Study among Pakistani Community Pharmacists to Promote Rational Antibiotic Use. Int J Environ Res Public Health. 2021;18(3).

200. Mustafa ZU, Nazir M, Majeed HK, Salman M, Hayat K, Khan AH, et al. Exploring Knowledge of Antibiotic Use, Resistance, and Stewardship Programs among Pharmacy Technicians Serving in Ambulatory Care Settings in Pakistan and the Implications. Antibiotics. 2022;11(7):921.
201. Hashmi A, Ul Haq MI, Malik M, Hussain A, Gajdács M, Jamshed S. Perceptions of community pharmacists regarding their role in antimicrobial stewardship in Pakistan: A way forward. Heliyon. 2023;9(4):e14843.
202. Nabeel M, Ali K, Sarwar MR, Waheed I. Assessment of knowledge, attitudes, and practices among community pharmacists in Lahore regarding antibiotic dispensing without prescription: A cross-sectional study. PLoS One. 2024;19(6):e0304361.
203. Rafi S, Anjum SM, Usman M, Nawaz HA, Chaudhry M, Babar ZU, et al. Availability of Access, Watch, and Reserve groups of essential antibiotics: a cross-sectional survey. Front Public Health. 2023;11:1251434.
204. Al-Halawa DA, Seir RA, Qasrawi R. Antibiotic Resistance Knowledge, Attitudes, and Practices among Pharmacists: A Cross-Sectional Study in West Bank, Palestine. Journal of Environmental and Public Health. 2023;2023:2294048.
205. Abdelrahman Hussain M, Osman Mohamed A, Sandel Abkar A, Siddig Mohamed F, Khider Elzubair H. Knowledge, Attitude and Practice of Community Pharmacists in Relation to Dispensing Antibiotics Without Prescription in Sudan: A Cross-sectional Study. Integr Pharm Res Pract. 2022;11:107-16.
206. Alkadhimi A, Dawood OT, Hassali MA. Dispensing of antibiotics in community pharmacy in Iraq: a qualitative study. Pharm Pract. 2020;18(4):2095.
207. Haddadin RN, Alsous M, Wazaify M, Tahaineh L. Evaluation of antibiotic dispensing practice in community pharmacies in Jordan: A cross sectional study. PLoS One. 2019;14(4):e0216115.
208. Darwish RM, Baqain GN, Aladwan H, Salamah LM, Madi R, Masri RMA. Knowledge, attitudes, and practices regarding antibiotic use and resistance among community pharmacists: a cross sectional study in Jordan. Int J Clin Pharm. 2021;43(5):1198-207.
209. Al-Taani GM, Al-Azzam S, Karasneh RA, Sadeq AS, Mazrouei NA, Bond SE, et al. Pharmacists' Knowledge, Attitudes, Behaviors and Information Sources on Antibiotic Use and Resistance in Jordan. Antibiotics. 2022;11(2).
210. Zahreddine L, Hallit S, Shakaroun S, Al-Hajje A, Awada S, Lahoud N. Knowledge of pharmacists and parents towards antibiotic use in pediatrics: a cross-sectional study in Lebanon. Pharm Pract. 2018;16(3):1194.
211. Sabra R, Safwan J, Dabbous M, Rida A, Malaeb D, Akel M, et al. Assessment of knowledge, attitude and practice of Lebanese pharmacists in providing patient counseling on urinary tract infection and its treatment. Pharm Pract. 2022;20(2):2653.
212. Al-Shami HA, Abubakar U, Hussein MSE, Hussin HFA, Al-Shami SA. Awareness, practices and perceptions of community pharmacists towards antimicrobial resistance and antimicrobial stewardship in Libya: a cross-sectional study. J Pharm Policy Pract. 2023;16(1):46.
213. Nepal A, Hendrie D, Robinson S, Selvey LA. Survey of the pattern of antibiotic dispensing in private pharmacies in Nepal. BMJ Open. 2019;9(10):e032422.
214. Goswami N, Dahal P, Shrestha S, Kc B, Mallik SK. Community Pharmacy Personnel Understanding of Antibiotic Dispensing in Eastern Nepal. Risk Manag Healthc Policy. 2020;13:1513-22.
215. Jha N, Shrestha S, Shankar PR, Khadka A, Ansari M, Sapkota B. Antibiotic Dispensing Practices at Community Pharmacies in Kathmandu and Lalitpur Districts of Nepal. Indian Journal of Pharmacy Practice. 2020;13:336
216. Koju P, Rousseau SP, Van der Putten M, Shrestha A, Shrestha R. Advertisement of antibiotics for upper respiratory infections and equity in access to treatment: a cross-sectional study in Nepal. J Pharm Policy Pract. 2020;13:4.
217. Acharya Y, Nepal P, Yang D, Karki K, Bajracharya D, Prentis T, et al. Economic and social drivers of antibiotic dispensing practices among community pharmacies in Nepal. Trop Med Int Health. 2021;26(5):557-71.
218. Adhikari B, Pokharel S, Raut S, Adhikari J, Thapa S, Paudel K, et al. Why do people purchase antibiotics over-the-counter? A qualitative study with patients, clinicians and dispensers in central, eastern and western Nepal. BMJ Glob Health. 2021;6(5).
219. Karki K, Kumar Neupane A, Raj Singh D. Antibiotic dispensing knowledge and practice among dispensing staff working in pharmacies near teaching hospitals in Kathmandu Valley, Nepal. Int J Pharm Pract. 2022;30(2):180-3.
220. Shrestha N, Manandhar S, Maharjan N, Twati D, Dongol S, Basnyat B, et al. Perspectives of pharmacy employees on an inappropriate use of antimicrobials in Kathmandu, Nepal. PLoS One. 2023;18(5):e0285287.

221. Marasini S, Sharma S, Joshi A, Kunwar S, Mahato RK, Shrestha A, et al. Exploring knowledge, perceptions, and practices of antimicrobials, and their resistance among medicine dispensers and community members in Kavrepalanchok District of Nepal. PLoS One. 2024;19(1):e0297282.
222. Darj E, Newaz MS, Zaman MH. Pharmacists' perception of their challenges at work, focusing on antimicrobial resistance: a qualitative study from Bangladesh. Glob Health Action. 2019;12(sup1):1735126.
223. Matin MA, Khan WA, Karim MM, Ahmed S, John-Langba J, Sankoh OA, et al. What influences antibiotic sales in rural Bangladesh? A drug dispensers' perspective. J Pharm Policy Pract. 2020;13:20.
224. Nizame FA, Shoaib DM, Rousham EK, Akter S, Islam MA, Khan AA, et al. Barriers and facilitators to adherence to national drug policies on antibiotic prescribing and dispensing in Bangladesh. Journal of Pharmaceutical Policy and Practice. 2021;14(1):85.
225. Orubu ESF, Samad MA, Rahman MT, Zaman MH, Wirtz VJ. Mapping the Antimicrobial Supply Chain in Bangladesh: A Scoping-Review-Based Ecological Assessment Approach. Glob Health Sci Pract. 2021;9(3):532-47.
226. Unicomb LE, Nizame FA, Uddin MR, Nahar P, Lucas PJ, Khisa N, et al. Motivating antibiotic stewardship in Bangladesh: identifying audiences and target behaviours using the behaviour change wheel. BMC Public Health. 2021;21(1):968.
227. Islam MA, Akhtar Z, Hassan MZ, Chowdhury S, Rashid MM, Aleem MA, et al. Pattern of Antibiotic Dispensing at Pharmacies According to the WHO Access, Watch, Reserve (AWaRe) Classification in Bangladesh. Antibiotics. 2022;11(2):247.
228. Ali SB, Chakma N, Islam MS, Amzad R, Khan MLH, Aziulla M, et al. Assessment of the impact of good pharmacy practices training among drug dispensers in Bangladesh. Front Pharmacol. 2023;14:1139632.
229. Rousham EK, Nahar P, Uddin MR, Islam MA, Nizame FA, Khisa N, et al. Gender and urban-rural influences on antibiotic purchasing and prescription use in retail drug shops: a one health study. BMC Public Health. 2023;23(1):229.
230. Al Masud A, Walpola RL, Sarker M, Kabir A, Asaduzzaman M, Islam MS, et al. Understanding antibiotic purchasing practices in community pharmacies: A potential driver of emerging antimicrobial resistance. Explor Res Clin Soc Pharm. 2024;15:100485.
231. Tenzin J, Tshomo KP, Wangda S, Gyeltshen W, Tshering G. Knowledge, attitude and practice on antimicrobial use and antimicrobial resistance among competent persons in the community pharmacies in Bhutan. Front Public Health. 2023;11:1113239.
232. Chandran DS, Manickavasagam PP. Sale of antibiotics without prescription in stand-alone pharmacies in Tamil Nadu. J Family Med Prim Care. 2022;11(9):5516-20.
233. Kumar KS, Saranya S, Rani NV. Community pharmacists' knowledge, attitude, and nonprescription dispensing practices of antibiotics: An explorative study in a selected city of South India. Journal of Research in Pharmacy Practice. 2022;11(2):51-8.
234. Dharanindra M, Shriram Dhanasekaran K, Rayana S, Noor SM, Bandela P, Viswanadh RPS, et al. Antibiotic-Dispensing Patterns and Awareness of Anti-microbial Resistance Among the Community Pharmacists in South-Central India. Cureus. 2023;15(10):e47043.
235. Ferdiana A, Liverani M, Khan M, Wulandari LPL, Mashuri YA, Batura N, et al. Community pharmacies, drug stores, and antibiotic dispensing in Indonesia: a qualitative study. BMC Public Health. 2021;21(1):1800.
236. Wulandari LPL, Khan M, Liverani M, Ferdiana A, Mashuri YA, Probandari A, et al. Prevalence and determinants of inappropriate antibiotic dispensing at private drug retail outlets in urban and rural areas of Indonesia: a mixed methods study. BMJ Glob Health. 2021;6(8).
237. Zawahir S, Lekamwasam S, Aslani P. A cross-sectional national survey of community pharmacy staff: Knowledge and antibiotic provision. PLoS One. 2019;14(4):e0215484.
238. Zawahir S, Lekamwasam S, Aslani P. Factors Related to Antibiotic Supply without a Prescription for Common Infections: A Cross-Sectional National Survey in Sri Lanka. Antibiotics. 2021;10(6).
239. Suy S, Rego S, Bory S, Chhorn S, Phou S, Prien C, et al. Invisible medicine sellers and their use of antibiotics: a qualitative study in Cambodia. BMJ Glob Health. 2019;4(5):e001787.
240. Thi Quynh Nhi L, de Alwis R, Khanh Lam P, Nhon Hoa N, Minh Nhan N, Thi Tu Oanh L, et al. Quantifying antimicrobial access and usage for paediatric diarrhoeal disease in an urban community setting in Asia. J Antimicrob Chemother. 2018;73(9):2546-54.
241. Nguyen HH, Ho DP, Vu TLH, Tran KT, Tran TD, Nguyen TKC, et al. "I can make more from selling medicine when breaking the rules" - understanding the antibiotic supply network in a rural community in Viet Nam. BMC Public Health. 2019;19(1):1560.

242. Nguyen NV, Do NTT, Nguyen CTK, Tran TK, Ho PD, Nguyen HH, et al. Community-level consumption of antibiotics according to the AWaRe (Access, Watch, Reserve) classification in rural Vietnam. JAC Antimicrob Resist. 2020;2(3):dlaa048.
243. Hayat K, Li P, Rosenthal M, Xu S, Chang J, Gillani AH, et al. Perspective of community pharmacists about community-based antimicrobial stewardship programs. A multicenter cross-sectional study from China. Expert Rev Anti Infect Ther. 2019;17(12):1043-50.
244. Feng Z, Hayat K, Huang Z, Shi L, Li P, Xiang C, et al. Knowledge, attitude, and practices of community pharmacy staff toward antimicrobial stewardship programs: a cross-sectional study from Northeastern China. Expert Rev Anti Infect Ther. 2021;19(4):529-36.
245. Zhang T, Lambert H, Zhao L, Liu R, Shen X, Wang D, et al. Antibiotic Stewardship in Retail Pharmacies and the Access-Excess Challenge in China: A Policy Review. Antibiotics. 2022;11(2).
246. Xu R, Mu T, Wang G, Shi J, Wang X, Ni X. Self-Medication with Antibiotics among University Students in LMIC: A systematic review and meta-analysis. J Infect Dev Ctries. 2019;13(8):678-89.
247. Seid MA, Hussen MS. Knowledge and attitude towards antimicrobial resistance among final year undergraduate paramedical students at University of Gondar, Ethiopia. BMC Infect Dis. 2018;18(1):312.
248. Fetensa G, Wakuma B, Tolossa T, Fekadu G, Bekuma TT, Fayisa L, et al. Knowledge and Attitude Towards Antimicrobial Resistance of Graduating Health Science Students of Wollega University. Infect Drug Resist. 2020;13:3937-44.
249. Chen J, Sidibi AM, Shen X, Dao K, Maiga A, Xie Y, et al. Lack of antibiotic knowledge and misuse of antibiotics by medical students in Mali: a cross-sectional study. Expert Rev Anti Infect Ther. 2021;19(6):797-804.
250. Kamoto A, Chapotera G, Suleman F. Knowledge, attitude and perception on antimicrobial use and antimicrobial resistance among final year medical students in the College of Medicine, Malawi. Malawi Med J. 2020;32(3):120-3.
251. Tuyishimire J, Okoya F, Adebayo AY, Humura F, Lucero-Prisno Iii DE. Assessment of self-medication practices with antibiotics among undergraduate university students in Rwanda. Pan Afr Med J. 2019;33:307.
252. Nisabwe L, Brice H, Umuhire MC, Gwira O, Harelimana JD, Nzeyimana Z, et al. Knowledge and attitudes towards antibiotic use and resistance among undergraduate healthcare students at University of Rwanda. J Pharm Policy Pract. 2020;13:7.
253. Chuwa BB, Njau LA, Msigwa KI, Shao E. Prevalence and factors associated with self medication with antibiotics among University students in Moshi Kilimanjaro Tanzania. Afr Health Sci. 2021;21(2):633-9.
254. Lubwama M, Onyuka J, Ayazika KT, Ssetaba LJ, Siboko J, Daniel O, et al. Knowledge, attitudes, and perceptions about antibiotic use and antimicrobial resistance among final year undergraduate medical and pharmacy students at three universities in East Africa. PLoS One. 2021;16(5):e0251301.
255. Shitindi L, Issa O, Poyongo BP, Horumpende PG, Kagashe GA, Sangeda RZ. Comparison of knowledge, attitude, practice and predictors of self-medication with antibiotics among medical and non-medical students in Tanzania. Front Pharmacol. 2023;14:1301561.
256. Kanyike AM, Olum R, Kajjimu J, Owembabazi S, Ojilong D, Nassozi DR, et al. Antimicrobial resistance and rational use of medicine: knowledge, perceptions, and training of clinical health professions students in Uganda. Antimicrob Resist Infect Control. 2022;11(1):145.
257. Kasujja H, Kajumbula H, Tusiimire J, Waswa JP, Nanyonga SM, Kiggundu R, et al. Engaging healthcare students in innovative approaches for antimicrobial resistance containment. 2024. 2024;15(1).
258. Nakato G, Adongo PR, Iramiot JS, Epuitai J. Practices and drivers of self-medication with antibiotics among undergraduate medical students in Eastern Uganda: A cross-sectional study. PLoS One. 2023;18(12):e0293685.
259. Zulu AC, Matafwali S, Banda M, Mudenda S. Assessment of knowledge, attitude and practices on antibiotic resistance among undergraduate medical students in the school of medicine at the University of Zambia. International Journal of Basic & Clinical Pharmacology. 2020;9:263.
260. Mudenda S, Chisha P, Chabalenge B, Daka V, Mfune RL, Kasanga M, et al. Antimicrobial stewardship: knowledge, attitudes and practices regarding antimicrobial use and resistance among non-healthcare students at the University of Zambia. JAC Antimicrob Resist. 2023;5(6):dlad116.
261. Nowbuth AA, Monteiro FJ, Sheets LR, Asombang AW. Assessment of the knowledge, attitudes and perceived quality of education about antimicrobial use and resistance of medical students in Zambia, Southern Africa. JAC Antimicrob Resist. 2023;5(3):dlad073.
262. Owusu-Ofori AK, Darko E, Danquah CA, Agyarko-Poku T, Buabeng KO. Self-Medication and Antimicrobial Resistance: A Survey of Students Studying Healthcare Programmes at a Tertiary Institution in Ghana. Front Public Health. 2021;9:706290.

263. Amponsah SK, Odamtten G, Adams I, Kretchy IA. A comparative analysis of pattern and attitude towards self-medication among pharmacy and non-pharmacy students in University of Ghana. Pan Afr Med J. 2022;41:254.
264. Sefah IA, Akwaboah E, Sarkodie E, Godman B, Meyer JC. Evaluation of Healthcare Students' Knowledge on Antibiotic Use, Antimicrobial Resistance and Antimicrobial Stewardship Programs and Associated Factors in a Tertiary University in Ghana: Findings and Implications. Antibiotics. 2022;11(12):1679.
265. Ajibola O, Omisakin OA, Eze AA, Omoleke SA. Self-Medication with Antibiotics, Attitude and Knowledge of Antibiotic Resistance among Community Residents and Undergraduate Students in Northwest Nigeria. Diseases. 2018;6(2).
266. Ayepola OO, Onile-Ere OA, Shodeko OE, Akinsiku FA, Ani PE, Egwari L. Dataset on the knowledge, attitudes and practices of university students towards antibiotics. Data Brief. 2018;19:2084-94.
267. Okedo-Alex I, Madubueze UC, Umeokonkwo CD, Oka OU, Adeke AS, Okeke KC. Knowledge of antibiotic use and resistance among students of a medical school in Nigeria. Malawi Med J. 2019;31(2):133-7.
268. Augie BM, van Zyl RL, McInerney PA, Miot J. Knowledge and perceptions about antibiotic resistance and prudent antibiotic prescribing among final year medical students in two African countries. Int J Pharm Pract. 2021;29(5):508-14.
269. Akande-Sholabi W, Ajamu AT. Antimicrobial stewardship: Assessment of knowledge, awareness of antimicrobial resistance and appropriate antibiotic use among healthcare students in a Nigerian University. BMC Med Educ. 2021;21(1):488.
270. Akande-Sholabi W, Ajamu AT, Adisa R. Prevalence, knowledge and perception of self-medication practice among undergraduate healthcare students. J Pharm Policy Pract. 2021;14(1):49.
271. Abdu-Aguye SN, Barde KG, Yusuf H, Lawal BK, Shehu A, Mohammed E. Investigating Knowledge of Antibiotics, Antimicrobial Resistance and Antimicrobial Stewardship Concepts Among Final Year Undergraduate Pharmacy Students in Northern Nigeria. Integr Pharm Res Pract. 2022;11:187-95.
272. Teague E, Bezuidenhout S, Meyer JC, Godman B, Engler D. Knowledge and Perceptions of Final-Year Nursing Students Regarding Antimicrobials, Antimicrobial Resistance, and Antimicrobial Stewardship in South Africa: Findings and Implications to Reduce Resistance. Antibiotics. 2023;12(12):1742.
273. Assar A, Abdelraoof MI, Abdel-Maboud M, Shaker KH, Menshawy A, Swelam AH, et al. Knowledge, attitudes, and practices of Egypt's future physicians towards antimicrobial resistance (KAP-AMR study): a multicenter cross-sectional study. Environ Sci Pollut Res Int. 2020;27(17):21292-8.
274. Mostafa A, Abdelzaher A, Rashed S, AlKhawaga SI, Afifi SK, AbdelAlim S, et al. Is health literacy associated with antibiotic use, knowledge and awareness of antimicrobial resistance among non-medical university students in Egypt? A cross-sectional study. BMJ Open. 2021;11(3):e046453.
275. Alsayed AR, Darwish El Hajji F, Al-Najjar MAA, Abazid H, Al-Dulaimi A. Patterns of antibiotic use, knowledge, and perceptions among different population categories: A comprehensive study based in Arabic countries. Saudi Pharm J. 2022;30(3):317-28.
276. Naser AY, Aboutaleb R, Khaleel A, Alsairafi ZK, Alwafi H, Qadus S, et al. Knowledge, attitude, and practices of pharmacy students in 7 Middle Eastern countries concerning antibiotic resistance: A cross-sectional study. Medicine. 2024;103(36):e39378.
277. Abubakar U, Muhammad HT, Sulaiman SAS, Ramatillah DL, Amir O. Knowledge and self-confidence of antibiotic resistance, appropriate antibiotic therapy, and antibiotic stewardship among pharmacy undergraduate students in three Asian countries. Curr Pharm Teach Learn. 2020;12(3):265-73.
278. Hussain I, Yousaf N, Haider S, Jalil P, Saleem MU, Imran I, et al. Assessing Knowledge and Perception Regarding Antimicrobial Stewardship and Antimicrobial Resistance in University Students of Pakistan: Findings and Implications. Antibiotics. 2021;10(7).
279. Hayat K, Jamshed S, Rosenthal M, Haq NU, Chang J, Rasool MF, et al. Understanding of Pharmacy Students towards Antibiotic Use, Antibiotic Resistance and Antibiotic Stewardship Programs: A Cross-Sectional Study from Punjab, Pakistan. Antibiotics. 2021;10(1).
280. Khan FU, Khan A, Shah S, Hayat K, Usman A, Khan FU, et al. Exploring Undergraduate Pharmacy Students Perspectives Towards Antibiotics Use, Antibiotic Resistance, and Antibiotic Stewardship Programs Along With the Pharmacy Teachers' Perspectives: A Mixed-Methods Study From Pakistan. Front Pharmacol. 2021;12:754000.
281. Shah S, Abbas G, Chauhdary Z, Aslam A, Rehman AU, Khurram H, et al. Antibiotic use: A cross-sectional survey assessing the knowledge, attitudes, and practices amongst students of Punjab, Pakistan. J Am Coll Health. 2022;70(8):2499-504.

282. Yasmin F, Asghar MS, Naeem U, Najeeb H, Nauman H, Ahsan MN, et al. Self-Medication Practices in Medical Students During the COVID-19 Pandemic: A Cross-Sectional Analysis. Front Public Health. 2022;10:803937.
283. Ahmed S, Tareq AH, Ilyas D. The Impact of Antimicrobial Resistance and Stewardship Training Sessions on Knowledge of Healthcare Students of Wah Cantonment, Pakistan. Inquiry. 2024;61:469580241228443.
284. Abuawad M, Ziyadeh-Isleem A, Mahamid A, Quzmar S, Ammar E, Shawahna R. Knowledge, perception, and attitudes of medical students towards antimicrobial resistance and stewardship: an observational cross-sectional study from Palestine. BMC Med Educ. 2024;24(1):302.
285. Elmahi OKO, Musa RAE, Shareef AAH, Omer MEA, Elmahi MAM, Altamih RAA, et al. Perception and practice of self-medication with antibiotics among medical students in Sudanese universities: A cross-sectional study. PLoS One. 2022;17(1):e0263067.
286. Abdelkarim OA, Abubakar U, Hussain MA, Abadi AEB, Mohamed AO, Osman W, et al. Knowledge, Perception, and Self-Confidence of Antibiotic Resistance, Appropriate Antibiotic Therapy, and Antibiotic Stewardship Among Undergraduate Pharmacy Students in Sudan. Infect Drug Resist. 2024;17:935-49.
287. Abdi A, Faraji A, Dehghan F, Khatony A. Prevalence of self-medication practice among health sciences students in Kermanshah, Iran. BMC Pharmacol Toxicol. 2018;19(1):36.
288. Najjar Y, Hassan Z. Self-Treatment with Antibiotics: Knowledge level, Prevalence and Indications for Practicing Among University Students in Jordan. Curr Drug Saf. 2021;16(1):82-9.
289. Al-Qerem W, Hammad A, Jarab A, M MS, Amawi HA, Ling J, et al. Knowledge, attitudes, and practice with respect to antibiotic use among pharmacy students: a cross-sectional study. Eur Rev Med Pharmacol Sci. 2022;26(10):3408-18.
290. Al-Taani GM, Karasneh RA, Al-Azzam S, Bin Shaman M, Jirjees F, Al-Obaidi H, et al. Knowledge, Attitude, and Behavior about Antimicrobial Use and Resistance among Medical, Nursing and Pharmacy Students in Jordan: A Cross Sectional Study. Antibiotics. 2022;11(11).
291. Sakr S, Ghaddar A, Hamam B, Sheet I. Antibiotic use and resistance: an unprecedented assessment of university students' knowledge, attitude and practices (KAP) in Lebanon. BMC Public Health. 2020;20(1):535.
292. Atallah S, Mansour H, Dimassi H, Kabbara WK. Impact of social media education on antimicrobial stewardship awareness among pharmacy, medical and nursing students and residents. BMC Med Educ. 2023;23(1):446.
293. Shah P, Shrestha R, Mao Z, Chen Y, Chen Y, Koju P, et al. Knowledge, Attitude, and Practice Associated with Antibiotic Use among University Students: A Survey in Nepal. Int J Environ Res Public Health. 2019;16(20).
294. Shrestha R. Knowledge, Attitude and Practice on Antibiotics Use and its Resistance Among Medical Students in A Tertiary Care Hospital. JNMA J Nepal Med Assoc. 2019;57(216):74-9.
295. Mandal NK, Rauniyar GP, Rai DS, Panday DR, Kushwaha R, Agrawal SK, et al. Self-medication Practice of Antibiotics among Medical and Dental Undergraduate Students in a Medical College in Eastern Nepal: A Descriptive Cross-sectional Study. J Nepal Med Assoc. 2020;58(225):328-32.
296. Shah K, Halder S, Haider SS. Assessment of knowledge, perception, and awareness about self-medication practices among university students in Nepal. Heliyon. 2021;7(1):e05976.
297. Seam MOR, Bhatta R, Saha BL, Das A, Hossain MM, Uddin SMN, et al. Assessing the Perceptions and Practice of Self-Medication among Bangladeshi Undergraduate Pharmacy Students. Pharmacy. 2018;6(1).
298. Marzan M, Islam DZ, Lugova H, Krishnapillai A, Haque M, Islam S. Knowledge, Attitudes, and Practices of Antimicrobial Uses and Resistance Among Public University Students in Bangladesh. Infect Drug Resist. 2021;14:519-33.
299. Paul GK, Swapon MS, Kaderi Kibria KM. Knowledge, awareness, and attitudes toward antibiotic resistance and practice of self-medication among university students in Bangladesh: A cross-sectional study. J Educ Health Promot. 2022;11:115.
300. Wahab A, Alam MM, Hasan S, Halder S, Ullah MO, Hossain A. Exploring the knowledge, practices & determinants of antibiotic self-medication among bangladeshi university students in the era of COVID-19: A cross-sectional study. Heliyon. 2023;9(9):e19923.
301. Gupta MK, Vohra C, Raghav P. Assessment of knowledge, attitudes, and practices about antibiotic resistance among medical students in India. Journal of family medicine and primary care. 2019;8(9):2864-9.
302. Ritchie O, Shetty V, Prabhu S, Shetty AK. Confidence in antibiotic prescribing intentions among senior medical students in India. The American Journal of Tropical Medicine and Hygiene. 2020;103(6):2561.

303. Agrawal A, Chauhan CS, Boliwal K, Sharma A. Acquaintance and Awareness of Budding Physicians toward Antimicrobials' Use: Need of the Hour. Journal of Laboratory Physicians. 2022;14(02):202-9.
304. Nabi N, Baluja Z, Mukherjee S, Kohli S. Trends in Practices of Self-Medication with Antibiotics among Medical Undergraduates in India. J Pharm Bioallied Sci. 2022;14(1):19-24.
305. Sakeena MH, Bennett AA, Mohamed F, Herath HM, Gawarammane I, McLachlan AJ. Investigating knowledge regarding antibiotics among pharmacy and allied health sciences students in a Sri Lankan university. J Infect Dev Ctries. 2018;12(9):726-32.
306. Sakeena MHF, Bennett AA, Jamshed S, Mohamed F, Herath DR, Gawarammana I, et al. Investigating knowledge regarding antibiotics and antimicrobial resistance among pharmacy students in Sri Lankan universities. BMC Infect Dis. 2018;18(1):209.
307. Jayaweerasingham M, Angulmaduwa S, Liyanapathirana V. Knowledge, beliefs and practices on antibiotic use and resistance among a group of trainee nurses in Sri Lanka. BMC Res Notes. 2019;12(1):601.
308. Sakeena MHF, Bennett AA, Carter SJ, McLachlan AJ. A comparative study regarding antibiotic consumption and knowledge of antimicrobial resistance among pharmacy students in Australia and Sri Lanka. PLoS One. 2019;14(3):e0213520.
309. Hu Y, Wang X, Tucker JD, Little P, Moore M, Fukuda K, et al. Knowledge, Attitude, and Practice with Respect to Antibiotic Use among Chinese Medical Students: A Multicentre Cross-Sectional Study. Int J Environ Res Public Health. 2018;15(6).
310. Peng D, Wang X, Xu Y, Sun C, Zhou X. Antibiotic misuse among university students in developed and less developed regions of China: a cross-sectional survey. Glob Health Action. 2018;11(1):1496973.
311. Lin L, Fearon E, Harbarth S, Wang X, Lu C, Zhou X, et al. Decisions to use antibiotics for upper respiratory tract infections across China: a large-scale cross-sectional survey among university students. BMJ Open. 2020;10(8):e039332.
312. Wang Y, Guo F, Wei J, Zhang Y, Liu Z, Huang Y. Knowledge, attitudes and practices in relation to antimicrobial resistance amongst Chinese public health undergraduates. J Glob Antimicrob Resist. 2020;23:9-15.
313. Min S, Zhou Y, Sun Y, Ye J, Dong Y, Wang X, et al. Knowledge, attitude, and practice associated with antimicrobial resistance among medical students between 2017 and 2022: A survey in East China. Front Public Health. 2022;10:1010582.
314. Yang C, Xie J, Chen Q, Yuan Q, Shang J, Wu H, et al. Knowledge, Attitude, and Practice About Antibiotic Use and Antimicrobial Resistance Among Nursing Students in China: A Cross Sectional Study. Infect Drug Resist. 2024;17:1085-98.
315. Haque M, Rahman NAA, McKimm J, Binti Abdullah SL, Islam MZ, Zulkifli Z, et al. A cross-sectional study evaluating the knowledge and beliefs about, and the use of antibiotics amongst Malaysian university students. Expert Rev Anti Infect Ther. 2019;17(4):275-84.
316. Haque M, Rahman NAA, McKimm J, Sartelli M, Kibria GM, Islam MZ, et al. Antibiotic Use: A Cross-Sectional Study Evaluating the Understanding, Usage and Perspectives of Medical Students and Pathfinders of a Public Defence University in Malaysia. Antibiotics. 2019;8(3).
317. Haque M, Ara T, Haq MA, Lugova H, Dutta S, Samad N, et al. Antimicrobial Prescribing Confidence and Knowledge Regarding Drug Resistance: Perception of Medical Students in Malaysia and the Implications. Antibiotics. 2022;11(5):540.
318. Sariola S, Butcher A, Cañada JA, Aïkpé M, Compaore A. Closing the GAP in Antimicrobial Resistance Policy in Benin and Burkina Faso. mSystems. 2022;7(4):e0015022.
319. Shembo AKP, Musumari PM, Srithanaviboonchai K, Tangmunkongvorakul A, Dalleur O. A qualitative study on community use of antibiotics in Kinshasa, Democratic Republic of Congo. PLoS One. 2022;17(4):e0267544.
320. Ateshim Y, Bereket B, Major F, Emun Y, Woldai B, Pasha I, et al. Prevalence of self-medication with antibiotics and associated factors in the community of Asmara, Eritrea: a descriptive cross sectional survey. BMC Public Health. 2019;19(1):726.
321. Russom M, Bahta M, Debesai M, Bahta I, Kessete A, Afendi A, et al. Knowledge, attitude and practice of antibiotics and their determinants in Eritrea: an urban population-based survey. BMJ Open. 2021;11(9):e046432.
322. Bogale AA, Amhare AF, Chang J, Bogale HA, Betaw ST, Gebrehiwot NT, et al. Knowledge, attitude, and practice of self-medication with antibiotics among community residents in Addis Ababa, Ethiopia. Expert Rev Anti Infect Ther. 2019;17(6):459-66.

323. Mengesha Y, Manaye B, Moges G. Assessment of Public Awareness, Attitude, and Practice Regarding Antibiotic Resistance in Kemissie Town, Northeast Ethiopia: Community-Based Cross-Sectional Study. Infect Drug Resist. 2020;13:3783-9.
324. Dejene H, Birhanu R, Tarekegn ZS. Knowledge, attitude and practices of residents toward antimicrobial usage and resistance in Gondar, Northwest Ethiopia. One Health Outlook. 2022;4(1):10.
325. Demissie F, Ereso K, Paulos G. Self-Medication Practice with Antibiotics and Its Associated Factors Among Community of Bule-Hora Town, South West Ethiopia. Drug Healthc Patient Saf. 2022;14:9-18.
326. Simegn W, Moges G. Awareness and knowledge of antimicrobial resistance and factors associated with knowledge among adults in Dessie City, Northeast Ethiopia: Community-based cross-sectional study. PLoS One. 2022;17(12):e0279342.
327. Jifar WW, Oumer OM, Muhammed, II, BaHammam AS. Assessment of factors associated with self-medication practices during the COVID-19 pandemic in southwestern Ethiopia: a community-based cross-sectional survey. BMC Infect Dis. 2024;24(1):925.
328. Muhummed AM, Alemu A, Maidane YO, Tschopp R, Hattendorf J, Vonaesch P, et al. Knowledge, Attitudes, and Practices of Rural Communities Regarding Antimicrobial Resistance and Climate Change in Adadle District, Somali Region, Ethiopia: A Mixed-Methods Study. Antibiotics. 2024;13(4).
329. Sambakunsi CS, Småbrekke L, Varga CA, Solomon V, Mponda JS. Knowledge, attitudes and practices related to self-medication with antimicrobials in Lilongwe, Malawi. Malawi Med J. 2019;31(4):225-32.
330. Machongo RB, Mipando ALN. "I don't hesitate to use the left-over antibiotics for my child" practices and experiences with antibiotic use among caregivers of paediatric patients at Zomba central hospital in Malawi. BMC Pediatr. 2022;22(1):466.
331. Limwado GD, Aron MB, Mpinga K, Phiri H, Chibvunde S, Banda C, et al. Prevalence of antibiotic self-medication and knowledge of antimicrobial resistance among community members in Neno District rural Malawi: A cross-sectional study. IJID Reg. 2024;13:100444.
332. Mate I, Come CE, Gonçalves MP, Cliff J, Gudo ES. Knowledge, attitudes and practices regarding antibiotic use in Maputo City, Mozambique. PLoS One. 2019;14(8):e0221452.
333. Cambaco O, Alonso Menendez Y, Kinsman J, Sigaúque B, Wertheim H, Do N, et al. Community knowledge and practices regarding antibiotic use in rural Mozambique: where is the starting point for prevention of antibiotic resistance? BMC Public Health. 2020;20(1):1183.
334. Lanyero H, Ocan M, Obua C, Stålsby Lundborg C, Agaba K, Kalyango JN, et al. Validity of caregivers' reports on prior use of antibacterials in children under five years presenting to health facilities in Gulu, northern Uganda. PLoS One. 2021;16(9):e0257328.
335. Nyeko R, Otim F, Obiya EM, Abala C. Pre-hospital exposures to antibiotics among children presenting with fever in northern Uganda: a facility-based cross-sectional study. BMC Pediatr. 2022;22(1):322.
336. Wildbret S, Stuck L, Luchen CC, Simuyandi M, Chisenga C, Schultsz C, et al. Drivers of informal sector and non-prescription medication use in pediatric populations in a low- and middle-income setting: A prospective cohort study in Zambia. PLOS Glob Public Health. 2023;3(7):e0002072.
337. Mudenda S, Simukoko N, Mohamed S. Knowledge, attitude and practices regarding antimicrobial use and resistance among community members of Mtendere Township in Lusaka, Zambia: findings and implications on antimicrobial stewardship. Int J Basic Clin Pharmacol. 2024;13(3):315-321
338. Kampamba M, Hamaambo B, Hikaambo CN, Mwanza B, Bambala A, Mutenda M, et al. Evaluation of knowledge and practices on antibiotic use: a cross-sectional study on self-reported adherence to short-term antibiotic utilization among patients visiting level-1 hospitals in Lusaka, Zambia. JAC Antimicrob Resist. 2024;6(4):dlae120.
339. Ngoma MT, Sitali D, Mudenda S, Mukuma M, Bumbangi FN, Bunuma E, et al. Community antibiotic consumption and associated factors in Lusaka district of Zambia: findings and implications for antimicrobial resistance and stewardship. JAC Antimicrob Resist. 2024;6(2):dlae034.
340. Aika IN, Enato E. Bridging the gap in knowledge and use of antibiotics among pediatric caregivers: comparing two educational interventions. J Pharm Policy Pract. 2023;16(1):76.
341. Ngu RC, Feteh VF, Kika BT, F EKN, Ayeah CM, Chifor T, et al. Prevalence and Determinants of Antibiotic Self-Medication among Adult Patients with Respiratory Tract Infections in the Mboppi Baptist Hospital, Douala, Cameroon: A Cross-Sectional Study. Diseases. 2018;6(2).
342. Penda CI, Moukoko ECE, Youmba JFN, Mpondo EM. Characterization of pharmaceutical medication without a medical prescription in children before hospitalization in a resource-limited setting, Cameroon. Pan Afr Med J. 2018;30:302.

343. Elong Ekambi GA, Okalla Ebongue C, Penda IC, Nnanga Nga E, Mpondo Mpondo E, Eboumbou Moukoko CE. Knowledge, practices and attitudes on antibiotics use in Cameroon: Self-medication and prescription survey among children, adolescents and adults in private pharmacies. PLoS One. 2019;14(2):e0212875.
344. Ahiabu MA, Magnussen P, Bygbjerg IC, Tersbøl BP. Treatment practices of households and antibiotic dispensing in medicine outlets in developing countries: The case of Ghana. Res Social Adm Pharm. 2018;14(12):1180-8.
345. Mensah BN, Agyemang IB, Afriyie DK, Amponsah SK. Self-medication practice in Akuse, a rural setting in Ghana. Niger Postgrad Med J. 2019;26(3):189-94.
346. Afari-Asiedu S, Oppong FB, Tostmann A, Ali Abdulai M, Boamah-Kaali E, Gyaase S, et al. Determinants of Inappropriate Antibiotics Use in Rural Central Ghana Using a Mixed Methods Approach. Front Public Health. 2020;8:90.
347. Afari-Asiedu S, Hulscher M, Abdulai MA, Boamah-Kaali E, Asante KP, Wertheim HFL. Every medicine is medicine; exploring inappropriate antibiotic use at the community level in rural Ghana. BMC Public Health. 2020;20(1):1103.
348. Effah CY, Amoah AN, Liu H, Agboyibor C, Miao L, Wang J, et al. A population-base survey on knowledge, attitude and awareness of the general public on antibiotic use and resistance. Antimicrob Resist Infect Control. 2020;9(1):105.
349. Jimah T, Fenny AP, Ogunseitan OA. Antibiotics stewardship in Ghana: a cross-sectional study of public knowledge, attitudes, and practices among communities. One Health Outlook. 2020;2:12.
350. Kretchy J-P, Adase SK, Gyansa-Lutterodt M. The prevalence and risks of antibiotic self-medication in residents of a rural community in Accra, Ghana. Scientific African. 2021;14:e01006.
351. Otieku E, Fenny AP, Labi AK, Owusu-Ofori AK, Kurtzhals J, Enemark U. Knowledge, attitudes and practices regarding antimicrobial use and resistance among healthcare seekers in two tertiary hospitals in Ghana: a quasi-experimental study. BMJ Open. 2023;13(2):e065233.
352. Vicar EK, Walana W, Mbabila A, Darko GK, Opare-Asamoah K, Majeed SF, et al. Drivers of household antibiotic use in urban informal settlements in Northern Ghana: Implications for antimicrobial resistance control. Health Sci Rep. 2023;6(7):e1388.
353. Hackman HK, Annison L, Arhin RE, Adjei GO, Otu P, Arthur-Hayford E, et al. Self-medication with antibiotics during the COVID-19 pandemic: A cross-sectional study among adults in Tema, Ghana. PLoS One. 2024;19(6):e0305602.
354. Badger-Emeka LI, Emeka PM, Okosi M. Evaluation of the extent and reasons for increased non-prescription antibiotics use in a University town, Nsukka Nigeria. Int J Health Sci. 2018;12(4):11-7.
355. Chukwu EE, Oladele DA, Awoderu OB, Afocha EE, Lawal RG, Abdus-Salam I, et al. A national survey of public awareness of antimicrobial resistance in Nigeria. Antimicrob Resist Infect Control. 2020;9(1):72.
356. Akande-Sholabi W, Oyesiji E. Antimicrobial stewardship: knowledge, perceptions, and factors associated with antibiotics misuse among consumer's visiting the community pharmacies in a Nigeria Southwestern State. J Pharm Policy Pract. 2023;16(1):120.
357. Idoko LO, Okafor KC, Lolo NS. KNOWLEDGE OF ANTIMICROBIAL DRUG RESISTANCE (AMR) AMONG PATIENTS IN A GENERAL OUTPATIENT CLINIC IN ABUJA MUNICIPAL AREA COUNCIL (AMAC), FEDERAL CAPITAL TERRITORY, NIGERIA. West Afr J Med. 2023;40(11 Suppl 1):S13.
358. Isah A, Aina AB, Ben-Umeh KC, Onyekwum CA, Egbuemike CC, Ezechukwu CV, et al. Assessment of public knowledge and attitude toward antibiotics use and resistance: a community pharmacy-based survey. J Pharm Policy Pract. 2023;16(1):107.
359. Bassoum O, Sougou NM, Diongue M, Lèye MMM, Mbodji M, Fall D, et al. Assessment of General Public's Knowledge and Opinions towards Antibiotic Use and Bacterial Resistance: A Cross-Sectional Study in an Urban Setting, Rufisque, Senegal. Pharmacy. 2018;6(4).
360. Horumpende PG, Said SH, Mazuguni FS, Antony ML, Kumburu HH, Sonda TB, et al. Prevalence, determinants and knowledge of antibacterial self-medication: A cross sectional study in North-eastern Tanzania. PLoS One. 2018;13(10):e0206623.
361. Mboya EA, Sanga LA, Ngocho JS. Irrational use of antibiotics in the Moshi Municipality Northern Tanzania: a cross sectional study. Pan Afr Med J. 2018;31:165.
362. Mboya EA, Davies ML, Horumpende PG, Ngocho JS. Inadequate knowledge on appropriate antibiotics use among clients in the Moshi municipality Northern Tanzania. PLoS One. 2020;15(9):e0239388.

363. Simon B, Kazaura M. Prevalence and Factors Associated with Parents Self-Medicating Under-Fives with Antibiotics in Bagamoyo District Council, Tanzania: a Cross-Sectional Study. Patient Prefer Adherence. 2020;14:1445-53.
364. Sindato C, Mboera LEG, Katale BZ, Frumence G, Kimera S, Clark TG, et al. Knowledge, attitudes and practices regarding antimicrobial use and resistance among communities of Ilala, Kilosa and Kibaha districts of Tanzania. Antimicrob Resist Infect Control. 2020;9(1):194.
365. Gabriel S, Manumbu L, Mkusa O, Kilonzi M, Marealle AI, Mutagonda RF, et al. Knowledge of use of antibiotics among consumers in Tanzania. JAC Antimicrob Resist. 2021;3(4):dlab183.
366. Mutagonda RF, Marealle AI, Nkinda L, Kibwana U, Maganda BA, Njiro BJ, et al. Determinants of misuse of antibiotics among parents of children attending clinics in regional referral hospitals in Tanzania. Sci Rep. 2022;12(1):4836.
367. Farley E, van den Bergh D, Coetzee R, Stewart A, Boyles T. Knowledge, attitudes and perceptions of antibiotic use and resistance among patients in South Africa: A cross-sectional study. S Afr J Infect Dis. 2019;34(1):118.
368. Mokoena TTW, Schellack N, Brink AJ. Driving antibiotic stewardship awareness through the minibus-taxi community across the Tshwane District, South Africa-a baseline evaluation. JAC Antimicrob Resist. 2021;3(3):dlab106.
369. Sono TM, Mboweni V, Jelić AG, Campbell SM, Marković-Peković V, Ramdas N, et al. Pilot Study to Evaluate Patients' Understanding of Key Terms and Aspects of Antimicrobial Use in a Rural Province in South Africa Findings and Implications. Advances in Human Biology. 2025;15(1):108-12.
370. Alshakka M, Hatem NA, Al-Abd N, Badullah W, Alawi S, Ibrahim MIM. Knowledge, attitude, and practice toward antibiotic use among the general public in a resource-poor setting: A case of Aden-Yemen. J Infect Dev Ctries. 2023;17(3):345-52.
371. Alshakka M, Badulla WFS, Bahattab AAS, Al-abd NM, Mahmoud MA, YasserAlahmadi, et al. Perception and practices of antimicrobial stewardship by communitypharmacists in Aden-Yemen. Biomedical Research-tokyo. 2019;30:819-25.
372. Burtscher D, Van den Bergh R, Nasim M, Mahama G, Au S, Williams A, et al. 'They eat it like sweets': A mixed methods study of antibiotic perceptions and their use among patients, prescribers and pharmacists in a district hospital in Kabul, Afghanistan. PLoS One. 2021;16(11):e0260096.
373. Maarouf L, Amin M, Evans BA, Abouelfetouh A. Knowledge, attitudes and behaviour of Egyptians towards antibiotic use in the community: can we do better? Antimicrob Resist Infect Control. 2023;12(1):50.
374. Ali HT, Barakat M, Abdelhalim AR, Al-Kurd IN, Muhammad MK, Sharkawy MM, et al. Unravelling the dilemma of self-medication in Egypt: a cross-sectional survey on knowledge, attitude, and practice of the general Egyptian population. BMC Public Health. 2024;24(1):652.
375. Elhaddadi H, Hamami A, Sara A, Elouali A, Babakhouya A, Rkain M. Prevalence and Determinants of the Use of Antibiotics by Self-Medication in the Pediatric Population in Oujda, Morocco. Cureus. 2024;16(5):e60126.
376. Akhund R, Jamshed F, Jaffry HA, Hanif H, Fareed S. Knowledge and Attitude of General Pakistani Population Towards Antibiotic Resistance. Cureus. 2019;11(3):e4266.
377. Atif M, Asghar S, Mushtaq I, Malik I, Amin A, Babar ZU, et al. What drives inappropriate use of antibiotics? A mixed methods study from Bahawalpur, Pakistan. Infect Drug Resist. 2019;12:687-99.
378. Khan FU, Khan FU, Hayat K, Chang J, Saeed A, Khan Z, et al. Knowledge, attitude and practices among consumers toward antibiotics use and antibiotic resistance in Swat, Khyber-Pakhtunkhwa, Pakistan. Expert Rev Anti Infect Ther. 2020;18(9):937-46.
379. Vaidya K, Aiemjoy K, Qamar FN, Saha SK, Tamrakar D, Naga SR, et al. Antibiotic Use Prior to Hospital Presentation Among Individuals With Suspected Enteric Fever in Nepal, Bangladesh, and Pakistan. Clin Infect Dis. 2020;71(Suppl 3):S285-s92.
380. Gillani AH, Chang J, Aslam F, Saeed A, Shukar S, Khanum F, et al. Public knowledge, attitude, and practice regarding antibiotics use in Punjab, Pakistan: a cross-sectional study. Expert Rev Anti Infect Ther. 2021;19(3):399-411.
381. Arshad H, Gillani AH, Akbar J, Abbas H, Bashir Ahmed A, Gillani SNH, et al. Knowledge on Multi-Drug Resistant Pathogens, Antibiotic Use and Self-Reported Adherence to Antibiotic Intake: A Population-Based Cross Sectional Survey From Pakistan. Frontiers in Pharmacology. 2022;13.
382. Khan FU, Mallhi TH, Khan FU, Hayat K, Rehman AU, Shah S, et al. Evaluation of Consumers Perspective on the Consumption of Antibiotics, Antibiotic Resistance, and Recommendations to Improve the

Rational use of Antibiotics: An Exploratory Qualitative Study From Post-Conflicted Region of Pakistan. Frontiers in Pharmacology. 2022;13.
383. Arifa S, Tahir Mehmood K, Allah B, Muhammad Osama Y, Alia S. Cross-sectional survey to explore knowledge, attitude, practices and impact of an intervention programme related to antibiotic misuse and self-medication among general population of Pakistan. BMJ Public Health. 2024;2(1):e000758.
384. Hussain MA, Mohamed AO, Abdelkarim OA, Yousef BA, Babikir AA, Mirghani MM, et al. Prevalence and Predictors of Antibiotic Self-Medication in Sudan: A Descriptive Cross-Sectional Study. Antibiotics. 2023;12(3):612.
385. Ben Mabrouk A, Larbi Ammari F, Werdani A, Jemmali N, Chelli J, Mrabet HE, et al. Parental self-medication with antibiotics in a Tunisian pediatric center. Therapie. 2022;77(4):477-85.
386. Nazari J, Chezani-Sharahi N, Eshrati B, Yadegari A, Naghshbandi M, Movahedi H, et al. Prevalence and determinants of self-medication consumption of antibiotics in children in Iran: A population-based cross-sectional study, 2018-19. PLoS One. 2022;17(12):e0278843.
387. Al-Taie A, Hussein AN, Albasry Z. A Cross-Sectional Study of Patients' Practices, Knowledge and Attitudes of Antibiotics among Iraqi Population. J Infect Dev Ctries. 2021;15(12):1845-53.
388. Muhammed R, Al-Ani A, Yassen A. Self-Medication with Antibiotic among Public Population in Erbil City. Journal of Pharmaceutical Research International. 2021;33:25-33.
389. Al-Yasseri BJH, Hussain NA. Public Knowledge and Attitudes Towards Antibiotics Use and Resistance in Baghdad, Iraq: A Survey Conducted in Outpatient Department of University Teaching Hospital. The Open Public Health Journal. 2019;12:567-74.
390. Hammour KA, Jalil MA, Hammour WA. An exploration of parents' knowledge, attitudes and practices towards the use of antibiotics in childhood upper respiratory tract infections in a tertiary Jordanian Hospital. Saudi Pharm J. 2018;26(6):780-5.
391. Yusef D, Babaa AI, Bashaireh AZ, Al-Bawayeh HH, Al-Rijjal K, Nedal M, et al. Knowledge, practices & attitude toward antibiotics use and bacterial resistance in Jordan: A cross-sectional study. Infect Dis Health. 2018;23(1):33-40.
392. Abdel-Qader DH, Albassam A, Ismael NS, El-Shara AA, Shehri A, Almutairi FS, et al. Awareness of Antibiotic Use and Resistance in Jordanian Community. J Prim Care Community Health. 2020;11:2150132720961255.
393. Abdelmalek S, AlEjielat R, Rayyan WA, Qinna N, Darwish D. Changes in public knowledge and perceptions about antibiotic use and resistance in Jordan: a cross-sectional eight-year comparative study. BMC Public Health. 2021;21(1):750.
394. Muflih SM, Al-Azzam S, Karasneh RA, Bleidt BA, Conway BR, Bond SE, et al. Public knowledge of antibiotics, self-medication, and household disposal practices in Jordan. Expert Rev Anti Infect Ther. 2023;21(4):477-87.
395. El Khoury G, Ramia E, Salameh P. Misconceptions and Malpractices Toward Antibiotic Use in Childhood Upper Respiratory Tract Infections Among a Cohort of Lebanese Parents. Eval Health Prof. 2018;41(4):493-511.
396. Mallah N, Badro DA, Figueiras A, Takkouche B. Association of knowledge and beliefs with the misuse of antibiotics in parents: A study in Beirut (Lebanon). PLoS One. 2020;15(7):e0232464.
397. Henaine AM, Lahoud N, Abdo R, Shdeed R, Safwan J, Akel M, et al. Knowledge of Antibiotics Use among Lebanese Adults: A study on the influence of sociodemographic characteristics. Sultan Qaboos Univ Med J. 2021;21(3):442-9.
398. Nepal A, Hendrie D, Robinson S, Selvey LA. Knowledge, attitudes and practices relating to antibiotic use among community members of the Rupandehi District in Nepal. BMC Public Health. 2019;19(1):1558.
399. Chowdhury M, Stewart Williams J, Wertheim H, Khan WA, Matin A, Kinsman J. Rural community perceptions of antibiotic access and understanding of antimicrobial resistance: qualitative evidence from the Health and Demographic Surveillance System site in Matlab, Bangladesh. Glob Health Action. 2019;12(sup1):1824383.
400. Lucas PJ, Uddin MR, Khisa N, Akter SMS, Unicomb L, Nahar P, et al. Pathways to antibiotics in Bangladesh: A qualitative study investigating how and when households access medicine including antibiotics for humans or animals when they are ill. PLoS One. 2019;14(11):e0225270.
401. Akhtar Z, Mah EMS, Rashid MM, Ahmed MS, Islam MA, Chowdhury S, et al. Antibiotics Use and Its Knowledge in the Community: A Mobile Phone Survey during the COVID-19 Pandemic in Bangladesh. Antibiotics. 2021;10(9).

402. Saha A, Marma KKS, Rashid A, Tarannum N, Das S, Chowdhury T, et al. Risk factors associated with self-medication among the indigenous communities of Chittagong Hill Tracts, Bangladesh. PLoS One. 2022;17(6):e0269622.
403. Azim MR, Ifteakhar KMN, Rahman MM, Sakib QN. Public knowledge, attitudes, and practices (KAP) regarding antibiotics use and antimicrobial resistance (AMR) in Bangladesh. Heliyon. 2023;9(10):e21166.
404. Islam MW, Shahjahan M, Azad AK, Hossain MJ. Factors contributing to antibiotic misuse among parents of school-going children in Dhaka City, Bangladesh. Sci Rep. 2024;14(1):2318.
405. Mannan A, Chakma K, Dewan G, Saha A, Chy N, Mehedi HMH, et al. Prevalence and determinants of antibiotics self-medication among indigenous people of Bangladesh: a cross-sectional study. BMJ Open. 2024;14(3):e071504.
406. Tamhankar AJ, Nachimuthu R, Singh R, Harindran J, Meghwanshi GK, Kannan R, et al. Characteristics of a nationwide voluntary antibiotic resistance awareness campaign in India; future paths and pointers for resource limited settings/low and middle income countries. International Journal of Environmental Research and Public Health. 2019;16(24):5141.
407. Chatterjee S, Hazra A, Chakraverty R, Shafiq N, Pathak A, Trivedi N, et al. A multicentric knowledge-attitude-practice survey in the community about antimicrobial use and resistance in India. Transactions of The Royal Society of Tropical Medicine and Hygiene. 2021;115(7):785-91.
408. Kotwani A, Joshi J, Lamkang AS, Sharma A, Kaloni D. Knowledge and behavior of consumers towards the non-prescription purchase of antibiotics: An insight from a qualitative study from New Delhi, India. Pharm Pract. 2021;19(1):2206.
409. Pattnaik M, Nayak AK, Karna S, Sahoo SK, Palo SK, Kanungo S, et al. Perception and determinants leading to antimicrobial (mis) use: A knowledge, attitude, and practices study in the rural communities of Odisha, India. Frontiers in public health. 2023;10:1074154.
410. Turankar TB, Gaidhane SA, Gaidhane AM, Sorte AG, Sawale SR. Comparison of health care professionals' and laypeople's knowledge, attitudes, and practices on the use of antimicrobial and antimicrobial resistance. Journal of Education and Health Promotion. 2023(1):313.
411. Gautham M, Bhattacharyya S, Maity S, Roy MB, Balasubramaniam P, Ebata A, et al. “Just as curry is needed to eat rice, antibiotics are needed to cure fever”—a qualitative study of individual, community and health system-level influences on community antibiotic practices in rural West Bengal, India. BMJ open. 2024;14(2):e076616.
412. Herawati F, Yulia R, Arifin B, Frasetyo I, Setiasih, Woerdenbag HJ, et al. Educational Video Improves Knowledge about Outpatients' Usage of Antibiotics in Two Public Hospitals in Indonesia. Antibiotics. 2021;10(5).
413. Karuniawati H, Hassali MAA, Suryawati S, Ismail WI, Taufik T, Hossain MS. Assessment of Knowledge, Attitude, and Practice of Antibiotic Use among the Population of Boyolali, Indonesia: A Cross-Sectional Study. Int J Environ Res Public Health. 2021;18(16).
414. Naurita M, Wibowo YI, Setiadi AP, Setiawan E, Halim SV, Sunderland B. Information on antibiotics in an Indonesian hospital outpatient setting: What is provided by pharmacy staff and recalled by patients? Pharm Pract. 2021;19(1):2167.
415. Yunita SL, Yang HW, Chen YC, Kao LT, Lu YZ, Wen YL, et al. Knowledge and practices related to antibiotic use among women in Malang, Indonesia. Front Pharmacol. 2022;13:1019303.
416. Sinuraya RK, Wulandari C, Amalia R, Puspitasari IM. Understanding Public Knowledge and Behavior Regarding Antibiotic Use in Indonesia. Infect Drug Resist. 2023;16:6833-42.
417. Miyano S, Htoon TT, Nozaki I, Pe EH, Tin HH. Public knowledge, practices, and awareness of antibiotics and antibiotic resistance in Myanmar: The first national mobile phone panel survey. PLoS One. 2022;17(8):e0273380.
418. Zawahir S, Lekamwasam S, Halvorsen KH, Rose G, Aslani P. Self-medication Behavior with antibiotics: a national cross-sectional survey in Sri Lanka. Expert Rev Anti Infect Ther. 2021;19(10):1341-52.
419. Miyazaki A, Tung R, Taing B, Matsui M, Iwamoto A, Cox SE. Frequent unregulated use of antibiotics in rural Cambodian infants. Trans R Soc Trop Med Hyg. 2020;114(6):401-7.
420. Lim JM, Chhoun P, Tuot S, Om C, Krang S, Ly S, et al. Public knowledge, attitudes and practices surrounding antibiotic use and resistance in Cambodia. JAC Antimicrob Resist. 2021;3(1):dlaa115.
421. Haenssgen MJ, Xayavong T, Charoenboon N, Warapikuptanun P, Khine Zaw Y. The Consequences of AMR Education and Awareness Raising: Outputs, Outcomes, and Behavioural Impacts of an Antibiotic-Related Educational Activity in Lao PDR. Antibiotics. 2018;7(4).

422. Haenssgen MJ, Charoenboon N, Zanello G, Mayxay M, Reed-Tsochas F, Lubell Y, et al. Antibiotic knowledge, attitudes and practices: new insights from cross-sectional rural health behaviour surveys in low-income and middle-income South-East Asia. BMJ Open. 2019;9(8):e028224.
423. Kounnavong S, Yan W, Sihavong A, Sychareun V, Eriksen J, Hanson C, et al. Antibiotic knowledge, attitudes and reported practice during pregnancy and six months after birth: a follow- up study in Lao PDR. BMC Pregnancy Childbirth. 2022;22(1):701.
424. Sychareun V, Phounsavath P, Sihavong A, Kounnavong S, Chaleunvong K, Machowska A, et al. Perceptions and reported practices of pregnant women and mothers of children under two years of age regarding antibiotic use and resistance in Vientiane province, Lao PDR: a qualitative study. BMC Pregnancy Childbirth. 2022;22(1):569.
425. Ha TV, Nguyen AMT, Nguyen HST. Public Awareness about Antibiotic Use and Resistance among Residents in Highland Areas of Vietnam. Biomed Res Int. 2019;2019:9398536.
426. McKinn S, Trinh DH, Drabarek D, Trieu TT, Nguyen PTL, Cao TH, et al. Drivers of antibiotic use in Vietnam: implications for designing community interventions. BMJ Glob Health. 2021;6(7).
427. Cai HTN, Tran HT, Nguyen YHT, Vu GQT, Tran TP, Bui PB, et al. Challenges and Lessons Learned in the Development of a Participatory Learning and Action Intervention to Tackle Antibiotic Resistance: Experiences From Northern Vietnam. Front Public Health. 2022;10:822873.
428. Di KN, Tay ST, Ponnampalavanar SSS, Pham DT, Wong LP. Socio-Demographic Factors Associated with Antibiotics and Antibiotic Resistance Knowledge and Practices in Vietnam: A Cross-Sectional Survey. Antibiotics. 2022;11(4).
429. Nguyen TTP, Do TX, Nguyen HA, Nguyen CTT, Meyer JC, Godman B, et al. A National Survey of Dispensing Practice and Customer Knowledge on Antibiotic Use in Vietnam and the Implications. Antibiotics. 2022;11(8):1091.
430. Ulaya G, Nguyen TCT, Vu BNT, Dang DA, Nguyen HAT, Tran HH, et al. Awareness of Antibiotics and Antibiotic Resistance in a Rural District of Ha Nam Province, Vietnam: A Cross-Sectional Survey. Antibiotics. 2022;11(12).
431. Cheng J, Coope C, Chai J, Oliver I, Kessel A, Wang D, et al. Knowledge and behaviors in relation to antibiotic use among rural residents in Anhui, China. Pharmacoepidemiol Drug Saf. 2018;27(6):652-9.
432. Diao M, Shen X, Cheng J, Chai J, Feng R, Zhang P, et al. How patients' experiences of respiratory tract infections affect healthcare-seeking and antibiotic use: insights from a cross-sectional survey in rural Anhui, China. BMJ Open. 2018;8(2):e019492.
433. Cheng J, Chai J, Sun Y, Wang D. Antibiotics use for upper respiratory tract infections among children in rural Anhui: children's presentations, caregivers' management, and implications for public health policy. J Public Health Policy. 2019;40(2):236-52.
434. Sun C, Hu YJ, Wang X, Lu J, Lin L, Zhou X. Influence of leftover antibiotics on self-medication with antibiotics for children: a cross-sectional study from three Chinese provinces. BMJ Open. 2019;9(12):e033679.
435. Wang J, Sheng Y, Ni J, Zhu J, Zhou Z, Liu T, et al. Shanghai Parents' Perception And Attitude Towards The Use Of Antibiotics On Children: A Cross-Sectional Study. Infect Drug Resist. 2019;12:3259-67.
436. Li P, Hayat K, Shi L, Lambojon K, Saeed A, Majid Aziz M, et al. Knowledge, Attitude, and Practices of Antibiotics and Antibiotic Resistance Among Chinese Pharmacy Customers: A Multicenter Survey Study. Antibiotics. 2020;9(4).
437. Xu Y, Lu J, Sun C, Wang X, Hu YJ, Zhou X. A cross-sectional study of antibiotic misuse among Chinese children in developed and less developed provinces. J Infect Dev Ctries. 2020;14(2):129-37.
438. Duan L, Liu C, Wang D. The General Population's Inappropriate Behaviors and Misunderstanding of Antibiotic Use in China: A Systematic Review and Meta-Analysis. Antibiotics. 2021;10(5).
439. Lin L, Harbarth S, Hargreaves JR, Zhou X, Li L. Large-scale survey of parental antibiotic use for paediatric upper respiratory tract infections in China: implications for stewardship programmes and national policy. Int J Antimicrob Agents. 2021;57(4):106302.
440. Luo Y, Tang X, Ding L, Shao Z, Yu J, Chen Y, et al. Non-prescriptionxx antibiotic use for people aged 15 years or older for cough in China: a community-based survey. Antimicrob Resist Infect Control. 2021;10(1):129.
441. Wu J, Yang F, Yang H, Zhang G, Mu K, Feng J, et al. Prevalence of antibiotic self-medication behavior and related factors among children aged 0 to 5 years. Expert Rev Anti Infect Ther. 2021;19(9):1157-64.
442. Yin X, Mu K, Yang H, Wang J, Chen Z, Jiang N, et al. Prevalence of self-medication with antibiotics and its related factors among Chinese residents: a cross-sectional study. Antimicrob Resist Infect Control. 2021;10(1):89.

443. Zhu Y, Tang X, Yan R, Shao Z, Zhou Y, Deng X, et al. Non-prescription antibiotic use for cough among Chinese children under 5 years of age: a community-based cross-sectional study. BMJ Open. 2021;11(12):e051372.
444. Zhou Z, Zhao D, Zhang H, Shen C, Cao D, Liu G, et al. Understanding parental self-medication with antibiotics among parents of different nationalities: a cross-sectional study. Glob Health Res Policy. 2021;6(1):42.
445. Yuan J, Du W, Li Z, Deng Q, Ma G. Prevalence and Risk Factors of Self-Medication Among the Pediatric Population in China: A National Survey. Front Public Health. 2021;9:770709.
446. Lin R, Duan L, Liu C, Wang D, Zhang X, Wang X, et al. The public's antibiotic use behavioural patterns and their determinants for upper respiratory tract infections: a latent class analysis based on consumer behaviour model in China. Front Public Health. 2023;11:1231370.
447. Qu W, Wang X, Liu Y, Mao J, Liu M, Zhong Y, et al. Self-Medication with Antibiotics Among Children in China: A Cross-Sectional Study of Parents' Knowledge, Attitudes, and Practices. Infect Drug Resist. 2023;16:7683-94.
448. Wang Q, Wu Y, Wang D, Lai X, Tan L, Zhou Q, et al. The impacts of knowledge and attitude on behavior of antibiotic use for the common cold among the public and identifying the critical behavioral stage: based on an expanding KAP model. BMC Public Health. 2023;23(1):1683.
449. Yan B, He Z, Dong S, Akezhuoli H, Xu X, Wang X, et al. The moderating effect of parental skills for antibiotic identification on the link between parental skills for antibiotic use and inappropriate antibiotic use for children in China. BMC Public Health. 2023;23(1):156.
450. Zhang T, Lin H, Zhao X, Wang W, Yan F, Lambert H. Influences on treatment-seeking and antibiotic use for common illnesses in eastern China. BMC Public Health. 2023;23(1):1849.
451. Choo SJ, Chang CT, Lee JCY, Munisamy V, Tan CK, Raj JD, et al. A cross-sectional study on public belief, knowledge and practice towards antibiotic use in the state of Perak, Malaysia. J Infect Dev Ctries. 2018;12(11):960-9.
452. Halim N, Chang CT, Chan HK, Hassali MA, Nouri A. Knowledge and Attitudes Concerning Antibiotic Use and Resistance among the Public in Pulau Pinang, Malaysia. Malays J Med Sci. 2018;25(6):141-7.
453. Irawati L, Alrasheedy AA, Hassali MA, Saleem F. Low-income community knowledge, attitudes and perceptions regarding antibiotics and antibiotic resistance in Jelutong District, Penang, Malaysia: a qualitative study. BMC Public Health. 2019;19(1):1292.
454. Aslam A, Zin CS, Ab Rahman NS, Gajdács M, Ahmed SI, Jamshed S. Self-Medication Practices with Antibiotics and Associated Factors among the Public of Malaysia: A Cross-Sectional Study. Drug Healthc Patient Saf. 2021;13:171-81.
455. Chang CT, Lee M, Lee JCY, Lee NCT, Ng TY, Shafie AA, et al. Public KAP towards COVID-19 and Antibiotics Resistance: A Malaysian Survey of Knowledge and Awareness. Int J Environ Res Public Health. 2021;18(8).
456. Kong LS, Islahudin F, Muthupalaniappen L, Chong WW. Knowledge and Expectations on Antibiotic Use Among the General Public in Malaysia: A Nationwide Cross-Sectional Survey. Patient Prefer Adherence. 2021;15:2405-16.
457. Thong KS, Chang CT, Lee M, Lee JCY, Tan HS, Shafie AA. Impact of targeted educational intervention towards public knowledge and perception of antibiotic use and resistance in the state of Perak, Malaysia. Antimicrob Resist Infect Control. 2021;10(1):29.
458. Wong LP, Alias H, Husin SA, Ali ZB, Sim B, Ponnampalavanar SSS. Factors influencing inappropriate use of antibiotics: Findings from a nationwide survey of the general public in Malaysia. PLoS One. 2021;16(10):e0258698.
459. Sneddon J, Drummond F, Guise T, Gilchrist M, Jenkins DR. Accreditation of antimicrobial stewardship programmes: addressing a global need to tackle antimicrobial resistance. JAC-Antimicrobial Resistance. 2024;6(1).
460. Ashiru-Oredope D, Garraghan F, Olaoye O, Krockow EM, Matuluko A, Nambatya W, et al. Development and Implementation of an Antimicrobial Stewardship Checklist in Sub-Saharan Africa: A Co-Creation Consensus Approach. Healthcare. 2022;10(9).
461. Etando A, Amu AA, Haque M, Schellack N, Kurdi A, Alrasheedy AA, et al. Challenges and Innovations Brought about by the COVID-19 Pandemic Regarding Medical and Pharmacy Education Especially in Africa and Implications for the Future. Healthcare. 2021;9(12).

462. Appiah B, Asamoah-Akuoko L, Samman E, Koduah A, Kretchy IA, Ludu JY, et al. The impact of antimicrobial resistance awareness interventions involving schoolchildren, development of an animation and parents engagements: a pilot study. Antimicrob Resist Infect Control. 2022;11(1):26.
